# Tuberculosis case fatality is higher in male than female patients in Europe: a systematic review and meta-analysis

**DOI:** 10.1007/s15010-024-02206-z

**Published:** 2024-03-23

**Authors:** Stephanie Pape, Sudip Jung Karki, Torben Heinsohn, Iris Brandes, Marie-Luise Dierks, Berit Lange

**Affiliations:** 1 Present Address: Institute for Epidemiology, Social Medicine and Health Systems, Hannover Medical School, Hannover, Germany; 2 Department of Epidemiology, Helmholtz Centre for Infection Research (HZI), Braunschweig, Germany; 3 German Center for Infection Research (DZIF), Braunschweig, Germany; 4 Present Address: Faculty of Medicine, Université Toulouse III Paul Sabatier, Toulouse, Occitanie France

**Keywords:** Europe, Humans, Sex factors, Male, Female, Tuberculosis

## Abstract

**Purpose:**

Epidemiological TB data indicate differences in infection prevalence, progression rates, and clinical disease incidence between sexes. In contrast, evidence on sex-specific differential (post) TB case fatality in Europe has not been synthesized systematically.

**Methods:**

We searched electronic databases and grey literature up to December 2020 for studies reporting sex-stratified TB death data for Europe. The JBI critical appraisal tools served for bias risk assessment and subgroup analyses for studying heterogeneity. Random-effects models meta-analyses enabled estimating pooled relative risks of sex-associated TB fatality. Considering associations of comorbidities and risk factors on fatality differences, we applied relative risk meta-regression.

**Results:**

Based on 17,400 records screened, 117 studies entered quantitative analyses. Seventy-five studies providing absolute participant data with moderate quality and limited sex stratification reported 33 to 235,000 TB cases and 7 to 27,108 deaths. The pooled male-to-female TB fatality risk ratio was 1.4 [1.3–1.5]. Heterogeneity was high between studies and subgroups. Study time, concurrent comorbidities (e.g., HIV, diabetes, cancers), and mean participant ages showed no effect modification. We identified higher male TB fatality in studies with higher homelessness (coefficient 3.18, 95% CI [-0.59 to 6.94], *p-*value 0.10) and lower migrants proportion (coefficient − 0.24, 95% CI [− 0.5 to 0.04], *p*-value 0.09).

**Conclusion:**

We found 30–50% higher TB case fatality for males in Europe. Except for homelessness, migration, and a trend for some comorbidities, assessing effect modification could not reduce our meta-analysis’ high heterogeneity. Public health authorities should take heed of this higher risk of dying in male patients’ treatment services.

**Supplementary Information:**

The online version contains supplementary material available at 10.1007/s15010-024-02206-z.

## Introduction

Tuberculosis (TB) is the most common bacterial infectious disease and the leading cause of death from a single infectious agent in humans [1]. The association between latent TB infection (LTBI) or TB disease and the presence of socio-demographic and other risk factors is well described. These include but are not limited to origin or nationality, medical comorbidities such as diabetes or co-infection with HIV and hepatitis viruses, risk factors like alcohol, drug consumption, and nicotine dependence, previous prison stays, and, last but not least, sex [2–20].

Globally, adult males show higher rates of TB than females. TB notification rates in most countries are generally 1.6- to 2.7-fold higher for HIV-negative men than for HIV-negative women [2]. Regardless of ethnicity or geographic location, epidemiologic data for TB indicate sex differences in infection prevalence, progression rates, clinical disease incidence, and morbidity and mortality rates at the expense of men [1].

The cause of this sex bias has not yet been fully elucidated; major influences include socio-economic and behavioral factors as well as underreporting of female cases, access to health care, but also potential genetic differences regarding susceptibility for tuberculous infection [2]. Lifetime prevalence estimates of self-reported TB in one of the largest population-based cohorts in Germany are not highly different for men and for women, which—in addition to potential sex-differentiated information and selection bias in this cohort study—could be an effect of shorter survival of men with TB [21]. Well-designed systematic analyses of differential TB case fatality for men and women in Europe are, therefore, critical but limited [4, 19, 20].

We, therefore, systematically assessed evidence of differences in TB case fatality between men and women in Europe and investigated additional characteristics modifying these differences.

There are different data sources available that deliver TB death information. Mortality data (about deaths from TB in HIV-negative persons) are usually derived from national vital registrations comprising death certificates based on ICD-10 or former ICD classification codes. The current ICD-10 codes for TB are A15 to A19, and B-90 for TB sequelae. Deaths of HIV-positive persons with TB comorbidities are usually coded for HIV as death cause [22].

TB fatality can be estimated based on the WHO treatment outcome definitions [23]. Many studies use TB registers/surveillance data as common sources for fatality estimations.

Usually, cohort studies follow-up on TB patients after treatment, either reporting mortality (mostly deaths of any cause compared with the general population) or fatality in the post-TB cohort.

## Methods

We conducted a systematic review and meta-analysis according to the PRISMA guidelines [24] to summarize current evidence on differences in TB case fatality between men and women in Europe and factors influencing these differences (PROSPERO (CRD42021224045)).

The target disease was active TB without further restrictions, which included persons with active TB or former TB disease for whom sex-specific disaggregated or aggregated TB and death data were available. Non-binary definitions of sex or intersexuality were not considered due to a lack of data on these categories.

The main outcome was during- and post-TB case fatality estimates, differentiated by sex. Relative risks served as measures of effect within all analyses. Insufficient data about extra-pulmonary TB (EPTB) cases did not allow stratified analyses for the type of active TB.

### Search strategy and selection criteria

We searched Medline, Pubmed, Embase, the Global Health databases, the Cochrane Library, and the grey literature for the European region, as defined by the WHO, up to December 11, 2020, without further time restrictions (Online Resource 1).

Records were included if they contained sex-specific TB death data without any restrictions to specific sites of TB disease (Online Resource 2). Eligible studies had to report during- or post-TB deaths of the general population within the WHO region Europe (Online Resource 3), representing information for both sexes. Furthermore, the studies had to provide sufficient information to calculate the relative risks of male and female TB case fatality and the respective risk ratios. We included observational studies, such as prevalence, cross-sectional studies, cohort and case–control studies, intervention studies, e.g., (randomized) controlled therapy studies, and diagnostic and prognostic studies. No restrictions were in force regarding time, language, or TB stage (exposure, latent, and active disease) or sources of TB data. We accepted TB death information from vital registration systems and TB surveillance/notification records, but also from another origin.

Due to a large number of records to screen, one author (SP) reviewed all titles and abstracts, while a second author (SJK) independently reviewed a random selection of 1,000 records for inclusion. Two authors (SP and TH) independently and completely screened all records eligible for full-text screening based on the before-mentioned criteria. The online tool Rayyan was used for screening [25]. Discrepancies were resolved and agreed upon in a consensus meeting. A third reviewer was available for the resolution of unsolved conflicts. The references excluded during full-text screening were listed, stating the reasons for exclusion according to the PRISMA guidelines [24].

### Data extraction

Applying a standardized pre-tested data extraction tool, SP extracted bibliographic, clinical, and demographic information (Online Resource 4). The TB incidence and death data retrieved for males and females from all publications included in quantitative analyses set the ground for meta-analyses of the entire dataset, stratified by reported outcome (dependent variable) and subgroups concerning sex differences in TB deaths in Europe. Considering the insufficient sex-stratified information concerning TB disease, deaths, comorbidities, and risk factors, we decided to merge the studies, despite their heterogeneous characteristics, by the reported effect estimates and investigate heterogeneity through subgroup and sensitivity analyses. Due to the restricted number of publications reporting outcomes other than absolute numbers of TB cases and TB deaths, subgroup analyses occurred in the studies providing absolute numbers exclusively.

### Risk of bias assessment

The Joanna Briggs Institute Critical Appraisal Tools (JBI CAT) were used for quality assessment for all included studies published in 1990 or later covering the 1990s upwards (Online Resource 5) [26].

### Data analysis

Random-effects meta-analyses and subgroup analyses were performed on the web server Onlinemeta v1.0: 2022.3.15, Update: 2022.10.27. JASP 0.16.3-Debug and Rstudio Cloud version 4.2.1 were applied to conduct meta-regressions [27–29]. The robvis web app served as a tool for the risk of bias visualization [30]. Krippendorff’s alpha was calculated to assess the reliability of inclusion decisions and the risk of bias assessment between the two independent investigators [31].

### Random-effects meta-analyses of sex-associated TB fatality

Based on the data extracted, we tabulated TB cases and deaths, stratified by study and sex, and calculated individual and pooled male-to-female risk ratio estimates and their 95% confidence intervals (CI). If studies provided death information in a data format other than absolute numbers, e.g., (standardized) mortality and risk rates, hazard or odds ratios, these effect sizes and 95% CIs were estimated separately (Online Resource 6).

The risk of publication bias investigation applied the funnel plot function and trim-and-fill method on the Onlinemeta web server.

Concerning heterogeneity, we conducted subgroup analyses based on the study characteristics. Investigating heterogeneity from a clinical perspective did not occur due to insufficient reporting of medical information regarding the various study populations. We studied heterogeneity visually in the forest plots and based on the *χ*^2^, *I*^2^, *τ*^2^, and *τ* statistics.

Considering sensitivity analyses, we excluded eight studies with effect size estimates higher than 2.0 from the full dataset and repeated all before-mentioned analyses with this restricted, “outlier-cleaned” dataset (Online Resource 7).

### Meta-regression

We used relative risk regression methods provided by the R package ‘metafor’ v3.8-1 to estimate the effects of comorbidities, socio-demographics, and risk factors on the TB fatality risk ratio between males and females [32]. The estimations relied on the proportions of each comorbidity, socio-demographic, or risk factor for each study population with sufficient data. Based on that, variables with ten or more data values entered univariate meta-regression analyses using a mixed-effects model with one moderator and the restricted maximum likelihood (REML) method for *τ*^2^ and *I*^2^ estimation considering heterogeneity [33]. Univariate and multivariable meta-regression models exclusively included continuous moderators (Online Resource 8).

## Results

### Systematic review

**Fig. 1:**
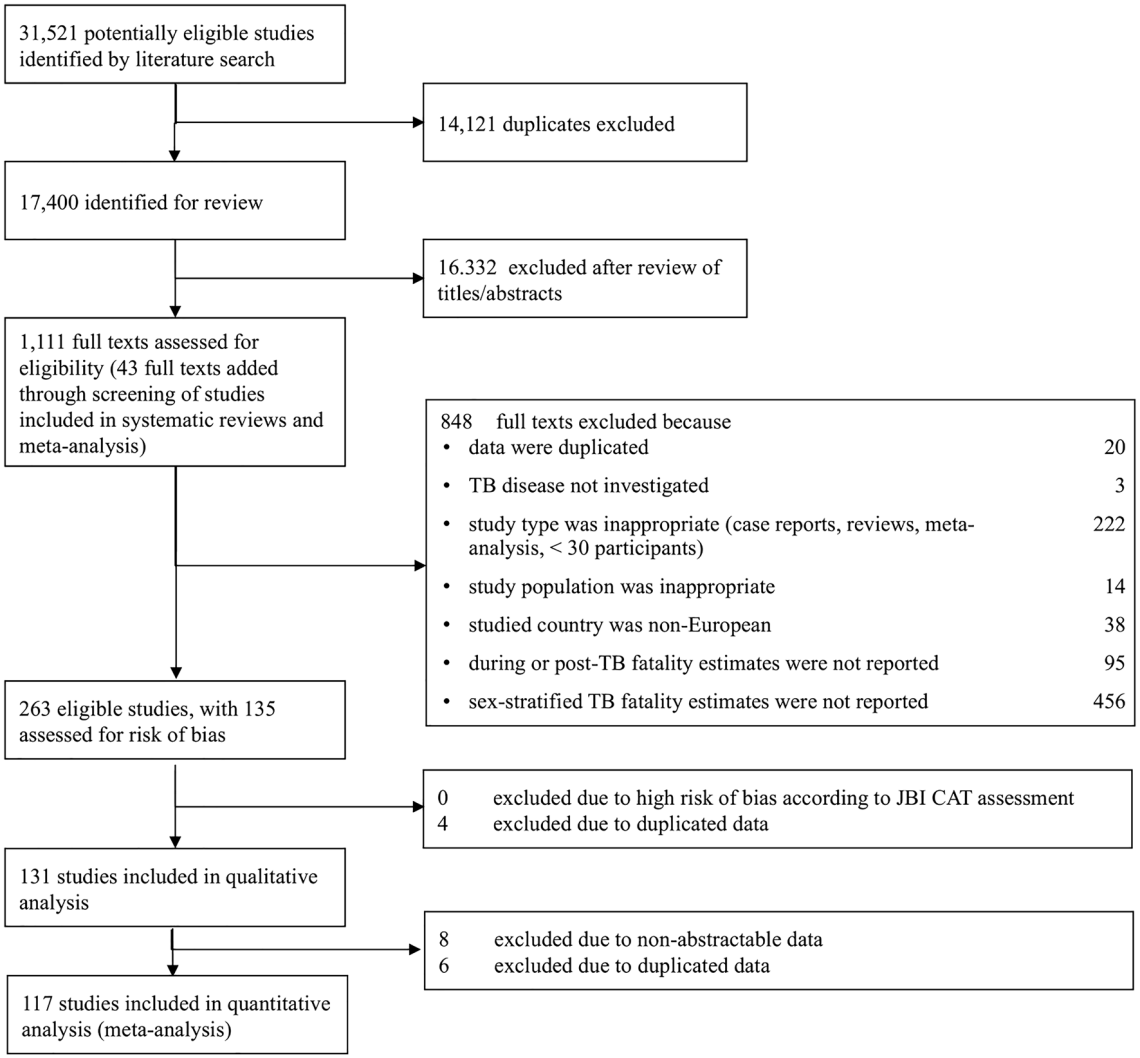
PRISMA flow chart

The database search identified 17,400 records, of which 16,332 were excluded during the titles and abstract review. Of 1,111 entries eligible for a full-text review, 848 were excluded, leaving 263 articles for qualitative assessment (Fig. 1).

The studies were published between 1904 and 2020, covering an observation period from 1786 to 2019. The time observed by each study ranged from 1 to 120 years (Online Resource 9). Based on these characteristics, we decided to focus the investigations on those studies published in 1990 or later with an observation period covering the 1990s up to the literature search date, resulting in 135 studies for further qualitative analyses (Online Resource 10). Briefly, the evidence body covered studies published between 1997 and 2020, with an observation period from 1980 to 2019, and reporting sex-related TB death data from several European regions and individual member states of the WHO region Europe. About one-fourth of the studies (*n* = 32) observed only 1 year, whereas the remaining publications covered 2–18 years, with one outlier reporting over 24 years (Online Resource 11). Among the countries contributing six or more studies were France, the Netherlands, Italy, Spain, the United Kingdom, Germany, Russia, and Poland. Austria, Greece, Hungary, and Iceland were no longer included in the dataset (Online Resource 12). Apart from vital statistics, the studies included in this review used notification data and information derived from hospitals and TB dispensaries as data sources. Most included studies focused on treatment outcomes, risk factors, and mortality investigations, mostly TB specific and independent from the study population or setting. Only two publications [34, 35] addressed sex-stratified mortality analysis in their objectives. Further information can be found in the Online Resource 13.

The risk of bias in the 131 publications assessed was moderate because of quality issues concerning the study population recruitment, patient flow, follow-up, exposure measurement, confounder identification, and control strategies (Online Resource 14).

The inter-rater reliability of the title/abstract screening, calculated as Krippendorff’s alpha, was *α* = 0.280 (SE 0.074; 95% CI [0.128–0.420]).^1^ All disagreements could be fully resolved.

After data extraction, 117 publications remained for meta-analyses reporting the sex-related TB death data as different effect estimates, mostly in absolute numbers (92 publications) (Online Resource 15). Regarding the absolute numbers dataset, we introduced the regional datasets provided by the Global Burden of Disease (GBD) Study 2014 [36] as separate entries, increasing the publication number from 92 to 94. Notably, the TB definition of the GBD study included the group of TB sequelae, which in many countries represents the majority of TB-related deaths.

Apart from that, the systematic review did not identify any studies reporting a diagnosis of non-communicable diseases or other sequelae after recovery from TB in the context of TB deaths of males and females in Europe.

### Meta-analyses of individual effect estimates of sex-stratified TB fatality

The available data enabled estimating and comparing the male-to-female TB fatality ratio estimates for each country or subnational region but not differentiating during- and post-TB events. By applying a random-effects model, we conducted separate meta-analyses of each reported effect size type except for risk ratios, and survival time (*n* = 3). The regression tests of asymmetry in the funnel plots did not indicate publication bias for the sex-stratified TB fatality risks within the final datasets analyzed (Online Resource 16).

Ninety-four studies provided absolute numbers for relative risk calculation (Table 1, Online Resource 17). We agreed to exclude another two records [37, 38] from the absolute numbers dataset due to data similarities and duplication concerns. As the remaining dataset included 17 publications (Online Resource 18) exclusively focusing on TB death or autopsy cases resulting in male-to-female fatality ratios with the value “1,” we decided to separate these studies and perform the main meta-analysis with a limited dataset (*n* = 75; including 29 cohort studies).

**Table 1:** Results of the sensitivity analyses (random-effects model meta-analyses)

Category	Overall estimates (random-effects model)						Test for overall effect		Heterogeneity				
Reported study outcome	No. studies	Males (*n*)	Females (*n*)	Ratio *M*/*F*	CI 95% lower	CI 95% upper	*Z* value	*p*	*χ*^2^	*df*	*p*	*I*^2^ (%)	*τ*^2^
Absolute numbers (main dataset)	*75*	*680,494*	*363,428*	*1.391*	*1.299*	*1.490*	*9.42*	< *0.01*	*943.47*	*74*	< *0.01*	*92*	*0.05*
Absolute numbers (restricted dataset)	67	679,080	362,950	1.375	1.283	1.474	9.01	< 0.01	935.36	66	< 0.01	93	0.049
Absolute numbers	94	697,645	371,717	1.356	1.071	1.716	2.53	0.01	1,316,275.90	93	0	100	1.270
Absolute numbers (w/o autopsy studies)	77	686,419	367,947	1.396	1.305	1.493	9.71	< 0.01	948.08	76	< 0.01	92	0.049
Hazard ratios	3	n/a	n/a	1.512	1.075	2.127	2.38	0.02	0.52	2	0.77	0	0
Mortality rates	15	392.23	184.08	1.467	0.881	2.441	1.47	0.14	0.63	14	1.00	0	0
Odds ratios	7	n/a	n/a	1.312	1.174	1.466	4.79	< 0.01	2.38	6	0.88	0	0
Mortality rates^a^	17	n/a	n/a	2.565	1.995	3.298	7.34	< 0.01	667.89	16	< 0.01	98	0.247
Mortality rates^a^	15	n/a	n/a	3.470	3.075	3.915	20.19	< 0.01	48.80	14	< 0.01	71	0.039
Standardized mortality rates^a^	3 (7 by sub-studies)	n/a	n/a	6.188	4.686	8.171	12.85	< 0.01	212.10	6	< 0.01	97	0.131

*M/F* male-to-female ratio, *CI 95%* 95% confidence interval, *p p* value, *df* degrees of freedom, *χ*^*2*^ Chi^2^ test value, *I*^*2*^* (%)* *I*^2^ test value, *τ*^*2*^ *τ*^2^ test value, *Z value* *Z* test value, *IR* incidence rate, *n* absolute numbers, *n/a* not applicable

^a^The calculations were performed without TB incidence rates serving as denominators

Italics value indicates significance level ∝ = 0.05

Meta-analysis was not applicable for three studies with outcomes other than the before-mentioned effect sizes facing missing outcome similarity or retraction [39–41].

### Main analysis

**Fig. 2:**
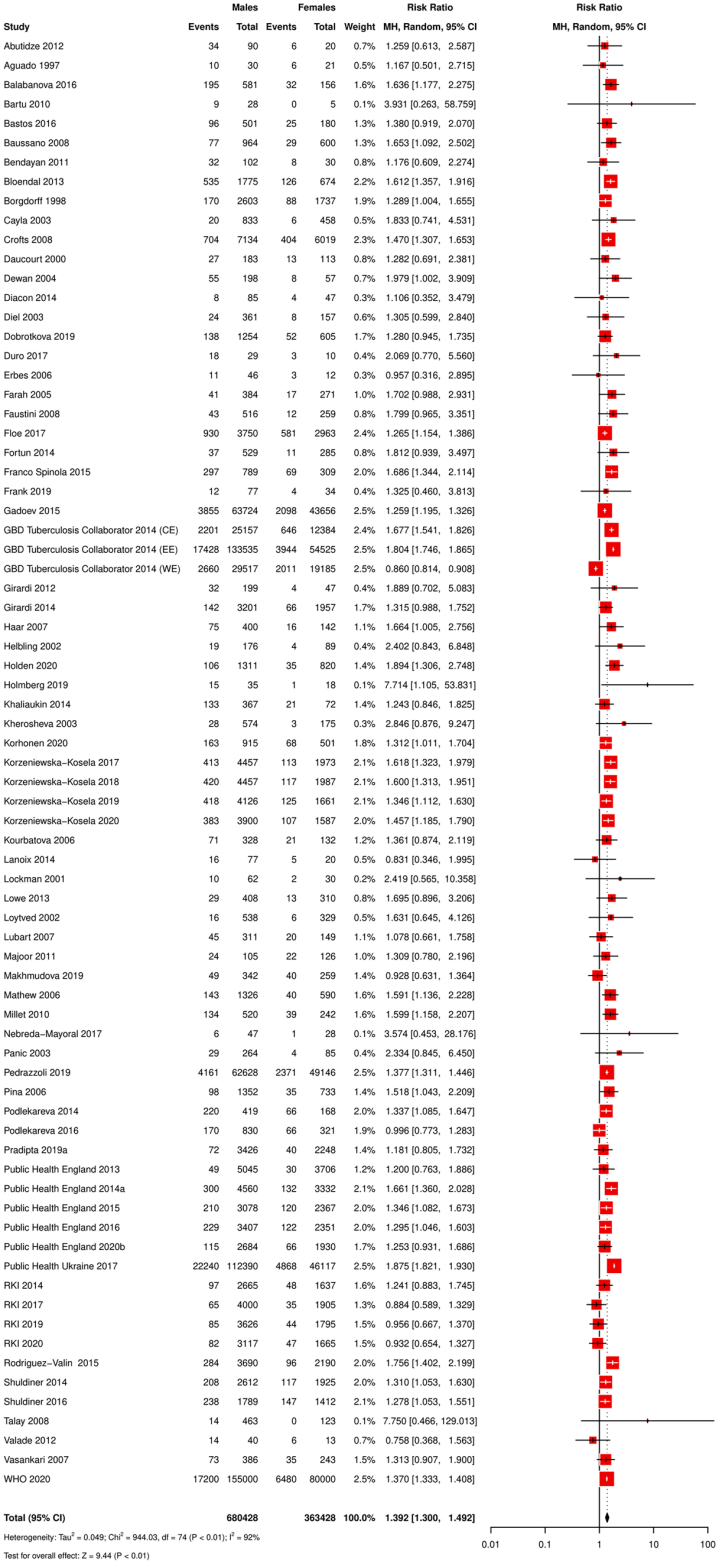
Forest plot of publications reporting absolute numbers (*n* = 75)

The 75 studies providing fatality data in absolute numbers comprised study populations of 33 to 235,000 TB cases and 7 to 27,108 death cases. The meta-analysis found a pooled male-to-female TB fatality risk estimate of 1.4 [1.3–1.5], with individual estimates ranging from 0.8 [0.4–1.6] to 7.8 [0.5–129.0] (Fig. 2). The pooled results of the full (*n* = 94) and the limited dataset (*n* = 75) did not differ remarkably. However, the full dataset came with a broader confidence interval (Table 1). The *χ*^2^, *I*^2^, and *τ*^2^ tests showed high heterogeneity between the sex-associated relative risks of TB fatality derived from the primary studies (Table 1, Online Resource 19).

We conducted subgroup analyses to explore heterogeneity based on study characteristics for the relative TB fatality estimates derived from absolute numbers (dataset *n* = 75).

Given the possible impact of different study designs on the effect estimates, the respective subgroup analysis presented a higher TB death risk for males throughout all designs (case–control, cohort, cross-sectional, descriptive, and interventional studies), covering pooled estimates from 1.1 [0.4–3.5] to 1.5 [1.3–1.8]. Concerning heterogeneity, the cohort study subgroup (male-to-female RR = 1.4 [1.3–1.4]) indicated the lowest variance in true effects related to the observed effects variance (*I*^2^ = 15%, *df* = 28, *p* = 0.24) (Online Resource 19).

To investigate the sex-stratified TB fatality by time and region, we grouped the studies in four regions (Europe, Central, Eastern, and Western Europe) and seven observation periods (1980s–1990s, 1990s, 1990s–2000s, 1990s–2010s, 2000s, 2000s–2010s, 2010s). The regional subgroup estimates reached from 1.2 [0.9–1.6] (Europe) to 1.6 [1.4–1.7] (Central Europe). The estimates of the subgroup analysis by time ranged from 1.2 [0.5–2.7] (1980s–1990s) to 1.6 [1.4–1.9] (1990s–2000s). Both analyses indicated relevant subgroup differences (Online Resources 19–21).

### Additional analyses

Apart from the main meta-analysis (*n* = 75), we performed sensitivity analyses with the remaining datasets and an outlier-restricted dataset of the main study (*n* = 67) (Table 1). The pooled estimate of the latter dataset (*n* = 67) was similar to the result of the main analysis.

Studies reporting hazard ratios (HR) (*n* = 3; all being cohort studies), odds ratios (OR) (*n* = 7; five of them being cohort studies), and TB mortality plus incidence rates (*n* = 15) resulted in pooled effect estimates between 1.3 [1.2–1.5] and 1.5 [0.9–2.4].

When restricting the dataset to (standardized) mortality rates (SMR) (Online Resources 22–24), the pooled relative risk of TB mortality in males compared to females was notably higher for SMR (6.2 [4.7–8.2]) (Online Resource 24), compared to non-standardized mortality rates (2.6 [2.0–3.3]) (Online Resource 22). However, the SMR results were mainly dictated by studies from Russia from the 1990s to 2010.

Throughout all meta-analyses, most primary studies estimates indicated a higher risk of dying for males, but not the Western European subgroup of the GBD Tuberculosis Collaborator publication (2014), comprising active TB cases and those with TB sequelae [36] (male-to-female RR = 0.862 [0.81–0.91]) (Table 2).

**Table 2:** Studies reporting higher TB deaths for females

Absolute numbers							
Study (country/region)	Events (*M*)	Total (*M*)	Events (*F*)	Total (*F*)	Risk ratio *M*/*F*	CI 95% lower	CI 95% upper
Erbes 2006 (Germany)	11	46	3	12	0.957	0.316	2.895
GBD Tuberculosis Collaborator 2014 (Western Europe)	2660	29,517	2011	19,185	0.860	0.814	0.908
Lanoix 2014 (France)	16	77	5	20	0.831	0.346	1.995
Makhmudova 2019 (Tajikistan)	49	342	40	259	0.928	0.631	1.364
Podlekareva 2016 (16 European countries)^a^	170 (EE: 160; WE: 10)	830 (EE: 626; WE: 204)	66 (EE: 63; WE: 3)	321 (EE: 208; WE: 113)	0.996 (EE: 0.844; WE: 1.846)	0.773 (EE: 0.660; WE: 0.519)	1.283 (EE: 1.079; WE: 6.572)
RKI 2017 (Germany)	65	4000	35	1905	0.884	0.589	1.329
RKI 2019 (Germany)	85	3626	44	1795	0.956	0.667	1.370
RKI 2020 (Germany)	82	3117	47	1665	0.932	0.654	1.327
Valade 2012 (France)	14	40	6	13	0.758	0.368	1.563

Odds ratio							
Study (country/region)	Events (*M*)	Total (*M*)	Events (*F*)	Total (*F*)	Odds ratio *M*/*F*	95% CI lower	95% CI upper
Aibana 2018 (Ukraine)	n/a	n/a	n/a	n/a	0.850	0.371	1.946
Cayla 2009 (Spain)	n/a	n/a	n/a	n/a	0.980	0.442	2.171

*Events* TB deaths, *total* all individuals diagnosed with TB, *M* males, *F* females, *risk ratio M/F* male-to-female risk ratio (relative risk), *odds ratio M/F* male-to-female odds ratio, *CI 95%* 95% confidence interval, *EE* Eastern Europe, *WE* Western Europe

^a^16 European countries (Western Europe (*n* = 317): Belgium, Denmark, France, Italy, Spain, Switzerland, and the UK. Eastern Europe (*n* = 834): Belarus, Estonia, Georgia, Latvia, Lithuania, Poland, Romania, Ukraine, and Russia)

### Factors modifying the association between sex and TB fatality

**Fig. 3:**
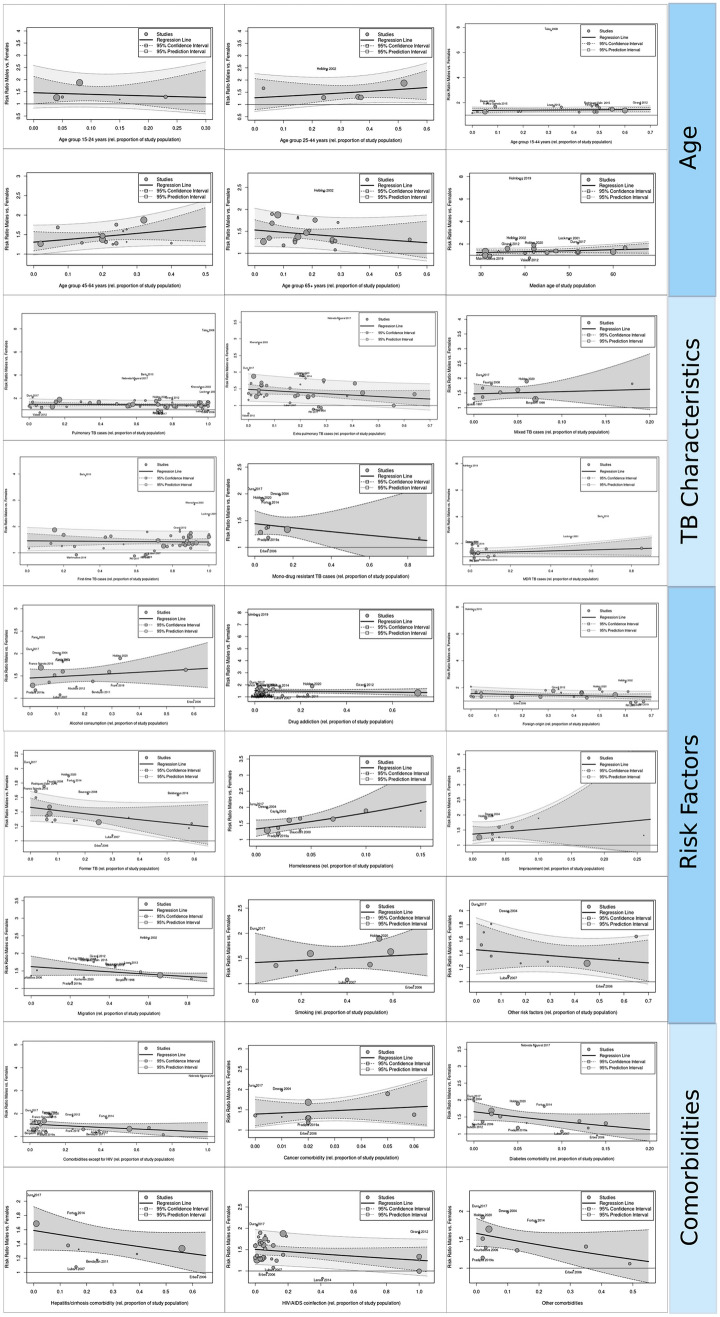
Bubble plots of univariate meta-regression models

Based on the dataset derived from studies reporting absolute numbers (*n* = 75), we found a tendency for higher risk ratios of TB fatality for males in those studies with a higher proportion of homeless persons. That means a 10% increase in *homelessness* within a study population let the male-to-female TB fatality rise by 0.3 to the extent of men (coefficient 3.18, 95% CI [− 0.59 to 6.94], *p*-value 0.10). The same applied in those studies with a lower proportion of *migrants*. In contrast to homelessness, a 10% increase in migrants within a study population let the male-to-female TB fatality shrink by 0.02, favoring women (coefficient − 0.24, 95% CI [− 0.5 to 0.04], *p*-value 0.09) (Fig. 3, Online Resources 25–27). Also, there was a tendency in those studies with a higher proportion of persons with comorbidities to report lower risk ratios of TB fatality for men than in those studies with lower proportions of comorbidities, in particular for diabetes, hepatitides/cirrhosis, and other comorbidities.

Mixed-effects univariate meta-regression did not reveal any effect modification of the covariates *age 15–24, age 15–44, age 45–64, age 65* +*, median age, foreign origin, new cases, EPTB, PTB, mixed TB, HIV/AIDS, diabetes, cancers, hepatitis/cirrhosis, other comorbidities, any comorbidity exc. HIV, any comorbidity, alcohol abuse, smoking, drug addiction, former TB, imprisonment, other risk factors, MDR-TB,* and *mono-drug-resistant TB* (Fig. 3, Online Resources 28–29).

## Discussion

### Main findings

Our work aimed to investigate sex-associated differences in TB fatality for the entire WHO region of Europe. Based on absolute numbers (dataset *n* = 75), we calculated a pooled male-to-female TB fatality risk estimate of 1.4 [1.3–1.5]. Our meta-regression only identified a few modifying factors of this excess fatality.

Our findings align with the meta-analysis by Romanowski and colleagues comprising TB data from the UK, Denmark, USA, India, Israel, Estonia, Ethiopia, China, and Vietnam [42]. Based on 10 publications comprising 40,781 post-TB patients and 6922 deaths globally, they found a higher SMR of 2.94 (95% CI 1.96–4.42) for men compared to women (SMR 2.33 (95% CI 1.59–3.41)). That extends our findings as our evidence body was not restricted to post-TB patients. It shows that accounting for sex-specific risk of dying is relevant for both during-TB and post-TB.

The most recent Global Burden of Disease Study (GBD) concerning TB focused on sex differences in TB burden on a global, regional, and national scale from 1990–2019, stratified by HIV status [43]. Based on vital registration, verbal autopsy, and surveillance, the GBD 2019 Tuberculosis Collaborators report sex-stratified incident cases and deaths in Central, Eastern, and Western Europe, comprising populations with active TB (ICD10: A15-19) or sequelae of TB (B90). For 2019, they found Armenia, Georgia, Ukraine, and Moldova among the states with the highest male-to-female ratios in age-standardized TB-associated mortality rates.

We conducted a random-effects model meta-analysis of the sex-associated relative risk of dying based on the GBD 2019 data of the female and male incident TB cases and deaths for Central, Eastern, and Western Europe. That provided a pooled male-to-female relative risk estimate (95% CI) of 1.27 [1.04–1.55] (Online Resource 30). In addition, this meta-analysis reaffirmed the findings of a higher risk for females in the Western European subgroup of the Global Burden of Disease Tuberculosis Collaborator publication (2014) [36] (M:F RR = 0.86 [0.81–0.91]).

Concerning the impact of comorbidities and risk factors on sex-associated TB fatality in Europe, our meta-regression (dataset *n* = 75) showed some evidence for effect modification of the proportion of *homelessness* and *migrants* (Online Resource 27) [13, 15, 16, 44–46]. Romanowski et al. found cardiovascular disease as an effect modifier for higher TB mortality in 4426 post-TB treatment deaths, whereas we mainly saw trends but no strong evidence of effect modification of comorbidities in our synthesis. Similarly, our investigation could not identify a large further range of modifying factors, which principally suggests a determining biological factor for the different TB deaths between men and women [18]. The involvement of the X chromosome and X inactivation in the immune system and its role in the sexual dimorphism of infectious diseases was the subject of a 2019 review [2], using TB susceptibility as an example. The authors concluded that the X chromosome and its inactivation are involved in TB susceptibility and male sexual dimorphism.

### Limitations

The review’s strengths rely on the broad search strategy regarding the search terms applied and language acceptance. Our literature search targeted primary research evidence. However, our research is prone to various weaknesses.

We found a high extent of heterogeneity regarding study design, regions covered, groups of populations included, and periods of observation. We decided to still provide pooled estimates for the comparison of fatality and mortality of males and females and made high efforts to assess the effect of this heterogeneity further in subgroup analysis and meta-regression. This allowed us to provide relative risk estimates for sex-associated TB death risks for the WHO region Europe.

The information abstracted from the studies included in the review was characterized by a high degree of missing data, in general, and stratified by sex concerning all covariates defined in the review protocol. Therefore, we could not estimate and compare the male-to-female ratio differentiated in during- and post-TB death estimates in total or within age groups or other strata.

We also could not differentiate the time point of death during or after recovery from active TB disease due to a lack of information provided by the publications. Thus, we were not able to assess “fatality” in the post-TB cohort properly. The same applied to the underlying cause of TB fatality. We could not determine in any publication over the entire range of studies whether the underlying cause of death was due to TB, with TB, or due to another cause. Therefore, our reporting relied on unknown causes.

## Conclusion

Our evidence synthesis revealed higher TB case fatality in men compared with women in Europe. We identified a few effect modifiers for this. Public health services should take heed of this higher death risk in males and adjust their programs concerning screening, diagnosis, and TB treatment.

A lack of sex and gender-stratified information regarding socio-demographics, comorbidities, and risk factors limited our findings. Future primary studies in TB research in Europe should focus on sex- and gender-related aspects of TB mortality and fatality to promote empirical evidence synthesis and informed health intervention decision-making in this field [47–49]. Undoubtedly, this approach will essentially contribute to the objectives of the WHO’s End TB Strategy [50].

## Supplementary Information

Below is the link to the electronic supplementary material.

**Online Resource 1** Literature search – search strategies (PDF 195 KB)

**Online Resource 2** Inclusion and exclusion criteria applied in the systematic review (PDF 183 KB)

**Online Resource 3** Countries of the WHO region of Europe (by May 30, 2020) (PDF 184 KB)

**Online Resource 4** Data extracted from the publications (PDF 219 KB)

**Online Resource 5** JBI Critical Appraisal Tools (JBI CAT) templates (XLSX 53 KB)

**Online Resource 6** Data files used for meta-analyses (PDF 281 KB)

**Online Resource 7** Publications with male-to-female relative risk estimates of TB fatality higher than 2.0 (PDF 194 KB)

**Online Resource 8** Variables encompassed by the meta-regression dataset and final meta-regression dataset (PDF 218 KB)

**Online Resource 9** Summarized study characteristics of the 263 publications included in the systematic review (PNG 117 KB)

**Online Resource 10** Summarized study characteristics of the 135 publications designated for risk of bias assessment (PNG 99 KB)

**Online Resource 11** Observation periods covered by the publications designated for risk of bias assessment (PNG 19 KB)

**Online Resource 12** Countries included in the risk of bias assessment and the number of publications, stratified by country (PNG 78 KB)

**Online Resource 13** Characteristics overview of the 131 studies included in quantitative analyses (PDF 236 KB)

**Online Resource 14** Quality assessment results, stratified by study type (PDF 759 KB)

**Online Resource 15** Overview of the effect estimates reported by the publications included in quanitative analysis (PDF 26 KB)

**Online Resource 16** Study characteristics of the publications included in quantitative analysis, their categories used in the subgroup analyses, plus results of the publication bias assessment (PDF 188 KB)

**Online Resource 17** Interim meta-analysis results of 94 studies providing absolute numbers for relative risk calculation (PDF 14 KB)

**Online Resource 18** Listing of the 17 publications exclusively reporting TB death or autopsy cases (PDF 186 KB)

**Online Resource 19** Results of the subgroup analyses for the publications reporting absolute numbers (full dataset n = 75) (XLSX 13 KB)

**Online Resource 20** Forest plot of publications reporting absolute numbers (n = 75), stratified by regions (PDF 14 KB)

**Online Resource 21** Forest plot of publications reporting absolute numbers (n = 75), stratified by observation period (PDF 14 KB)

**Online Resource 22** Forest plot of publications reporting death cases in absolute numbers (n = 17) (PDF 7 KB)

**Online Resource 23** Forest plot of publications reporting mortality rates (MR), calculated by absolute numbers (n = 15) (PDF 7 KB)

**Online Resource 24** Forest plot of publications reporting standardized mortality rates (SMR) (PDF 6 KB)

**Online Resource 25** Bubble plot of moderator homelessness (univariate meta-regression) (PDF 30 KB)

**Online Resource 26** Bubble plot of moderator migration (univariate meta-regression) (PDF 30 KB)

**Online Resource 27** Univariate meta-regression – Moderator forest and bubble plots and prediction results (PDF 1472 KB)

**Online Resource 28** Results of univariate mixed-effects meta-regression models (ln scale, reference: females) (XLSX 11 KB)

**Online Resource 29** Multivariable meta-regression results (incl. R code) (PDF 269 KB)

**Online Resource 30** Forest plot of the male: female relative risk estimates derived from the Global Burden of Disease 2019 (PNG 229 KB)

**Online Resource 31** Listing of the random sample of 14 publications for risk of bias assessment (by a second reviewer) (PDF 185 KB)

## Data Availability

Most data generated or analyzed during this study are included in this published article [and its supplementary information files]. The remaining unpublished datasets used and/or analyzed during the current study are available from the corresponding author upon reasonable request.

## Footnotes

1 Two raters/measurements and one thousand and two subjects/items. Confidence intervals are based on percentiles from 1,000 bootstrap replications; calculated with JASP.
