# Supplementary material for: Tuberculosis case fatality is higher in male than female patients in Europe: a systematic review and meta-analysis
Source: Infection. 2024 Mar 23;52(5):1775–86. doi: 10.1007/s15010-024-02206-z (PMC11499538; doi:10.1007/s15010-024-02206-z)
Supplement: Supplementary file 1 — Online Resource 1 Literature search – search strategies (PDF 195 KB) [file 15010_2024_2206_MOESM1_ESM.pdf]

Search Strategies

I. Pubmed/Medline search strategy

		PubMed/Medline
Concept	#	Search terms
TB	1	((“tuberculosis”[MeSH Terms] OR “tuberculosis” OR “Tuberculoses”) OR (“Mycobacterium tuberculosis”[MeSH terms])) NOT ((“animals”[MeSH Terms] NOT (“humans”[MeSH Terms] AND “animals”[MeSH Terms]))
Gender	2	((((((((sex[MeSH Terms]) OR (sex)) OR ((gender[MeSH Terms]) OR (gender))) OR ((male[MeSH Terms]) OR (male))) OR ((female[MeSH Terms]) OR (female))) OR ((women[MeSH Terms]) OR (women))) OR ((men[MeSH Terms]) OR (men))) OR ((woman[MeSH Terms]) OR (woman))) OR ((man[MeSH Terms]) OR (man))
European countries	3	(europe[MeSH Terms]) OR (europe)
European countries	4	(((Albania[MeSH Terms]) OR (Andorra[MeSH Terms])) OR (Armenia[MeSH Terms])) OR (Austria[MeSH Terms])) OR (azerbaijan[MeSH Terms])) OR (Belarus[MeSH Terms])) OR (Belgium[MeSH Terms])) OR (Bosnia and Herzegovina[MeSH Terms])) OR (Bulgaria[MeSH Terms])) OR (Croatia[MeSH Terms])) OR (Cyprus[MeSH Terms])) OR (Czechia[MeSH Terms])) OR (Denmark[MeSH Terms])) OR (Estonia[MeSH Terms])) OR (Finland[MeSH Terms])) OR (France[MeSH Terms])) OR (Georgia[MeSH Terms]) OR ((("Germany"[Mesh]) OR "Germany, West"[Mesh]) OR "Germany, East"[Mesh])) OR (Greece[MeSH Terms])) OR (Hungary[MeSH Terms])) OR (Iceland[MeSH Terms])) OR (Ireland[MeSH Terms])) OR (Israel[MeSH Terms])) OR (Italy[MeSH Terms])) OR (Kazakhstan[MeSH Terms])) OR (Kyrgyzstan[MeSH Terms])) OR (Latvia[MeSH Terms])) OR (Lithuania[MeSH Terms])) OR (Luxembourg[MeSH Terms])) OR (Malta[MeSH Terms])) OR (Monaco[MeSH Terms])) OR (Montenegro[MeSH Terms])) OR (Netherlands[MeSH Terms])) OR (North Macedonia[MeSH Terms])) OR (Norway[MeSH Terms])) OR (Poland[MeSH Terms])) OR (Portugal[MeSH Terms])) OR (Republic of Moldova[MeSH Terms])) OR (Romania[MeSH Terms])) OR (Russian Federation[MeSH Terms])) OR (San Marino[MeSH Terms])) OR (Serbia[MeSH Terms])) OR (Slovakia[MeSH Terms])) OR (Slovenia[MeSH Terms])) OR (Spain[MeSH Terms])) OR (Sweden[MeSH Terms])) OR (Switzerland[MeSH Terms])) OR (Tajikistan[MeSH Terms])) OR (Turkey[MeSH Terms])) OR (Turkmenistan[MeSH Terms])) OR (Ukraine[MeSH Terms])) OR (United Kingdom[MeSH Terms])) OR (Uzbekistan[MeSH Terms])) OR (USSR[MeSH Terms])) OR (Czech Republic[MeSH Terms]))) OR (Yugoslavia[MeSH Terms])
European countries	5	(((Albania) OR (Andorra)) OR (Armenia)) OR (Austria)) OR (Azerbaijan)) OR (Belarus)) OR (Belgium)) OR (Bosnia and Herzegovina)) OR (Bulgaria)) OR (Croatia)) OR (Cyprus)) OR (Czechia)) OR (Denmark)) OR (Estonia)) OR (Finland)) OR (France)) OR (Georgia)) OR (Germany)) OR (Greece)) OR (Hungary)) OR (Iceland)) OR (Ireland)) OR (Israel)) OR (Italy)) OR (Kazakhstan)) OR (Kyrgyzstan)) OR (Latvia)) OR

		(Lithuania)) OR (Luxembourg)) OR (Malta)) OR (Monaco)) OR (Montenegro)) OR (Netherlands)) OR (North Macedonia)) OR (Norway)) OR (Poland)) OR (Portugal)) OR (Republic of Moldova)) OR (Romania)) OR (Russian Federation)) OR (San Marino)) OR (Serbia)) OR (Slovakia)) OR (Slovenia)) OR (Spain)) OR (Sweden)) OR (Switzerland)) OR (Tajikistan)) OR (Turkey)) OR (Turkmenistan)) OR (Ukraine)) OR (United Kingdom)) OR (Great Britain)) OR (Northern Ireland)) OR (Uzbekistan)) OR (USSR)) OR (UDSSR)) OR (Czech Republic)) OR (Yugoslavia)
European countries	6	(#3 OR #4 OR #5)
Study	7	((((((study[MeSH Terms]) OR (trial[MeSH Terms])) OR (study[Text Word])) OR (studies[Text Word])) OR (trial[Text Word])) OR (trials[Text Word])) NOT ((case reports[MeSH Terms]) OR (case report[Text Word]))
	8	(#1 AND #2 AND #6 AND #7)

II. EMBASE/GLOBAL HEALTH SEARCH STRATEGY

Concept	#	Embase/Global Health<br>Search terms
TB	1	((tuberculos* or Mycobacterium tuberculosis) NOT (animals not (humans and animals))).kw,ti.
Gender	2	(sex OR gender OR male OR female OR woman OR women OR man OR men)
European countries	3	Europe.kw,ti,ab.
European countries	4	((Albania) OR (Andorra)) OR (Armenia)) OR (Austria)) OR (Azerbaijan)) OR (Belarus)) OR (Belgium)) OR (Bosnia and Herzegovina)) OR (Bulgaria)) OR (Croatia)) OR (Cyprus)) OR (Czechia)) OR (Denmark)) OR (Estonia)) OR (Finland)) OR (France)) OR (Georgia)) OR (Germany)) OR (Greece)) OR (Hungary)) OR (Iceland)) OR (Ireland)) OR (Israel)) OR (Italy)) OR (Kazakhstan)) OR (Kyrgyzstan)) OR (Latvia)) OR (Lithuania)) OR (Luxembourg)) OR (Malta)) OR (Monaco)) OR (Montenegro)) OR (Netherlands)) OR (North Macedonia)) OR (Norway)) OR (Poland)) OR (Portugal)) OR (Republic of Moldova)) OR (Romania)) OR (Russian Federation)) OR (San Marino)) OR (Serbia)) OR (Slovakia)) OR (Slovenia)) OR (Spain)) OR (Sweden)) OR (Switzerland)) OR (Tajikistan)) OR (Turkey)) OR (Turkmenistan)) OR (Ukraine)) OR (United Kingdom)) OR (Great Britain)) OR (Northern Ireland)) OR (Uzbekistan)) OR (USSR)) OR (UDSSR)) OR (Czech Republic)) OR (Yugoslavia).kw,ti,ab.
European countries	5	(#3 OR #4)
Study	6	(((study or studies) OR (trial or trials)) NOT (case report or case reports)). kw
	7	(#1 AND #2 AND #5 AND #6)

III. COCHRANE LIBRARY SEARCH STRATEGY

		Cochrane Library
Concept	#	Search terms
TB	1	(tuberculos* OR "Mycobacterium tuberculosis"):ti
	2	(tuberculos* OR "Mycobacterium tuberculosis"):kw
	3	MeSH descriptor: [Tuberculosis] explode all trees
	4	MeSH descriptor: [Mycobacterium tuberculosis] this term only
Gender	5	(sex OR gender OR male OR female OR woman OR women OR man OR men):all text
	6	MeSH descriptor: [Sex] explode all trees
	7	MeSH descriptor: [Gender Identity] explode all trees
	8	MeSH descriptor: [Male] explode all trees
	9	MeSH descriptor: [Female] explode all trees
European Countries	10	(Europe):ti,ab,kw
	11	MeSH descriptor: [Europe] explode all trees
	12	(((Albania) OR (Andorra)) OR (Armenia)) OR (Austria)) OR (Azerbaijan)) OR (Belarus)) OR (Belgium)) OR (Bosnia and Herzegovina)) OR (Bulgaria)) OR (Croatia)) OR (Cyprus)) OR (Czechia)) OR (Denmark)) OR (Estonia)) OR (Finland)) OR (France)) OR (Georgia)) OR (Germany)) OR (Greece)) OR (Hungary)) OR (Iceland)) OR (Ireland)) OR (Israel)) OR (Italy)) OR (Kazakhstan)) OR (Kyrgyzstan)) OR (Latvia)) OR (Lithuania)) OR (Luxembourg)) OR (Malta)) OR (Monaco)) OR (Montenegro)) OR (Netherlands)) OR (North Macedonia)) OR (Norway)) OR (Poland)) OR (Portugal)) OR (Republic of Moldova)) OR (Romania)) OR (Russian Federation)) OR (San Marino)) OR (Serbia)) OR (Slovakia)) OR (Slovenia)) OR (Spain)) OR (Sweden)) OR (Switzerland)) OR (Tajikistan)) OR (Turkey)) OR (Turkmenistan)) OR (Ukraine)) OR (United Kingdom)) OR (Great Britain)) OR (Northern Ireland)) OR (Uzbekistan)) OR (USSR)) OR (UDSSR)) OR (Czech Republic)) OR (Yugoslavia)
TB	13	(#1 OR #2 OR #3 OR #4)
Gender	14	(#5 OR #6 OR #7 OR #8 OR #9)
European Countries	15	(#10 OR #11 OR #12)
	16	(#13 AND #14 AND #15)
