# Supplementary material for: Tuberculosis case fatality is higher in male than female patients in Europe: a systematic review and meta-analysis
Source: Infection. 2024 Mar 23;52(5):1775–86. doi: 10.1007/s15010-024-02206-z (PMC11499538; doi:10.1007/s15010-024-02206-z)
Supplement: Supplementary file 2 — Online Resource 2 Inclusion and exclusion criteria applied in the systematic review (PDF 183 KB) [file 15010_2024_2206_MOESM2_ESM.pdf]

*Inclusion and exclusion criteria*

| **Inclusion criteria** | **Abbr.** | **Exclusion criteria** | **Abbr.** |
|---|---|---|---|
| Target disease | | Target disease | |
| All TB disease forms | I-0 | No TB disease investigated (e.g., vaccination, LTBI) | E-0 |
| Study types | | Study types | |
| Observational studies, such as prevalence studies and other cross-sectional studies, cohort and case-control studies, as well as intervention studies, e.g. (randomized) controlled therapy studies; diagnostic and prognostic studies | I-1 | Case notification reports, single case reports, patient data reports from health care facilities, small number case series, and studies with less than 30 participants in total or per group studied. | E-1 |
| Participants | | Participants | |
| General population: males and females; both genders represented | I-2 | Specific groups lacking representativeness of the general population, e.g., occupational settings like miners;<br>Children < 15 yrs;<br>Inter-sexual persons;<br>Single-sex represented;<br>Prison inmates; | E-2 |
| Setting | | Setting | |
| European countries* | I-3 | Non-European countries | E-3 |
| Outcomes | | Outcomes | |
| During and post-TB fatality estimates (primary outcome) | I-4 | No during and post-TB fatality estimates (primary outcome) | E-4 |
| | | No TB outcome-related sex data | E-5 |
| | | No TB outcome-related age data | E-6 |
| | | No diagnosis of non-communicable diseases after recovery from TB (secondary outcome) | E-7 |
