# Supplementary material for: Tuberculosis case fatality is higher in male than female patients in Europe: a systematic review and meta-analysis
Source: Infection. 2024 Mar 23;52(5):1775–86. doi: 10.1007/s15010-024-02206-z (PMC11499538; doi:10.1007/s15010-024-02206-z)
Supplement: Supplementary file 3 — Online Resource 3 Countries of the WHO region of Europe (by May 30, 2020) (PDF 184 KB) [file 15010_2024_2206_MOESM3_ESM.pdf]

The WHO region Europe

Table A- 1: Countries of the WHO region Europe (by May 30, 2020) [31]

**Country**
Albania
Andorra
Armenia
Austria
Azerbaijan
Belarus
Belgium
Bosnia and Herzegovina
Bulgaria
Croatia
Cyprus
Czechia
Denmark
Estonia
Finland
France
Georgia
Germany
Greece
Hungary
Iceland
Ireland
Israel
Italy
Kazakhstan
Kyrgyzstan
Latvia
Lithuania
Luxembourg
Malta
Monaco
Montenegro
Netherlands
North Macedonia
Norway
Poland
Portugal
Republic of Moldova
Romania
Russian Federation
San Marino
Serbia
Slovakia
Slovenia
Spain
Sweden
Switzerland
Tajikistan
Turkey
Turkmenistan
Ukraine
United Kingdom of Great Britain and Northern Ireland
Uzbekistan

Table A- 2: European regions (by June 10, 2020)[32]

**European region**	**Countries**
Eastern Europe<br>(Lacking: Kazakhstan, Kyrgyzstan, Tajikistan, Turkmenistan, Uzbekistan)	Belarus BLR<br>Bulgaria BGR<br>Czechia CZE<br>Hungary HUN<br>Poland POL<br>Republic of Moldova MDA<br>Romania ROU<br>Russian Federation RUS<br>Slovakia SVK<br>Ukraine
Northern Europe	Åland Islands ALA<br>Channel Islands<br>Guernsey GGY<br>Jersey JEY<br>Sark<br>Denmark DNK<br>Estonia EST<br>Faroe Islands FRO<br>Finland FIN<br>Iceland ISL<br>Ireland IRL<br>Isle of Man IMN<br>Latvia LVA<br>Lithuania LTU<br>Norway NOR<br>Svalbard and Jan Mayen Islands SJM<br>Sweden SWE<br>United Kingdom of Great Britain and Northern Ireland GBR
Southern Europe	Albania ALB<br>Andorra AND<br>Bosnia and Herzegovina BIH<br>Croatia HRV<br>Gibraltar GIB<br>Greece GRC<br>Holy See VAT<br>Italy ITA<br>Malta MLT<br>Montenegro MNE<br>North Macedonia MKD<br>Portugal PRT<br>San Marino SMR<br>Serbia SRB<br>Slovenia SVN<br>Spain ESP
Western Europe	Austria AUT<br>Belgium BEL<br>France FRA<br>Germany DEU<br>Liechtenstein LIE<br>Luxembourg LUX<br>Monaco MCO<br>Netherlands NLD<br>Switzerland CHE
