# Supplementary material for: Tuberculosis case fatality is higher in male than female patients in Europe: a systematic review and meta-analysis
Source: Infection. 2024 Mar 23;52(5):1775–86. doi: 10.1007/s15010-024-02206-z (PMC11499538; doi:10.1007/s15010-024-02206-z)
Supplement: Supplementary file 4 — Online Resource 4 Data extracted from the publications (PDF 219 KB) [file 15010_2024_2206_MOESM4_ESM.pdf]

Data extracted from the publications

Table A- 3: Variables and their types, categories, function/definition, and coding

Variable	Type	Categories	Function/definition	Coding
No. (Rayyan ID)	Ordinal	As many as publications	Study identifier, unique for each publication included; automatically created by Rayyan software	Nine-digit sequence
Study author	Nominal	As many as publications	Study characteristic; last name of the first author or name of authors' group	Not applicable
Publication year	Discrete	As reported by the publication	Study characteristic; year of publication as stated by the publishing institution	YYYY in digits
Study design	Nominal	Seven	Study characteristic; study design stated by the author(s) or as determined by the doctoral student based on the information derived from the publication	1-7
		Case-control study		1
		Case series		2
		Cohort study		3
		Cross-sectional study		4
		Descriptive study	Studies w/ unclear design features not eligible for classification as case-control, cohort, cross-sectional, interventional study, or case series	5
		Systematic review, meta-analysis		6
		Interventional study		7
Observation period start	Discrete	As reported by the publication	Observation period classifier;	MM/YYYY in digits
Observation period end	Discrete	As reported by the publication	Observation period classifier;	MM/YYYY in digits
Years observed	Discrete	As calculated	Observation period classifier; calculated difference of years between the reported end and the start of a study's observation period	Count numbers
Country	Nominal	As reported by the publication	Country classifier; country/countries reported as study sites	Not applicable
City/ region	Nominal	As reported by the publication	City/region classifier; location(s) at city or region level reported as study sites	Not applicable
Setting	Nominal	As reported by the publication	Setting classifier; first, original setting description including study population provided by publication; later grouped into specific categories as indicated herein.	Not applicable
Study population	Nominal	As reported by the publication	Study population classifier; first, original setting description including study population provided by publication; later grouped into specific categories as indicated herein	Not applicable
N (total)	Discrete	As reported by the publication	Total number of individuals comprised by the study population reported in the publication.	Not applicable
TB (site)	Nominal	As reported by the publication	TB disease type classifier; description of the kind of TB disease provided by the publication	0-24
		Extrapulmonary	TB disease defined or reported as extrapulmonary TB by the publication	0
		Pulmonary	TB disease defined or reported as pulmonary TB by the publication	1

Variable	Type	Categories	Function/definition	Coding
		Miliary	TB disease defined or reported as miliary TB by the publication	2
		Genitourinary	TB disease defined or reported as genitourinary (concerning the genitals or urinary system) TB by the publication	3
		Meningitis	TB disease defined or reported as TB meningitis by the publication	4
		Peritonitis	TB disease defined or reported as TB peritonitis by the publication	5
		Pericarditis	TB disease defined or reported as TB pericarditis by the publication	6
		Lymphadenitis	TB disease defined or reported as TB lymphadenitis by the publication	7
		Cutaneous	TB disease defined or reported as cutaneous TB by the publication	8
		Bones/Joints	TB disease defined or reported as TB of the bones/ joints by the publication	9
		Gastrointestinal	TB disease defined or reported as gastrointestinal TB by the publication	10
		Liver	TB disease defined or reported as TB of the liver by the publication	11
		Other	TB disease defined or reported as TB of other locations as listed herein by the publication	12
		Mixed/ Multiple organs	TB disease defined or reported as mixed TB or TB of multiple organs by the publication	13
		Unknown	TB disease defined or reported as TB of unknown form by the publication	14
		All forms	TB disease defined or reported as TB of all forms by the publication	15
		Extrathoracic Lymph Nodes	TB disease defined or reported as TB of the extrathoracic lymph nodes by the publication	16
		Pleura	TB disease defined or reported as TB of the pleura by the publication	17
		Intrathoracic Lymph Nodes	TB disease defined or reported as TB of the intrathoracic lymph nodes by the publication	18
		Abdomen	TB disease defined or reported as TB lymphadenitis by the publication	19
		Spine	TB disease defined or reported as TB of the spine by the publication	20
		Disseminated	TB disease defined or reported as disseminated TB by the publication	21
		CNS other than meninges	TB disease defined or reported as TB of the CNS other than meninges by the publication	22
		Other extrapulmonary organs	TB disease defined or reported as TB in another extrapulmonary organ as listed herein by the publication	23
		TB sequelae	TB disease defined or reported as TB sequelae by the publication	24
TB cases (total)	Discrete	As reported by the publication	Total number of TB cases reported in the publication	Count numbers
Age (Median/ Average)	Nominal	As reported by the publication	Age classifier; the age of TB cases (total) in years (acc. Information	0-1

Variable	Type	Categories	Function/definition	Coding
			provided by the publication)	
		Mean	If aggregated age of TB cases (total) was reported as mean	0
		Median	If aggregated age of TB cases (total) was reported as median	1
Age TB cases (total)	Continuous	As reported by the publication	Age of TB cases (total) in years as reported by the publication	Real numbers
Range (IQR, SD)	Nominal	As reported by the publication	Age range classifier; selection based on the reported point estimate of Age TB cases (total)	0-3
		SD	Standard deviation calculated in days based on the reported time measurement in the publication	0
		Range	Time range calculated in days based on the reported time measurement in the publication	1
		IQR	Inter-quartile (25-75%) range calculated in days based on the reported time measurement in the publication	2
		95% CI	95% confidence interval calculated in days based on the reported time measurement in the publication	3
Lower limit Age TB cases (total)	Continuous	Years	Lower 95% CI bound of the age of TB cases (total, male, female) calculated in years based on the reported measurement in the publication.	Real numbers
Upper limit Age TB cases (total)	Continuous	Years	Upper 95% CI bound of the age of TB cases (total, male, female) calculated in years based on the reported measurement in the publication	Real numbers
TB cases (foreign origin)	Discrete	As reported by the publication	Number of TB cases per sex (total, male, or female) w/ foreign origin as reported by the publication	Count numbers
TB cases (rural)	Discrete	As reported by the publication	Number of TB cases per sex (total, male, or female) w/ rural residency as reported by the publication	Count numbers
TB cases (urban)	Discrete	As reported by the publication	Number of TB cases per sex (total, male, or female) w/ urban residency as reported by the publication	Count numbers
TB cases (new)	Discrete	As reported by the publication	Number of TB cases per sex (total, male, or female) defined or reported as an incident (new/ first-time) case by publication	Count numbers
Incidence rate (/100.000 population)	Continuous	As reported by the publication	Incidence rate of TB cases per sex (total, male, or female) as reported by the publication, calculated per 100,000 population.	Real numbers
Lower limit CI Incidence rate	Continuous	As reported by the publication	Lower 95% CI bound of the incidence rate of TB cases per sex (total, male, or female) calculated per 100,000 based on the reported measurement in the publication.	Real numbers
Upper limit CI Incidence rate	Continuous	As reported by the publication	Upper 95% CI bound of the incidence rate of TB cases per sex (total, male, or female) calculated per 100,000 based on the reported measurement in the publication	Real numbers
Prevalence (/100.000 population)	Continuous	As reported by the publication	Prevalence rate of TB cases per sex (total, male, or female) as reported by the publication, calculated per 100,000 population.	Real numbers
Lower limit CI Prevalence	Continuous	As reported by the publication	Lower 95% CI bound of the prevalence rate of TB cases per sex (total, male, or female) calculated per 100,000 based on the reported measurement in the publication.	Real numbers
Upper limit CI	Continuous	As reported by the	Upper 95% CI bound of the prevalence	Real numbers

Variable	Type	Categories	Function/definition	Coding
Prevalence		publication	rate of TB cases per sex (total, male, or female) calculated per 100,000 based on the reported measurement in the publication	
Sex	Nominal	As reported by the publication	Sex reported as male, female, or total (non-stratified)	0-2
		Female	Sex reported as female	0
		Male	Sex reported as male	1
		Total	Sex reported as total (non-stratified)	2
Measure	Nominal	Six	Outcome measurement identifier; identification of outcomes as reported in the publication	0-6
		AN	Male and female fatal TB cases reported in absolute numbers	0
		MR (per 100.000)	Male and female fatal TB cases reported as mortality rates per 100,000 population or calculated per 100,000 population by the doctoral student	1
		IR (per 100.000)	Male and female incident and fatal TB cases reported as mortality rates per 100,000 population or calculated per 100,000 population by the doctoral student	2
		SDR (per 100.000)	Male and female fatal TB cases reported as standardized death (mortality) rates per 100,000 population or calculated per 100,000 population by the doctoral student	3
		OR	Male and female fatal TB cases reported as odds ratio	4
		HR (unadj.)	Male and female fatal TB cases reported as unadjusted hazard ratios	5
		RR	Male and female fatal TB cases, reported as risk ratio, also called relative risk	6
TB cases (sex)	Discrete	As reported by the publication	Number of TB cases per sex (total, male, or female) as defined or reported by the publication	Count numbers
Lower CI TB cases (sex)	Discrete	As reported by the publication	Lower 95% CI bound of the number of TB cases per sex (total, male, or female) as defined or reported by publication.	Count numbers
Upper CI TB cases (sex)	Discrete	As reported by the publication	Upper 95% CI bound of the number of TB cases per sex (total, male, or female) as defined or reported by the publication	Count numbers
Age sex (Median/ Average)	Nominal	As reported by the publication	Age classifier; age at the time of death w/ or after TB disease calculated in years (acc. Information provided by the publication)	0-1
		Mean	If aggregated age at death was reported as mean	0
		Median	If aggregated age at death was reported as median	1
Age TB cases (sex)	Continuous	Years	Age of a person in years w/ or after TB disease (total, male, or female)	Real numbers
Range age TB cases (IQR, SD)	Nominal	As reported by the publication	Age range classifier; selection based on the reported point estimate of Age sex (median/average)	0-3
		SD	Standard deviation calculated in days based on the reported time measurement in the publication	0
		Range	Time range calculated in days based on the reported time measurement in the publication	1
		IQR	Inter-quartile (25-75%) range	2

Variable	Type	Categories	Function/definition	Coding
			calculated in days based on the reported time measurement in the publication	
		95% CI	95% confidence interval calculated in days based on the reported time measurement in the publication	3
Lower CI Age TB cases (sex)	Continuous	Years	Lower 95% CI bound of the age of TB cases (total, male, female) calculated in years based on the reported measurement in the publication.	Real numbers
Upper CI Age TB cases (sex)	Continuous	Years	Upper 95% CI bound of the age of TB cases (total, male, female) calculated in years based on the reported measurement in the publication	Real numbers
TB death cases (total)	Discrete	As reported by the publication	Total number of TB fatalities reported in the publication	Count numbers
Sex TB death cases	Nominal	As reported by the publication	Sex of TB fatalities reported as male, female, or total (non-stratified)	0-2
		Female	Sex of TB fatalities reported as female	0
		Male	Sex of TB fatalities reported as male	1
		Total	Sex of TB fatalities reported as total (non-stratified)	2
TB death cases (sex)	Discrete	As reported by the publication	Number of TB fatalities per sex (total, male, or female) as defined or reported by the publication	Count numbers
Lower CI TB death cases (sex)	Discrete	As reported by the publication	Lower 95% CI bound of the number of TB fatalities per sex (total, male, or female) as defined or reported by the publication.	Count numbers
Upper CI TB death cases (sex)	Discrete	As reported by the publication	Upper 95% CI bound of the number of TB fatalities per sex (total, male, or female) as defined or reported by the publication	Count numbers
TB death cases (foreign)	Discrete	As reported by the publication	Number of TB fatalities of foreign origin per sex (total, male, or female) as defined or reported by the publication	Count numbers
Age category	Nominal	As reported by the publication	Age categorizer; information on whether the publication reported age at the time of death or at the time of diagnosis	0-1
		Age at death	If age at the time of death was reported	0
		Age at TB Dx	If age at the time of TB diagnosis was reported	1
Age deaths (Median/Average)	Nominal	As reported by the publication	Age classifier; age at the time of death w/ or after TB disease calculated in years (acc. Information provided by the publication)	0-1
		Mean	If aggregated age at death was reported as mean	0
		Median	If aggregated age at death was reported as median	1
Age TB death cases (sex)	Continuous	Years	Age of a person in years died w/ or after TB disease (total, male, or female)	Real numbers
Range age TB deaths (IQR, SD)	Nominal	As reported by the publication	Age range classifier; selection based on the reported point estimate of age (in years) of TB fatalities (median/average)	0-3
		SD	Standard deviation calculated in years based on the reported time measurement in the publication	0
		Range	Time range calculated in years based on the reported time measurement in the publication	1
		IQR	Inter-quartile (25-75%) range calculated in years based on the reported time measurement in the publication	2
		95% CI	95% confidence interval calculated in	3

Variable	Type	Categories	Function/definition	Coding
			years based on the reported time measurement in the publication	
Lower CI Age TB death cases (sex)	Continuous	Years	Lower 95% CI bound of the age of TB fatalities calculated in years based on the reported measurement in the publication.	Real numbers
Upper CI Age TB death cases (sex)	Continuous	Years	Upper 95% CI bound of the age of TB fatalities calculated in years based on the reported measurement in the publication	Real numbers
Time of death	Nominal	As reported by the publication	Time of death classifier; time of death w/ or after TB disease calculated by specific starting points (acc. Information provided by the publication)	0-13
		Before Dx	Death occurred before TB disease was diagnosed; TB diagnosis during death certification or autopsy	0
		w/o therapy	Death occurred after TB disease was diagnosed but w/o treatment initiation	1
		during therapy	Death occurred during TB treatment (independently of the treatment scheme)	2
		after therapy (<1y)	Death occurred after TB treatment completion (independently of the treatment scheme) but within the first year after completion	3
		1y after therapy	Death occurred after TB treatment completion (independently of the treatment scheme) but within the second year after completion	4
		2ys after therapy	Death occurred after TB treatment completion (independently of the treatment scheme) but within the third year after completion	5
		3ys after therapy	Death occurred after TB treatment completion (independently of the treatment scheme) but within the fourth year after completion	6
		4ys after therapy	Death occurred after TB treatment completion (independently of the treatment scheme) but within the fifth year after completion	7
		5ys after therapy	Death occurred after TB treatment completion (independently of the treatment scheme) but within the sixth year after completion	8
		6-10ys after therapy	Death occurred after TB treatment completion (independently of treatment scheme), but within the seventh to eleventh year after completion	9
		>10ys after therapy	Death occurred after TB treatment completion (independently of the treatment scheme) but after the eleventh year after completion	10
		Within 12 months after Dx	Death occurred within the first 12 months after TB disease was diagnosed but w/o providing information concerning treatment initiation	11
		Last known outcome	Category comprising persons with various outcomes documented as the last known outcome	12
Survival (Median/ Average)	Nominal	As reported by the publication	Survival time classifier; survival time w/ or after TB disease calculated by time of death groups (acc. information provided by the publication)	0-1
		Mean	If aggregated survival time was reported as mean	0

Variable	Type	Categories	Function/definition	Coding
		Median	If aggregated survival time was reported as median	1
Survival time (days)	Discrete	Days	Survival time calculated in days based on the period reported by the publication.	Not applicable
Range (IQR, SD)	Nominal	As reported by the publication	Time range classifier; selection based on the reported point estimate of survival time by the time of death groups (median/average)	0-3
		SD	Standard deviation calculated in days based on the reported time measurement in the publication	0
		Range	Time range calculated in days based on the reported time measurement in the publication	1
		IQR	Inter-quartile (25-75%) range calculated in days based on the reported time measurement in the publication	2
		95% CI	95% confidence interval calculated in days based on the reported time measurement in the publication	3
Lower limit survival time (days)	Discrete	Days	Lower 95% CI bound of TB duration calculated in days based on the reported measurement in the publication.	Count numbers
Upper limit survival time (days)	Discrete	Days	Upper 95% CI bound of TB duration calculated in days based on the reported measurement in the publication	Count numbers
Survival (Median/ Average)	Nominal		Duplicate if both mean and median survivals were reported.	
Survival time (days)	Discrete		Duplicate if both mean and median survivals were reported.	
Range (IQR, SD)	Nominal		Duplicate if both mean and median survivals were reported.	
Lower limit survival time (days)	Discrete		Duplicate if both mean and median survivals were reported.	
Upper limit survival time (days)	Discrete		Duplicate if both mean and median survivals were reported.	
TB site x	Nominal	As reported by the publication	TB type classifier; documented TB type for up to 7 sites per publication (x = 1-7)	0-24
		Same categories as for variable TB (site)		Same coding as for variable TB (site)
TB cases (site x)	Discrete	As reported by the publication	Number of TB cases per sex (total, male, or female) within the defined TB site x as reported by the publication	Count numbers
TB cases (site x; foreign origin)	Discrete	As reported by the publication	Number of TB cases of foreign origin per sex (total, male, or female) within the defined TB site x as reported by the publication	Count numbers
TB-type death cases x	Nominal	Same categories as for variable TB (site)		Same coding as for variable TB (site)
Death category x	Nominal		Death category classifier; death case category defined or reported by the publication	0-1
		In-hospital mortality	Death case category defined or reported as in-hospital mortality by the publication	0
		Overall mortality	Death case category defined or reported as overall mortality by the publication	1
Case category x	Nominal	As reported by the publication	TB case classifier; case category defined or reported by the publication	0-5
		New cases	Persons defined or reported as new (incident/ first-time) TB cases by	0

Variable	Type	Categories	Function/definition	Coding
			publication	
		Relapses	Persons defined or reported as relapsed TB cases by publication	1
		Failures	Persons defined or reported as failure TB cases	2
		All cases	All persons defined or reported as TB cases w/o any differentiation	3
		Chronic cases	Persons defined or reported as chronic TB cases by publication	4
		Previously treated	Persons defined or reported as previously treated TB cases by publication	5
Death cause x	Nominal	As reported by the publication	Death cause classifier; death cause defined or reported by the publication	0-6
		Any cause	Death cause defined or reported as any cause by the publication	0
		TB disease	Death cause defined or reported as TB disease by the publication	1
		TB-induced ARDS/CV failure	Death cause defined or reported as TB-induced ARDS/CV failure by the publication	2
		Pulmonary coinfection	Death cause defined or reported as pulmonary coinfection by the publication	3
		Hospital-acquired pneumonia	Death cause defined or reported as hospital-acquired pneumonia by the publication	4
		Pulmonary embolism	Death cause defined or reported as pulmonary embolism by the publication	5
		Non-TB related	Death cause defined or reported as non-TB related cause by the publication	6
TB death cases (sex) by cause x	Discrete	As reported by the publication	Number of TB fatalities by sex (total, male, or female) and cause within the defined TB site x as reported by the publication	Count numbers
Age group x	Ordinal	Age groups, as reported in the publication	Age group classifier; documented age group for up to 9 groups per publication (x = 1-9)	AG1-9
TB cases (AG x)	Discrete	As reported by the publication	Number of TB cases per sex (total, male, or female) within the defined age group as reported by the publication	Count numbers
Deaths (AG x)	Discrete	As reported by the publication	Number of TB fatalities per sex (total, male, or female) within the defined age group as reported by the publication	Count numbers
Drug resistance (total)	Discrete	Number of individuals, as reported in the publication	Documentation of the total number of individuals with documented drug resistance	Not applicable
Drug resistance x (type)	Nominal	As reported by the publication	Drug resistance classifier; documented drug resistance for up to 8 drugs per publication (x = 1-8)	0-13
		Sensible	TB bacteria fully susceptible to any anti-TB drug applied	0
		MDR	Multidrug-resistant TB (MDR TB); TB bacteria resistant to at least isoniazid and rifampin	1
		XDR	Extensively drug-resistant TB (XDR TB); TB bacteria resistant to isoniazid and rifampin, a fluoroquinolone, and a second-line injectable (amikacin, capreomycin, and kanamycin) OR to isoniazid, rifampin, a fluoroquinolone, and bedaquiline or linezolid	2
		INH	Mono-resistant TB; TB bacteria resistant to isoniazid only	3

Variable	Type	Categories	Function/definition	Coding
		RMP	Mono-resistant TB; TB bacteria resistant to rifampicin only	4
		PZA/EMB	Mono-resistant TB; TB bacteria resistant to pyrazinamide only; PZA resistance may come along together with ethambutol resistance	5
		MonoDR	Mono-resistant TB; TB bacteria resistant to one first-line anti-TB drug only	6
		EMB	Mono-resistant TB; TB bacteria resistant to Ethambutol only	7
		SM	Mono-resistant TB; TB bacteria resistant to streptomycin only	8
		KM	Mono-resistant TB; TB bacteria resistant to kanamycin only	9
		Poly	Poly-resistant TB; TB bacteria resistant to more than one first-line anti-TB drug other than both isoniazid and rifampicin	10
		Any	Any resistant TB; any TB bacteria resistance other than drug sensibility	11
		Pre-XDR	Pre-Extensively Drug-resistant TB (pre-XDR TB); TB bacteria resistant to isoniazid, rifampin, and a fluoroquinolone OR to isoniazid, rifampin, and a second-line injectable (amikacin, capreomycin, and kanamycin)	12
		Inj. Second-line drugs	TB bacteria resistant to a or more second-line injectables (amikacin, capreomycin, and kanamycin)	13
TB cases (DR x)	Discrete	As reported by the publication	Number of TB cases per sex (total, male, or female) with reported drug resistance x	Count numbers
TB cases (foreign; DR x)	Discrete	As reported by the publication	Number of TB cases of foreign origin per sex (total, male, or female) with reported drug resistance x	Count numbers
TB death cases (sex) by DR x	Discrete	As reported by the publication	Number of TB fatalities per sex (total, male, or female) with reported drug resistance x	Count numbers
TB treatment x	Nominal	As reported by the publication	TB treatment classifier; documented TB drug therapy for up to 2 drug treatment schemes per publication (x = 1-2)	0-3
		Standardized	Standardized WHO TB treatment comprising INH, RMP, PZA, EMB, and/or SM	0
		Other	Other TB treatments than the standardized WHO scheme; include different combinations of anti-tuberculosis drugs, including INH, RMP, PZA, EMB, SM, and KM.	1
		No treatment	No TB treatment provided	2
		Standardized + Corticoids	Standardized WHO TB treatment comprising INH, RMP, PZA, EMB, and/or SM plus corticoid application	3
TB cases (sex) by treatment x	Discrete	As reported by the publication	Number of TB cases per sex (total, male, or female) with reported treatment x (x = 1-2; up to 2 schemes reported by publication)	Count numbers
TB death cases (sex) by treatment x	Discrete	As reported by the publication	Number of TB fatalities per sex (total, male, or female) with reported treatment x (x = 1-2; up to 2 schemes reported by publication)	Count numbers
TB duration (category)	Nominal	Survival calculated by specific starting points	Survival time classifier; survival time w/ or after TB disease calculated by	0-2

Variable	Type	Categories	Function/definition	Coding
			specific starting points (acc. Information provided by the publication)	
		Survival after hospital admission	Survival time w/ or after TB disease calculated by the time of hospital admission (for inpatient populations)	0
		Survival after TB treatment initiation	Survival time w/ or after TB disease calculated by the time after starting TB treatment (for in- or outpatient populations)	1
		Survival after TB Dx	Survival time w/ or after TB disease calculated by the time after TB diagnosis (for in- or outpatient populations)	2
TB duration (Median/Average)	Nominal		Survival time classifier; survival time w/ or after TB disease calculated by specific starting points (acc. Information provided by the publication)	0-1
		Mean	If aggregated survival time was reported as mean	0
		Median	If aggregated survival time was reported as median	1
TB duration (time)	Discrete	Days	Survival time calculated in days based on the period reported by the publication.	Not applicable
Range TB duration (IQR, SD)	Nominal	As reported by the publication	Time range classifier; selection based on the reported point estimate of TB duration (median/average)	0-3
		SD	Standard deviation calculated in days based on the reported time measurement in the publication	0
		Range	Time range calculated in days based on the reported time measurement in the publication	1
		IQR	Inter-quartile (25-75%) range calculated in days based on the reported time measurement in the publication	2
		95% CI	95% confidence interval calculated in days based on the reported time measurement in the publication	3
Lower CI TB duration	Discrete	Days	Lower 95% CI bound of TB duration calculated in days based on the reported measurement in the publication.	Count numbers
Upper CI TB duration	Discrete	Days	Upper 95% CI bound of TB duration calculated in days based on the reported measurement in the publication	Count numbers
Education (category x)	Nominal	Two	Education categorizer; publications included used years or levels to describe the educational status	0-1
		Years	Educational status as defined or classified by years of education	0
		Level	Educational status as defined or classified by level of education	1
Education C x (grade)	Nominal	As reported by the publication	Educational status (highest achieved) as defined or reported by the publication (acc. respective educational category of years or level)	0-12
		Unknown	Educational status defined or reported as unknown by the publication	0
		0-8 yrs	Educational status defined or reported as 0-8 years by the publication (w/ years as category)	1
		9-18 yrs	Educational status defined or reported as 9-18 years by the publication (w/ years as category)	2
		Illiterate	Educational status defined or reported	3

Variable	Type	Categories	Function/definition	Coding
			as illiterate by the publication (w/ level as category)	
		Primary school	Educational status defined or reported as primary by the publication (w/ level as category)	4
		Secondary school	Educational status defined or reported as secondary by the publication (w/ level as category)	5
		High school	Educational status defined or reported as high school level by the publication (w/ level as category)	6
		University	Educational status defined or reported as university level by the publication (w/ level as category)	7
		High school or lower level	Educational status defined or reported as high school or lower level by the publication (w/ level as category)	8
		University or higher	Educational status defined or reported as a university or a higher level by the publication (w/ level as category)	9
		Less than primary	Educational status defined or reported as less than primary by the publication (w/ level as category)	10
		Primary/secondary	Educational status defined or reported as primary/secondary level by the publication (w/ level as category)	11
		Tertiary	Educational status defined or reported as tertiary level by the publication (w/ level as category)	12
Cases (Edu C x)	Discrete	As reported by the publication	Number of TB cases per sex (total, male, or female) with reported educational level x (x = 1-5; up to 5 grades reported by publication)	Count numbers
Deaths (Edu C x)	Discrete	As reported by the publication	Number of TB fatalities per sex (total, male, or female) with reported educational level x (x = 1-5; up to 5 grades reported by publication)	Count numbers
Comorb x	Nominal	As reported by the publication, but sorted into specific disease groups defined by the doctoral student	Comorbidity classifier; documented comorbidity for up to 8 comorbidities per publication (x = 1-8)	0-16
		COPD	Respiratory disease defined or reported as COPD by the publication	0
		Malignancies	Any disease defined or reported as malignancy or cancer by the publication	1
		Diabetes	Any disease defined or reported as diabetes by the publication	2
		Heart disease	Any cardiovascular disease defined or reported as such by the publication	3
		Hepatitis	Any disease defined or reported as acute hepatitis of any type by the publication	4
		Chronic hepatic disease/ Liver cirrhosis	Any disease defined or reported as chronic hepatic disease or liver cirrhosis of any type by the publication	5
		HIV/AIDS	Any disease defined or reported as HIV or a shift to AIDS by the publication	6
		Renal dysfunction/dialysis	Any disease defined or reported as renal dysfunction or any person receiving dialysis reported by the publication	7
		Organ transplantation	Any person defined or reported to having received organ transplantation by the publication	8

Variable	Type	Categories	Function/definition	Coding
		Any comorbidity except for HIV/AIDS	Any disease except for HIV/AIDS defined or reported as a disease by the publication	9
		AIDS	Any disease defined or reported as fully developed AIDS stage by the publication	10
		Any comorbidity	Any disease except defined or reported as a disease by the publication	11
		Silicosis	Any disease defined or reported as silicosis by the publication	12
		Other comorbidities	Any disease defined or reported as another disease by the publication; that excludes any disease mentioned by the respective publication; generally, those publications reported diseases such as HIV/AIDS, diabetes, etc., separately	13
		Sarcoidosis	Any disease defined or reported as sarcoidosis by the publication	14
		Immunosuppression	Any medical measure or treatment defined or reported as immunosuppression by the publication	15
		Respiratory system	Any disease defined or reported as a disease of the respiratory system except for pulmonary TB or any other TB form involving the respiratory system by the publication	16
Cases (CM x)	Discrete	As reported by the publication	Number of TB cases per sex (total, male, or female) with reported comorbidity x	Count numbers
Deaths (CM x)	Discrete	As reported by the publication	Number of TB fatalities per sex (total, male, or female) with reported comorbidity x	Count numbers
Risk factor x	Nominal	As reported by the publication	Risk factor classifier; documented risk factor for up to 7 factors per publication (x = 1-7)	0-12
		Alcohol	Any behavior related to alcohol consumption or abuse as defined or reported by the publication	0
		Delayed diagnosis	Any disease diagnosis defined or reported as delayed by the publication	1
		Homelessness	Any living condition defined or reported as homelessness by the publication	2
		Unemployment	Any employment condition defined or reported as unemployment by the publication	3
		Cachexia	Any ill condition reported or defined as cachexia by the publication	4
		Smokers	Any behavior related to smoking (including passive exposition) as defined or reported by the publication	5
		Drug addiction	Any behavior related to illicit drug consumption or abuse as defined or reported by the publication	6
		Migrants	Any administrative status of a person defined or reported as migrant/ immigration history by the publication	7
		Former TB disease	Any ill condition reported or defined as former TB disease by the publication	8
		Imprisonment	Any living condition defined or reported as imprisonment (due to criminal activities) by the publication	9
		Undernutrition	Any health condition reported or	10

Variable	Type	Categories	Function/definition	Coding
			defined as undernutrition by the publication	
		Immunosuppressive therapy	Any medical treatment defined or reported as immunosuppressive therapy by the publication	11
		Rural residency	Any living condition defined or reported as a rural residency by the publication	12
Cases (RF x)	Discrete	As reported by the publication	Number of TB cases per sex (total, male, or female) with reported risk factor x	Count numbers
Deaths (RF x)	Discrete	As reported by the publication	Number of TB fatalities per sex (total, male, or female) with reported risk factor x	Count numbers
Comment	Nominal	Not applicable	Field for comments/ notes about issues/ topics related to the specific publication	Not applicable
Language	Nominal	As identified for a specific publication	Language classifier; language in which the study was published	Two-character country code/ abbr. acc. ISO-3166-1 ALPHA-2; https://laendercode.net/de/2-letter-list.html
Data extracted	Nominal	Three	Working status classifier; indicated whether data were extracted or not	0-2
		No		0
		Not abstractable		1
		Yes		2
