# Supplementary material for: Tuberculosis case fatality is higher in male than female patients in Europe: a systematic review and meta-analysis
Source: Infection. 2024 Mar 23;52(5):1775–86. doi: 10.1007/s15010-024-02206-z (PMC11499538; doi:10.1007/s15010-024-02206-z)
Supplement: Supplementary file 6 — Online Resource 6 Data files used for meta-analyses (PDF 281 KB) [file 15010_2024_2206_MOESM6_ESM.pdf]

Data files used for meta-analyses

I. Studies reporting absolute numbers

Primary files

Legend of primary files: study = publication identifier comprising the first author and publication year; event.e = number of male TB fatalities, n.e = number of male TB cases; event.c = number of female TB fatalities, n.c = number of female TB cases

Dataset n = 94 (interim analyis)

study	event.e	n.e	event.c	n.c
Abutidze 2012	34	90	6	20
Aguado 1997	10	30	6	21
Alvarez 2011	6227	6227	2303	2303
Arghir 2018	213	213	34	34
Balabanova 2016	195	581	32	156
Bartu 2010	9	28	0	5
Bastos 2016	96	501	25	180
Baussano 2008	77	964	29	600
Bendayan 2011	32	102	8	30
Bloendal 2013	535	1775	126	674
Borgdorff 1998	170	2603	88	1737
Cayla 2003	20	833	6	458
Conti 2007	254	254	152	152
Crofts 2008	704	7134	404	6019
Daucourt 2000	27	183	13	113
Dewan 2004	55	198	8	57
Diacon 2014	8	85	4	47
Diel 2003	24	361	8	157
Dobrotkova 2019	138	1254	52	605
Duro 2017	18	29	3	10
Erbes 2006	11	46	3	12
Farah 2005	41	384	17	271
Faustini 2008	43	516	12	259
Floe 2017	930	3750	581	2963
Forssbohm 2011	139	139	84	84
Fortun 2014	37	529	11	285
Franco Spinola 2015	297	789	69	309
Frank 2019	12	77	4	34
Gadoev 2015	3855	63724	2098	43656
GBD Tuberculosis Collaborator 2014 (CE)	2201	25157	646	12384
GBD Tuberculosis Collaborator 2014 (EE)	17428	133535	3944	54525
GBD Tuberculosis Collaborator 2014 (WE)	2660	29517	2011	19185
Girardi 2012	32	199	4	47
Girardi 2014	142	3201	66	1957
Grabauskas 2011	1784	1784	346	346
Haar 2007	75	400	16	142
Helbling 2002	19	176	4	89
Holden 2020	106	1311	35	820
Holmberg 2019	15	35	1	18
Jamilloux 2016	6	6	10	10

study	event.e	n.e	event.c	n.c
Khaliaukin 2014	133	367	21	72
Kherosheva 2003	28	574	3	175
Kleina 2017	81	81	23	23
Korhonen 2020	163	915	68	501
Korzeniewska-Kosela 2017	413	4457	113	1973
Korzeniewska-Kosela 2018	420	4457	117	1987
Korzeniewska-Kosela 2019	418	4126	125	1661
Korzeniewska-Kosela 2020	383	3900	107	1587
Kourbatova 2006	71	328	21	132
Lanoix 2014	16	77	5	20
Lockman 2001	10	62	2	30
Lowe 2013	29	408	13	310
Loytved 2002	16	538	6	329
Lubart 2007	45	311	20	149
Majoor 2011	24	105	22	126
Makhmudova 2019	49	342	40	259
Mathew 2006	143	1326	40	590
Mileeva 2002	52	52	10	10
Millet 2010	134	520	39	242
Minelli 2018	171	171	137	137
Nebreda-Mayoral 2017	6	47	1	28
Ordobas 2003	915	915	291	291
Panic 2003	29	264	4	85
Pedrazzoli 2019	4161	62628	2371	49146
Pina 2006	98	1352	35	733
Podlekareva 2014	220	419	66	168
Podlekareva 2016	170	830	66	321
Pogorelova 2010	258	258	45	45
Pradipta 2019a	72	3426	40	2248
Public Health England 2013	49	5045	30	3706
Public Health England 2014a	300	4560	132	3332
Public Health England 2014b	300	3596	132	2842
Public Health England 2015	210	3078	120	2367
Public Health England 2016	229	3407	122	2351
Public Health England 2020a	115	2329	66	1677
Public Health England 2020b	115	2684	66	1930
Public Health Ukraine 2017	22240	112390	4868	46117
RKI 2014	97	2665	48	1637
RKI 2017	65	4000	35	1905
RKI 2019	85	3626	44	1795
RKI 2020	82	3117	47	1665
Rodriguez-Valin 2015	284	3690	96	2190
Safaryan 2002	163	163	35	35
Savic 2016	14	14	21	21
Shuldiner 2014	208	2612	117	1925
Shuldiner 2016	238	1789	147	1412
Talay 2008	14	463	0	123
Theegarten 2006	34	34	21	21
Valade 2012	14	40	6	13
Valek 2003	7	7	4	4

study	event.e	n.e	event.c	n.c
Vasankari 2007	73	386	35	243
Vichi 2010	199	199	151	151
WHO 2020	17200	155000	6480	80000
Zaridze 2009	709	709	103	103

Dataset n = 77 (interim analysis)

study	event.e	n.e	event.c	n.c
Abutidze 2012	34	90	6	20
Aguado 1997	10	30	6	21
Balabanova 2016	195	581	32	156
Bartu 2010	9	28	0	5
Bastos 2016	96	501	25	180
Baussano 2008	77	964	29	600
Bendayan 2011	32	102	8	30
Bloendal 2013	535	1775	126	674
Borgdorff 1998	170	2603	88	1737
Cayla 2003	20	833	6	458
Crofts 2008	704	7134	404	6019
Daucourt 2000	27	183	13	113
Dewan 2004	55	198	8	57
Diacon 2014	8	85	4	47
Diel 2003	24	361	8	157
Dobrotkova 2019	138	1254	52	605
Duro 2017	18	29	3	10
Erbes 2006	11	46	3	12
Farah 2005	41	384	17	271
Faustini 2008	43	516	12	259
Floe 2017	930	3750	581	2963
Fortun 2014	37	529	11	285
Franco Spinola 2015	297	789	69	309
Frank 2019	12	77	4	34
Gadoev 2015	3855	63724	2098	43656
GBD Tuberculosis Collaborator 2014 (CE)	2201	25157	646	12384
GBD Tuberculosis Collaborator 2014 (EE)	17428	133535	3944	54525
GBD Tuberculosis Collaborator 2014 (WE)	2660	29517	2011	19185
Girardi 2012	32	199	4	47
Girardi 2014	142	3201	66	1957
Haar 2007	75	400	16	142
Helbling 2002	19	176	4	89
Holden 2020	106	1311	35	820
Holmberg 2019	15	35	1	18
Khaliaukin 2014	133	367	21	72
Kherosheva 2003	28	574	3	175
Korhonen 2020	163	915	68	501
Korzeniewska-Kosela 2017	413	4457	113	1973
Korzeniewska-Kosela 2018	420	4457	117	1987
Korzeniewska-Kosela 2019	418	4126	125	1661
Korzeniewska-Kosela 2020	383	3900	107	1587
Kourbatova 2006	71	328	21	132
Lanoix 2014	16	77	5	20
Lockman 2001	10	62	2	30
Lowe 2013	29	408	13	310
Loytved 2002	16	538	6	329
Lubart 2007	45	311	20	149
Majoor 2011	24	105	22	126

study	event.e	n.e	event.c	n.c
Makhmudova 2019	49	342	40	259
Mathew 2006	143	1326	40	590
Millet 2010	134	520	39	242
Nebreda-Mayoral 2017	6	47	1	28
Panic 2003	29	264	4	85
Pedrazzoli 2019	4161	62628	2371	49146
Pina 2006	98	1352	35	733
Podlekareva 2014	220	419	66	168
Podlekareva 2016	170	830	66	321
Pradipta 2019a	72	3426	40	2248
Public Health England 2013	49	5045	30	3706
Public Health England 2014a	300	4560	132	3332
Public Health England 2014b	300	3596	132	2842
Public Health England 2015	210	3078	120	2367
Public Health England 2016	229	3407	122	2351
Public Health England 2020a	115	2329	66	1677
Public Health England 2020b	115	2684	66	1930
Public Health Ukraine 2017	22240	112390	4868	46117
RKI 2014	97	2665	48	1637
RKI 2017	65	4000	35	1905
RKI 2019	85	3626	44	1795
RKI 2020	82	3117	47	1665
Rodriguez-Valin 2015	284	3690	96	2190
Shuldiner 2014	208	2612	117	1925
Shuldiner 2016	238	1789	147	1412
Talay 2008	14	463	0	123
Valade 2012	14	40	6	13
Vasankari 2007	73	386	35	243
WHO 2020	17200	155000	6480	80000

Dataset n = 75 (main analysis)

study	event.e	n.e	event.c	n.c
Abutidze 2012	34	90	6	20
Aguado 1997	10	30	6	21
Balabanova 2016	195	581	32	156
Bartu 2010	9	28	0	5
Bastos 2016	96	501	25	180
Baussano 2008	77	964	29	600
Bendayan 2011	32	102	8	30
Bloendal 2013	535	1775	126	674
Borgdorff 1998	170	2603	88	1737
Cayla 2003	20	833	6	458
Crofts 2008	704	7134	404	6019
Daucourt 2000	27	183	13	113
Dewan 2004	55	198	8	57
Diacon 2014	8	85	4	47
Diel 2003	24	361	8	157
Dobrotkova 2019	138	1254	52	605
Duro 2017	18	29	3	10
Erbes 2006	11	46	3	12
Farah 2005	41	384	17	271
Faustini 2008	43	516	12	259
Floe 2017	930	3750	581	2963
Fortun 2014	37	529	11	285
Franco Spinola 2015	297	789	69	309
Frank 2019	12	77	4	34
Gadoev 2015	3855	63724	2098	43656
GBD Tuberculosis Collaborator 2014 (CE)	2201	25157	646	12384
GBD Tuberculosis Collaborator 2014 (EE)	17428	133535	3944	54525
GBD Tuberculosis Collaborator 2014 (WE)	2660	29517	2011	19185
Girardi 2012	32	199	4	47
Girardi 2014	142	3201	66	1957
Haar 2007	75	400	16	142
Helbling 2002	19	176	4	89
Holden 2020	106	1311	35	820
Holmberg 2019	15	35	1	18
Khaliaukin 2014	133	367	21	72
Kherosheva 2003	28	574	3	175
Korhonen 2020	163	915	68	501
Korzeniewska-Kosela 2017	413	4457	113	1973
Korzeniewska-Kosela 2018	420	4457	117	1987
Korzeniewska-Kosela 2019	418	4126	125	1661
Korzeniewska-Kosela 2020	383	3900	107	1587
Kourbatova 2006	71	328	21	132
Lanoix 2014	16	77	5	20
Lockman 2001	10	62	2	30
Lowe 2013	29	408	13	310
Loytved 2002	16	538	6	329
Lubart 2007	45	311	20	149
Majoor 2011	24	105	22	126

study	event.e	n.e	event.c	n.c
Makhmudova 2019	49	342	40	259
Mathew 2006	143	1326	40	590
Millet 2010	134	520	39	242
Nebreda-Mayoral 2017	6	47	1	28
Panic 2003	29	264	4	85
Pedrazzoli 2019	4161	62628	2371	49146
Pina 2006	98	1352	35	733
Podlekareva 2014	220	419	66	168
Podlekareva 2016	170	830	66	321
Pradipta 2019a	72	3426	40	2248
Public Health England 2013	49	5045	30	3706
Public Health England 2014a	300	4560	132	3332
Public Health England 2015	210	3078	120	2367
Public Health England 2016	229	3407	122	2351
Public Health England 2020b	115	2684	66	1930
Public Health Ukraine 2017	22240	112390	4868	46117
RKI 2014	97	2665	48	1637
RKI 2017	65	4000	35	1905
RKI 2019	85	3626	44	1795
RKI 2020	82	3117	47	1665
Rodriguez-Valin 2015	284	3690	96	2190
Shuldiner 2014	208	2612	117	1925
Shuldiner 2016	238	1789	147	1412
Talay 2008	14	463	0	123
Valade 2012	14	40	6	13
Vasankari 2007	73	386	35	243
WHO 2020	17200	155000	6480	80000

Dataset n = 67 (sensitivity analysis)

study	event.e	n.e	event.c	n.c
Abutidze 2012	34	90	6	20
Aguado 1997	10	30	6	21
Balabanova 2016	195	581	32	156
Bastos 2016	96	501	25	180
Baussano 2008	77	964	29	600
Bendayan 2011	32	102	8	30
Bloendal 2013	535	1775	126	674
Borgdorff 1998	170	2603	88	1737
Cayla 2003	20	833	6	458
Crofts 2008	704	7134	404	6019
Daucourt 2000	27	183	13	113
Dewan 2004	55	198	8	57
Diacon 2014	8	85	4	47
Diel 2003	24	361	8	157
Dobrotkova 2019	138	1254	52	605
Erbes 2006	11	46	3	12
Farah 2005	41	384	17	271
Faustini 2008	43	516	12	259
Floe 2017	930	3750	581	2963
Fortun 2014	37	529	11	285
Franco Spinola 2015	297	789	69	309
Frank 2019	12	77	4	34
Gadoev 2015	3855	63724	2098	43656
GBD Tuberculosis Collaborator 2014 (CE)	2201	25157	646	12384
GBD Tuberculosis Collaborator 2014 (EE)	17428	133535	3944	54525
GBD Tuberculosis Collaborator 2014 (WE)	2660	29517	2011	19185
Girardi 2012	32	199	4	47
Girardi 2014	142	3201	66	1957
Haar 2007	75	400	16	142
Holden 2020	106	1311	35	820
Khaliaukin 2014	133	367	21	72
Korhonen 2020	163	915	68	501
Korzeniewska-Kosela 2017	413	4457	113	1973
Korzeniewska-Kosela 2018	420	4457	117	1987
Korzeniewska-Kosela 2019	418	4126	125	1661
Korzeniewska-Kosela 2020	383	3900	107	1587
Kourbatova 2006	71	328	21	132
Lanoix 2014	16	77	5	20
Lowe 2013	29	408	13	310
Loytved 2002	16	538	6	329
Lubart 2007	45	311	20	149
Majoor 2011	24	105	22	126
Makhmudova 2019	49	342	40	259
Mathew 2006	143	1326	40	590
Millet 2010	134	520	39	242
Panic 2003	29	264	4	85
Pedrazzoli 2019	4161	62628	2371	49146
Pina 2006	98	1352	35	733

study	event.e	n.e	event.c	n.c
Podlekareva 2014	220	419	66	168
Podlekareva 2016	170	830	66	321
Pradipta 2019a	72	3426	40	2248
Public Health England 2013	49	5045	30	3706
Public Health England 2014a	300	4560	132	3332
Public Health England 2015	210	3078	120	2367
Public Health England 2016	229	3407	122	2351
Public Health England 2020b	115	2684	66	1930
Public Health Ukraine 2017	22240	112390	4868	46117
RKI 2014	97	2665	48	1637
RKI 2017	65	4000	35	1905
RKI 2019	85	3626	44	1795
RKI 2020	82	3117	47	1665
Rodriguez-Valin 2015	284	3690	96	2190
Shuldiner 2014	208	2612	117	1925
Shuldiner 2016	238	1789	147	1412
Valade 2012	14	40	6	13
Vasankari 2007	73	386	35	243
WHO 2020	17200	155000	6480	80000

Group files

Annotation: Group files exclusively matter to the primary files of absolute numbers, as subgroup analyses only occurred for these datasets. Given the explorative investigations of the 94 and 77 studies comprising datasets, the group files presentation focuses on the datasets n = 75 (main analysis) and n = 67 (sensitivity analysis).

Legend of group files: study = publication identifier comprising the first author and publication year; group: The categorial feature of an individual publication related to the study characteristic of interest as defined in Appendix 10 (Study characteristics and their categories used in the subgroup analyses).

Dataset n = 75 (main analysis)

Group file countries

study	group
Abutidze 2012	Georgia
Aguado 1997	Spain
Balabanova 2016	Eastern Europe
Bartu 2010	Czech Republic
Bastos 2016	Portugal
Baussano 2008	Italy
Bendayan 2011	Israel
Bloendal 2013	Estonia
Borgdorff 1998	Netherlands
Cayla 2003	Spain
Crofts 2008	United Kingdom
Daucourt 2000	France
Dewan 2004	Russia
Diacon 2014	Eastern Europe
Diel 2003	Germany
Dobrotkova 2019	Slovakia
Duro 2017	Portugal
Erbes 2006	Germany
Farah 2005	Norway
Faustini 2008	Italy
Floe 2017	Denmark
Fortun 2014	Spain
Franco Spinola 2015	Portugal
Frank 2019	Georgia
Gadoev 2015	Uzbekistan
GBD Tuberculosis Collaborator 2014 (CE)	Central Europe
GBD Tuberculosis Collaborator 2014 (EE)	Eastern Europe
GBD Tuberculosis Collaborator 2014 (WE)	Western Europe
Girardi 2012	Italy
Girardi 2014	France
Haar 2007	Netherlands
Helbling 2002	Switzerland
Holden 2020	Denmark
Holmberg 2019	Finland
Khaliaukin 2014	Belarus
Kherosheva 2003	Russia
Korhonen 2020	Finland
Korzeniewska-Kosela 2017	Poland
Korzeniewska-Kosela 2018	Poland
Korzeniewska-Kosela 2019	Poland
Korzeniewska-Kosela 2020	Poland
Kourbatova 2006	Russia
Lanoix 2014	France
Lockman 2001	Estonia
Lowe 2013	United Kingdom
Loytved 2002	Germany
Lubart 2007	Israel

study	group
Majoor 2011	Netherlands
Makhmudova 2019	Tajikistan
Mathew 2006	Russia
Millet 2010	Spain
Nebreda-Mayoral 2017	Spain
Panic 2003	Yugoslavia
Pedrazzoli 2019	United Kingdom
Pina 2006	Spain
Podlekareva 2014	Eastern Europe
Podlekareva 2016	Europe
Pradipta 2019a	Netherlands
Public Health England 2013	United Kingdom
Public Health England 2014a	United Kingdom
Public Health England 2015	United Kingdom
Public Health England 2016	United Kingdom
Public Health England 2020b	United Kingdom
Public Health Ukraine 2017	Ukraine
RKI 2014	Germany
RKI 2017	Germany
RKI 2019	Germany
RKI 2020	Germany
Rodriguez-Valin 2015	Spain
Shuldiner 2014	Israel
Shuldiner 2016	Israel
Talay 2008	Turkey
Valade 2012	France
Vasankari 2007	Finland
WHO 2020	Europe

Group file data source

study	group
Abutidze 2012	Hospital data
Aguado 1997	Hospital data
Balabanova 2016	TB dispensary data
Bartu 2010	Hospital data
Bastos 2016	TB dispensary data
Baussano 2008	Notification data
Bendayan 2011	Hospital data
Bloendal 2013	Notification data
Borgdorff 1998	Notification data
Cayla 2003	Hospital data
Crofts 2008	Notification data
Daucourt 2000	Notification data
Dewan 2004	TB dispensary data
Diacon 2014	Hospital data
Diel 2003	Notification data
Dobrotkova 2019	Notification data
Duro 2017	Hospital data
Erbes 2006	Hospital data
Farah 2005	Notification data
Faustini 2008	Notification data
Floe 2017	Notification data
Fortun 2014	Hospital data
Franco Spinola 2015	Notification data
Frank 2019	Notification data
Gadoev 2015	Notification data
GBD Tuberculosis Collaborator 2014 (CE)	Notification data
GBD Tuberculosis Collaborator 2014 (EE)	Notification data
GBD Tuberculosis Collaborator 2014 (WE)	Notification data
Girardi 2012	Hospital data
Girardi 2014	Notification data
Haar 2007	Notification data
Helbling 2002	Notification data
Holden 2020	Notification data
Holmberg 2019	Hospital data
Khaliaukin 2014	Hospital data
Kherosheva 2003	Notification data
Korhonen 2020	Notification data
Korzeniewska-Kosela 2017	Notification data
Korzeniewska-Kosela 2018	Notification data
Korzeniewska-Kosela 2019	Notification data
Korzeniewska-Kosela 2020	Notification data
Kourbatova 2006	TB dispensary data
Lanoix 2014	Notification data
Lockman 2001	Notification data
Lowe 2013	Hospital data
Loytved 2002	Notification data
Lubart 2007	Hospital data
Majoor 2011	Notification data
Makhmudova 2019	Hospital data

study	group
Mathew 2006	Notification data
Millet 2010	Notification data
Nebreda-Mayoral 2017	Hospital data
Panic 2003	Hospital data
Pedrazzoli 2019	Notification data
Pina 2006	Notification data
Podlekareva 2014	Hospital data
Podlekareva 2016	Hospital data
Pradipta 2019a	Notification data
Public Health England 2013	Notification data
Public Health England 2014a	Notification data
Public Health England 2015	Notification data
Public Health England 2016	Notification data
Public Health England 2020b	Notification data
Public Health Ukraine 2017	Notification data
RKI 2014	Notification data
RKI 2017	Notification data
RKI 2019	Notification data
RKI 2020	Notification data
Rodriguez-Valin 2015	Notification data
Shuldiner 2014	TB dispensary data
Shuldiner 2016	Notification data
Talay 2008	TB dispensary data
Valade 2012	Hospital data
Vasankari 2007	Hospital data
WHO 2020	Notification data

Group file group size

study	group
Abutidze 2012	Group size less than 30
Aguado 1997	Group size less than 30
Balabanova 2016	Group size equal or more than 30
Bartu 2010	Group size less than 30
Bastos 2016	Group size equal or more than 30
Baussano 2008	Group size equal or more than 30
Bendayan 2011	Group size equal or more than 30
Bloendal 2013	Group size equal or more than 30
Borgdorff 1998	Group size equal or more than 30
Cayla 2003	Group size equal or more than 30
Crofts 2008	Group size equal or more than 30
Daucourt 2000	Group size equal or more than 30
Dewan 2004	Group size equal or more than 30
Diacon 2014	Group size equal or more than 30
Diel 2003	Group size equal or more than 30
Dobrotkova 2019	Group size equal or more than 30
Duro 2017	Group size less than 30
Erbes 2006	Group size less than 30
Farah 2005	Group size equal or more than 30
Faustini 2008	Group size equal or more than 30
Floe 2017	Group size equal or more than 30
Fortun 2014	Group size equal or more than 30
Franco Spinola 2015	Group size equal or more than 30
Frank 2019	Group size equal or more than 30
Gadoev 2015	Group size equal or more than 30
GBD Tuberculosis Collaborator 2014 (CE)	Group size equal or more than 30
GBD Tuberculosis Collaborator 2014 (EE)	Group size equal or more than 30
GBD Tuberculosis Collaborator 2014 (WE)	Group size equal or more than 30
Girardi 2012	Group size equal or more than 30
Girardi 2014	Group size equal or more than 30
Haar 2007	Group size equal or more than 30
Helbling 2002	Group size equal or more than 30
Holden 2020	Group size equal or more than 30
Holmberg 2019	Group size less than 30
Khaliaukin 2014	Group size equal or more than 30
Kherosheva 2003	Group size equal or more than 30
Korhonen 2020	Group size equal or more than 30
Korzeniewska-Kosela 2017	Group size equal or more than 30
Korzeniewska-Kosela 2018	Group size equal or more than 30
Korzeniewska-Kosela 2019	Group size equal or more than 30
Korzeniewska-Kosela 2020	Group size equal or more than 30
Kourbatova 2006	Group size equal or more than 30
Lanoix 2014	Group size less than 30
Lockman 2001	Group size equal or more than 30
Lowe 2013	Group size equal or more than 30
Loytved 2002	Group size equal or more than 30
Lubart 2007	Group size equal or more than 30
Majoor 2011	Group size equal or more than 30
Makhmudova 2019	Group size equal or more than 30

study	group
Mathew 2006	Group size equal or more than 30
Millet 2010	Group size equal or more than 30
Nebreda-Mayoral 2017	Group size less than 30
Panic 2003	Group size equal or more than 30
Pedrazzoli 2019	Group size equal or more than 30
Pina 2006	Group size equal or more than 30
Podlekareva 2014	Group size equal or more than 30
Podlekareva 2016	Group size equal or more than 30
Pradipta 2019a	Group size equal or more than 30
Public Health England 2013	Group size equal or more than 30
Public Health England 2014a	Group size equal or more than 30
Public Health England 2015	Group size equal or more than 30
Public Health England 2016	Group size equal or more than 30
Public Health England 2020b	Group size equal or more than 30
Public Health Ukraine 2017	Group size equal or more than 30
RKI 2014	Group size equal or more than 30
RKI 2017	Group size equal or more than 30
RKI 2019	Group size equal or more than 30
RKI 2020	Group size equal or more than 30
Rodriguez-Valin 2015	Group size equal or more than 30
Shuldiner 2014	Group size equal or more than 30
Shuldiner 2016	Group size equal or more than 30
Talay 2008	Group size equal or more than 30
Valade 2012	Group size less than 30
Vasankari 2007	Group size equal or more than 30
WHO 2020	Group size equal or more than 30

Group file observation period

study	group
Abutidze 2012	Observation period: 2000s
Aguado 1997	Observation period: 1980s-1990s
Balabanova 2016	Observation period: 2000s-2010s
Bartu 2010	Observation period: 2000s
Bastos 2016	Observation period: 2000s-2010s
Baussano 2008	Observation period: 2000s
Bendayan 2011	Observation period: 2000s
Bloendal 2013	Observation period: 2000s-2010s
Borgdorff 1998	Observation period: 1990s
Cayla 2003	Observation period: 1990s-2000s
Crofts 2008	Observation period: 2000s
Daucourt 2000	Observation period: 1990s
Dewan 2004	Observation period: 1990s-2000s
Diacon 2014	Observation period: 2000s-2010s
Diel 2003	Observation period: 1990s-2000s
Dobrotkova 2019	Observation period: 2000s-2010s
Duro 2017	Observation period: 2000s-2010s
Erbes 2006	Observation period: 1990s-2000s
Farah 2005	Observation period: 1990s-2000s
Faustini 2008	Observation period: 1990s-2000s
Floe 2017	Observation period: 1990s-2010s
Fortun 2014	Observation period: 1990s-2000s
Franco Spinola 2015	Observation period: 2000s-2010s
Frank 2019	Observation period: 2010s
Gadoev 2015	Observation period: 2000s-2010s
GBD Tuberculosis Collaborator 2014 (CE)	Observation period: 1990s-2010s
GBD Tuberculosis Collaborator 2014 (EE)	Observation period: 1990s-2010s
GBD Tuberculosis Collaborator 2014 (WE)	Observation period: 1990s-2010s
Girardi 2012	Observation period: 1990s-2000s
Girardi 2014	Observation period: 2010s
Haar 2007	Observation period: 1990s-2000s
Helbling 2002	Observation period: 1990s
Holden 2020	Observation period: 2000s-2010s
Holmberg 2019	Observation period: 1990s-2010s
Khaliaukin 2014	Observation period: 2000s-2010s
Kherosheva 2003	Observation period: 1990s-2000s
Korhonen 2020	Observation period: 2000s-2010s
Korzeniewska-Kosela 2017	Observation period: 2010s
Korzeniewska-Kosela 2018	Observation period: 2010s
Korzeniewska-Kosela 2019	Observation period: 2010s
Korzeniewska-Kosela 2020	Observation period: 2010s
Kourbatova 2006	Observation period: 1990s-2000s
Lanoix 2014	Observation period: 2000s
Lockman 2001	Observation period: 1990s
Lowe 2013	Observation period: 1990s-2000s
Loytved 2002	Observation period: 1990s-2000s
Lubart 2007	Observation period: 2000s
Majoor 2011	Observation period: 1990s-2000s
Makhmudova 2019	Observation period: 2010s

study	group
Mathew 2006	Observation period: 2000s
Millet 2010	Observation period: 1990s-2000s
Nebreda-Mayoral 2017	Observation period: 2000s-2010s
Panic 2003	Observation period: 1990s
Pedrazzoli 2019	Observation period: 2000s-2010s
Pina 2006	Observation period: 1990s
Podlekareva 2014	Observation period: 2000s-2010s
Podlekareva 2016	Observation period: 2010s
Pradipta 2019a	Observation period: 2000s-2010s
Public Health England 2013	Observation period: 2010s
Public Health England 2014a	Observation period: 2010s
Public Health England 2015	Observation period: 2010s
Public Health England 2016	Observation period: 2010s
Public Health England 2020b	Observation period: 2010s
Public Health Ukraine 2017	Observation period: 2010s
RKI 2014	Observation period: 2010s
RKI 2017	Observation period: 2010s
RKI 2019	Observation period: 2010s
RKI 2020	Observation period: 2010s
Rodriguez-Valin 2015	Observation period: 2010s
Shuldiner 2014	Observation period: 2000s-2010s
Shuldiner 2016	Observation period: 2000s-2010s
Talay 2008	Observation period: 1990s-2000s
Valade 2012	Observation period: 2000s
Vasankari 2007	Observation period: 1990s
WHO 2020	Observation period: 2010s

Group file publication date

study	group
Abutidze 2012	Published 2010-19
Aguado 1997	Published 1990-99
Balabanova 2016	Published 2010-19
Bartu 2010	Published 2010-19
Bastos 2016	Published 2010-19
Baussano 2008	Published 2000-09
Bendayan 2011	Published 2010-19
Bloendal 2013	Published 2010-19
Borgdorff 1998	Published 1990-99
Cayla 2003	Published 2000-09
Crofts 2008	Published 2000-09
Daucourt 2000	Published 2000-09
Dewan 2004	Published 2000-09
Diacon 2014	Published 2010-19
Diel 2003	Published 2000-09
Dobrotkova 2019	Published 2010-19
Duro 2017	Published 2010-19
Erbes 2006	Published 2000-09
Farah 2005	Published 2000-09
Faustini 2008	Published 2000-09
Floe 2017	Published 2010-19
Fortun 2014	Published 2010-19
Franco Spinola 2015	Published 2010-19
Frank 2019	Published 2010-19
Gadoev 2015	Published 2010-19
GBD Tuberculosis Collaborator 2014 (CE)	Published 2010-19
GBD Tuberculosis Collaborator 2014 (EE)	Published 2010-19
GBD Tuberculosis Collaborator 2014 (WE)	Published 2010-19
Girardi 2012	Published 2010-19
Girardi 2014	Published 2010-19
Haar 2007	Published 2000-09
Helbling 2002	Published 2000-09
Holden 2020	Published 2020-
Holmberg 2019	Published 2010-19
Khaliaukin 2014	Published 2010-19
Kherosheva 2003	Published 2000-09
Korhonen 2020	Published 2020-
Korzeniewska-Kosela 2017	Published 2010-19
Korzeniewska-Kosela 2018	Published 2010-19
Korzeniewska-Kosela 2019	Published 2010-19
Korzeniewska-Kosela 2020	Published 2020-
Kourbatova 2006	Published 2000-09
Lanoix 2014	Published 2010-19
Lockman 2001	Published 2000-09
Lowe 2013	Published 2010-19
Loytved 2002	Published 2000-09
Lubart 2007	Published 2000-09
Majoor 2011	Published 2010-19
Makhmudova 2019	Published 2010-19

study	group
Mathew 2006	Published 2000-09
Millet 2010	Published 2010-19
Nebreda-Mayoral 2017	Published 2010-19
Panic 2003	Published 2000-09
Pedrazzoli 2019	Published 2010-19
Pina 2006	Published 2000-09
Podlekareva 2014	Published 2010-19
Podlekareva 2016	Published 2010-19
Pradipta 2019a	Published 2010-19
Public Health England 2013	Published 2010-19
Public Health England 2014a	Published 2010-19
Public Health England 2015	Published 2010-19
Public Health England 2016	Published 2010-19
Public Health England 2020b	Published 2020-
Public Health Ukraine 2017	Published 2010-19
RKI 2014	Published 2010-19
RKI 2017	Published 2010-19
RKI 2019	Published 2010-19
RKI 2020	Published 2020-
Rodriguez-Valin 2015	Published 2010-19
Shuldiner 2014	Published 2010-19
Shuldiner 2016	Published 2010-19
Talay 2008	Published 2000-09
Valade 2012	Published 2010-19
Vasankari 2007	Published 2000-09
WHO 2020	Published 2020-

Group file regions

study	group
Abutidze 2012	Eastern Europe
Aguado 1997	Western Europe
Balabanova 2016	Eastern Europe
Bartu 2010	Central Europe
Bastos 2016	Western Europe
Baussano 2008	Western Europe
Bendayan 2011	Western Europe
Bloendal 2013	Eastern Europe
Borgdorff 1998	Western Europe
Cayla 2003	Western Europe
Crofts 2008	Western Europe
Daucourt 2000	Western Europe
Dewan 2004	Eastern Europe
Diacon 2014	Eastern Europe
Diel 2003	Western Europe
Dobrotkova 2019	Central Europe
Duro 2017	Western Europe
Erbes 2006	Western Europe
Farah 2005	Western Europe
Faustini 2008	Western Europe
Floe 2017	Western Europe
Fortun 2014	Western Europe
Franco Spinola 2015	Western Europe
Frank 2019	Eastern Europe
Gadoev 2015	Eastern Europe
GBD Tuberculosis Collaborator 2014 (CE)	Central Europe
GBD Tuberculosis Collaborator 2014 (EE)	Eastern Europe
GBD Tuberculosis Collaborator 2014 (WE)	Western Europe
Girardi 2012	Western Europe
Girardi 2014	Western Europe
Haar 2007	Western Europe
Helbling 2002	Western Europe
Holden 2020	Western Europe
Holmberg 2019	Western Europe
Khaliaukin 2014	Eastern Europe
Kherosheva 2003	Eastern Europe
Korhonen 2020	Western Europe
Korzeniewska-Kosela 2017	Central Europe
Korzeniewska-Kosela 2018	Central Europe
Korzeniewska-Kosela 2019	Central Europe
Korzeniewska-Kosela 2020	Central Europe
Kourbatova 2006	Eastern Europe
Lanoix 2014	Western Europe
Lockman 2001	Eastern Europe
Lowe 2013	Western Europe
Loytved 2002	Western Europe
Lubart 2007	Western Europe
Majoor 2011	Western Europe
Makhmudova 2019	Eastern Europe

study	group
Mathew 2006	Eastern Europe
Millet 2010	Western Europe
Nebreda-Mayoral 2017	Western Europe
Panic 2003	Central Europe
Pedrazzoli 2019	Western Europe
Pina 2006	Western Europe
Podlekareva 2014	Eastern Europe
Podlekareva 2016	Europe
Pradipta 2019a	Western Europe
Public Health England 2013	Western Europe
Public Health England 2014a	Western Europe
Public Health England 2015	Western Europe
Public Health England 2020a	Western Europe
Public Health England 2020b	Western Europe
Public Health Ukraine 2017	Eastern Europe
RKI 2014	Western Europe
RKI 2017	Western Europe
RKI 2019	Western Europe
RKI 2020	Western Europe
Rodriguez-Valin 2015	Western Europe
Shuldiner 2014	Western Europe
Shuldiner 2016	Western Europe
Talay 2008	Central Europe
Valade 2012	Western Europe
Vasankari 2007	Western Europe
WHO 2020	Europe

Group file study design

study	group
Abutidze 2012	Cohort
Aguado 1997	Cohort
Balabanova 2016	Cohort
Bartu 2010	Cross-Sectional
Bastos 2016	Cohort
Baussano 2008	Cohort
Bendayan 2011	Cohort
Bloendal 2013	Cohort
Borgdorff 1998	Cohort
Cayla 2003	Cohort
Crofts 2008	Descriptive
Daucourt 2000	Cohort
Dewan 2004	Case-Control
Diacon 2014	Interventional (Trial)
Diel 2003	Cohort
Dobrotkova 2019	Cross-Sectional
Duro 2017	Cross-Sectional
Erbes 2006	Descriptive
Farah 2005	Cohort
Faustini 2008	Case-Control
Floe 2017	Case-Control
Fortun 2014	Cross-Sectional
Franco Spinola 2015	Case-Control
Frank 2019	Cohort
Gadoev 2015	Cohort
GBD Tuberculosis Collaborator 2014 (CE)	Cross-Sectional
GBD Tuberculosis Collaborator 2014 (EE)	Cross-Sectional
GBD Tuberculosis Collaborator 2014 (WE)	Cross-Sectional
Girardi 2012	Cohort
Girardi 2014	Cross-Sectional
Haar 2007	Cohort
Helbling 2002	Cohort
Holden 2020	Cohort
Holmberg 2019	Cohort
Khaliaukin 2014	Cohort
Kherosheva 2003	Descriptive
Korhonen 2020	Cohort
Korzeniewska-Kosela 2017	Descriptive
Korzeniewska-Kosela 2018	Descriptive
Korzeniewska-Kosela 2019	Descriptive
Korzeniewska-Kosela 2020	Descriptive
Kourbatova 2006	Case-Control
Lanoix 2014	Cross-Sectional
Lockman 2001	Case-Control
Lowe 2013	Cross-Sectional
Loytved 2002	Cross-Sectional
Lubart 2007	Cross-Sectional
Majoor 2011	Descriptive
Makhmudova 2019	Cross-Sectional

study	group
Mathew 2006	Descriptive
Millet 2010	Cohort
Nebreda-Mayoral 2017	Cross-Sectional
Panic 2003	Descriptive
Pedrazzoli 2019	Cohort
Pina 2006	Cross-Sectional
Podlekareva 2014	Cohort
Podlekareva 2016	Cohort
Pradipta 2019a	Cohort
Public Health England 2013	Descriptive
Public Health England 2014a	Descriptive
Public Health England 2015	Descriptive
Public Health England 2016	Descriptive
Public Health England 2020b	Descriptive
Public Health Ukraine 2017	Descriptive
RKI 2014	Descriptive
RKI 2017	Descriptive
RKI 2019	Descriptive
RKI 2020	Descriptive
Rodriguez-Valin 2015	Cross-Sectional
Shuldiner 2014	Cohort
Shuldiner 2016	Cohort
Talay 2008	Cohort
Valade 2012	Cross-Sectional
Vasankari 2007	Cross-Sectional
WHO 2020	Descriptive

Group file study population

study	group
Abutidze 2012	Persons living with HIV
Aguado 1997	Specific patients (hospitalized)
Balabanova 2016	Drug-resistant TB cases
Bartu 2010	Drug-resistant TB cases
Bastos 2016	Specific patients (hospitalized)
Baussano 2008	TB cases, all forms
Bendayan 2011	Drug-resistant TB cases
Bloendal 2013	Drug-resistant TB cases
Borgdorff 1998	TB cases, all forms
Cayla 2003	TB cases, all forms
Crofts 2008	TB cases, all forms
Daucourt 2000	TB cases, all forms
Dewan 2004	TB cases, all forms
Diacon 2014	Drug-resistant TB cases
Diel 2003	TB cases, all forms
Dobrotkova 2019	TB cases, all forms
Duro 2017	Specific patients (hospitalized)
Erbes 2006	Specific patients (hospitalized)
Farah 2005	TB cases, all forms
Faustini 2008	TB cases, all forms
Floe 2017	TB cases, all forms
Fortun 2014	Specific patients (hospitalized)
Franco Spinola 2015	TB cases, all forms
Frank 2019	Drug-resistant TB cases
Gadoev 2015	TB cases, all forms
GBD Tuberculosis Collaborator 2014 (CE)	TB cases, all forms
GBD Tuberculosis Collaborator 2014 (EE)	TB cases, all forms
GBD Tuberculosis Collaborator 2014 (WE)	TB cases, all forms
Girardi 2012	Persons living with HIV
Girardi 2014	TB cases, all forms
Haar 2007	Persons living with HIV
Helbling 2002	TB cases, all forms
Holden 2020	TB cases, all forms
Holmberg 2019	Persons living with HIV
Khaliaukin 2014	Drug-resistant TB cases
Kherosheva 2003	TB cases, all forms
Korhonen 2020	TB cases, all forms
Korzeniewska-Kosela 2017	TB cases, all forms
Korzeniewska-Kosela 2018	TB cases, all forms
Korzeniewska-Kosela 2019	TB cases, all forms
Korzeniewska-Kosela 2020	TB cases, all forms
Kourbatova 2006	TB cases, all forms
Lanoix 2014	Specific patients (hospitalized)
Lockman 2001	Drug-resistant TB cases
Lowe 2013	Specific patients (hospitalized)
Loytved 2002	TB cases, all forms
Lubart 2007	Specific patients (hospitalized)
Majoor 2011	Notified M. bovis cases
Makhmudova 2019	Drug-resistant TB cases

study	group
Mathew 2006	TB cases, all forms
Millet 2010	TB cases, all forms
Nebreda-Mayoral 2017	Notified M. bovis cases
Panic 2003	TB cases, all forms
Pedrazzoli 2019	TB cases, all forms
Pina 2006	TB cases, all forms
Podlekareva 2014	Persons living with HIV
Podlekareva 2016	Persons living with HIV
Pradipta 2019a	TB cases, all forms
Public Health England 2013	TB cases, all forms
Public Health England 2014b	TB cases, all forms
Public Health England 2015	TB cases, all forms
Public Health England 2016	TB cases, all forms
Public Health England 2020b	TB cases, all forms
Public Health Ukraine 2017	TB cases, all forms
RKI 2014	TB cases, all forms
RKI 2017	TB cases, all forms
RKI 2019	TB cases, all forms
RKI 2020	TB cases, all forms
Rodriguez-Valin 2015	TB cases, all forms
Shuldiner 2014	TB cases, all forms
Shuldiner 2016	TB cases, all forms
Talay 2008	TB cases, all forms
Valade 2012	Specific patients (hospitalized)
Vasankari 2007	TB cases, all forms
WHO 2020	TB cases, all forms

Dataset n = 67 (sensitivity analysis)

Group file countries

study	group
Abutidze 2012	Georgia
Aguado 1997	Spain
Balabanova 2016	Eastern Europe
Bastos 2016	Portugal
Baussano 2008	Italy
Bendayan 2011	Israel
Bloendal 2013	Estonia
Borgdorff 1998	Netherlands
Cayla 2003	Spain
Crofts 2008	United Kingdom
Daucourt 2000	France
Dewan 2004	Russia
Diacon 2014	Eastern Europe
Diel 2003	Germany
Dobrotkova 2019	Slovakia
Erbes 2006	Germany
Farah 2005	Norway
Faustini 2008	Italy
Floe 2017	Denmark
Fortun 2014	Spain
Franco Spinola 2015	Portugal
Frank 2019	Georgia
Gadoev 2015	Uzbekistan
GBD Tuberculosis Collaborator 2014 (CE)	Central Europe
GBD Tuberculosis Collaborator 2014 (EE)	Eastern Europe
GBD Tuberculosis Collaborator 2014 (WE)	Western Europe
Girardi 2012	Italy
Girardi 2014	France
Haar 2007	Netherlands
Holden 2020	Denmark
Khaliaukin 2014	Belarus
Korhonen 2020	Finland
Korzeniewska-Kosela 2017	Poland
Korzeniewska-Kosela 2018	Poland
Korzeniewska-Kosela 2019	Poland
Korzeniewska-Kosela 2020	Poland
Kourbatova 2006	Russia
Lanoix 2014	France
Lowe 2013	United Kingdom
Loytved 2002	Germany
Lubart 2007	Israel
Majoor 2011	Netherlands
Makhmudova 2019	Tajikistan
Mathew 2006	Russia
Millet 2010	Spain
Panic 2003	Yugoslavia
Pedrazzoli 2019	United Kingdom

study	group
Pina 2006	Spain
Podlekareva 2014	Eastern Europe
Podlekareva 2016	Europe
Pradipta 2019a	Netherlands
Public Health England 2013	United Kingdom
Public Health England 2014a	United Kingdom
Public Health England 2015	United Kingdom
Public Health England 2016	United Kingdom
Public Health England 2020b	United Kingdom
Public Health Ukraine 2017	Ukraine
RKI 2014	Germany
RKI 2017	Germany
RKI 2019	Germany
RKI 2020	Germany
Rodriguez-Valin 2015	Spain
Shuldiner 2014	Israel
Shuldiner 2016	Israel
Valade 2012	France
Vasankari 2007	Finland
WHO 2020	Europe

Group file data source

study	group
Abutidze 2012	Hospital data
Aguado 1997	Hospital data
Balabanova 2016	TB dispensary data
Bastos 2016	TB dispensary data
Baussano 2008	Notification data
Bendayan 2011	Hospital data
Bloendal 2013	Notification data
Borgdorff 1998	Notification data
Cayla 2003	Hospital data
Crofts 2008	Notification data
Daucourt 2000	Notification data
Dewan 2004	TB dispensary data
Diacon 2014	Hospital data
Diel 2003	Notification data
Dobrotkova 2019	Notification data
Erbes 2006	Hospital data
Farah 2005	Notification data
Faustini 2008	Notification data
Floe 2017	Notification data
Fortun 2014	Hospital data
Franco Spinola 2015	Notification data
Frank 2019	Notification data
Gadoev 2015	Notification data
GBD Tuberculosis Collaborator 2014 (CE)	Notification data
GBD Tuberculosis Collaborator 2014 (EE)	Notification data
GBD Tuberculosis Collaborator 2014 (WE)	Notification data
Girardi 2012	Hospital data
Girardi 2014	Notification data
Haar 2007	Notification data
Holden 2020	Notification data
Khaliaukin 2014	Hospital data
Korhonen 2020	Notification data
Korzeniewska-Kosela 2017	Notification data
Korzeniewska-Kosela 2018	Notification data
Korzeniewska-Kosela 2019	Notification data
Korzeniewska-Kosela 2020	Notification data
Kourbatova 2006	TB dispensary data
Lanoix 2014	Notification data
Lowe 2013	Hospital data
Loytved 2002	Notification data
Lubart 2007	Hospital data
Majoor 2011	Notification data
Makhmudova 2019	Hospital data
Mathew 2006	Notification data
Millet 2010	Notification data
Panic 2003	Hospital data
Pedrazzoli 2019	Notification data
Pina 2006	Notification data
Podlekareva 2014	Hospital data

study	group
Podlekareva 2016	Hospital data
Pradipta 2019a	Notification data
Public Health England 2013	Notification data
Public Health England 2014a	Notification data
Public Health England 2015	Notification data
Public Health England 2016	Notification data
Public Health England 2020b	Notification data
Public Health Ukraine 2017	Notification data
RKI 2014	Notification data
RKI 2017	Notification data
RKI 2019	Notification data
RKI 2020	Notification data
Rodriguez-Valin 2015	Notification data
Shuldiner 2014	TB dispensary data
Shuldiner 2016	Notification data
Valade 2012	Hospital data
Vasankari 2007	Hospital data
WHO 2020	Notification data

Group file group size

study	group
Abutidze 2012	Group size less than 30
Aguado 1997	Group size less than 30
Balabanova 2016	Group size equal or more than 30
Bastos 2016	Group size equal or more than 30
Baussano 2008	Group size equal or more than 30
Bendayan 2011	Group size equal or more than 30
Bloendal 2013	Group size equal or more than 30
Borgdorff 1998	Group size equal or more than 30
Cayla 2003	Group size equal or more than 30
Crofts 2008	Group size equal or more than 30
Daucourt 2000	Group size equal or more than 30
Dewan 2004	Group size equal or more than 30
Diacon 2014	Group size equal or more than 30
Diel 2003	Group size equal or more than 30
Dobrotkova 2019	Group size equal or more than 30
Erbes 2006	Group size less than 30
Farah 2005	Group size equal or more than 30
Faustini 2008	Group size equal or more than 30
Floe 2017	Group size equal or more than 30
Fortun 2014	Group size equal or more than 30
Franco Spinola 2015	Group size equal or more than 30
Frank 2019	Group size equal or more than 30
Gadoev 2015	Group size equal or more than 30
GBD Tuberculosis Collaborator 2014 (CE)	Group size equal or more than 30
GBD Tuberculosis Collaborator 2014 (EE)	Group size equal or more than 30
GBD Tuberculosis Collaborator 2014 (WE)	Group size equal or more than 30
Girardi 2012	Group size equal or more than 30
Girardi 2014	Group size equal or more than 30
Haar 2007	Group size equal or more than 30
Holden 2020	Group size equal or more than 30
Khaliaukin 2014	Group size equal or more than 30
Korhonen 2020	Group size equal or more than 30
Korzeniewska-Kosela 2017	Group size equal or more than 30
Korzeniewska-Kosela 2018	Group size equal or more than 30
Korzeniewska-Kosela 2019	Group size equal or more than 30
Korzeniewska-Kosela 2020	Group size equal or more than 30
Kourbatova 2006	Group size equal or more than 30
Lanoix 2014	Group size less than 30
Lowe 2013	Group size equal or more than 30
Loytved 2002	Group size equal or more than 30
Lubart 2007	Group size equal or more than 30
Majoor 2011	Group size equal or more than 30
Makhmudova 2019	Group size equal or more than 30
Mathew 2006	Group size equal or more than 30
Millet 2010	Group size equal or more than 30
Panic 2003	Group size equal or more than 30
Pedrazzoli 2019	Group size equal or more than 30
Pina 2006	Group size equal or more than 30
Podlekareva 2014	Group size equal or more than 30

study	group
Podlekareva 2016	Group size equal or more than 30
Pradipta 2019a	Group size equal or more than 30
Public Health England 2013	Group size equal or more than 30
Public Health England 2014a	Group size equal or more than 30
Public Health England 2015	Group size equal or more than 30
Public Health England 2016	Group size equal or more than 30
Public Health England 2020b	Group size equal or more than 30
Public Health Ukraine 2017	Group size equal or more than 30
RKI 2014	Group size equal or more than 30
RKI 2017	Group size equal or more than 30
RKI 2019	Group size equal or more than 30
RKI 2020	Group size equal or more than 30
Rodriguez-Valin 2015	Group size equal or more than 30
Shuldiner 2014	Group size equal or more than 30
Shuldiner 2016	Group size equal or more than 30
Valade 2012	Group size less than 30
Vasankari 2007	Group size equal or more than 30
WHO 2020	Group size equal or more than 30

Group file observation period

study	group
Abutidze 2012	Observation period: 2000s
Aguado 1997	Observation period: 1980s-1990s
Balabanova 2016	Observation period: 2000s-2010s
Bastos 2016	Observation period: 2000s-2010s
Baussano 2008	Observation period: 2000s
Bendayan 2011	Observation period: 2000s
Bloendal 2013	Observation period: 2000s-2010s
Borgdorff 1998	Observation period: 1990s
Cayla 2003	Observation period: 1990s-2000s
Crofts 2008	Observation period: 2000s
Daucourt 2000	Observation period: 1990s
Dewan 2004	Observation period: 1990s-2000s
Diacon 2014	Observation period: 2000s-2010s
Diel 2003	Observation period: 1990s-2000s
Dobrotkova 2019	Observation period: 2000s-2010s
Erbes 2006	Observation period: 1990s-2000s
Farah 2005	Observation period: 1990s-2000s
Faustini 2008	Observation period: 1990s-2000s
Floe 2017	Observation period: 1990s-2010s
Fortun 2014	Observation period: 1990s-2000s
Franco Spinola 2015	Observation period: 2000s-2010s
Frank 2019	Observation period: 2010s
Gadoev 2015	Observation period: 2000s-2010s
GBD Tuberculosis Collaborator 2014 (CE)	Observation period: 1990s-2010s
GBD Tuberculosis Collaborator 2014 (EE)	Observation period: 1990s-2010s
GBD Tuberculosis Collaborator 2014 (WE)	Observation period: 1990s-2010s
Girardi 2012	Observation period: 1990s-2000s
Girardi 2014	Observation period: 2010s
Haar 2007	Observation period: 1990s-2000s
Holden 2020	Observation period: 2000s-2010s
Khaliaukin 2014	Observation period: 2000s-2010s
Korhonen 2020	Observation period: 2000s-2010s
Korzeniewska-Kosela 2017	Observation period: 2010s
Korzeniewska-Kosela 2018	Observation period: 2010s
Korzeniewska-Kosela 2019	Observation period: 2010s
Korzeniewska-Kosela 2020	Observation period: 2010s
Kourbatova 2006	Observation period: 1990s-2000s
Lanoix 2014	Observation period: 2000s
Lowe 2013	Observation period: 1990s-2000s
Loytved 2002	Observation period: 1990s-2000s
Lubart 2007	Observation period: 2000s
Majoor 2011	Observation period: 1990s-2000s
Makhmudova 2019	Observation period: 2010s
Mathew 2006	Observation period: 2000s
Millet 2010	Observation period: 1990s-2000s
Panic 2003	Observation period: 1990s
Pedrazzoli 2019	Observation period: 2000s-2010s
Pina 2006	Observation period: 1990s
Podlekareva 2014	Observation period: 2000s-2010s

study	group
Podlekareva 2016	Observation period: 2010s
Pradipta 2019a	Observation period: 2000s-2010s
Public Health England 2013	Observation period: 2010s
Public Health England 2014a	Observation period: 2010s
Public Health England 2015	Observation period: 2010s
Public Health England 2016	Observation period: 2010s
Public Health England 2020b	Observation period: 2010s
Public Health Ukraine 2017	Observation period: 2010s
RKI 2014	Observation period: 2010s
RKI 2017	Observation period: 2010s
RKI 2019	Observation period: 2010s
RKI 2020	Observation period: 2010s
Rodriguez-Valin 2015	Observation period: 2010s
Shuldiner 2014	Observation period: 2000s-2010s
Shuldiner 2016	Observation period: 2000s-2010s
Valade 2012	Observation period: 2000s
Vasankari 2007	Observation period: 1990s
WHO 2020	Observation period: 2010s

Group file publication date

study	group
Abutidze 2012	Published 2010-19
Aguado 1997	Published 1990-99
Balabanova 2016	Published 2010-19
Bastos 2016	Published 2010-19
Baussano 2008	Published 2000-09
Bendayan 2011	Published 2010-19
Bloendal 2013	Published 2010-19
Borgdorff 1998	Published 1990-99
Cayla 2003	Published 2000-09
Crofts 2008	Published 2000-09
Daucourt 2000	Published 2000-09
Dewan 2004	Published 2000-09
Diacon 2014	Published 2010-19
Diel 2003	Published 2000-09
Dobrotkova 2019	Published 2010-19
Erbes 2006	Published 2000-09
Farah 2005	Published 2000-09
Faustini 2008	Published 2000-09
Floe 2017	Published 2010-19
Fortun 2014	Published 2010-19
Franco Spinola 2015	Published 2010-19
Frank 2019	Published 2010-19
Gadoev 2015	Published 2010-19
GBD Tuberculosis Collaborator 2014 (CE)	Published 2010-19
GBD Tuberculosis Collaborator 2014 (EE)	Published 2010-19
GBD Tuberculosis Collaborator 2014 (WE)	Published 2010-19
Girardi 2012	Published 2010-19
Girardi 2014	Published 2010-19
Haar 2007	Published 2000-09
Holden 2020	Published 2020-
Khaliaukin 2014	Published 2010-19
Korhonen 2020	Published 2020-
Korzeniewska-Kosela 2017	Published 2010-19
Korzeniewska-Kosela 2018	Published 2010-19
Korzeniewska-Kosela 2019	Published 2010-19
Korzeniewska-Kosela 2020	Published 2020-
Kourbatova 2006	Published 2000-09
Lanoix 2014	Published 2010-19
Lowe 2013	Published 2010-19
Loytved 2002	Published 2000-09
Lubart 2007	Published 2000-09
Majoor 2011	Published 2010-19
Makhmudova 2019	Published 2010-19
Mathew 2006	Published 2000-09
Millet 2010	Published 2010-19
Panic 2003	Published 2000-09
Pedrazzoli 2019	Published 2010-19
Pina 2006	Published 2000-09
Podlekareva 2014	Published 2010-19

study	group
Podlekareva 2016	Published 2010-19
Pradipta 2019a	Published 2010-19
Public Health England 2013	Published 2010-19
Public Health England 2014a	Published 2010-19
Public Health England 2015	Published 2010-19
Public Health England 2016	Published 2010-19
Public Health England 2020b	Published 2020-
Public Health Ukraine 2017	Published 2010-19
RKI 2014	Published 2010-19
RKI 2017	Published 2010-19
RKI 2019	Published 2010-19
RKI 2020	Published 2020-
Rodriguez-Valin 2015	Published 2010-19
Shuldiner 2014	Published 2010-19
Shuldiner 2016	Published 2010-19
Valade 2012	Published 2010-19
Vasankari 2007	Published 2000-09
WHO 2020	Published 2020-

Group file regions

study	group
Abutidze 2012	Eastern Europe
Aguado 1997	Western Europe
Balabanova 2016	Eastern Europe
Bastos 2016	Western Europe
Baussano 2008	Western Europe
Bendayan 2011	Western Europe
Bloendal 2013	Eastern Europe
Borgdorff 1998	Western Europe
Cayla 2003	Western Europe
Crofts 2008	Western Europe
Daucourt 2000	Western Europe
Dewan 2004	Eastern Europe
Diacon 2014	Eastern Europe
Diel 2003	Western Europe
Dobrotkova 2019	Central Europe
Erbes 2006	Western Europe
Farah 2005	Western Europe
Faustini 2008	Western Europe
Floe 2017	Western Europe
Fortun 2014	Western Europe
Franco Spinola 2015	Western Europe
Frank 2019	Eastern Europe
Gadoev 2015	Eastern Europe
GBD Tuberculosis Collaborator 2014 (CE)	Central Europe
GBD Tuberculosis Collaborator 2014 (EE)	Eastern Europe
GBD Tuberculosis Collaborator 2014 (WE)	Western Europe
Girardi 2012	Western Europe
Girardi 2014	Western Europe
Haar 2007	Western Europe
Holden 2020	Western Europe
Khaliaukin 2014	Eastern Europe
Korhonen 2020	Western Europe
Korzeniewska-Kosela 2017	Central Europe
Korzeniewska-Kosela 2018	Central Europe
Korzeniewska-Kosela 2019	Central Europe
Korzeniewska-Kosela 2020	Central Europe
Kourbatova 2006	Eastern Europe
Lanoix 2014	Western Europe
Lowe 2013	Western Europe
Loytved 2002	Western Europe
Lubart 2007	Western Europe
Majoor 2011	Western Europe
Makhmudova 2019	Eastern Europe
Mathew 2006	Eastern Europe
Millet 2010	Western Europe
Panic 2003	Central Europe
Pedrazzoli 2019	Western Europe
Pina 2006	Western Europe
Podlekareva 2014	Eastern Europe

study	group
Podlekareva 2016	Europe
Pradipta 2019a	Western Europe
Public Health England 2013	Western Europe
Public Health England 2014a	Western Europe
Public Health England 2015	Western Europe
Public Health England 2020a	Western Europe
Public Health England 2020b	Western Europe
Public Health Ukraine 2017	Eastern Europe
RKI 2014	Western Europe
RKI 2017	Western Europe
RKI 2019	Western Europe
RKI 2020	Western Europe
Rodriguez-Valin 2015	Western Europe
Shuldiner 2014	Western Europe
Shuldiner 2016	Western Europe
Valade 2012	Western Europe
Vasankari 2007	Western Europe
WHO 2020	Europe

Group file study design

study	group
Abutidze 2012	Cohort
Aguado 1997	Cohort
Balabanova 2016	Cohort
Bastos 2016	Cohort
Baussano 2008	Cohort
Bendayan 2011	Cohort
Bloendal 2013	Cohort
Borgdorff 1998	Cohort
Cayla 2003	Cohort
Crofts 2008	Descriptive
Daucourt 2000	Cohort
Dewan 2004	Case-Control
Diacon 2014	Interventional (Trial)
Diel 2003	Cohort
Dobrotkova 2019	Cross-Sectional
Erbes 2006	Descriptive
Farah 2005	Cohort
Faustini 2008	Case-Control
Floe 2017	Case-Control
Fortun 2014	Cross-Sectional
Franco Spinola 2015	Case-Control
Frank 2019	Cohort
Gadoev 2015	Cohort
GBD Tuberculosis Collaborator 2014 (CE)	Cross-Sectional
GBD Tuberculosis Collaborator 2014 (EE)	Cross-Sectional
GBD Tuberculosis Collaborator 2014 (WE)	Cross-Sectional
Girardi 2012	Cohort
Girardi 2014	Cross-Sectional
Haar 2007	Cohort
Holden 2020	Cohort
Khaliaukin 2014	Cohort
Korhonen 2020	Cohort
Korzeniewska-Kosela 2017	Descriptive
Korzeniewska-Kosela 2018	Descriptive
Korzeniewska-Kosela 2019	Descriptive
Korzeniewska-Kosela 2020	Descriptive
Kourbatova 2006	Case-Control
Lanoix 2014	Cross-Sectional
Lowe 2013	Cross-Sectional
Loytved 2002	Cross-Sectional
Lubart 2007	Cross-Sectional
Majoor 2011	Descriptive
Makhmudova 2019	Cross-Sectional
Mathew 2006	Descriptive
Millet 2010	Cohort
Panic 2003	Descriptive
Pedrazzoli 2019	Cohort
Pina 2006	Cross-Sectional
Podlekareva 2014	Cohort

study	group
Podlekareva 2016	Cohort
Pradipta 2019a	Cohort
Public Health England 2013	Descriptive
Public Health England 2014a	Descriptive
Public Health England 2015	Descriptive
Public Health England 2016	Descriptive
Public Health England 2020b	Descriptive
Public Health Ukraine 2017	Descriptive
RKI 2014	Descriptive
RKI 2017	Descriptive
RKI 2019	Descriptive
RKI 2020	Descriptive
Rodriguez-Valin 2015	Cross-Sectional
Shuldiner 2014	Cohort
Shuldiner 2016	Cohort
Valade 2012	Cross-Sectional
Vasankari 2007	Cross-Sectional
WHO 2020	Descriptive

Group file study population

study	group
Abutidze 2012	Persons living with HIV
Aguado 1997	Specific patients (hospitalized)
Balabanova 2016	Drug-resistant TB cases
Bastos 2016	Specific patients (hospitalized)
Baussano 2008	TB cases, all forms
Bendayan 2011	Drug-resistant TB cases
Bloendal 2013	Drug-resistant TB cases
Borgdorff 1998	TB cases, all forms
Cayla 2003	TB cases, all forms
Crofts 2008	TB cases, all forms
Daucourt 2000	TB cases, all forms
Dewan 2004	TB cases, all forms
Diacon 2014	Drug-resistant TB cases
Diel 2003	TB cases, all forms
Dobrotkova 2019	TB cases, all forms
Erbes 2006	Specific patients (hospitalized)
Farah 2005	TB cases, all forms
Faustini 2008	TB cases, all forms
Floe 2017	TB cases, all forms
Fortun 2014	Specific patients (hospitalized)
Franco Spinola 2015	TB cases, all forms
Frank 2019	Drug-resistant TB cases
Gadoev 2015	TB cases, all forms
GBD Tuberculosis Collaborator 2014 (CE)	TB cases, all forms
GBD Tuberculosis Collaborator 2014 (EE)	TB cases, all forms
GBD Tuberculosis Collaborator 2014 (WE)	TB cases, all forms
Girardi 2012	Persons living with HIV
Girardi 2014	TB cases, all forms
Haar 2007	Persons living with HIV
Holden 2020	TB cases, all forms
Khaliaukin 2014	Drug-resistant TB cases
Korhonen 2020	TB cases, all forms
Korzeniewska-Kosela 2017	TB cases, all forms
Korzeniewska-Kosela 2018	TB cases, all forms
Korzeniewska-Kosela 2019	TB cases, all forms
Korzeniewska-Kosela 2020	TB cases, all forms
Kourbatova 2006	TB cases, all forms
Lanoix 2014	Specific patients (hospitalized)
Lowe 2013	Specific patients (hospitalized)
Loytved 2002	TB cases, all forms
Lubart 2007	Specific patients (hospitalized)
Majoor 2011	Notified M. bovis cases
Makhmudova 2019	Drug-resistant TB cases
Mathew 2006	TB cases, all forms
Millet 2010	TB cases, all forms
Panic 2003	TB cases, all forms
Pedrazzoli 2019	TB cases, all forms
Pina 2006	TB cases, all forms
Podlekareva 2014	Persons living with HIV

study	group
Podlekareva 2016	Persons living with HIV
Pradipta 2019a	TB cases, all forms
Public Health England 2013	TB cases, all forms
Public Health England 2014b	TB cases, all forms
Public Health England 2015	TB cases, all forms
Public Health England 2016	TB cases, all forms
Public Health England 2020b	TB cases, all forms
Public Health Ukraine 2017	TB cases, all forms
RKI 2014	TB cases, all forms
RKI 2017	TB cases, all forms
RKI 2019	TB cases, all forms
RKI 2020	TB cases, all forms
Rodriguez-Valin 2015	TB cases, all forms
Shuldiner 2014	TB cases, all forms
Shuldiner 2016	TB cases, all forms
Valade 2012	Specific patients (hospitalized)
Vasankari 2007	TB cases, all forms
WHO 2020	TB cases, all forms

II. *Studies reporting hazard ratios*

Primary files

Legend of primary files: study = publication identifier comprising the first author and publication year; HR = hazard ratio of male to female TB fatality hazards; lower = lower 95% confidence interval of the hazard ratio; upper = upper 95% confidence interval of the hazard ratio.

study	HR	lower	upper
Andreychyn 2013	2.00	0.63	6.25
Balabanova 2011	1.41	0.95	2.08
Girardi 2001	1.82	0.76	4.35

III. Studies reporting mortality rates

Primary files

Legend of primary files: study = publication identifier comprising the first author and publication year; event.e = male TB mortality rate (per 100,000 population); n.e = male TB incidence rate (per 100,000 population); event.c = female TB mortality rate (per 100,000 population); n.c = female TB incidence rate (per 100,000 population).

For the datasets with mortality rates recalculated in absolute numbers: event.e = numbers of male TB fatalities (per 10,000,000 population); n.e = numbers of males w/o a TB fatality event (per 10,000,000 population); event.c = numbers of female TB fatalities (per 10,000,000 population); n.c = numbers of females w/o a TB fatality event (per 10,000,000 population).

Dataset n = 15 using TB mortality and incidence rates

study	event.e	n.e	event.c	n.c
Brodhun 2015	0.24	6.6	0.12	3.9
Brya 2017	3.53	3.53	0.98	0.98
Gledovic 2006	6	43.9	2.3	25.4
Hauer 2011	1.2	11.2	0.7	6.8
Korzeniewska-Kosela 2010	3.2	29.8	0.8	13.2
Korzeniewska-Kosela 2011	3.3	30.1	0.9	13.6
Korzeniewska-Kosela 2012	3.2	28	0.8	11.9
Korzeniewska-Kosela 2013	2.5	31	0.6	14
Korzeniewska-Kosela 2014	2.7	27.4	0.7	12.2
Korzeniewska-Kosela 2015	2.6	26.8	0.8	11.4
Korzeniewska-Kosela 2016	2.2	24.6	0.6	10.7
Szczuka 2006	3.5	34.1	0.8	14.2
Szczuka 2007	3.4	32.9	0.9	16.7
Szczuka 2008	3.4	30.9	0.9	14.7
Szczuka 2009	3.1	31.4	0.8	14.4

Dataset n = 14 (w/o Brya 2017) using TB mortality and incidence rates

study	event.e	n.e	event.c	n.c
Brodhun 2015	0.24	6.6	0.12	3.9
Gledovic 2006	6	43.9	2.3	25.4
Hauer 2011	1.2	11.2	0.7	6.8
Korzeniewska-Kosela 2010	3.2	29.8	0.8	13.2
Korzeniewska-Kosela 2011	3.3	30.1	0.9	13.6
Korzeniewska-Kosela 2012	3.2	28	0.8	11.9
Korzeniewska-Kosela 2013	2.5	31	0.6	14
Korzeniewska-Kosela 2014	2.7	27.4	0.7	12.2
Korzeniewska-Kosela 2015	2.6	26.8	0.8	11.4
Korzeniewska-Kosela 2016	2.2	24.6	0.6	10.7
Szczuka 2006	3.5	34.1	0.8	14.2
Szczuka 2007	3.4	32.9	0.9	16.7
Szczuka 2008	3.4	30.9	0.9	14.7
Szczuka 2009	3.1	31.4	0.8	14.4

Dataset n = 15 using absolute numbers recalculated from TB mortality rates (TB fatalities per 10,000,000 population)

study	event.e	n.e	event.c	n.c
Brodhun 2015	24	10000000	12	10000000
Brya 2017	353	10000000	98	10000000
Gledovic 2006	600	10000000	230	10000000
Hauer 2011	120	10000000	70	10000000
Korzeniewska-Kosela 2010	320	10000000	80	10000000
Korzeniewska-Kosela 2011	330	10000000	90	10000000
Korzeniewska-Kosela 2012	320	10000000	80	10000000
Korzeniewska-Kosela 2013	250	10000000	60	10000000
Korzeniewska-Kosela 2014	270	10000000	70	10000000
Korzeniewska-Kosela 2015	260	10000000	80	10000000
Korzeniewska-Kosela 2016	220	10000000	60	10000000
Szczuka 2006	350	10000000	80	10000000
Szczuka 2007	340	10000000	90	10000000
Szczuka 2008	340	10000000	90	10000000
Szczuka 2009	310	10000000	80	10000000

Dataset n = 14 (w/o Brya 2017) using absolute numbers recalculated from TB mortality rates (TB fatalities per 10,000,000 population)

study	event.e	n.e	event.c	n.c
Brodhun 2015	24	10000000	12	10000000
Gledovic 2006	600	10000000	230	10000000
Hauer 2011	120	10000000	70	10000000
Korzeniewska-Kosela 2010	320	10000000	80	10000000
Korzeniewska-Kosela 2011	330	10000000	90	10000000
Korzeniewska-Kosela 2012	320	10000000	80	10000000
Korzeniewska-Kosela 2013	250	10000000	60	10000000
Korzeniewska-Kosela 2014	270	10000000	70	10000000
Korzeniewska-Kosela 2015	260	10000000	80	10000000
Korzeniewska-Kosela 2016	220	10000000	60	10000000
Szczuka 2006	350	10000000	80	10000000
Szczuka 2007	340	10000000	90	10000000
Szczuka 2008	340	10000000	90	10000000
Szczuka 2009	310	10000000	80	10000000

IV. Studies reporting odds ratios

Primary files

Legend of the primary files: study = publication identifier comprising the first author and publication year; HR = odds ratio of male to female TB fatality odds; lower = lower 95% confidence interval of the odds ratio; upper = upper 95% confidence interval of the odds ratio.

study	HR	lower	upper
Aibana 2018	0.85	0.37	1.94
Anyama 2007	1.22	0.67	2.22
Babalik 2014	1.18	0.78	1.78
Bhering 2019	1.62	0.8	3.3
Cayla 2009	0.98	0.44	2.16
Milanov 2015	1.5	0.42	5.32
Stosic 2020	1.34	1.19	1.52

V. *Studies reporting standardized mortality rates*

Primary files

Legend of primary files: study = publication identifier comprising the first author and publication year; event.e = numbers of male TB fatalities (per 10,000,000 population); n.e = numbers of males w/o a TB fatality event (per 10,000,000 population); event.c = numbers of female TB fatalities (per 10,000,000 population); n.c = numbers of females w/o a TB fatality event (per 10,000,000 population).

study	event.e	n.e	event.c	n.c
Al-Rahamneh 2017	406	10000000	94	10000000
Shkolnikov 2001 (1991)	2160	10000000	220	10000000
Shkolnikov 2001 (1994)	3800	10000000	360	10000000
Shkolnikov 2001 (1998)	3820	10000000	430	10000000
Shkolnikov 2013 RUS (2010)	2510	10000000	520	10000000
Shkolnikov 2013 RUS (2003)	3910	10000000	590	10000000
Shkolnikov 2013 UK (2009)	60	10000000	30	10000000
