# Supplementary material for: Tuberculosis case fatality is higher in male than female patients in Europe: a systematic review and meta-analysis
Source: Infection. 2024 Mar 23;52(5):1775–86. doi: 10.1007/s15010-024-02206-z (PMC11499538; doi:10.1007/s15010-024-02206-z)
Supplement: Supplementary file 7 — Online Resource 7 Publications with male-to-female relative risk estimates of TB fatality higher than 2.0 (PDF 194 KB) [file 15010_2024_2206_MOESM7_ESM.pdf]

Table A- 8: Publications with extreme relative risk estimates

Author/ Year	Events (M)	Total (M)	Events (F)	Total (F)	Risk Ratio M/F	CI 95% lower	CI 95% upper
Bartu 2010 [27]	9	28	0	5	3.67	0.25	54.2
Duro 2017 [59]	18	29	3	10	2.07	0.77	5.56
Helbling 2002 [145]	19	176	4	89	2.40	0.84	6.85
Holmberg 2019 [78]	15	35	1	18	7.71	1.11	53.83
Kherosheva 2003 [82]	28	574	3	175	2.85	0.88	9.25
Lockman 2001 [97]	10	62	2	30	2.42	0.57	10.36
Nebreda-Mayoral 2017 [108]	6	47	1	28	3.57	0.45	28.18
Talay 2008 [29]	14	463	0	123	7.73	0.46	128.63
