# Supplementary material for: Tuberculosis case fatality is higher in male than female patients in Europe: a systematic review and meta-analysis
Source: Infection. 2024 Mar 23;52(5):1775–86. doi: 10.1007/s15010-024-02206-z (PMC11499538; doi:10.1007/s15010-024-02206-z)
Supplement: Supplementary file 8 — Online Resource 8 Variables encompassed by the meta-regression dataset and final meta-regression dataset (PDF 218 KB) [file 15010_2024_2206_MOESM8_ESM.pdf]

Table A- 4: Variables encompassed by the meta-regression dataset

**Variable**	**Type**	**Categories**	**Function/definition**
study	Nominal	Acc. publications	Study characteristic; last name of first author or name of authors' group plus year of publication as stated by the publishing institution
overall	Discrete	As reported by the publication	Total number of individuals comprised by the study population reported in the publication.
total.e	Discrete	As reported by the publication	Total number of male and female TB fatalities as reported in the publication
total.n	Discrete	As reported by the publication	Total number of male and female TB cases as reported in the publication
m.e	Discrete	As reported by the publication	Total number of male TB fatalities, as reported in the publication
m.n	Discrete	As reported by the publication	Total number of male TB cases as reported in the publication
m.ne	Continuous	Calculated based on abstracted data	The proportion of male TB fatalities (numerator: variable m.e) to the male TB cases (denominator: variable m.n); equals the risk of males dying w/ or because of TB.
f.e	Discrete	As reported by the publication	Total number of female TB fatalities, as reported in the publication
f.n	Discrete	As reported by the publication	Total number of female TB cases as reported in the publication
f.ne	Continuous	Values calculated based on abstracted data	The proportion of female TB fatalities (numerator: variable f.e) to the female TB cases (denominator: variable f.n); equals the risk of females dying w/ or because of TB
mf.e	Continuous	Values calculated based on abstracted data	The proportion of male TB fatalities (numerator: variable m.e) to female TB fatalities (denominator: variable f.e)
mf.n	Continuous	Values calculated based on abstracted data	The proportion of male TB cases (numerator: variable m.n) to the female TB cases (denominator: variable f.n)
rrmf	Continuous	Values calculated based on abstracted data	The relative risk of males compared to females to die w/ or because of TB
rrcilow	Continuous	Values calculated based on abstracted data	Lower 95% CI bound of the relative risk of males compared to females to die w/ or because of TB.
rrciup	Continuous	Values calculated based on abstracted data	Upper 95% CI bound of the relative risk of males compared to females to die w/ or because of TB.
lnte	Continuous	Values calculated based on abstracted data	Logarithmically transformed (natural logarithm ln) relative risks (variable rrmf)
sete	Continuous	Values calculated based on abstracted data	Standard error of the lnte point estimates, also on the ln scale
var	Continuous	Values calculated based on abstracted data	Variance of the lnte point estimates, also on the ln scale

Variable	Type	Categories	Function/definition
weights.reml	Continuous	Values calculated based on abstracted data	The weights of the individual publications derived from the meta-analysis of the dataset comprising studies reporting absolute numbers (n = 75)
meanage	Discrete	As reported by the publication	Mean age of the study population, as reported in the publication
medianage	Discrete	As reported by the publication	Median age of the study population, as reported in the publication
age1524.rel	Continuous	Values calculated based on abstracted data	The proportion of individuals aged 15 to 24 (numerator) to the total study population (denominator, variable overall)
age2544.rel	Continuous	Values calculated based on abstracted data	The proportion of individuals aged 25 to 44 (numerator) to the total study population (denominator, variable overall)
age1544.rel	Continuous	Values calculated based on abstracted data	The proportion of individuals aged 15 to 44 (numerator) to the total study population (denominator, variable overall)
age4564.rel	Continuous	Values calculated based on abstracted data	The proportion of individuals aged 45 to 64 (numerator) to the total study population (denominator, variable overall)
age65.rel	Continuous	Values calculated based on abstracted data	The proportion of individuals aged 65 and over (numerator) to the total study population (denominator, variable overall)
foreignor.rel	Continuous	Values calculated based on abstracted data	The proportion of individuals of foreign origin (numerator) to the total study population (denominator, variable overall)
newcases.rel	Continuous	Values calculated based on abstracted data	The proportion of individuals with incident/ first-time TB disease (numerator) to the total study population (denominator, variable overall)
eptb.rel	Continuous	Values calculated based on abstracted data	The proportion of individuals with extrapulmonary TB (numerator) to the total study population (denominator, variable overall)
ptb.rel	Continuous	Values calculated based on abstracted data	The proportion of individuals with pulmonary TB (numerator) to the total study population (denominator, variable overall)
mixedtb.rel	Continuous	Values calculated based on abstracted data	The proportion of individuals with mixed/multiple-site TB (numerator) to the total study population (denominator, variable overall)
hivaids.rel	Continuous	Values calculated based on abstracted data	The proportion of individuals with HIV/AIDS (numerator) to the total study population (denominator, variable overall)
diabetes.rel	Continuous	Values calculated based on abstracted data	The proportion of individuals with diabetes (numerator) to the total study population (denominator, variable overall)
cancers.rel	Continuous	Values calculated based on abstracted data	The proportion of individuals with malignancies (numerator) to the total study population (denominator, variable overall)
hepcirrh.rel	Continuous	Values calculated based on abstracted data	The proportion of individuals with any form of hepatitis or liver cirrhosis (numerator) to the total study population (denominator, variable overall)
other.rel	Continuous	Values calculated based on abstracted data	The proportion of individuals with any other comorbidity than those mentioned in this list (numerator) to the total study population (denominator, variable overall)

Variable	Type	Categories	Function/definition
anynothiv.rel	Continuous	Values calculated based on abstracted data	The proportion of individuals with any comorbidity except for HIV/AIDS (numerator) to the total study population (denominator, variable overall)
anycm.rel	Continuous	Values calculated based on abstracted data	The proportion of individuals with any comorbidity (numerator) to the total study population (denominator, variable overall)
alcohol.rel	Continuous	Values calculated based on abstracted data	The proportion of individuals with alcohol abuse (numerator) to the total study population (denominator, variable overall)
nohome.rel	Continuous	Values calculated based on abstracted data	The proportion of homeless individuals (numerator) to the total study population (denominator, variable overall)
smoker.rel	Continuous	Values calculated based on abstracted data	The proportion of individuals with smoking habits (numerator) to the total study population (denominator, variable overall)
drugs.rel	Continuous	Values calculated based on abstracted data	The proportion of drug-addicted individuals (numerator) to the total study population (denominator, variable overall)
migrant.rel	Continuous	Values calculated based on abstracted data	The proportion of individuals with migration history (numerator) to the total study population (denominator, variable overall)
formertb.rel	Continuous	Values calculated based on abstracted data	The proportion of individuals with former/previous TB disease (numerator) to the total study population (denominator, variable overall)
prison.rel	Continuous	Values calculated based on abstracted data	The proportion of individuals with imprisonment experiences (numerator) to the total study population (denominator, variable overall)
otherrf.rel	Continuous	Values calculated based on abstracted data	The proportion of individuals with any other risk factor than those mentioned in this list (numerator) to the total study population (denominator, variable overall)
mdr.rel	Continuous	Values calculated based on abstracted data	The proportion of individuals with multi-drug-resistant TB (numerator) to the total study population (denominator, variable overall)
xdr.rel	Continuous	Values calculated based on abstracted data	The proportion of individuals with extensively drug-resistant TB (numerator) to the total study population (denominator, variable overall)
monodr.rel	Continuous	Values calculated based on abstracted data	The proportion of individuals with mono-drug resistant TB (numerator) to the total study population (denominator, variable overall)
lntenew	Continuous	Values calculated based on abstracted data	The recalculation of the variable lnte applying R software due to the error message provided for the variable lnte during the posit cloud sessions

Final meta-regression dataset

study	overall	total.e	total.n	m.e	m.n	m.ne	f.e	f.n	f.ne	mf.e	mf.n	rrmf	rrcilow	rrciup	lnte	sete	var	weights.reml
Abutidze 2012	410	40	110	34	90	56	6	20	14	5.67	4.5	1.26	0.61	2.59	0.231	0.3689	0.135	0.7
Aguado 1997	6326	16	51	10	30	20	6	21	15	1.67	1.43	1.17	0.5	2.71	0.157	0.4311	0.1857	0.5
Balabanova 2016	737	227	737	195	581	386	32	156	124	6.09	3.72	1.64	1.18	2.27	0.495	0.1669	0.0282	1.6
Bartu 2010	50	9	33	9	28	19	0	5	5		5.6	3.67	0.25	54.2	1.3	1.3722	1.8936	0.1
Bastos 2016	681	121	681	96	501	405	25	180	155	3.84	2.78	1.38	0.92	2.07	0.322	0.2069	0.0429	1.3
Baussano 2008	1564	106	1564	77	964	887	29	600	571	2.66	1.61	1.65	1.09	2.5	0.501	0.2118	0.0448	1.3
Bendayan 2011	132	40	132	32	102	70	8	30	22	4	3.4	1.18	0.61	2.27	0.166	0.3352	0.1131	0.8
Bloendal 2013	2449	661	2449	535	1775	1240	126	674	548	4.25	2.63	1.61	1.36	1.92	0.476	0.088	0.0078	2.2
Borgdorff 1998	4981	258	4340	170	2603	2433	88	1737	1649	1.93	1.5	1.29	1	1.66	0.255	0.1293	0.0163	1.9
Cayla 2003	1520	26	1291	20	833	813	6	458	452	3.33	1.82	1.83	0.74	4.53	0.604	0.4622	0.2133	0.5
Crofts 2008	13391	1108	13153	704	7134	6430	404	6019	5615	1.74	1.19	1.47	1.31	1.65	0.385	0.0589	0.0036	2.4
Daucourt 2000	296	40	296	27	183	156	13	113	100	2.08	1.62	1.28	0.69	2.38	0.247	0.3159	0.0996	0.8
Dewan 2004	1069	63	255	55	198	143	8	57	49	6.88	3.47	1.98	1	3.91	0.683	0.3478	0.1206	0.7
Diacon 2014	160	12	132	8	85	77	4	47	43	2	1.81	1.11	0.35	3.48	0.104	0.5859	0.342	0.3
Diel 2003	529	32	518	24	361	337	8	157	149	3	2.3	1.3	0.6	2.84	0.262	0.3966	0.1575	0.6
Dobrotkova 2019	1859	190	1859	138	1254	1116	52	605	553	2.65	2.07	1.28	0.94	1.73	0.247	0.1556	0.024	1.7
Duro 2017	1273	21	39	18	29	11	3	10	7	6	2.9	2.07	0.77	5.56	0.728	0.5043	0.2544	0.4
Erbes 2006	58	14	58	11	46	35	3	12	9	3.67	3.83	0.96	0.32	2.89	−0.041	0.5614	0.3192	0.3
Farah 2005	655	58	655	41	384	343	17	271	254	2.41	1.42	1.7	0.99	2.93	0.531	0.2768	0.0769	1
Faustini 2008	974	55	775	43	516	473	12	259	247	3.58	1.99	1.8	0.97	3.35	0.588	0.3162	0.1008	0.8
Floe 2017	42140	1511	6713	930	3750	2820	581	2963	2382	1.6	1.27	1.26	1.15	1.39	0.231	0.0484	0.2349	2.4
Fortun 2014	814	48	814	37	529	492	11	285	274	3.36	1.86	1.81	0.94	3.5	0.593	0.3354	0.1125	0.8
Franco Spinola 2015	5003	366	1098	297	789	492	69	309	240	4.3	2.55	1.69	1.34	2.11	0.525	0.1158	0.0134	2
Frank 2019	111	16	111	12	77	65	4	34	30	3	2.26	1.32	0.46	3.81	0.278	0.5393	0.2909	0.4
Gadoev 2015	107380	5953	107380	3855	63724	59869	2098	43656	41558	1.84	1.46	1.26	1.2	1.33	0.231	0.0262	0.0007	2.5
GBD Tuberculosis Collaborator 2014 (CE)	37541	2847	37541	2201	25157	22956	646	12384	11738	3.41	2.03	1.68	1.54	1.83	0.519	0.044	0.0019	2.4
GBD Tuberculosis Collaborator 2014 (EE)	188060	21372	188060	17428	133535	116107	3944	54525	50581	4.42	2.45	1.8	1.75	1.87	0.588	0.0169	0.0003	2.5
GBD Tuberculosis Collaborator 2014 (WE)	48702	4671	48702	2660	29517	26857	2011	19185	17174	1.32	1.54	0.86	0.81	0.91	−0.151	0.0297	0.0008	2.5
Girardi 2012	246	36	246	32	199	167	4	47	43	8	4.23	1.89	0.7	5.08	0.637	0.5056	0.2549	0.4
Girardi 2014	5158	208	5158	142	3201	3059	66	1957	1891	2.15	1.64	1.32	0.99	1.75	0.278	0.1453	0.0214	1.8
Haar 2007	13943	91	542	75	400	325	16	142	126	4.69	2.82	1.66	1	2.76	0.507	0.259	0.0663	1.1
Helbling 2002	271	23	265	19	176	157	4	89	85	4.75	1.98	2.4	0.84	6.85	0.875	0.5354	0.2857	0.4
Holden 2020	2131	141	2131	106	1311	1205	35	820	785	3.03	1.6	1.89	1.31	2.75	0.637	0.1892	0.036	1.4
Holmberg 2019	1939	16	53	15	35	20	1	18	17	15	1.94	7.71	1.11	53.83	2.043	0.9902	0.9825	0.1
Khaliaukin 2014	517	154	439	133	367	234	21	72	51	6.33	5.1	1.24	0.85	1.83	0.215	0.1956	0.0385	1.4
Kherosheva 2003	749	31	749	28	574	546	3	175	172	9.33	3.28	2.85	0.88	9.25	1.047	0.6001	0.3616	0.3
Korhonen 2020	1416	231	1416	163	915	752	68	501	433	2.4	1.83	1.31	1.01	1.7	0.27	0.1328	0.0178	1.9
Korzeniewska-Kosela 2017	6430	526	6430	413	4457	4044	113	1973	1860	3.65	2.26	1.62	1.32	1.98	0.482	0.1034	0.0105	2.1
Korzeniewska-Kosela 2018	6444	537	6444	420	4457	4037	117	1987	1870	3.59	2.24	1.6	1.31	1.95	0.47	0.1015	0.0102	2.1
Korzeniewska-Kosela 2019	5787	543	5787	418	4126	3708	125	1661	1536	3.34	2.48	1.35	1.11	1.63	0.3	0.098	0.0095	2.1
Korzeniewska-Kosela 2020	5487	490	5487	383	3900	3517	107	1587	1480	3.58	2.46	1.46	1.19	1.79	0.378	0.1041	0.0111	2.1
Kourbatova 2006	3451	92	460	71	328	257	21	132	111	3.38	2.48	1.36	0.87	2.12	0.307	0.2272	0.0511	1.2
Lanoix 2014	97	21	97	16	77	61	5	20	15	3.2	3.85	0.83	0.35	1.99	−0.186	0.4434	0.1995	0.5
Lockman 2001	92	12	92	10	62	52	2	30	28	5	2.07	2.42	0.57	10.36	0.884	0.7398	0.5505	0.2
Lowe 2013	1236	42	718	29	408	379	13	310	297	2.23	1.32	1.69	0.9	3.21	0.525	0.3244	0.1057	0.8
Loytved 2002	867	22	867	16	538	522	6	329	323	2.67	1.64	1.63	0.64	4.13	0.489	0.4757	0.2243	0.4
Lubart 2007	460	65	460	45	311	266	20	149	129	2.25	2.09	1.08	0.66	1.76	0.077	0.2502	0.0623	1.1
Majoor 2011	16059	46	231	24	105	81	22	126	104	1.09	0.83	1.31	0.78	2.2	0.27	0.2645	0.0697	1

study	**overall**	**total.e**	**total.n**	**m.e**	**m.n**	**m.ne**	**f.e**	**f.n**	**f.ne**	**mf.e**	**mf.n**	**rrmf**	**rrcilow**	**rrciup**	**lnte**	**sete**	**var**	**weights.reml**
Makhmudova 2019	601	89	601	49	342	293	40	259	219	1.23	1.32	0.93	0.63	1.36	−0.073	0.1963	0.0386	1.4
Mathew 2006	1916	183	1916	143	1326	1183	40	590	550	3.58	2.25	1.59	1.14	2.23	0.464	0.1712	0.0295	1.6
Millet 2010	1695	173	762	134	520	386	39	242	203	3.44	2.15	1.6	1.16	2.21	0.47	0.1644	0.027	1.6
Nebreda-Mayoral 2017	75	7	75	6	47	41	1	28	27	6	1.68	3.57	0.45	28.18	1.273	1.0554	1.1097	0.1
Panic 2003	349	33	349	29	264	235	4	85	81	7.25	3.11	2.33	0.85	6.45	0.846	0.517	0.2689	0.4
Pedrazzoli 2019	41	6532	111774	4161	62628	58467	2371	49146	46775	1.75	1.27	1.38	1.31	1.45	0.322	0.0259	0.0006	2.5
Pina 2006	2085	133	2085	98	1352	1254	35	733	698	2.8	1.84	1.52	1.04	2.21	0.419	0.1923	0.0367	1.4
Podlekareva 2014	587	286	587	220	419	199	66	168	102	3.33	2.49	1.34	1.08	1.65	0.293	0.1081	0.0114	2
Podlekareva 2016	1151	236	1151	170	830	660	66	321	255	2.58	2.59	1	0.77	1.28	0	0.1296	0.0167	1.9
Pradipta 2019a	5674	112	5674	72	3426	3354	40	2248	2208	1.8	1.52	1.18	0.81	1.73	0.166	0.1936	0.0382	1.4
Public Health England 2013	8805	79	8751	49	5045	4996	30	3706	3676	1.63	1.36	1.2	0.76	1.89	0.182	0.2324	0.0533	1.2
Public Health England 2014a	8630	432	7892	300	4560	4260	132	3332	3200	2.27	1.37	1.66	1.36	2.03	0.507	0.1022	0.0104	2.1
Public Health England 2015	7257	330	5445	210	3078	2868	120	2367	2247	1.75	1.3	1.35	1.08	1.67	0.3	0.1112	0.0123	2
Public Health England 2016	6472	351	5758	229	3407	3178	122	2351	2229	1.88	1.45	1.3	1.05	1.6	0.262	0.1075	0.0118	2
Public Health England 2020b	4614	181	4614	115	2684	2569	66	1930	1864	1.74	1.39	1.25	0.93	1.69	0.223	0.1524	0.023	1.7
Public Health Ukraine 2017	158507	27108	158507	22240	112390	90150	4868	46117	41249	4.57	2.44	1.87	1.82	1.93	0.626	0.015	0.0002	2.5
RKI 2014	4318	145	4302	97	2665	2568	48	1637	1589	2.02	1.63	1.24	0.88	1.74	0.215	0.1739	0.0302	1.5
RKI 2017	5915	100	5905	65	4000	3935	35	1905	1870	1.86	2.1	0.88	0.59	1.33	−0.128	0.2073	0.0432	1.3
RKI 2019	5429	129	5421	85	3626	3541	44	1795	1751	1.93	2.02	0.96	0.67	1.37	−0.041	0.1825	0.0337	1.5
RKI 2020	4791	129	4782	82	3117	3035	47	1665	1618	1.74	1.87	0.93	0.65	1.33	−0.073	0.1826	0.0326	1.5
Rodriguez-Valin 2015	5880	380	5880	284	3690	3406	96	2190	2094	2.96	1.68	1.76	1.4	2.2	0.565	0.1153	0.0132	2
Shuldiner 2014	4555	325	4537	208	2612	2404	117	1925	1808	1.78	1.36	1.31	1.05	1.63	0.27	0.1122	0.0125	2
Shuldiner 2016	3201	385	3201	238	1789	1551	147	1412	1265	1.62	1.27	1.28	1.05	1.55	0.247	0.0994	0.0097	2.1
Talay 2008	586	14	586	14	463	449	0	123	123		3.76	7.73	0.46	128.63	2.045	1.4371	2.0612	0.1
Valade 2012	824	20	53	14	40	26	6	13	7	2.33	3.08	0.76	0.37	1.56	−0.274	0.3671	0.1362	0.7
Vasankari 2007	629	108	629	73	386	313	35	243	208	2.09	1.59	1.31	0.91	1.9	0.27	0.1878	0.0356	1.5
WHO 2020	235000	23680	235000	17200	155000	137800	6480	80000	73520	2.65	1.94	1.37	1.33	1.41	0.315	0.0149	0.0002	2.5

Final meta-regression dataset (continued)

study	age1524.rel	age2544.rel	age1544.rel	age4564.rel	age65.rel	foreignor.rel	newcases.rel	eptb.rel	ptb.rel	mixedtb.rel	hivaids.rel	diabetes.rel	cancers.rel	hepcirrh.rel
Abutidze 2012			0.18				0.19	0.06	0.21		1	0		0.39
Aguado 1997			0				0.01	0	0.01	0				
Balabanova 2016			0.35	0.27			0.46		0.96		0.03			
Bartu 2010							0.32		0.66					
Bastos 2016											0.17	0.12	0.06	0.13
Baussano 2008						0.43	0.79							
Bendayan 2011							0.42					0.13		0.24
Bloendal 2013						0.43	1		1		0.05			
Borgdorff 1998	0.23	0.37		0.14	0.14		0.87	0.25	0.55	0.07	0.03		0.02	
Cayla 2003					0.15						0.06			
Crofts 2008			0.55	0.2	0.18	0.56	0.72		0.58					
Daucourt 2000			0.02			0.16	0.8	0.38	0.62		0.1			
Dewan 2004			0.06									0	0.01	
Diacon 2014														
Diel 2003						0.4	0.88		0.98		0.06			
Dobrotkova 2019	0.05	0.24		0.4	0.28		0.84							
Duro 2017						0		0	0.02	0.01	0.01	0	0	0
Erbes 2006						0.19			1		0.07	0.14	0.02	0.62
Farah 2005			0.47		0.29	0.61	1		1					
Faustini 2008					0.15	0.29	0.66		0.65	0.02	0.02			
Floe 2017			0.05	0.02	0.03			0.03	0.13					
Fortun 2014							0.84	0.22	0.59	0.18	0.19	0.08		0.16
Franco Spinola 2015			0.09	0.07	0.06		0.2	0.05	0.15		0.03	0.02	0.02	0.01
Frank 2019							0.53	0.05	0.88	0.07	0.03	0.06	0.01	0.17
Gadoev 2015	0.04						0.75	0.23	0.77		0.01			
GBD Tuberculosis Collaborator 2014 (CE)														
GBD Tuberculosis Collaborator 2014 (EE)														
GBD Tuberculosis Collaborator 2014 (WE)														
Girardi 2012			0.66			0.34	0.84	0.2	0.8		1			
Girardi 2014							1	0.07	0.93					
Haar 2007		0.03				0.02		0.01	0.02	0.01	0.04			
Helbling 2002		0.25			0.24	0.6								
Holden 2020					0.06	0.5		0.21	0.73	0.06	0.03	0.05	0.05	
Holmberg 2019						0.01					1			
Khaliaukin 2014			0.42				0.26				0.13			
Kherosheva 2003							0.92	0.04	0.96					
Korhonen 2020						0.27								
Korzeniewska-Kosela 2017							0.9	0.05	0.95					
Korzeniewska-Kosela 2018							0.89	0.05	0.95					
Korzeniewska-Kosela 2019							0.89	0.04	0.96					
Korzeniewska-Kosela 2020							0.88	0.04	0.72					
Kourbatova 2006			0.06			0	0.13	0	0.04	0.01	0.01	0.01	0	
Lanoix 2014											0.41			
Lockman 2001							1		1					
Lowe 2013			0.32			0.51		0.29	0.29		0.08			
Loytved 2002						0.35		0.2	0.8					
Lubart 2007					0.28		0.7	0.16	0.97		0.11	0.1		0.16
Majoor 2011						0.01		0.01	0	0				
Makhmudova 2019							0.27							

study	**age1524.rel**	**age2544.rel**	**age1544.rel**	**age4564.rel**	**age65.rel**	**foreignor.rel**	**newcases.rel**	**eptb.rel**	**ptb.rel**	**mixedtb.rel**	**hivaids.rel**	**diabetes.rel**	**cancers.rel**	**hepcirrh.rel**
Mathew 2006			0.5	0.26			0.86				0	0.02		
Millet 2010						0.04	0.43	0.07	0.34	0.05	0.11			
Nebreda-Mayoral 2017								0.35	0.59			0.07		
Panic 2003														
Pedrazzoli 2019			0.6	0.2	0.15		0.93	0.44	0.56					
Pina 2006			0.54	0.19	0.19			0.16	0.49	0.03	0.06	0.03		
Podlekareva 2014							0.86	0.64			1			0.56
Podlekareva 2016								0.56			1			
Pradipta 2019a	0.15				0.1		0.9		0.07			0.05	0.02	
Public Health England 2013														
Public Health England 2014a							0.89	0.41	0.39					
Public Health England 2015					0.05									
Public Health England 2016														
Public Health England 2020b			0.49	0.23	0.14									
Public Health Ukraine 2017	0.08	0.52		0.32	0.08		0.15	0.02	0.17		0.17			
RKI 2014						0.45	0.77	0.23	0.76					
RKI 2017						0.62	0.59	0.25	0.74					
RKI 2019						0.67	0.66	0.27	0.72					
RKI 2020						0.64	0.65	0.28	0.72					
Rodriguez-Valin 2015			0.49	0.24	0.21	0.32	0.9	0.29	0.71		0.06			
Shuldiner 2014		0.36		0.21	0.27	0.16		0.19	0.81		0.05			
Shuldiner 2016			0.48	0.24	0.27			0.18	0.82		0.04			
Talay 2008			0.31				0.77		1					
Valade 2012								0	0.06					
Vasankari 2007			0.19	0.27	0.54	0.04	0.91		1		0	0.15	0.02	
WHO 2020														

Final meta-regression dataset (continued)

study	other.rel	anynothiv.rel	anycm.rel	alcohol.rel	nohome.rel	smoker.rel	drugs.rel	migrant.rel	formertb.rel	prison.rel	otherrf.rel	mdr.rel	xdr.rel	monodr.rel	lntenew
Abutidze 2012		0.39	0.66	0.17		0.18	0.04		0.08	0.04	0.18	0.03			0.231111721
Aguado 1997															0.1570037488
Balabanova 2016		0.05	0.08	0.57	0.07	0.59			0.54		0.65	0.85			0.4946962418
Bartu 2010												0.66			1.300191662
Bastos 2016	0.35	0.67	0.84	0.23		0.5								0.07	0.3220834992
Baussano 2008					0.04			0.43	0.21						0.5007752879
Bendayan 2011		0.37	0.37	0.26			0.23		0.58				0.08	0.86	0.1655144385
Bloendal 2013			0.05					0.43							0.476234179
Borgdorff 1998		0.02	0.05	0.01	0.01		0.03	0.48	0.09						0.2546422184
Cayla 2003			0.06	0.12	0.02		0.06								0.6043159669
Crofts 2008								0.56	0.07						0.3852624008
Daucourt 2000			0.1		0.04				0.17		0.29	0.01			0.2468600779
Dewan 2004	0.1	0.11	0.11	0.11	0.01					0.03	0.13	0.01		0.13	0.6830968447
Diacon 2014															0.1043600153
Diel 2003			0.06												0.2623642645
Dobrotkova 2019									0.16					0.03	0.2468600779
Duro 2017	0.01	0.01	0.02	0.01	0	0.01	0.01		0		0.01			0	0.7275486073
Erbes 2006	0.31			0.6		0.69			0.26		0.52			0.07	-0.04082199452
Farah 2005															0.5306282511
Faustini 2008			0.02					0.29	0.09						0.5877866649
Floe 2017															0.231111721
Fortun 2014	0.19	0.43	0.62	0.12			0.11	0.23	0.16		0.06	0.04		0.08	0.5933268453
Franco Spinola 2015	0.04	0.08	0.11	0.04			0.02		0.02						0.5247285289
Frank 2019		0.24	0.27	0.32		0.35	0.03		0.36	0.26	0.58				0.2776317366
Gadoev 2015			0.01						0.25	0.01	0.45				0.231111721
GBD Tuberculosis Collaborator 2014 (CE)															0.5187937934
GBD Tuberculosis Collaborator 2014 (EE)															0.5877866649
GBD Tuberculosis Collaborator 2014 (WE)															-0.1508228897
Girardi 2012		0.24	1		0.15		0.48	0.34		0.1					0.6365768291
Girardi 2014															0.2776317366
Haar 2007			0.04				0.01								0.5068176024
Helbling 2002								0.6							0.8754687374
Holden 2020	0.02	0.12	0.15	0.33	0.1	0.54	0.25		0.14	0.02		0.01	0	0.04	0.6365768291
Holmberg 2019			0.03				0.01					0			2.042518188
Khaliaukin 2014			0.13						0.59						0.2151113796
Kherosheva 2003															1.047318994
Korhonen 2020								0.27							0.2700271372
Korzeniewska-Kosela 2017															0.4824261492
Korzeniewska-Kosela 2018															0.4700036292
Korzeniewska-Kosela 2019															0.3001045925
Korzeniewska-Kosela 2020															0.3784364357
Kourbatova 2006	0.03	0.04	0.04	0.07	0.02	0.09	0.01	0		0.03	0.06	0.01		0.06	0.3074846997
Lanoix 2014			0.41												-0.1863295782
Lockman 2001												0.5			0.8837675402
Lowe 2013		0.03	0.12					0.51			0.03				0.5247285289
Loytved 2002															0.4885800148
Lubart 2007	0.49	0.75	0.86	0.11		0.4	0.12		0.3		0.13	0.12	0.03		0.07696104114
Majoor 2011															0.2700271372
Makhmudova 2019															-0.07257069283

study	**other.rel**	**anynothiv.rel**	**anycm.rel**	**alcohol.rel**	**nohome.rel**	**smoker.rel**	**drugs.rel**	**migrant.rel**	**formertb.rel**	**prison.rel**	**otherrf.rel**	**mdr.rel**	**xdr.rel**	**monodr.rel**	lntenew
Mathew 2006		0.02	0.03	0.29			0.03			0.06		0.08			0.4637340162
Millet 2010			0.11	0.12	0.03	0.24	0.08		0.02	0.04		0.01			0.4700036292
Nebreda-Mayoral 2017		0.97	1												1.272565596
Panic 2003				0.03											0.8458682676
Pedrazzoli 2019								0.66	0.07			0.01			0.3220834992
Pina 2006	0.02	0.06	0.12	0.09			0.05	0.03			0.02				0.4187103349
Podlekareva 2014		0.56	1				0.71							0.17	0.292669614
Podlekareva 2016												0.09			0
Pradipta 2019a	0.02	0.09	0.09	0.02	0.02		0.03	0.21		0.03		0.02		0.07	0.1655144385
Public Health England 2013															0.1823215568
Public Health England 2014a															0.5068176024
Public Health England 2015									0.06						0.3001045925
Public Health England 2016									0.06						0.2623642645
Public Health England 2020b															0.2231435513
Public Health Ukraine 2017			0.17												0.6259384309
RKI 2014												0.02			0.2151113796
RKI 2017												0.02			-0.1278333715
RKI 2019															-0.04082199452
RKI 2020												0.02			-0.07257069283
Rodriguez-Valin 2015			0.06					0.32	0.05						0.5653138091
Shuldiner 2014			0.05									0.04			0.2700271372
Shuldiner 2016			0.04					0.82				0.04			0.2468600779
Talay 2008															2.045108863
Valade 2012															-0.2744368457
Vasankari 2007	0.13	0.3	0.3												0.2700271372
WHO 2020															0.3148107398

Final meta-regression dataset (continued)

study	**meanage**	**medianage**
Abutidze 2012		42
Aguado 1997	45,3	
Balabanova 2016		
Bartu 2010	48	
Bastos 2016		47
Baussano 2008		62,7
Bendayan 2011		40
Bloendal 2013		
Borgdorff 1998		
Cayla 2003		
Crofts 2008		
Daucourt 2000		52
Dewan 2004		
Diacon 2014		
Diel 2003	44,2	
Dobrotkova 2019		52
Duro 2017		52
Erbes 2006	47,8	
Farah 2005		
Faustini 2008		
Floe 2017		
Fortun 2014	43,7	
Franco Spinola 2015		
Frank 2019		34
Gadoev 2015		
GBD Tuberculosis Collaborator 2014 (CE)		
GBD Tuberculosis Collaborator 2014 (EE)		
GBD Tuberculosis Collaborator 2014 (WE)		
Girardi 2012		36,7
Girardi 2014	52,1	
Haar 2007		
Helbling 2002		38
Holden 2020		42
Holmberg 2019		38,9
Khaliaukin 2014		45
Kherosheva 2003		
Korhonen 2020		60
Korzeniewska-Kosela 2017	53,7	
Korzeniewska-Kosela 2018	53,2	
Korzeniewska-Kosela 2019	53,9	
Korzeniewska-Kosela 2020	54,2	
Kourbatova 2006	43	
Lanoix 2014	47,4	
Lockman 2001		50
Lowe 2013		
Loytved 2002	49,3	
Lubart 2007	47	
Majoor 2011		
Makhmudova 2019		32

study	**meanage**	**medianage**
Mathew 2006		42
Millet 2010		36
Nebreda-Mayoral 2017	66,2	
Panic 2003	51	
Pedrazzoli 2019		
Pina 2006		
Podlekareva 2014		31
Podlekareva 2016		31
Pradipta 2019a		
Public Health England 2013		
Public Health England 2014a		
Public Health England 2015		
Public Health England 2016		
Public Health England 2020b		
Public Health Ukraine 2017		
RKI 2014		
RKI 2017		
RKI 2019		
RKI 2020		
Rodriguez-Valin 2015		
Shuldiner 2014		
Shuldiner 2016		
Talay 2008	32,5	
Valade 2012		41
Vasankari 2007		
WHO 2020		
