# Supplementary figures and images for: Tuberculosis case fatality is higher in male than female patients in Europe: a systematic review and meta-analysis
Source: Infection. 2024 Mar 23;52(5):1775–86. doi: 10.1007/s15010-024-02206-z (PMC11499538; doi:10.1007/s15010-024-02206-z)

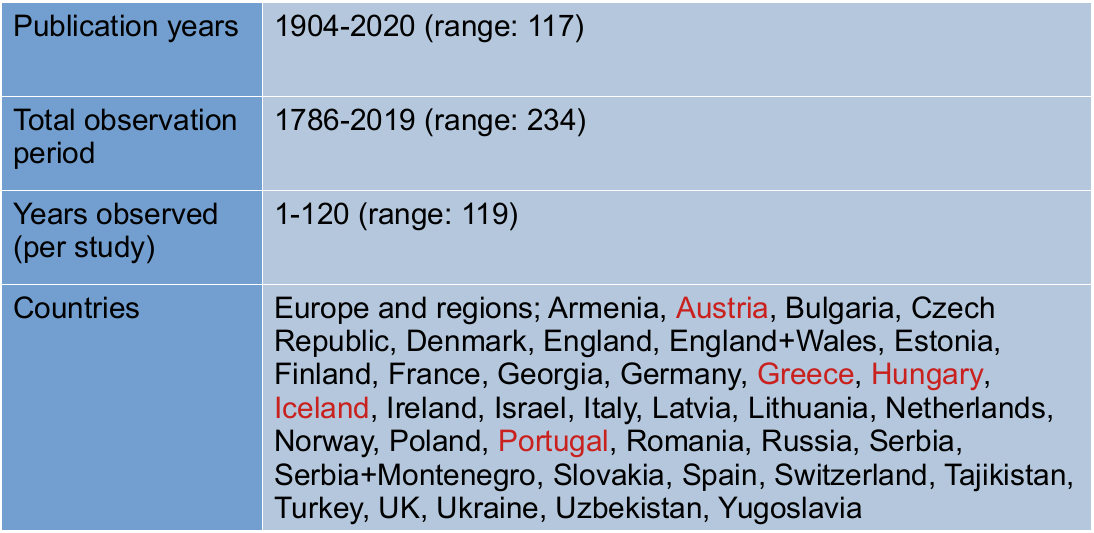

Supplement: Supplementary file 9 — Online Resource 9 Summarized study characteristics of the 263 publications included in the systematic review (PNG 117 KB) [file 15010_2024_2206_MOESM9_ESM.png]

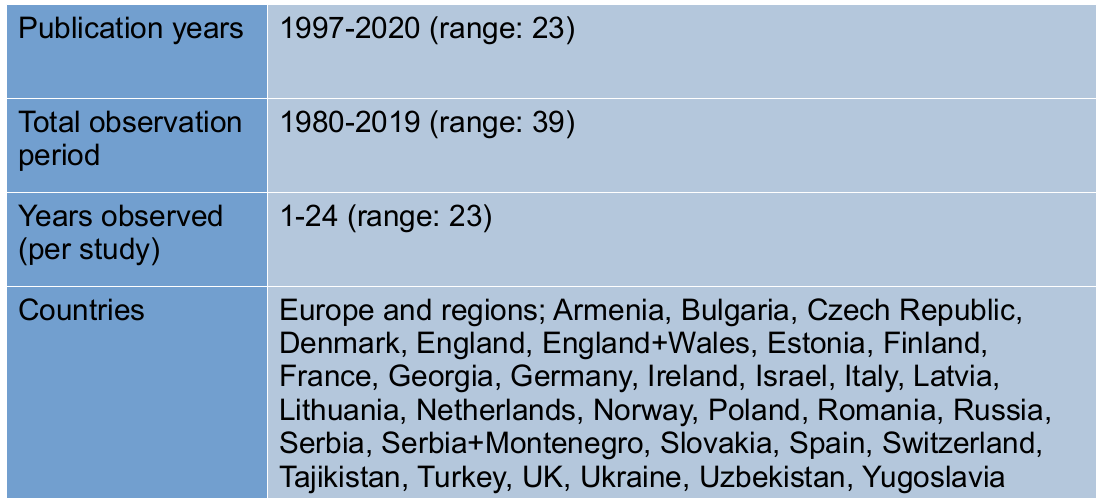

Supplement: Supplementary file 10 — Online Resource 10 Summarized study characteristics of the 135 publications designated for risk of bias assessment (PNG 99 KB) [file 15010_2024_2206_MOESM10_ESM.png]

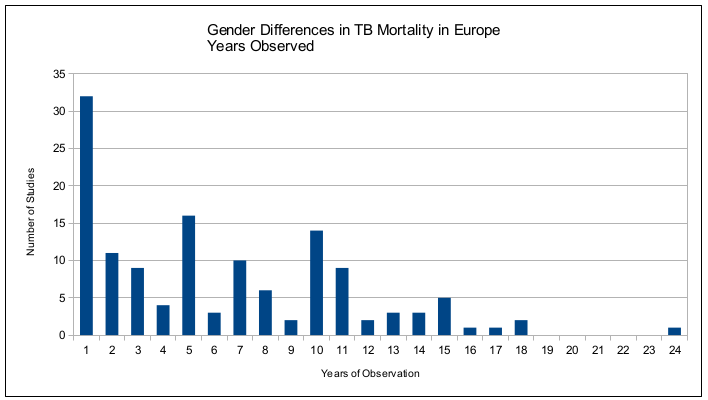

Supplement: Supplementary file 11 — Online Resource 11 Observation periods covered by the publications designated for risk of bias assessment (PNG 19 KB) [file 15010_2024_2206_MOESM11_ESM.png]

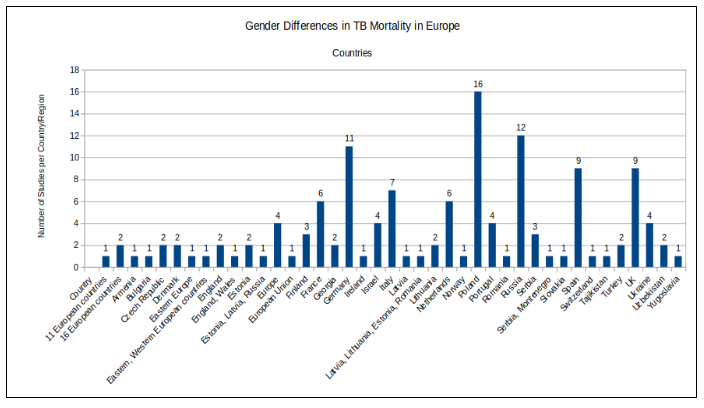

Supplement: Supplementary file 12 — Online Resource 12 Countries included in the risk of bias assessment and the number of publications, stratified by country (PNG 78 KB) [file 15010_2024_2206_MOESM12_ESM.png]

## Effect Estimates Overview Studies Included

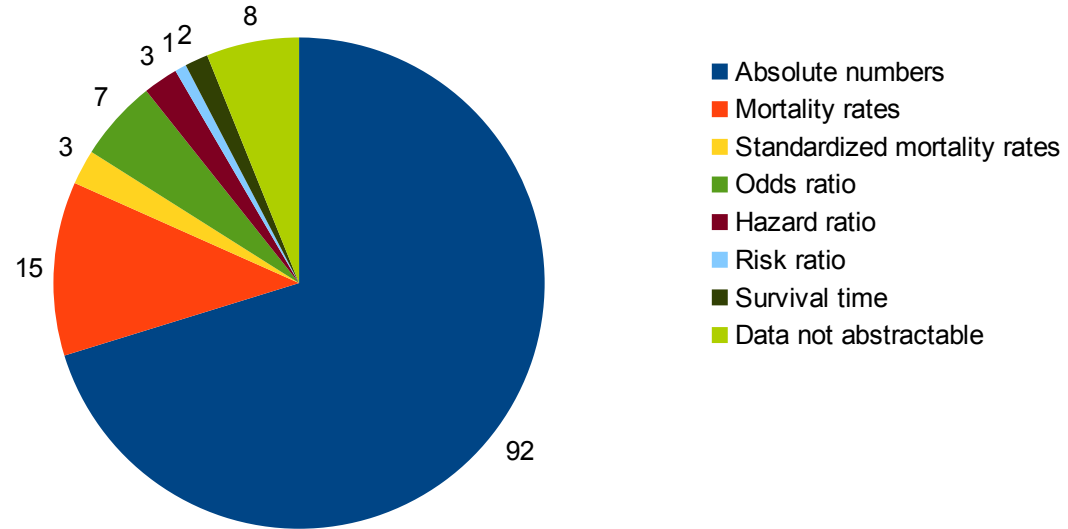

Supplement: Supplementary file 15 — Online Resource 15 Overview of the effect estimates reported by the publications included in quanitative analysis (PDF 26 KB) [file 15010_2024_2206_MOESM15_ESM.pdf]

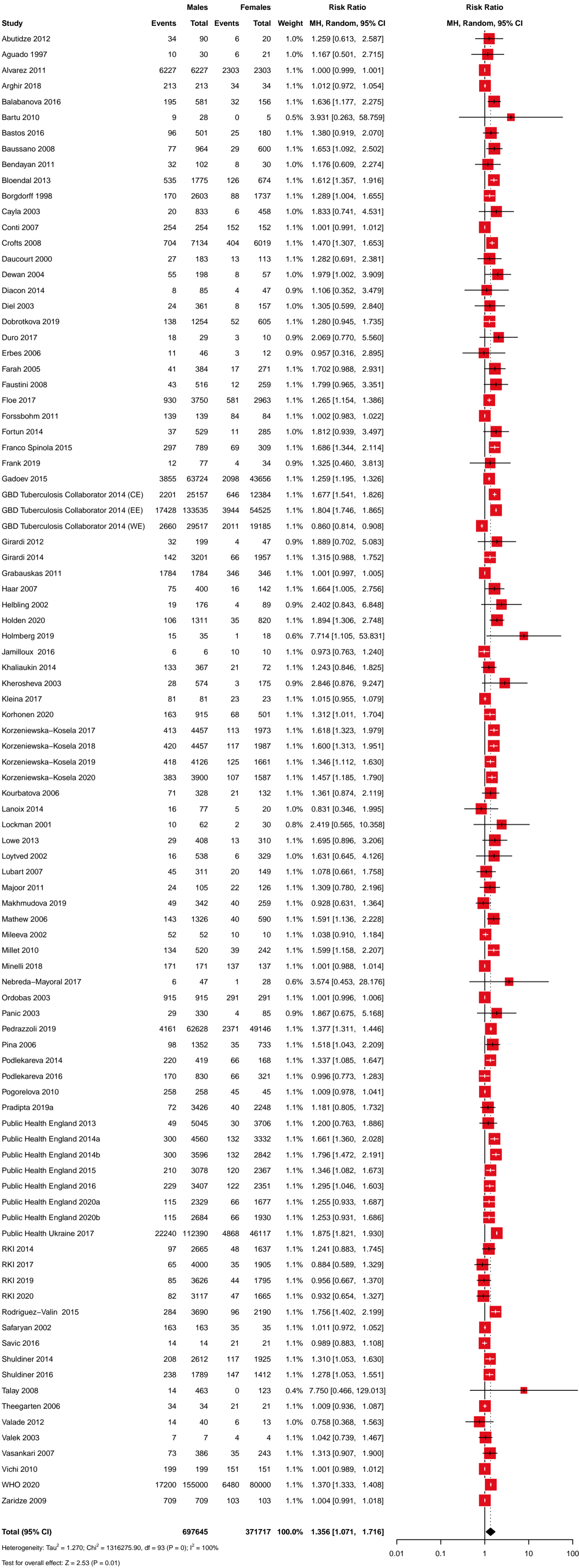

Supplement: Supplementary file 17 — Online Resource 17 Interim meta-analysis results of 94 studies providing absolute numbers for relative risk calculation (PDF 14 KB) [file 15010_2024_2206_MOESM17_ESM.pdf]

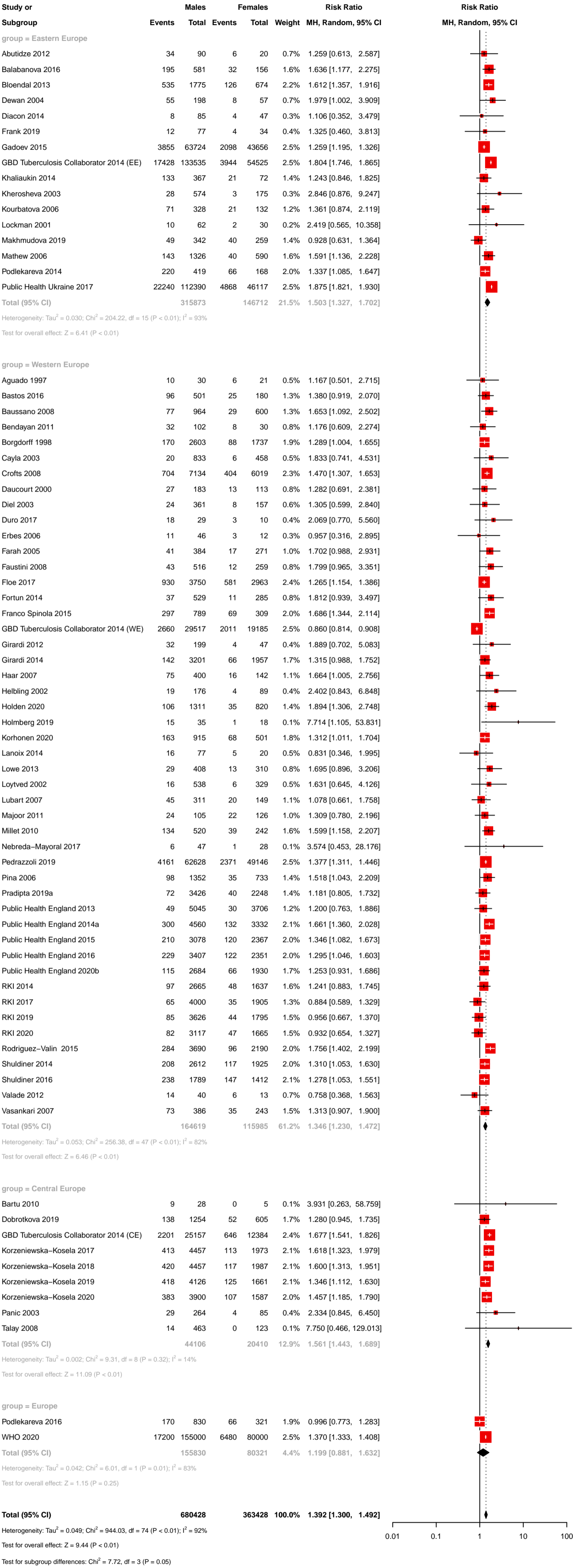

Supplement: Supplementary file 20 — Online Resource 20 Forest plot of publications reporting absolute numbers (n = 75), stratified by regions (PDF 14 KB) [file 15010_2024_2206_MOESM20_ESM.pdf]

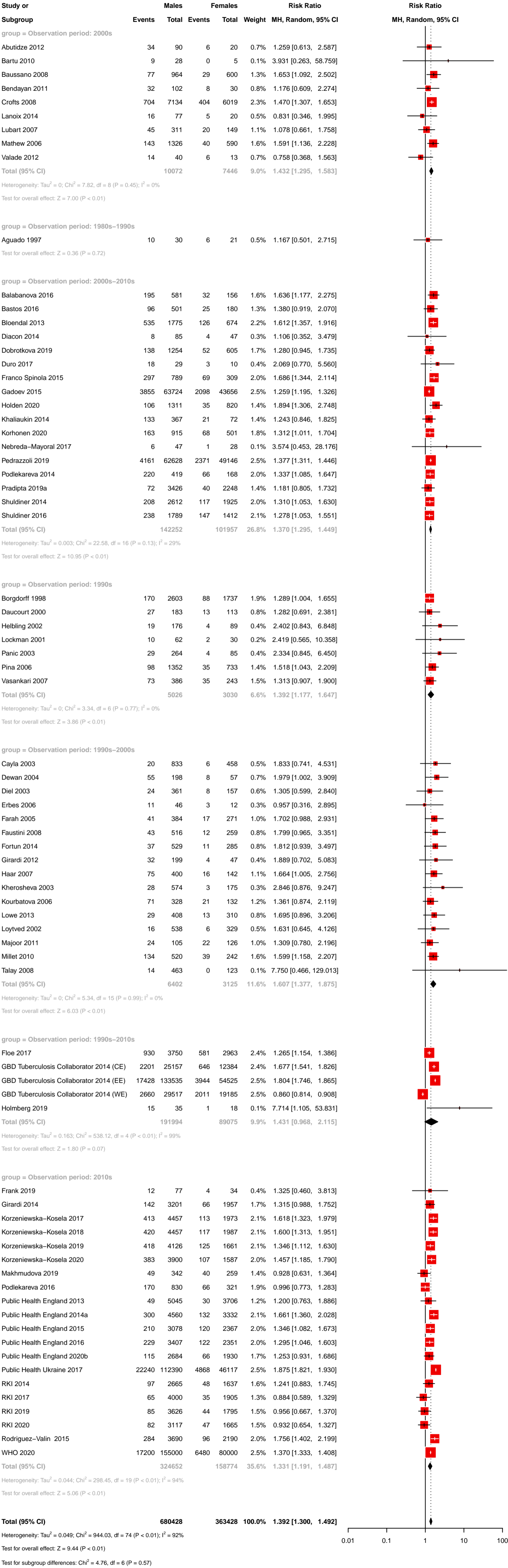

Supplement: Supplementary file 21 — Online Resource 21 Forest plot of publications reporting absolute numbers (n = 75), stratified by observation period (PDF 14 KB) [file 15010_2024_2206_MOESM21_ESM.pdf]

# Bubble plot of moderator homelessness

$$\text{Formula: } y_i = 1.33 + 3.18x_i + u_i + \varepsilon_i$$

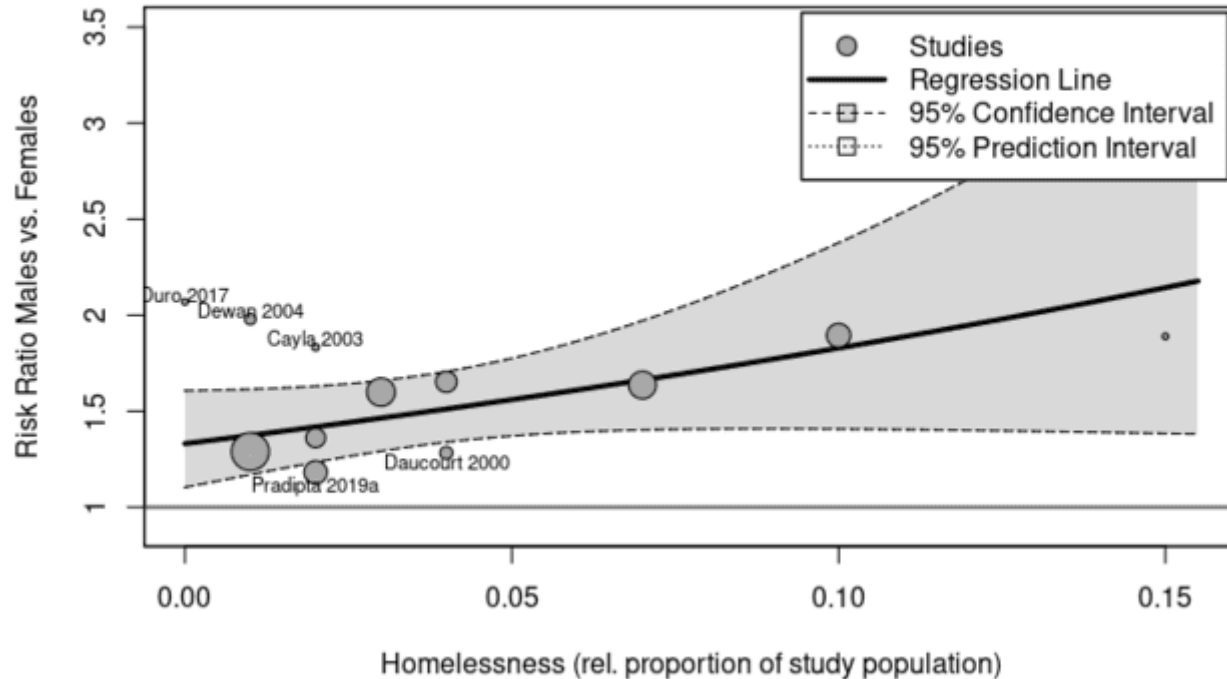

Supplement: Supplementary file 25 — Online Resource 25 Bubble plot of moderator homelessness (univariate meta-regression) (PDF 30 KB) [file 15010_2024_2206_MOESM25_ESM.pdf]

# Bubble plot of moderator migration

$$\text{Formula: } y_i = 1.62 - 0.24x_i + u_i + \varepsilon_i$$

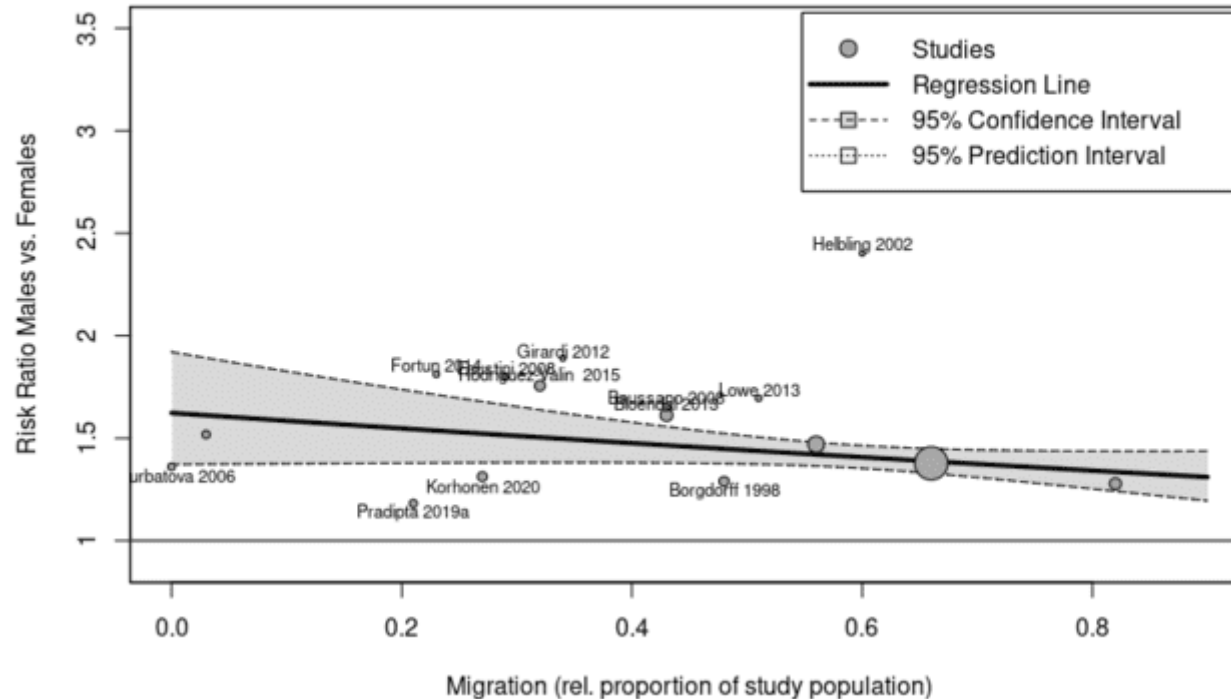

Supplement: Supplementary file 26 — Online Resource 26 Bubble plot of moderator migration (univariate meta-regression) (PDF 30 KB) [file 15010_2024_2206_MOESM26_ESM.pdf]

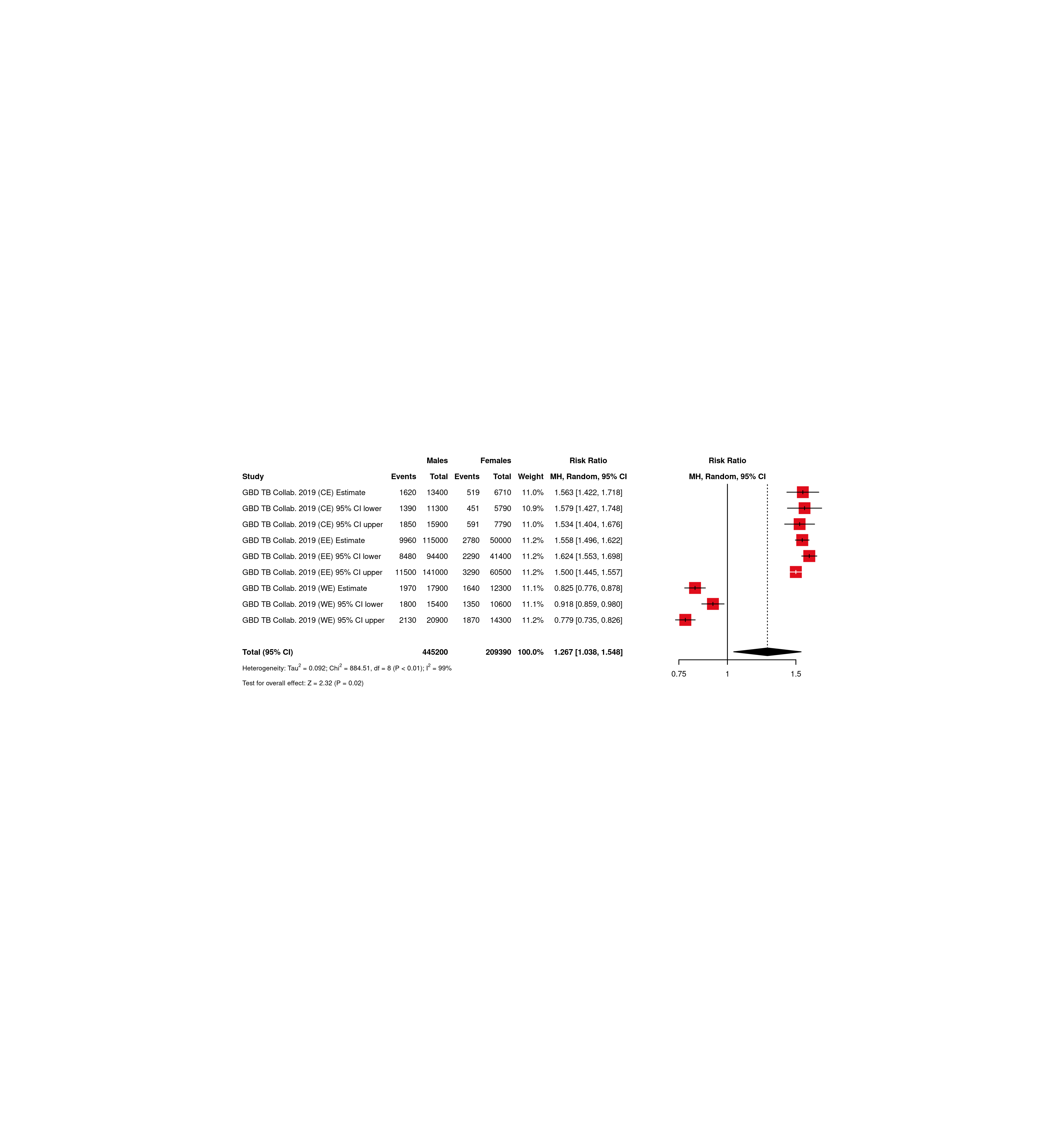

Supplement: Supplementary file 30 — Online Resource 30 Forest plot of the male: female relative risk estimates derived from the Global Burden of Disease 2019 (PNG 229 KB) [file 15010_2024_2206_MOESM30_ESM.png]
