# Supplementary material for: Tuberculosis case fatality is higher in male than female patients in Europe: a systematic review and meta-analysis
Source: Infection. 2024 Mar 23;52(5):1775–86. doi: 10.1007/s15010-024-02206-z (PMC11499538; doi:10.1007/s15010-024-02206-z)
Supplement: Supplementary file 13 — Online Resource 13 Characteristics overview of the 131 studies included in quantitative analyses (PDF 236 KB) [file 15010_2024_2206_MOESM13_ESM.pdf]

Table A- 6: Characteristics overview of the 131 studies included in quantitative analyses

Record No.	Author/ Year	Study Design	Observation period start	Observation period end	Years observed	Country	City/ region	Setting	Study population	Objective	Outcome
167943076	Abutidze 2012 [33]	Cohort	2008	2009	2	Georgia	Tbilisi	Hospital	HIV-infected patients	To determine TB incidence rate among HIV-infected individuals receiving ART and to estimate the independent association between ART use and mortality among HIV-infected individuals.	TB incidence, mortality
167942798	Aguado 1997 [34]	Cohort	1980	1994	15	Spain	Madrid, Barcelona, Valencia, Córdoba	Hospital	Solid-organ transplant recipients	To assess the incidence, clinical features, and response to therapy of M. tuberculosis infection	Incidence, treatment outcome
167943249	Aibana 2018 [35]	Cross-Sectional	2012	2014	2	Ukraine	Kyiv Oblast	Notification data	incident pulmonary TB patients	To evaluate baseline patient factors as predictors of poor DSTB treatment outcomes	Treatment outcome, predictors
167943209	Al-Rahamneh 2017 [19]	Descriptive	2000	2010	11	European Union + Switzerland		Notification data	Men and women > 20 years	To update and analyze TB mortality data for men and women separately; to detect changes in trends and the association with the economic situation and inequalities.	Mortality, Estimated Percentage of Annual Change (EPAC)
167942878	Álvarez 2011 [36]	Descriptive	1990	2003	14	16 European countries		Notification data	Male, female, urban, and rural populations	To describe the magnitude of socioeconomic inequalities in tuberculosis (TB) mortality by levels of education	Mortality, Relative Index of Inequality, slope index of inequality
167942880	Andreychyn 2013 [37]	Cohort	2005	2011	7	Ukraine	Zaporizhzhya	Hospital	HIV/ TB-infected patients	To evaluate the survival and the effect of HAART on mortality	Survival
167943719	Anyama 2007 [38]	Cohort	2000	2003	4	England	East	Notification data	Incident TB cases	To assess treatment outcomes one year after treatment	Treatment outcome, predictors

Record No.	Author/ Year	Study Design	Observation period start	Observation period end	Years observed	Country	City/ region	Setting	Study population	Objective	Outcome
										initiation	
167942892	Arghir 2018 [39]	Cohort	2010	2017	8	Romania	Constanta	Hospital	Dead inpatients from Clinical Pneumophtisiology Hospital	To determine the level of TB-associated mortality in the deaths cohort and to identify risk factors of TB death-related.	Risk factors
167943125	Babalik 2014 [40]	Cohort	2006	2010	5	Turkey	Istanbul	Notification data	Incident TB cases	To investigate the relationship between (i) the diverse quality of life across specific urban regions, (ii) TB incidence rates, inclusive of demographic and clinical characteristics of TB patients, and (iii) adverse treatment outcomes.	Treatment outcome, predictors
167943421	Balabanova 2011 [25]	Cohort	2002	2008	7	Russia	Samara Oblast	TB dispensaries and hospitals	Non-MDRTB cohort, MDRTB civilian and prison patients, civilian XDRTB cohort	To assess the survival and risk factors for death	Survival, risk factors
167943423	Balabanova 2016 [41]	Cohort	2009	2012	3	Latvia, Lithuania, Estonia, Romania	Bucharest City	TB dispensaries and hospitals	Adult new and retreatment patients with culture-confirmed pulmonary MDR-TB	To analyze survival	Survival, risk factors
167942993	Bartu 2010 [27]	Cross-Sectional	2001	2009	9	Czech Republic	Prague	Hospital	Inpatients with MDR-TB of native and foreign origin	To evaluate and compare 50 patients with MDR-TB according to birthplace, resistance type, clinical outcome, length of bacteriological positivity of sputum, and length of hospitalization	Treatment outcome, predictors
167943257	Bastos 2016 [42]	Cohort	2007	2014	8	Portugal	Porto, Vila Nova de Gaia	TB dispensaries and hospitals	Inpatients with pulmonary TB	To develop a severity assessment tool for stratifying mortality risk in pulmonary tuberculosis (PTB) patients	Prognostic factors, clinical scoring system
167942875	Batozhargalovna 2020 [43]	Descriptive	2006	2018	13	Russia		Notification data	Notified HIV and TB-infected cases	To assess the dynamics of mortality rates from Tuberculosis and HIV infection	Mortality, predictors

Record No.	Author/ Year	Study Design	Observation period start	Observation period end	Years observed	Country	City/ region	Setting	Study population	Objective	Outcome
167943256	Baussano 2008 [44]	Cohort	2001	2005	5	Italy	Piemont	Notification data	Register-based cohort of pulmonary tuberculosis (PTB) cases	To identify predictors of unsuccessful PTB treatment and generate a nomogram to assist treating physicians and public health authorities with identifying cases needing close follow-up.	Treatment outcome, predictors
167943235	Bendayan 2011 [45]	Cohort	2000	2005	6	Israel	Zefad, Beer-Yaakov	Hospital	MDR-TB inpatients of national referral TB centers	To outline characteristics and outcomes of hospitalized MDR-TB patients	Treatment outcome, predictors
167943213	Bhering 2019 [46]	Cohort	2000	2014	15	Portugal	Lisbon	Notification data	Notified MDR-TB cases	To identify clinical and demographic factors associated with unfavorable treatment results	Treatment outcome, predictors
167943244	Blöndal 2013 [47]	Cohort	2002	2011	10	Estonia		Notification data	TB and MDR-TB patients	To assess overall and cause-specific mortality	Standardized mortality ratios (SMR), mortality rate ratios (RR)
167943165	Borgdorff 1998 [6]	Cohort	1993	1995	3	Netherlands		Notification data	TB patients	To estimate excess mortality and identify risk factors for TB-associated mortality.	Survival, risk factors
167942836	Brodhun 2015 [48]	Descriptive	2001	2013	13	Germany		Notification data	Notified TB cases	To provide an overview of the TB epidemiology with emphasis on drug resistance and predominantly affected population groups	Incidence, treatment outcome
167943393	Brya 2017 [18]	Cross-Sectional	1999	2012	14	Poland		Notification data	Notified death cases	To evaluate changes in mortality due to TB and years of life lost	Mortality, Crude (CDRs) and standardized (SDRs) death rates, standard expected years of life lost per living person (SEYLLp)
167942861	Caro-Vega 2018 [11]	Cohort	2011	2013	3	Eastern and Western European countries		Hospital	HIV/ TB-infected patients > 15yrs under ART/TB treatment	To assess clinical outcomes (mortality, virological suppression, loss to follow-up during the first year following TB diagnosis) when treated	Mortality, clinical outcome after 12 months, predictors

Record No.	Author/ Year	Study Design	Observation period start	Observation period end	Years observed	Country	City/ region	Setting	Study population	Objective	Outcome
										with efavirenz and non-efavirenz-containing ART regimens.	
167942842	Caylà 2003 [49]	Cohort	1999	2000	1	Spain		Hospital	All TB patients under treatment, diagnosed by a member of the Tuberculosis and Respiratory Infections Group of the Sociedad Española de Neumología y Cirugía Torácica (SEPAR)	To determine treatment completion and to analyze factors associated with treatment default and fatality.	Mortality, treatment outcome, predictors
167943671	Caylà 2009 [50]	Cohort	2006	2009	4	Spain		Hospital	All TB patients under treatment, diagnosed by a member of the Tuberculosis and Respiratory Infections Group of the Sociedad Española de Neumología y Cirugía Torácica (SEPAR)	To identify factors associated with defaulting from or dying during antituberculosis treatment	Mortality, treatment outcome, predictors
167943199	Conti 2007 [51]	Descriptive	2001	2001	1	Italy		Notification data	Entire national population	To assess general mortality and 44 main death causes in the given year	Mortality, standardized rates
167943215	Cox 2007 [12]	Cohort	2003	2005	2	Uzbekistan	Nukus City, Chimbay/ Karakalpak-stan	TB dispensaries and hospitals	MDR-TB cases (first enrolled patient cohort)	To describe the treatment process and outcomes	Treatment outcome
167942952	Crofts 2008 [52]	Descriptive	2001	2002	2	England, Wales		Notification data	Notified TB cases who died	To obtain a more accurate estimate of tuberculosis (TB) case fatality within 12 months of starting treatment or notification	Mortality, capture-recapture estimates
167943004	Daucourt 2000 [53]	Cohort	1995	1996		France	Gironde District	Notification data	TB patients enrolled by the Reseau Tuberculose Gironde (RTG)	To describe the outcome and identify the prognostic factors of success and survival after treatment, emphasizing HIV co-infection.	Treatment outcome, survival, risk factors
167943343	Dewan 2004 [54]	Case-Control	1999	2001	3	Russia	Orel Oblast	TB dispensaries and hospitals	Notified TB cases, incl. TB death cases	To identify risk factors for mortality	Risk factors
779572847	Diacon 2014 [55]	Interventional (Trial)	2008	2012	4	Russia, Latvia, and		Hospital	Patients with newly diagnosed, smear-	To evaluate Bedaquilin treatment outcome in	Time to sputum-culture conversion in liquid

Record No.	Author/ Year	Study Design	Observation period start	Observation period end	Years observed	Country	City/ region	Setting	Study population	Objective	Outcome
						Non-EU countries			positive MDR TB	patients receiving a preferred five-drug background regimen	broth, the rates of culture conversion and drug resistance, pharmacokinetics, and safety over 120 weeks
167943239	Diel 2003 [56]	Cohort	1997	2001	5	Germany	Hamburg	Notification data	New and retreatment patients with culture-confirmed pulmonary TB	To determine risk factors affecting TB treatment outcome	Treatment outcome, predictors
167943062	Dobrotková 2019 [57]	Cross-Sectional	2007	2016	10	Slovakia		Notification data	Notified TB cases	To determine risk factors associated with TB-specific mortality and cure	Mortality, treatment outcome, predictors
167943221	Drobniewski 2002 [58]	Cohort	1996	1998	3	UK		Notification data	MDR TB cases	To describe the clinical, microbiological, and molecular epidemiology and treatment of MDR TB cases in the UK and to determine factors associated with survival.	Survival, risk factors
167943367	Duro 2017 [59]	Cross-Sectional	2007	2014	7	Portugal	Porto	Hospital	TB patients admitted to the ICU of the Infectious Diseases Department of Centro Hospitalar de São João.	To describe the characteristics of TB patients requiring intensive care and to determine the in-hospital mortality and the associated predictive factors.	Mortality, predictors
167942779	Erbes 2006 [60]	Descriptive	1990	2001	12	Germany	Berlin	Hospital	Patients with confirmed pulmonary TB admitted to ICU	To describe severe TB requiring ICU care and to determine the mortality rate and risk factors associated with mortality	Mortality (in-hospital), predictors
167943449	Farah 2005 [61]	Cohort	1996	2002	7	Norway		Notification data	Newly registered cases of culture-positive pulmonary TB	To evaluate the treatment outcome and to identify factors associated with non-successful treatment	Treatment outcome, predictors
167943456	Faustini 2008 [62]	Case-Control	1999	2005	6	Italy	Lazio	Notification data	Notified pulmonary TB cases	To enhance tuberculosis (TB) treatment outcome monitoring by linking diverse surveillance systems and estimating	Treatment outcome, predictors

Record No.	Author/ Year	Study Design	Observation period start	Observation period end	Years observed	Country	City/ region	Setting	Study population	Objective	Outcome
										treatment outcomes, including relapse.	
167942809	Fløe 2017 [30]	Case-Control	1998	2010	13	Denmark		Notification data	Notified TB cases	To evaluate the impact of comorbidities, age, and clinical presentation of TB on mortality among Danish patients with TB	Mortality, comorbidities, predictors
167942844	Forssbohm 2011 [63]	Descriptive	1997	1998	2	Germany		Notification data	Fatal cases classified either as "death from TB" or as "death due to other causes" out of the TB patients in the DZK study	To investigate the validity of the statistics on deaths caused by TB.	Number of fatal cases and death causes
167942988	Fortun 2014 [64]	Cross-Sectional	1997	2008	12	Spain	Madrid	Hospital	EPTB cases diagnosed at Ramon y Cajal Hospital	To identify differential parameters that could predict the presence of EPTB at the initial presentation.	Risk factors
167943499	Franco Spínola 2015 [65]	Case-Control	2008	2012	5	Portugal	Northern Region	Notification data	Notified TB cases with treatment completion	To characterize the factors that relate to mortality among TB patients	Survival, risk factors
167943141	Frank 2019 [66]	Cohort	2011	2013	3	Georgia		Notification data	Notified patients with XDR-TB	To assess the long-term outcomes	Mortality, treatment outcome, predictors
167942996	Gadoev 2015 [67]	Cohort	2006	2010	5	Uzbekistan		Notification data	New and previously treated TB patients registered in the National TB program.	To determine trends in unfavorable outcomes (lost-to-follow-ups, deaths, and treatment failures) and describe their associations with socio-demographic and clinical factors.	Treatment outcome, predictors
167943031	GBD Tuberculosis Collaborator 2014 [22]	Cross-Sectional	1990	2013	14	Central, Eastern, Western Europe, Central Asia (+Mongolia)		Notification data	General population	To estimate mortality using cause-of-death ensemble modeling and to generate consistent trends for corrected case notifications, expert opinions on the case-detection rate, prevalence surveys, and estimated cause-specific mortality using Bayesian meta-regression	Incidence, mortality, predictors

Record No.	Author/ Year	Study Design	Observation period start	Observation period end	Years observed	Country	City/ region	Setting	Study population	Objective	Outcome
167943017	GBD Tuberculosis Collaborator 2017 [16]	Cross-Sectional	2015	2015	1	Europe		Notification data	General population	To estimate tuberculosis mortality rates and disability-adjusted life-years attributable to the independent effects of risk factors, to generate internally consistent estimates of incidence, prevalence, and mortality using DisMod-MR 2.1.	Incidence, prevalence, mortality, DALYs
167943029	GBD Tuberculosis Collaborator 2018 [68]	Cross-Sectional	1990	2016	17	Europe		Notification data	General population	To estimate tuberculosis mortality rates and disability-adjusted life-years attributable to the independent effects of risk factors, to generate internally consistent estimates of incidence, prevalence, and mortality using DisMod-MR 2.1.	Incidence, prevalence, mortality, socio-demographic Index (SDI)
167942774	Girardi 2001 [69]	Cohort	1995	1999	5	Italy	Rome	TB dispensaries and hospitals	HIV-infected patients with culture-confirmed TB in two clinical referral centers	To assess changes in clinical presentation and outcome of HIV-associated TB before and after widespread HAART implementation	Survival, risk factors
775518929	Girardi 2012 [70]	Cohort	1999	2000	2	Italy		Hospital	HIV-infected TB inpatients who started TB treatment	To identify baseline factors associated with treatment outcome, to explain the effect of ART during tuberculosis treatment on mortality	Treatment outcome, predictors
167942935	Girard 2014 [71]	Cross-Sectional	2010	2010	1	France		Notification data	Incident cases of active pulmonary TB	To compare cases reported by the hospital record database (PMSI) and on mandatory notification for better case description	Incidence, mortality, predictors
167942744	Gledovic 2006 [72]	Descriptive	1992	2002	11	Serbia, Montenegro	Central Serbia and Vojvodina	Notification data	Notified TB and death cases	To estimate the TB burden based on incidence, mortality, and DALYs.	Incidence, mortality, DALYs

Record No.	Author/ Year	Study Design	Observation period start	Observation period end	Years observed	Country	City/ region	Setting	Study population	Objective	Outcome
167943466	Grabauskas 2011 [73]	Descriptive	2001	2008	8	Lithuania		Notification data	Notified death cases	To assess the level of avoidable mortality and its changes over time and to define the impact of avoidable mortality on life expectancy.	Mortality, average annual percent change (AAPC)
167943679	Guthmann 2020 [74]	Cohort	2008	2014	7	France		Notification data	Notified non-MDR TB cases	To present surveillance data and identify factors associated with potentially unfavorable treatment outcomes.	Treatment outcome 12 months after treatment initiation, predictors
167943048	Haar 2007 [75]	Cohort	1993	2001	9	Netherlands		Notification data	Notified TB and TB/HIV cases	To study the effect of HIV infection on mortality and the trend in mortality	Mortality, treatment outcome (HAART vs. non), predictors
167943587	Hauer 2011 [76]	Cross-Sectional	2002	2006	5	Germany		Notification data	Notified TB cases	To assess age-related (>/< 60 yrs) differences in TB incidence, mortality, treatment outcome	Incidence, mortality, treatment outcome by age
167943238	Helbling 2002 [28]	Cohort	1996	1996	1	Switzerland		Notification data	Notified TB cases with positive sputum culture	To assess treatment outcomes under current conditions ten months after notification	Treatment outcome, predictors
167942780	Holden 2020 [77]	Cohort	2009	2014	6	Denmark		Notification data	Notified TB cases	To examine and describe predictors associated with TB-related mortality	Mortality, treatment outcome, predictors
167942924	Holmberg 2019 [78]	Cohort	1998	2015	18	Finland	Helsinki	Hospital	HIV cohort of the Helsinki University Hospital	To describe the epidemiology and outcome of HIV-TB co-infections	Incidence, treatment outcome, predictors
167943012	Ilic 2016 [79]	Descriptive	1991	2014	24	Serbia	w/o Kosovo & Metohia	Notification data	Notified death due to infectious diseases from the entire population of the Republic of Serbia (w/o the Autonomous Province of Kosovo & Metohia)	To analyze the trends in mortality from infectious diseases using joinpoint regression analysis.	Mortality, predictors
167943358	Jamilloux 2016 [80]	Descriptive	2002	2011	10	France		Notification data	French decedents with sarcoidosis	To evaluate mortality rates and underlying causes of death	Mortality, predictors
167943413	Khaliaukin 2014 [81]	Cohort	2009	2014	6	Belarus	Gomel Region	Hospital	MDR TB cases from TB health facilities	To determine treatment outcomes among	Treatment outcome, predictors

Record No.	Author/ Year	Study Design	Observation period start	Observation period end	Years observed	Country	City/ region	Setting	Study population	Objective	Outcome
										MDR-TB patients diagnosed in 2009–2010 and factors associated with unsuccessful outcomes (death, failure, and loss to follow-up)	
167942894	Kherosheva 2003 [82]	Descriptive	1999	2000	1	Russia	Orel Oblast	Notification data	TB patients treated in the first year of a TB control demonstration project.	To evaluate treatment outcomes of a revised DOTS strategy	Treatment outcome
167943217	Kleina 2017 [83]	Descriptive	2010	2016	7	Latvia	Riga	Hospital	HIV/AIDS autopsy cases of the Pathology Centre of Riga Eastern Clinical University Hospital	To evaluate the spectrum and morphology of combined lung pathologies in fatal HIV/AIDS cases	Mortality, comorbidities, predictors
167943341	Korhonen 2020 [84]	Cohort	2007	2014	18	Finland		Notification data	Notified microbiologically confirmed pulmonary non-MDR TB cases	To identify potential risk factors for non-successful TB treatment outcomes, with a particular focus on the impact of comorbidities, and to evaluate the treatment outcome monitoring system	Treatment outcome, predictors
167943554	Korzeniewska-Kosela 2010 [85]	Descriptive	2008	2008	1	Poland		Notification data	Notified TB cases	To assess TB epidemiology in the given year	Incidence, treatment outcome, resistance, mortality, predictors
167943546	Korzeniewska-Kosela 2011 [86]	Descriptive	2009	2009	1	Poland		Notification data	Notified TB cases	To assess TB epidemiology in the given year	Incidence, treatment outcome, resistance, mortality, predictors
167943555	Korzeniewska-Kosela 2012 [87]	Descriptive	2010	2010	1	Poland		Notification data	Notified TB cases	To assess TB epidemiology in the given year	Incidence, treatment outcome, resistance, mortality, predictors
167943556	Korzeniewska-Kosela 2013 [88]	Descriptive	2011	2011	1	Poland		Notification data	Notified TB cases	To assess TB epidemiology in the given year	Incidence, treatment outcome, resistance, mortality, predictors
167943557	Korzeniewska-Kosela 2014 [89]	Descriptive	2012	2012	1	Poland		Notification data	Notified TB cases	To assess TB epidemiology in the given year	Incidence, treatment outcome, resistance, mortality, predictors
167943558	Korzeniewska-Kosela 2015 [90]	Descriptive	2013	2013	1	Poland		Notification data	Notified TB cases	To assess TB epidemiology in the given year	Incidence, treatment outcome, resistance, mortality, predictors
167943547	Korzeniewska-	Descriptive	2014	2014	1	Poland		Notification data	Notified TB cases	To assess TB	Incidence, treatment

Record No.	Author/ Year	Study Design	Observation period start	Observation period end	Years observed	Country	City/ region	Setting	Study population	Objective	Outcome
	Kosela 2016 [91]									epidemiology in the given year	outcome, resistance, mortality, predictors
167943559	Korzeniewska-Kosela 2017 [92]	Descriptive	2015	2015	1	Poland		Notification data	Notified TB cases	To assess TB epidemiology in the given year	Incidence, treatment outcome, resistance, mortality, predictors
167943560	Korzeniewska-Kosela 2018 [93]	Descriptive	2016	2016	1	Poland		Notification data	Notified TB cases	To assess TB epidemiology in the given year	Incidence, treatment outcome, resistance, mortality, predictors
167943561	Korzeniewska-Kosela 2019 [94]	Descriptive	2017	2017	1	Poland		Notification data	Notified TB cases	To assess TB epidemiology in the given year	Incidence, treatment outcome, resistance, mortality, predictors
167943562	Korzeniewska-Kosela 2020 [95]	Descriptive	2018	2018	1	Poland		Notification data	Notified TB cases	To assess TB epidemiology in the given year	Incidence, treatment outcome, resistance, mortality, predictors
167943344	Kourbatova 2006 [7]	Case-Control	1999	2003	5	Russia	Samara Oblast	TB dispensaries and hospitals	Notified adult patients with newly diagnosed TB	To identify predictors of all-cause mortality within 12 months after diagnosis	Survival, risk factors
167943264	Kurbatova 2012 [14]	Cohort	2000	2004	5	Estonia, Latvia, Russia		TB dispensaries and hospitals	MDR-TB patients treated with second-line drugs in DOTS-plus projects	To identify predictors of death, failure, and default	Treatment outcome, predictors
167943594	Lanoix 2014 [96]	Cross-Sectional	2000	2009	10	France	Paris	Notification data	ICU patients with active TB at the Bichat-Claude Bernard Hospital	To assess the risk factors for mortality due to TB in a low-burden setting	Survival, risk factors
167942797	Lockman 2001 [97]	Case-Control	1994	1997	4	Estonia		Notification data	Notified cases with pan-sensitive or MDR TB	To compare clinical outcomes	Treatment outcome, predictors
167943224	Lowe 2013 [98]	Cross-Sectional	1999	2006	8	UK	London	Hospital	TB patients of the Newham University Hospital Trust and King's College Hospital	To analyze the overall prognostic significance of neutrophilia in human TB	Mortality, predictors
775029822	Loytved 2002 [99]	Cross-Sectional	1995	2001	7	Germany	Lower Franconia	Notification data	Notified TB cases	To analyze active case-finding effectiveness, treatment outcome, and risk factors for poor outcome	Incidence, treatment outcome, predictors
167943052	Lubart 2007 [100]	Cross-Sectional	2000	2004	5	Israel	Tel Aviv	Hospital	Active pulmonary TB inpatients of the pulmonary diseases department of Shmuel Harofe Hospital	To determine the mortality rate and predictors of mortality	Mortality, predictors
167942933	Majoor 2011	Descriptive	1993	2007	15	Netherlands		Notification data	Notified M. bovis cases	To investigate the	Incidence, treatment

Record No.	Author/ Year	Study Design	Observation period start	Observation period end	Years observed	Country	City/ region	Setting	Study population	Objective	Outcome
	[101]									magnitude of M. bovis infection	outcome, predictors
167943348	Makhmudova 2019 [102]	Cross-Sectional	2012	2013	2	Tajikistan		Hospital	Rifampicin-resistant (RR)/MDR-TB patients enrolled for second-line drug treatment	To investigate the risk factors for unfavorable treatment outcomes.	Treatment outcome, predictors
167942760	Mathew 2006 [103]	Descriptive	2002	2003	2	Russia	Tomsk Oblast/ Siberia	Notification data	TB patients under treatment	To identify risk factors and causes of death	Treatment outcome, predictors
167942994	Milanov 2015 [104]	Cohort	2009	2010	2	Bulgaria	Gabrovo	TB dispensaries and hospitals	MDR TB cases under treatment	To analyze determinants of success and death	Treatment outcome, predictors
167943152	Mileeva 2002 [105]	Descriptive	1991	2000	10	Russia	Kirov Region	TB dispensaries and hospitals	TB patients died in the hospital of the Kirov Regional TB dispensary	To analyze the sociomedical structure and causes of death among new cases and registered cases	Mortality (in-hospital), predictors
167943262	Millet 2010 [106]	Cohort	1995	2005	11	Spain	Barcelona	Notification data	Cohort of successfully treated TB patients	To identify the probability of death and its predictive factors	Mortality, predictors
167943201	Minelli 2018 [107]	Descriptive	2015	2015	1	Italy		Notification data	Entire national population	To assess general mortality and 35 main death causes in the given year	Mortality, standardized rates
167943050	Nebreda-Mayoral 2017 [108]	Cross-Sectional	2006	2015	10	Spain	Castile, Leon	Hospital	Notified M. bovis cases	To determine the patients' epidemiological, clinical, and microbiological characteristics	Epidemiology, clinical course PTB vs. EPTB, predictors
167943418	Olurotimi Bankole 2014 [109]	Cohort	2008	2012	5	Ireland	Cork City	Hospital	Bacteriologically confirmed TB patients from two teaching hospitals	To estimate survival time (ST) and to assess the association and impact of TB risk factors on event status and ST	Survival, risk factors
167943170	Ordobás 2003 [110]	Descriptive	1991	1998	8	Spain	Madrid	Notification data	Notified death cases	To describe TB as a cause of death in the region	Mortality, predictors
167943385	Panic 2003 [111]	Descriptive	1990	1999	10	Yugoslavia	North-West	Hospital	Notified PTB cases from war-affected areas (WAA) and the general population (GP)	To describe differences between WAA and GP groups, to evaluate the portion and characteristics of WAA patients in PTB hospital mortality rate,	Epidemiology, mortality (in-hospital), predictors, "stigma."

Record No.	Author/ Year	Study Design	Observation period start	Observation period end	Years observed	Country	City/ region	Setting	Study population	Objective	Outcome
										and to evaluate TB as "stigma."	
167943465	Pedrazzoli 2019 [112]	Cohort	2001	2014	15	UK		Notification data	Notified TB cases	To describe trends in deaths among notified TB patients, explore risk factors associated with death and compare all-cause mortality in TB patients with age-specific mortality rates in the general UK population.	Mortality, predictors
775518931	Pina 2006 [113]	Cross-Sectional	1996	1997	1	Spain	Catalonia	Notification data	Notified TB cases	To calculate excess mortality and study the factors associated with death.	Mortality, treatment outcome, predictors
167943371	Podlekareva 2014 [114]	Cohort	2004	2010	7	Eastern Europe (11 European countries)		Hospital	HIV/ TB-infected patients enrolled in the TB: HIV study.	To assess and compare mortality rates and causes of death	Mortality, predictors
167943687	Podlekareva 2016 [115]	Cohort	2011	2013	3	16 European countries		Hospital	HIV/ TB-infected patients enrolled in the TB: HIV study.	To report 1-year mortality estimates	Mortality, predictors
167943230	Pogorelova 2010 [116]	Descriptive	2006	2006	1	Russia	Orloskaya, Tulskaya Oblast	TB dispensaries and hospitals	Notified TB death cases	To assess the validity of TB mortality indicators	Mortality, indicator validity
167943261	Pradipta 2019a [117]	Cohort	2005	2015	11	Netherlands		Notification data	Notified native- and foreign-born patients with drug-susceptible TB	To evaluate treatment outcomes and predictors for poor treatment outcomes	Treatment outcome, predictors
167943459	Pradipta 2019b [17]	Cohort	2005	2015	11	Netherlands		Notification data	Notified treated adult patients with confirmed DR-TB	To analyze patient risk factors associated with the incidence of poor outcomes of TB treatment	Treatment outcome, predictors
167943608	Public Health England 2013 [118]	Descriptive	2011	2011	1	UK		Notification data	Notified TB cases	To assess TB epidemiology in the given year	Incidence, treatment outcome, resistance, mortality, predictors
167943605	Public Health England 2014a [119]	Descriptive	2012	2012	1	UK		Notification data	Notified TB cases	To assess TB epidemiology in the given year	Incidence, treatment outcome, resistance, mortality, predictors
167943430	Public Health England 2014b [119]	Descriptive	2012	2012	1	England		Notification data	Notified TB cases	To assess TB epidemiology in the given year	Incidence, treatment outcome, resistance, mortality, predictors

Record No.	Author/ Year	Study Design	Observation period start	Observation period end	Years observed	Country	City/ region	Setting	Study population	Objective	Outcome
167943513	Public Health England 2015 [120]	Descriptive	2013	2013	1	England		Notification data	Notified TB cases	To assess TB epidemiology in the given year	Incidence, treatment outcome, resistance, mortality, predictors
167943514	Public Health England 2016 [121]	Descriptive	2014	2014	1	England		Notification data	Notified TB cases	To assess TB epidemiology in the given year	Incidence, treatment outcome, resistance, mortality, predictors
167943703	Public Health England 2020a [122]	Descriptive	2009	2018	10	England		Notification data	Notified TB cases	To assess TB epidemiology in the given year	Incidence, treatment outcome, resistance, mortality, predictors
167943515	Public Health England 2020b [123]	Descriptive	2018	2018	1	England		Notification data	Notified TB cases	To assess TB epidemiology in the given year	Incidence, treatment outcome, resistance, mortality, predictors
167943615	Public Health Ukraine 2017 [23]	Descriptive	2012	2016	5	Ukraine		Notification data	Notified TB cases	To assess TB epidemiology in the given year	Incidence, treatment outcome, resistance, mortality, predictors
167943351	RKI 2014 [124]	Descriptive	2013	2013	1	Germany		Notification data	Notified TB cases	To assess TB epidemiology in the given year	Incidence, treatment outcome, resistance, mortality, predictors
167943352	RKI 2017 [125]	Descriptive	2016	2016	1	Germany		Notification data	Notified TB cases	To assess TB epidemiology in the given year	Incidence, treatment outcome, resistance, mortality, predictors
167943353	RKI 2019 [126]	Descriptive	2018	2018	1	Germany		Notification data	Notified TB cases	To assess TB epidemiology in the given year	Incidence, treatment outcome, resistance, mortality, predictors
167943350	RKI 2020 [127]	Descriptive	2019	2019	1	Germany		Notification data	Notified TB cases	To assess TB epidemiology in the given year	Incidence, treatment outcome, resistance, mortality, predictors
167943347	Rodríguez-Valín 2015 [128]	Cross-Sectional	2012	2012	1	Spain		Notification data	Notified TB cases	To determine the risk factors for unsuccessful completion of treatment and deaths of TB cases	Treatment outcome, predictors
167942714	Safaryan 2002 [129]	Descriptive	1996	2000	5	Armenia		TB dispensaries and hospitals	Notified TB death cases	To analyze TB mortality and the underlying causes of death	Mortality, predictors
167942801	Savic 2016 [130]	Descriptive	2008	2014	7	Serbia	Belgrad	Hospital	Autopsy cases at the Institute of Pathology	To study the frequency of miliary TB (MT) diagnosed at autopsy and determine clinical diagnoses that masked TB, causes of death, and comorbidities	Clinically unrecognized MT predictors
167942771	Shkolnikov 2001 [20]	Descriptive	1991	1998	8	Russia		Notification data	Notified death cases	To analyze the changes in life expectancy	Mortality, predictors

Record No.	Author/ Year	Study Design	Observation period start	Observation period end	Years observed	Country	City/ region	Setting	Study population	Objective	Outcome
167942821	Shkolnikov 2013 [131]	Cross-Sectional	2004	2010	7	Russia, UK		Notification data	Notified death cases	To determine the causes and age groups that account for the additional years of life gained in 2004-10 and the remaining gap between Russia and Western countries, to assess to what extent these recent trends represent a new development relative to previous mortality fluctuations, and to identify possible explanations for the improvement	Mortality, predictors
167943208	Shuldiner 2014 [132]	Cohort	2000	2010	11	Israel		TB dispensaries and hospitals	Notified TB cases	To assess TB case fatality rate (CFR) and mortality trends during treatment and to identify risk factors associated with mortality.	Mortality, treatment outcome, predictors
167943162	Shuldiner 2016 [133]	Cohort	2000	2010	11	Israel		Notification data	Notified TB cases who recovered	To assess long-term mortality and to compare the mortality rate and causes of death with those of the general population.	Mortality, predictors
167943471	Stosic 2020 [26]	Cross-Sectional	2005	2015	11	Serbia		Notification data	Notified TB cases	To analyze the trends in notification and treatment success rates and identify predictors of treatment outcomes.	Incidence, treatment outcome, mortality, predictors
167943550	Szczuka 2006 [134]	Descriptive	2004	2004	1	Poland		Notification data	Notified TB cases	To assess TB epidemiology in the given year	Incidence, treatment outcome, resistance, mortality, predictors
167943551	Szczuka 2007 [135]	Descriptive	2005	2005	1	Poland		Notification data	Notified TB cases	To assess TB epidemiology in the given year	Incidence, treatment outcome, resistance, mortality, predictors
167943552	Szczuka 2008 [136]	Descriptive	2006	2006	1	Poland		Notification data	Notified TB cases	To assess TB epidemiology in the given year	Incidence, treatment outcome, resistance, mortality, predictors
167943553	Szczuka 2009 [137]	Descriptive	2007	2007	1	Poland		Notification data	Notified TB cases	To assess TB epidemiology in the given year	Incidence, treatment outcome, resistance, mortality, predictors

Record No.	Author/ Year	Study Design	Observation period start	Observation period end	Years observed	Country	City/ region	Setting	Study population	Objective	Outcome
167942995	Talay 2008 [29]	Cohort	1999	2004	5	Turkey	Istanbul	TB dispensaries and hospitals	Adult pulmonary TB patients (> 15 years) documented in the Istanbul Eyup Tuberculosis Dispensary.	To evaluate the treatment outcome of pulmonary TB patients and factors affecting treatment outcomes.	Treatment outcome, predictors
167943008	Theegarten 2006 [138]	Cross-Sectional	1990	2004	15	Germany	Bochum	Hospital	Autopsy cases at the Institute of Pathology	To investigate the frequency of TB in autopsies	Incidence
167943596	Valade 2012 [139]	Cross-Sectional	2000	2009	10	France	Paris	Hospital	TB patients admitted to the ICU of the Hôpitaux de Paris, Hôpital Lariboisière.	To determine prognostic factors of death in TB patients admitted to the ICU and to develop a simple predictive scoring system.	Mortality, predictors, predictive fatality score
167943543	Valek 2003 [140]	Case Series	1991	2000	10	Czech Republic	Prague	Hospital	End-stage renal disease patients undergoing dialysis at the Department of Medicine Strahov, General University Hospital, incl. Death and autopsy cases	To analyze the incidence of TB	Incidence, epidemiology, predictors
167943346	Vasankari 2007 [141]	Cross-Sectional	1995	1996	2	Finland		Hospital	Treated culture-verified pulmonary TB cases	To investigate the patient- and treatment-system-dependent factors affecting treatment outcomes to establish a basis for improving outcomes.	Treatment outcome, predictors
167943200	Vichi 2010 [142]	Descriptive	2010	2010	1	Italy		Notification data	Entire national population	To assess general mortality and 35 main death causes in the given year	Mortality, standardized rates
167943294	WHO 2020 [143]	Descriptive	2019	2019	1	Europe		Notification data	Notified TB cases	To provide a comprehensive and up-to-date assessment of the TB epidemic's status and progress in response to the epidemic – at global, regional, and country levels.	Incidence, treatment outcome, resistance, mortality, predictors
167942699	Zaridze 2009 [144]	Case-Control	1990	2005	16	Russia	Tomsk, Barnaul, Biysk	Notification data	Addresses of residents who had died at ages 15–74 years in 1990–2001	To investigate the effects of alcohol consumption on male and female cause-	Mortality, predictors

Record No.	Author/ Year	Study Design	Observation period start	Observation period end	Years observed	Country	City/ region	Setting	Study population	Objective	Outcome
										specific mortality	
