# Supplementary material for: Tuberculosis case fatality is higher in male than female patients in Europe: a systematic review and meta-analysis
Source: Infection. 2024 Mar 23;52(5):1775–86. doi: 10.1007/s15010-024-02206-z (PMC11499538; doi:10.1007/s15010-024-02206-z)
Supplement: Supplementary file 14 — Online Resource 14 Quality assessment results, stratified by study type (PDF 759 KB) [file 15010_2024_2206_MOESM14_ESM.pdf]

Quality assessment results

I. Case-control studies

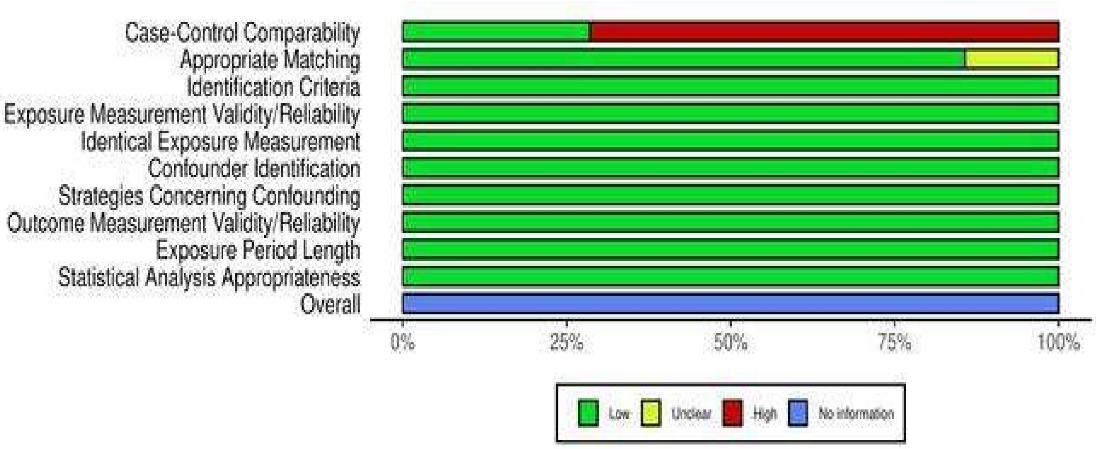

Figure A- 1: Case-control studies - Summary of quality assessment results

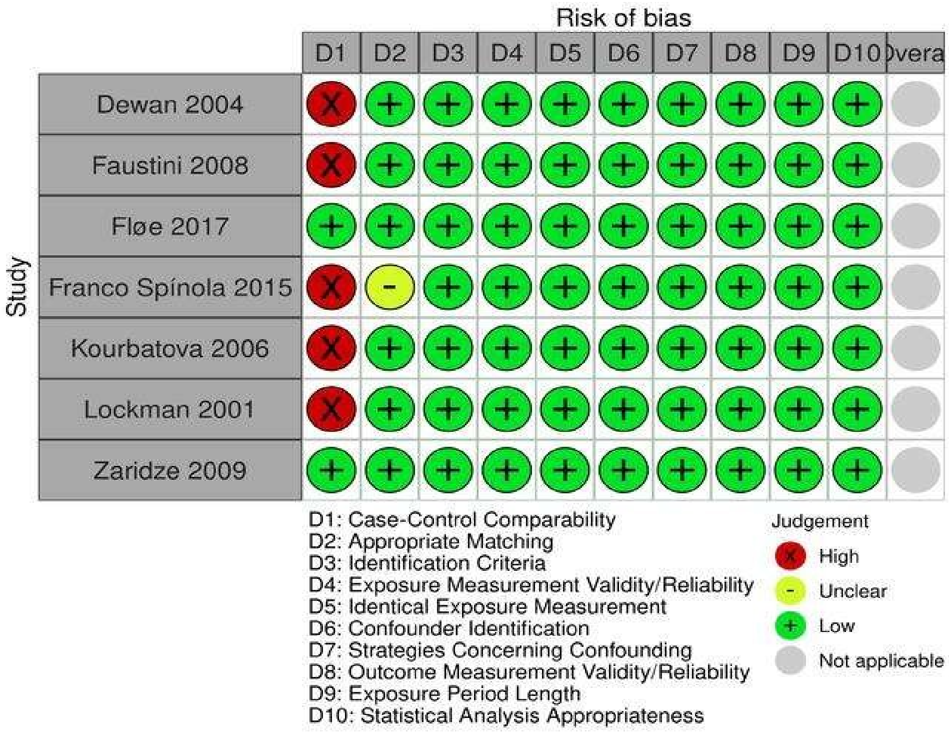

Figure A- 2: Case-control studies - Traffic light panel of quality assessment results

II. Case series

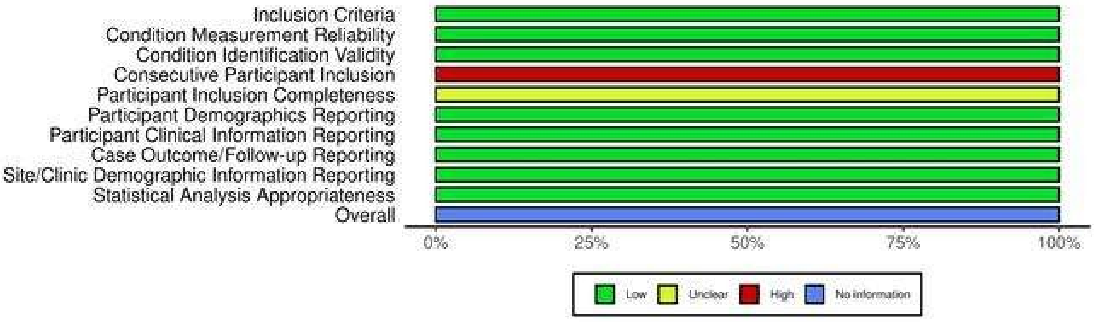

Figure A- 3: Case series - Summary of quality assessment results

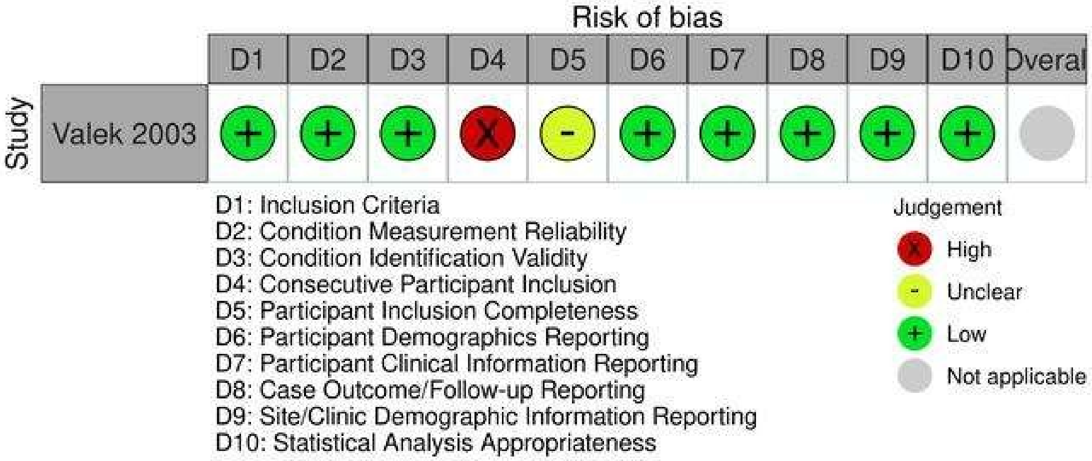

Figure A- 4: Case series - Traffic light panel of quality assessment results

III. Cohort studies

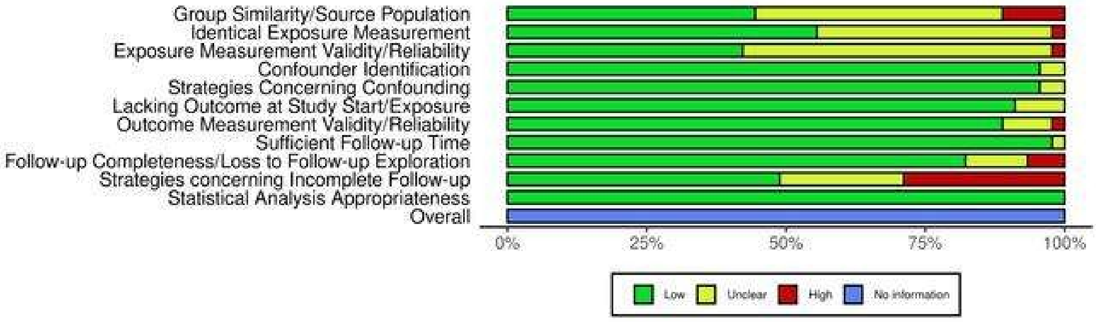

Figure A- 5: Cohort studies - Summary of quality assessment results

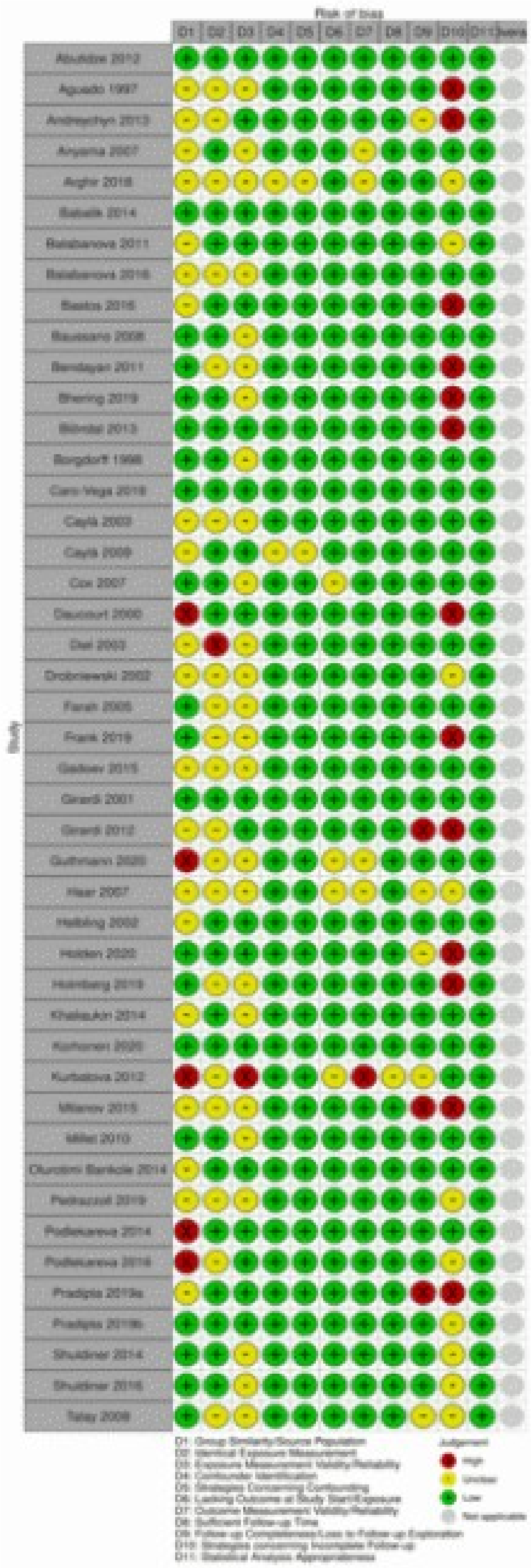

Figure A- 6: Cohort studies - Traffic light panel of quality assessment results

IV. Cross-sectional studies

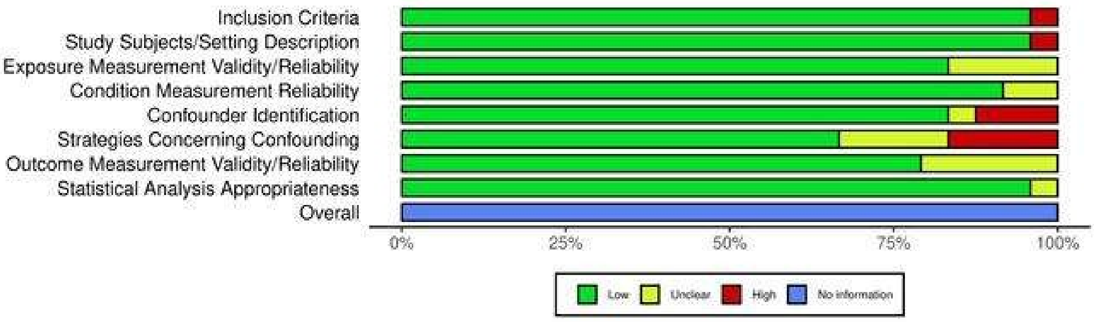

Figure A- 7: Cross-sectional studies - Summary of quality assessment results

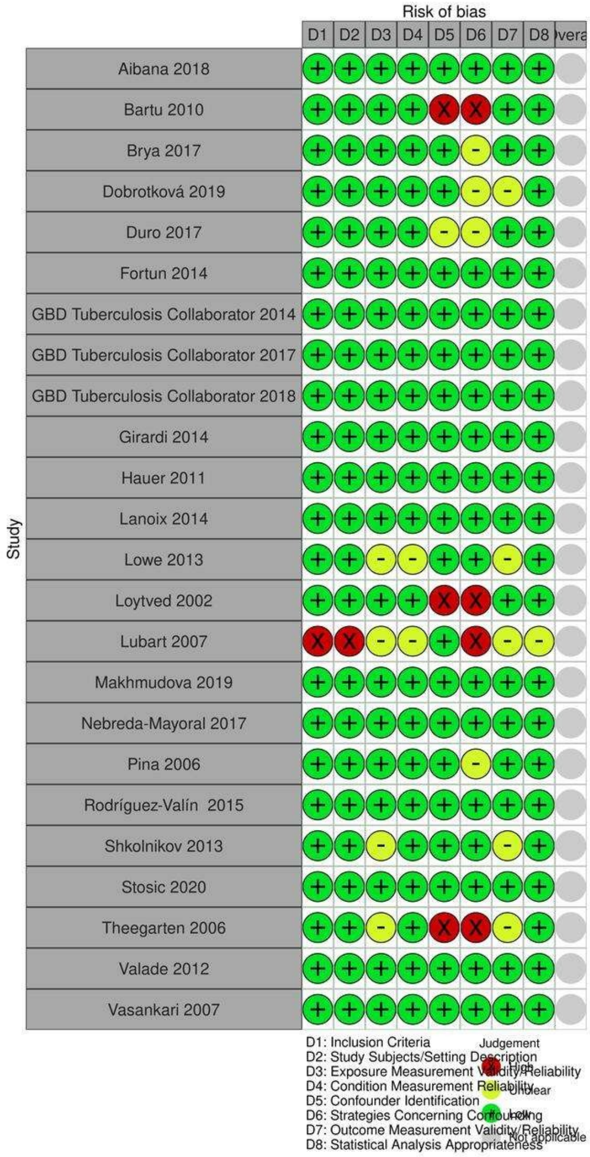

Figure A- 8: Cross-sectional studies - Traffic light panel of quality assessment results

V. Descriptive studies

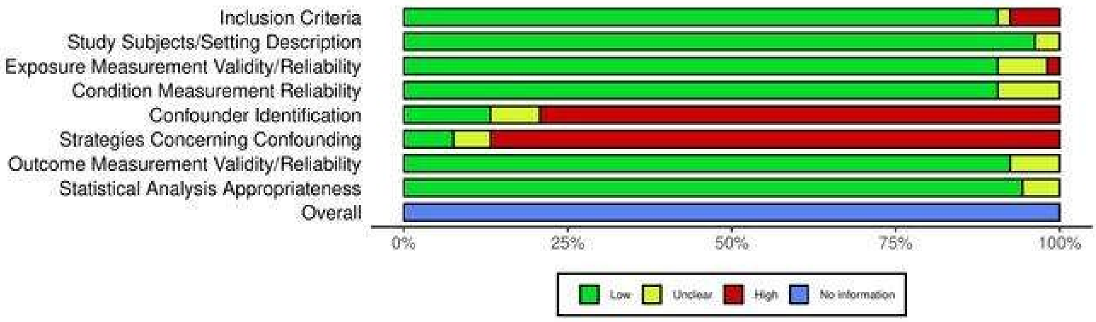

Figure A- 9: Descriptive studies - Summary of quality assessment results

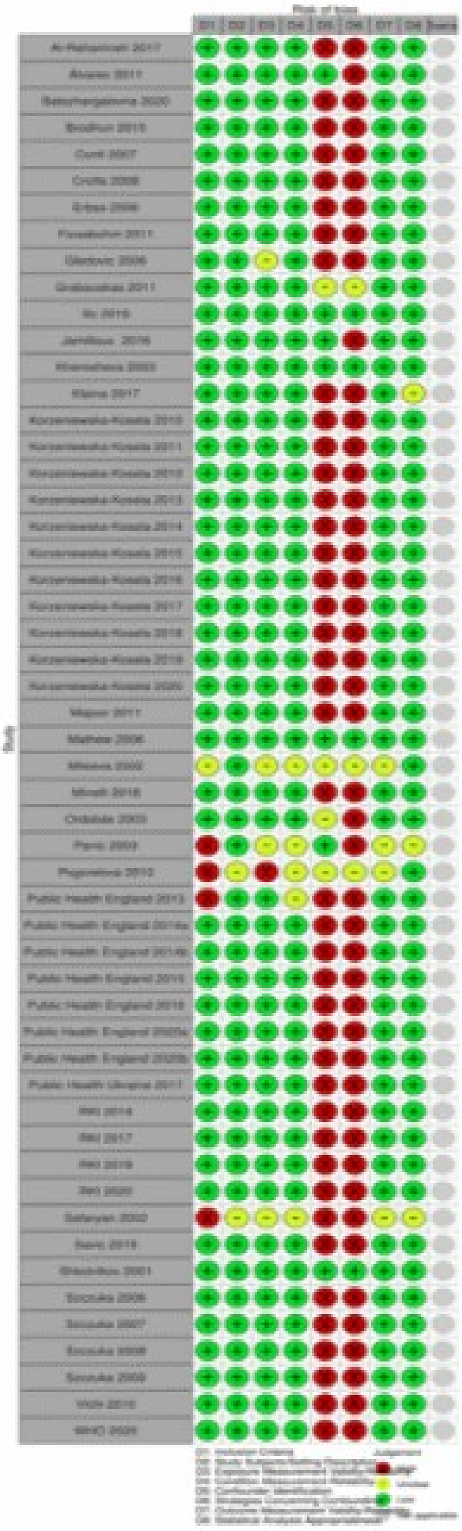

Figure A- 10: Descriptive studies - Traffic light panel of quality assessment results

VI. *Interventional studies*

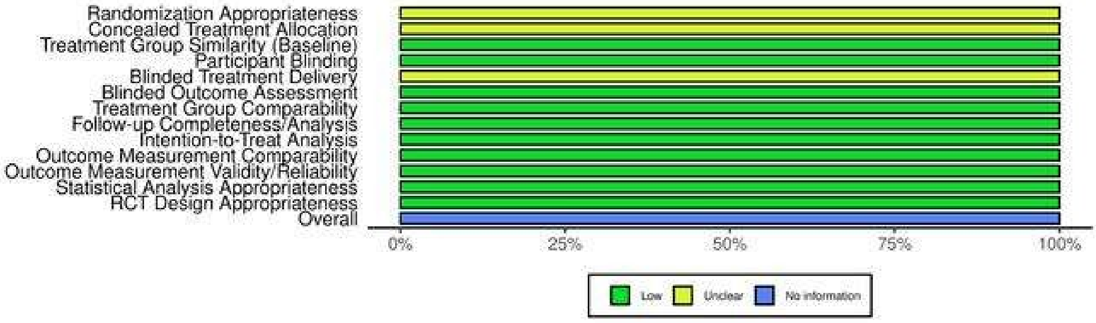

Figure A- 11: Interventional studies - Summary of quality assessment results

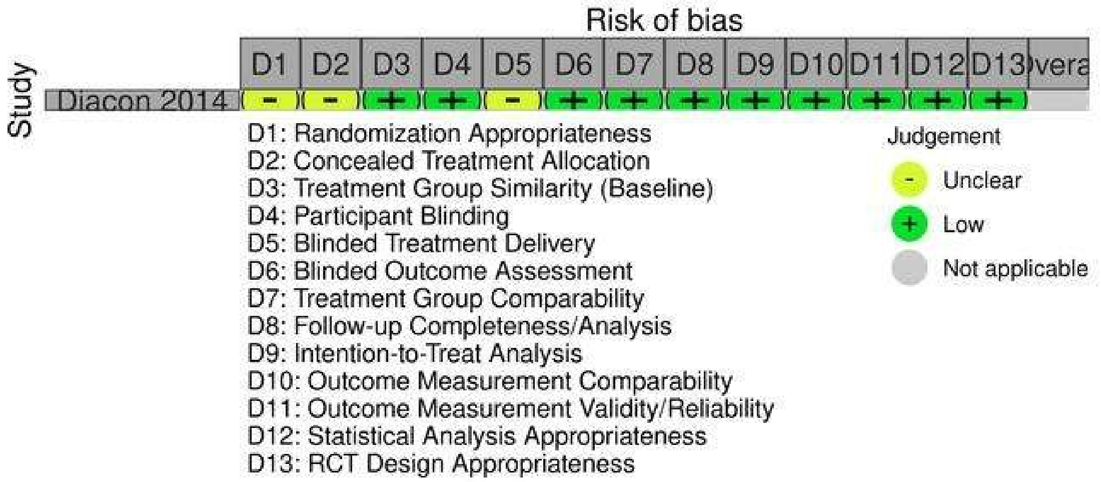

Figure A- 12: Interventional studies - Traffic light panel of quality assessment results
