# Supplementary material for: Tuberculosis case fatality is higher in male than female patients in Europe: a systematic review and meta-analysis
Source: Infection. 2024 Mar 23;52(5):1775–86. doi: 10.1007/s15010-024-02206-z (PMC11499538; doi:10.1007/s15010-024-02206-z)
Supplement: Supplementary file 16 — Online Resource 16 Study characteristics of the publications included in quantitative analysis, their categories used in the subgroup analyses, plus results of the publication bias assessment (PDF 188 KB) [file 15010_2024_2206_MOESM16_ESM.pdf]

*Table A- 6: Study characteristics and their categories used in the subgroup analyses*

| Study characteristic | Categories |
|---|---|
| Countries | Armenia |
| | Belarus |
| | Czech Republic |
| | Denmark |
| | Estonia |
| | Finland |
| | France |
| | Georgia |
| | Germany |
| | Israel |
| | Italy |
| | Latvia |
| | Lithuania |
| | Netherlands |
| | Norway |
| | Poland |
| | Portugal |
| | Romania |
| | Russia |
| | Serbia |
| | Slovakia |
| | Spain |
| | Switzerland |
| | Tajikistan |
| | Turkey |
| | Ukraine |
| | United Kingdom |
| | Uzbekistan |
| | Yugoslavia |
| | Europe |
| | Central Europe |
| | Eastern Europe |
| | Western Europe |
| Regions | Europe |
| | Central Europe |
| | Eastern Europe |
| | Western Europe |
| Autopsy/ death case studies vs. other studies | Alive |
| | Dead |
| Data source | Hospital data |

| **Study characteristic** | **Categories** |
|---|---|
| | Notification data |
| | TB dispensary data |
| Design | Case-control |
| | Case series |
| | Cohort |
| | Cross-sectional |
| | Descriptive |
| | Interventional (trial) |
| Observation period | 1980s-1990s |
| | 1990s |
| | 1990s-2000s |
| | 1990-2010s |
| | 2000s |
| | 2000s-2010s |
| | 2010s |
| Population | Death cases |
| | Drug-resistant TB cases |
| | Persons living with HIV |
| | Specific patients (hospitalized) |
| | TB cases, all forms |
| Publication date | Published 1990-99 |
| | Published 2000-09 |
| | Published 2010-19 |
| | Published 2020- |
| Size | Group size less than 30 |
| | Group size equal to or more than 30 |

**Publication Bias Assessment**

| Reported Outcome | No. Studies | Trim-Fill Method | Visual Assessment | Egger's test (P-value) | Begg's test (P-value) |
|---|---|---|---|---|---|
| Hazard ratios | 3 | yes | Asymmetry | **0.0496** | 0.1172 |
| Mortality rates | 15 | yes | Asymmetry | 0.0578 | 0.4002 |
| Mortality rates | 14 | yes | No asymmetry | 0.7405 | 0.2503 |
| Odds ratios | 7 | yes | No asymmetry | 0.2844 | 0.2931 |
| Absolute numbers | 94 | yes | Asymmetry | **0.0001** | 0.1461 |
| Absolute numbers | 77 | yes | No asymmetry (for studies w/ small SE) | 0.2529 | 0.0105 |
| Absolute numbers | 75 | yes | No asymmetry (for studies w/ small SE) | 0.2492 | 0.0049 |
| Absolute numbers | 67 | yes | No asymmetry (for studies w/ small SE) | 0.1408 | 0.2626 |

| Reported Outcome | No. Studies | Trim-Fill Method | Visual Assessment | Egger's test (z-value) | Egger's test (P-value) | Begg's test Kendall's τ) | Begg's test (P-value) |
|---|---|---|---|---|---|---|---|
| Hazard ratios | 3 | no | No asymmetry | 0.722 | 0.471 | 1 | 0.333 |
| Mortality rates | 15 | no | Data not available | | | | |
| Mortality rates | 14 | no | No asymmetry | -0.046 | 0.963 | -0.187 | 0.388 |
| Odds ratios | 7 | no | No asymmetry | -0.729 | 0.466 | -0.333 | 0.381 |
| Absolute numbers | 94 | no | Data not available | | | | |
| Absolute numbers | 77 | no | Data not available | | | | |
| Absolute numbers | 75 | no | No asymmetry | 0.9 | 0.368 | 0.23 | 0.003 |
| Absolute numbers | 67 | no | No asymmetry | -0.785 | 0.433 | 0.102 | 0.226 |
