# Supplementary material for: Tuberculosis case fatality is higher in male than female patients in Europe: a systematic review and meta-analysis
Source: Infection. 2024 Mar 23;52(5):1775–86. doi: 10.1007/s15010-024-02206-z (PMC11499538; doi:10.1007/s15010-024-02206-z)
Supplement: Supplementary file 18 — Online Resource 18 Listing of the 17 publications exclusively reporting TB death or autopsy cases (PDF 186 KB) [file 15010_2024_2206_MOESM18_ESM.pdf]

Publications exclusively reporting TB death or autopsy cases

1. Álvarez, J. L., A. E. Kunst, M. Leinsalu, M. Bopp, Str, B. H., G. Menvielle, et al. 2011. “Educational Inequalities in Tuberculosis Mortality in Sixteen European Populations.” International Journal of Tuberculosis and Lung Disease 15 (11): 1461–67.

2. Arghir, O. C., E. Dantes, M. Otelea, A. Rascu, E. Borgazi, and S. C. Cambrea. 2018. “Eight Year Survey of Tuberculosis In-Hospital Mortality in the South Eastern Part of Romania.” Romanian Journal of Legal Medicine 26 (2): 183–87.

3. Conti, S., S. M. D’Ottavi, M. Masocco, P. Meli, G. Minelli, G. Rago, R. Solimini, et al. 2007. “Mortality in Italy in 2001.” Rapporti ISTISAN - Istituto Superiore Di Sanità, 185.

4. Forssbohm, M., R. Kropp, G. Loytved, A. Neher, M. Simma, M. Rabbow, and H. Becher. n.d. “Death Due to Tuberculosis Requiring Treatment or an Accompanying Disease? A Contribution to the Lethality and Mortality of Tuberculosis in Germany.” Pneumologie 65 (10): 607–14.

5. Grabauskas, V., A. Gaizauskiene, S. Sauliune, and R. Miseikyte. 2011. “Trends in Avoidable Mortality in Lithuania during 2001-2008 and Their Impact on Life Expectancy.” Medicina (Kaunas, Lithuania) 47 (9): 504–11.

6. Jamilloux, Y., M. B. Delphine, S. Kerever, M. Gerfaud-Valentin, C. Broussolle, M. Eb, D. Valeyre, and P. Seve. 2016. “Sarcoidosis-Related Mortality in France: A Multiple-Cause-of-Death Analysis.” European Respiratory Journal 48 (6): 1700–1709.

7. Kleina, R., O. Fjodorova, A. Dabužinskienė, J. Nazarovs, and O. Mahmajeva. 2017. “Multiform Spectrum of Pulmonary Disease in Lethal HIV Infection Cases in Latvia (2012-2016).” Papers on Anthropology 26 (2): 53–67.

8. Mileeva, L. M., and V. P. Motovilova. 2002. “Medicosocial Structure and Cause of Death of Patients with Tuberculosis.” Problemy Tuberkuleza, no. 11: 16–17; discussion 17-18.

9. Minelli, G., M. Demaria, V. Manno, M. Vichi, S. M. D’Ottavi, G. Loreto, L. d Pasquale, et al. 2018. “Mortality in Italy in 2015.” Rapporti ISTISAN - Istituto Superiore Di Sanità, ii +160.

10. Ordobás, M., G, A. arillas, K. Fernández de la Hoz, and S. Fernández Rodríguez. 2003. “Mortality and Tuberculosis: Analysis of Multiple Cause-of-Death Data in the Community of Madrid, 1991-1998.” Revista Española de Salud Pública 77 (2): 189–200.

11. Pogorelova, E. 2010. “On Developing the Indicators of Tuberculosis Mortality in Russia.” Problemy Sotsial’noi Gigieny, Zdravookhraneniia i Istorii Meditsiny, no. 2: 3–6.

12. Safarian, M., and L. Nikolaian. 2002. “Analysis of Tuberculosis Mortality in Armenia.” Problemy Tuberkuleza, no. 5: 12.

13. Savic, I., V. Trifunovic-Skodric, and D. Mitrovic. 2016. “Clinically Unrecognized Miliary Tuberculosis: An Autopsy Study.” Annals of Saudi Medicine 36 (1): 42–50.

14. Theegarten, D., B. Kahl, and M. Ebsen. 2006. “Frequency and Morphology of Tuberculosis in Autopsies: Increase of Active Forms.” Deutsche Medizinische Wochenschrift 131 (24): 1371–76.

15. Valek, M., S. Sulkova, A. Slovakova, M. Drobilkova, and M. Sagova. 2003. “Tuberculosis in Patients with Chronic Kidney Failure.” Casopis Lekaru Ceskych 142 (5): 271–75.

16. Vichi, M., G. Minelli, S. M. D’Ottavi, G. Loreto, V. Manno, M. Masocco, G. Rago, et al. 2013. “Mortality in Italy in 2010.” Rapporti ISTISAN - Istituto Superiore Di Sanità, ii + 155.

17. Zaridze, D., P. Brennan, J. Boreham, A. Boroda, R. Karpov, A. Lazarev, I. Konobeevskaya, et al. 2009. “Alcohol and Cause-Specific Mortality in Russia: A Retrospective Case-Control Study of 48 557 Adult Deaths.” Lancet (British Edition) 373 (9682): 2201–14.
