# Supplementary material for: Tuberculosis case fatality is higher in male than female patients in Europe: a systematic review and meta-analysis
Source: Infection. 2024 Mar 23;52(5):1775–86. doi: 10.1007/s15010-024-02206-z (PMC11499538; doi:10.1007/s15010-024-02206-z)

Study	Males Events	Males Total	Females Events	Females Total	Weight	Risk Ratio MH, Random, 95% CI
Alvarez 2011	6227	8530	2303	8530	6.6%	2.704 [2.605, 2.806]
Arghir 2018	213	247	34	247	6.0%	6.265 [4.567, 8.594]
Conti 2007	254	406	152	406	6.5%	1.671 [1.443, 1.935]
Forssbohm 2011	139	223	84	223	6.4%	1.655 [1.359, 2.016]
Grabauskas 2011	1784	2130	346	2130	6.6%	5.156 [4.674, 5.688]
Jamilloux 2016	6	16	10	16	4.2%	0.600 [0.287, 1.255]
Kleina 2017	81	104	23	104	5.8%	3.522 [2.421, 5.124]
Mileeva 2002	52	62	10	62	4.9%	5.200 [2.917, 9.269]
Minelli 2018	171	308	137	308	6.5%	1.248 [1.064, 1.465]
Ordobas 2003	915	1206	291	1206	6.6%	3.144 [2.831, 3.493]
Pogorelova 2010	258	303	45	303	6.2%	5.733 [4.361, 7.538]
Safaryan 2002	163	198	35	198	6.1%	4.657 [3.425, 6.333]
Savic 2016	14	35	21	35	5.3%	0.667 [0.409, 1.086]
Theegarten 2006	34	55	21	55	5.7%	1.619 [1.090, 2.404]
Valek 2003	7	11	4	11	3.6%	1.750 [0.711, 4.306]
Vichi 2010	199	350	151	350	6.5%	1.318 [1.133, 1.533]
Zaridze 2009	709	812	103	812	6.4%	6.883 [5.736, 8.260]
**Total (95% CI)**		**14996**		**14996**	**100.0%**	**2.565 [1.995, 3.298]**

Heterogeneity: $Tau^2 = 0.247$; $Chi^2 = 667.89$, df = 16 ($P < 0.01$); $I^2 = 98\%$

Test for overall effect: $Z = 7.34$ ($P < 0.01$)

Risk Ratio

MH, Random, 95% CI

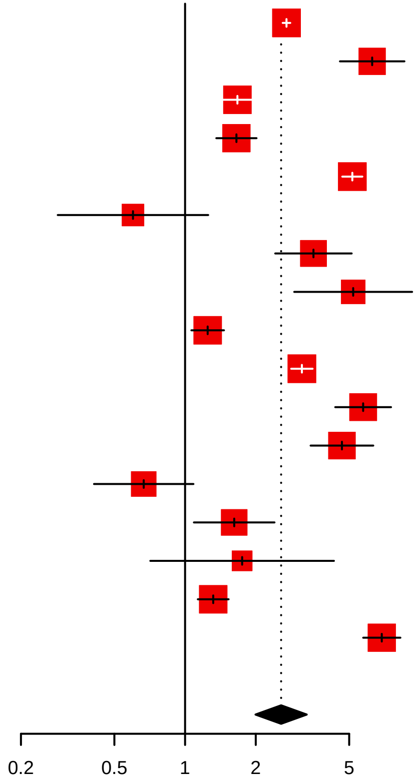

Supplement: Supplementary file 22 — Online Resource 22 Forest plot of publications reporting death cases in absolute numbers (n = 17) (PDF 7 KB) [file 15010_2024_2206_MOESM22_ESM.pdf]
