# Supplementary material for: Tuberculosis case fatality is higher in male than female patients in Europe: a systematic review and meta-analysis
Source: Infection. 2024 Mar 23;52(5):1775–86. doi: 10.1007/s15010-024-02206-z (PMC11499538; doi:10.1007/s15010-024-02206-z)
Supplement: Supplementary file 23 — Online Resource 23 Forest plot of publications reporting mortality rates (MR), calculated by absolute numbers (n = 15) (PDF 7 KB) [file 15010_2024_2206_MOESM23_ESM.pdf]

Study	Males Events	Males Total	Females Events	Females Total	Weight	Risk Ratio MH, Random, 95% CI
Brodhun 2015	24	10000000	12	10000000	2.3%	2.000 [1.000, 3.999]
Brya 2017	353	10000000	98	10000000	7.3%	3.602 [2.880, 4.505]
Gledovic 2006	600	10000000	230	10000000	8.5%	2.609 [2.241, 3.037]
Hauer 2011	120	10000000	70	10000000	6.2%	1.714 [1.277, 2.302]
Korzeniewska−Kosela 2010	320	10000000	80	10000000	7.0%	4.000 [3.131, 5.110]
Korzeniewska−Kosela 2011	330	10000000	90	10000000	7.2%	3.667 [2.904, 4.629]
Korzeniewska−Kosela 2012	320	10000000	80	10000000	7.0%	4.000 [3.131, 5.110]
Korzeniewska−Kosela 2013	250	10000000	60	10000000	6.4%	4.167 [3.144, 5.523]
Korzeniewska−Kosela 2014	270	10000000	70	10000000	6.7%	3.857 [2.966, 5.017]
Korzeniewska−Kosela 2015	260	10000000	80	10000000	6.9%	3.250 [2.530, 4.176]
Korzeniewska−Kosela 2016	220	10000000	60	10000000	6.3%	3.667 [2.756, 4.878]
Szczuka 2006	350	10000000	80	10000000	7.0%	4.375 [3.432, 5.578]
Szczuka 2007	340	10000000	90	10000000	7.2%	3.778 [2.995, 4.766]
Szczuka 2008	340	10000000	90	10000000	7.2%	3.778 [2.995, 4.766]
Szczuka 2009	310	10000000	80	10000000	7.0%	3.875 [3.031, 4.955]
**Total (95% CI)**		**150000000**		**150000000**	**100.0%**	**3.470 [3.075, 3.915]**

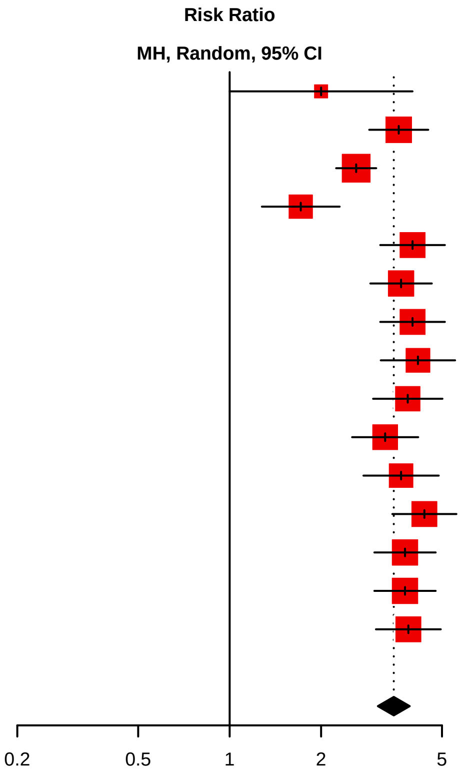

Heterogeneity: $Tau^2 = 0.039$; $Chi^2 = 48.80$, $df = 14$ ($P < 0.01$); $I^2 = 71\%$

Test for overall effect: $Z = 20.19$ ($P < 0.01$)
