# Supplementary material for: Tuberculosis case fatality is higher in male than female patients in Europe: a systematic review and meta-analysis
Source: Infection. 2024 Mar 23;52(5):1775–86. doi: 10.1007/s15010-024-02206-z (PMC11499538; doi:10.1007/s15010-024-02206-z)

	Males		Females			Risk Ratio
**Study**	**Events**	**Total**	**Events**	**Total**	**Weight**	**MH, Random, 95% CI**
Al−Rahamneh 2017	406	10000000	94	10000000	13.9%	4.319 [3.451, 5.405]
Shkolnikov 2001 (1991)	2160	10000000	220	10000000	14.8%	9.818 [8.547, 11.279]
Shkolnikov 2001 (1994)	3800	10000000	360	10000000	15.0%	10.556 [9.474, 11.760]
Shkolnikov 2001 (1998)	3820	10000000	430	10000000	15.0%	8.884 [8.041, 9.815]
Shkolnikov 2013 RUS (2010)	2510	10000000	520	10000000	15.1%	4.827 [4.392, 5.305]
Shkolnikov 2013 RUS (2003)	3910	10000000	590	10000000	15.1%	6.627 [6.078, 7.226]
Shkolnikov 2013 UK (2009)	60	10000000	30	10000000	11.1%	2.000 [1.290, 3.100]
**Total (95% CI)**		**70000000**		**70000000**	**100.0%**	**6.188 [4.686, 8.171]**

Heterogeneity: $Tau^2 = 0.131$; $Chi^2 = 212.10$, df = 6 ($P < 0.01$); $I^2 = 97\%$

Test for overall effect: $Z = 12.85$ ($P < 0.01$)

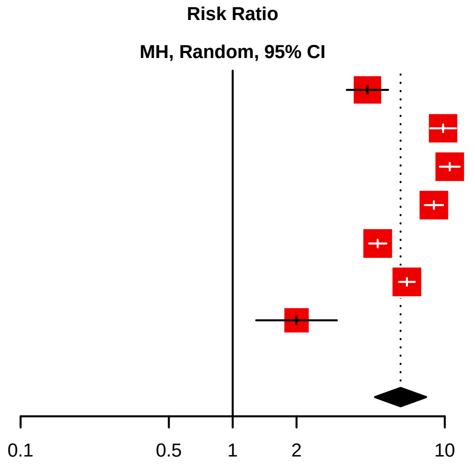

Supplement: Supplementary file 24 — Online Resource 24 Forest plot of publications reporting standardized mortality rates (SMR) (PDF 6 KB) [file 15010_2024_2206_MOESM24_ESM.pdf]
