# Supplementary material for: Tuberculosis case fatality is higher in male than female patients in Europe: a systematic review and meta-analysis
Source: Infection. 2024 Mar 23;52(5):1775–86. doi: 10.1007/s15010-024-02206-z (PMC11499538; doi:10.1007/s15010-024-02206-z)
Supplement: Supplementary file 27 — Online Resource 27 Univariate meta-regression – Moderator forest and bubble plots and prediction results (PDF 1472 KB) [file 15010_2024_2206_MOESM27_ESM.pdf]

Meta-regression – Moderator forest and bubble plots and prediction results

I. *Moderator age group 15-24*

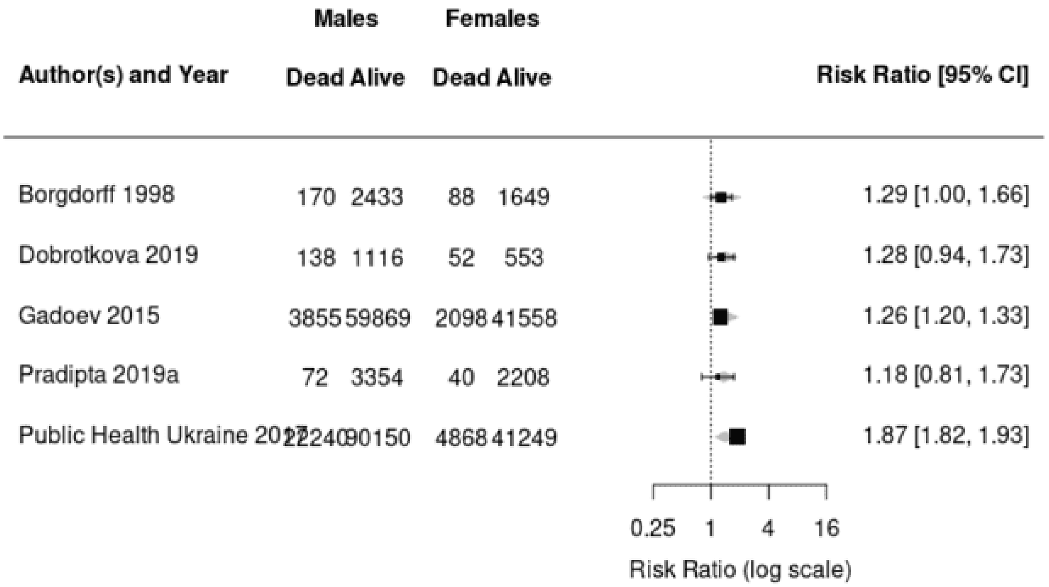

Figure A- 13: Forest plot of moderator age group 15-24

Moderator age group 15-24 - Predicted pooled risk ratio (with 95% confidence/prediction intervals)

	pred	ci.lb	ci.ub	pi.lb	pi.ub
Borgdorff 1998	1.31	0.84	2.04	0.71	2.42
Dobrotkova 2019	1.42	1.09	1.86	0.86	2.36
Gadoev 2015	1.43	1.07	1.91	0.86	2.39
Pradipta 2019a	1.36	1.05	1.76	0.83	2.23
Public Health Ukraine 2017	1.41	1.12	1.76	0.87	2.27

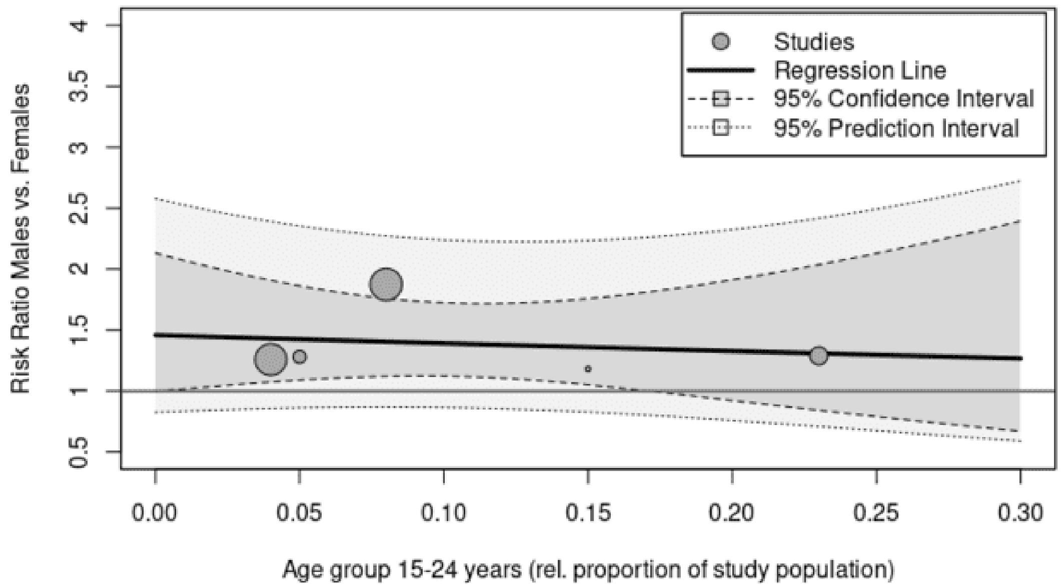

Figure A- 15: Bubble plot of moderator age group 15-24

II. Moderator age group 25-44

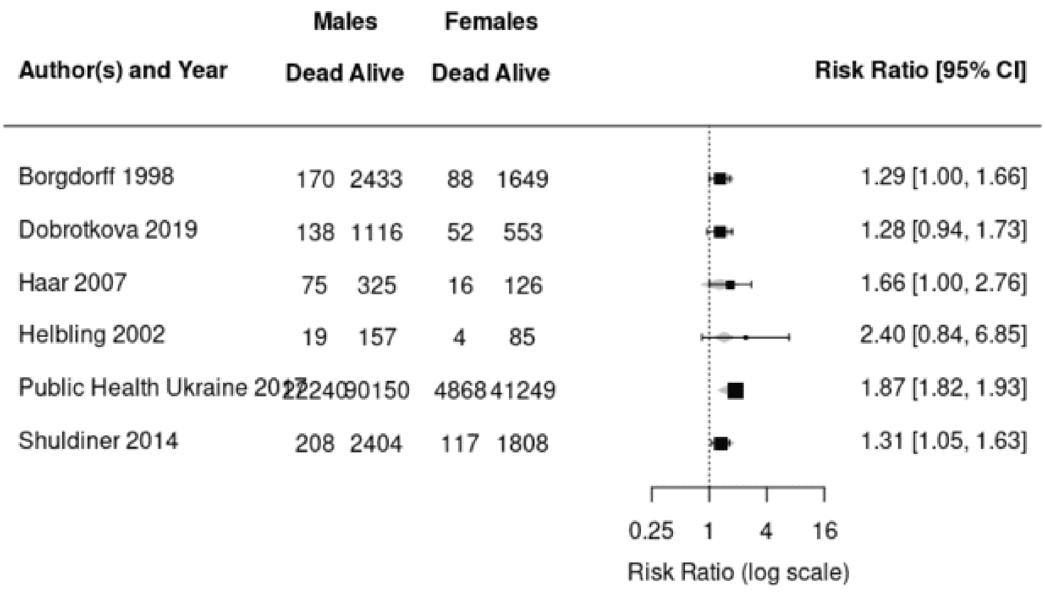

Figure A- 17: Forest plot of moderator age group 25-44

Moderator age group 25-44 - Predicted pooled risk ratio (with 95% confidence/prediction intervals)

	pred	ci.lb	ci.ub	pi.lb	pi.ub
Borgdorff 1998	1.52	1.27	1.81	1.06	2.18
Dobrotkova 2019	1.43	1.13	1.80	0.97	2.11
Haar 2007	1.29	0.83	2.02	0.75	2.23
Helbling 2002	1.43	1.15	1.79	0.98	2.11
Public Health Ukraine 2017	1.63	1.25	2.12	1.08	2.46
Shuldiner 2014	1.51	1.27	1.80	1.05	2.17

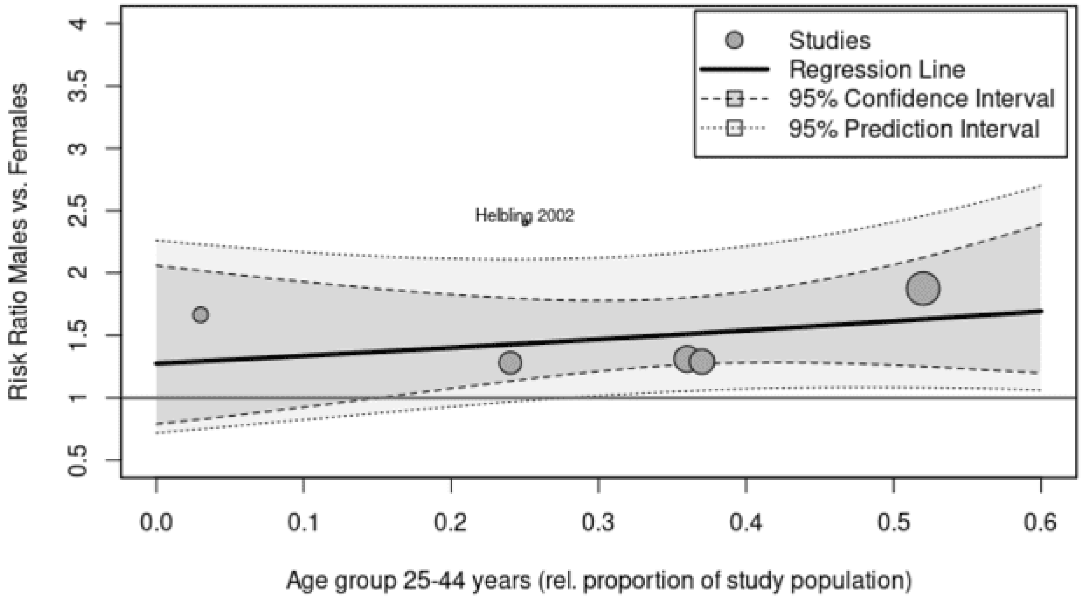

Figure A- 19: Bubble plot of moderator age group 25-44

III. Moderator age group 15-44

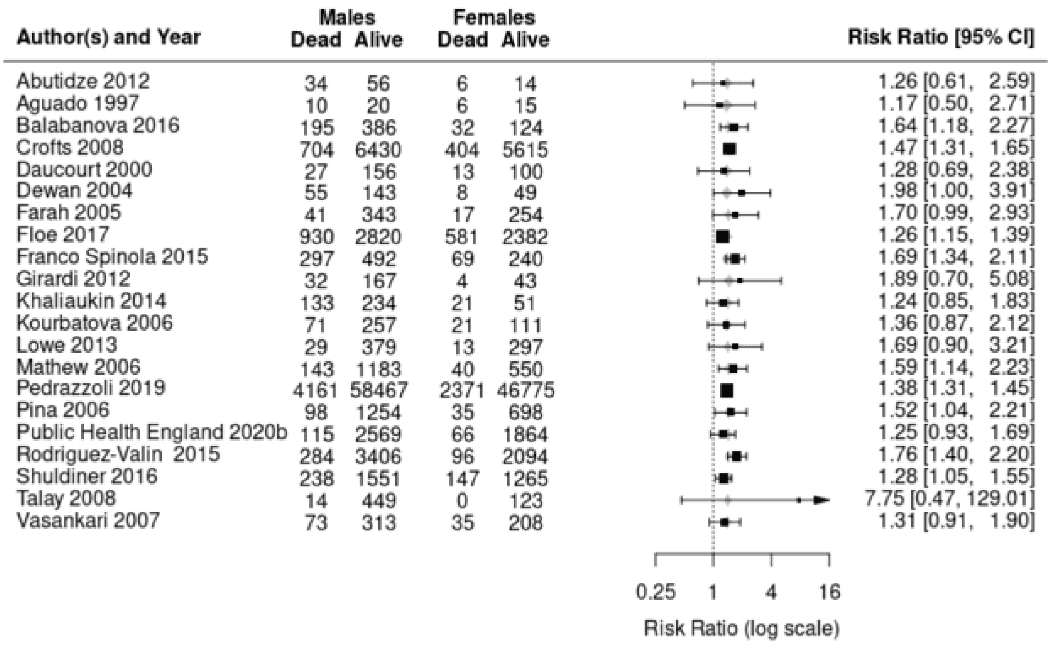

Figure A- 21: Forest plot of moderator age group 15-44

Moderator age group 15-44 - Predicted pooled risk ratio (with 95% confidence/prediction intervals)

	pred	ci.lb	ci.ub	pi.lb	pi.ub
Abutidze 2012	1.39	1.27	1.52	1.19	1.63
Aguado 1997	1.37	1.20	1.56	1.14	1.65
Balabanova 2016	1.41	1.32	1.51	1.22	1.64
Crofts 2008	1.44	1.33	1.56	1.23	1.68
Daucourt 2000	1.37	1.21	1.56	1.14	1.65
Dewan 2004	1.38	1.23	1.55	1.16	1.64
Farah 2005	1.43	1.33	1.53	1.23	1.66
Floe 2017	1.38	1.22	1.55	1.15	1.64
Franco Spinola 2015	1.38	1.24	1.54	1.17	1.64
Girardi 2012	1.45	1.31	1.61	1.23	1.72
Khaliaukin 2014	1.42	1.33	1.52	1.23	1.65
Kourbatova 2006	1.38	1.23	1.55	1.16	1.64
Lowe 2013	1.41	1.32	1.51	1.22	1.63
Mathew 2006	1.43	1.33	1.54	1.23	1.67
Pedrazzoli 2019	1.45	1.32	1.59	1.23	1.70
Pina 2006	1.44	1.33	1.56	1.23	1.68
Public Health England 2020b	1.43	1.33	1.54	1.23	1.66
Rodriguez-Valin 2015	1.43	1.33	1.54	1.23	1.66
Shuldiner 2016	1.43	1.33	1.54	1.23	1.66
Talay 2008	1.41	1.32	1.51	1.22	1.63

Vasankari 2007	1.39	1.28	1.52	1.19	1.63

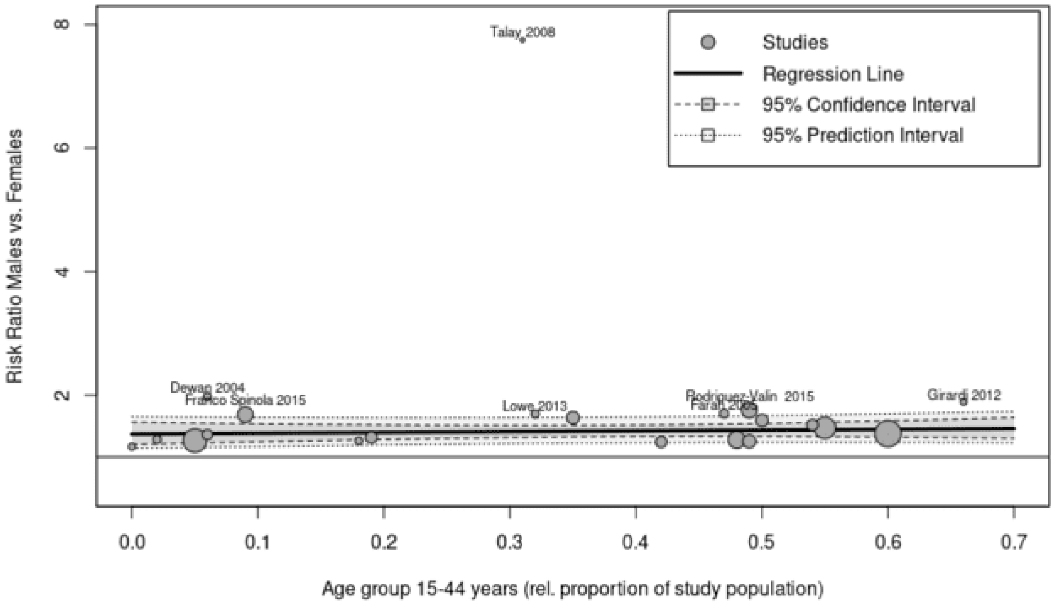

Figure A- 23: Bubble plot of moderator age group 15-44

IV. *Moderator age group 45-64*

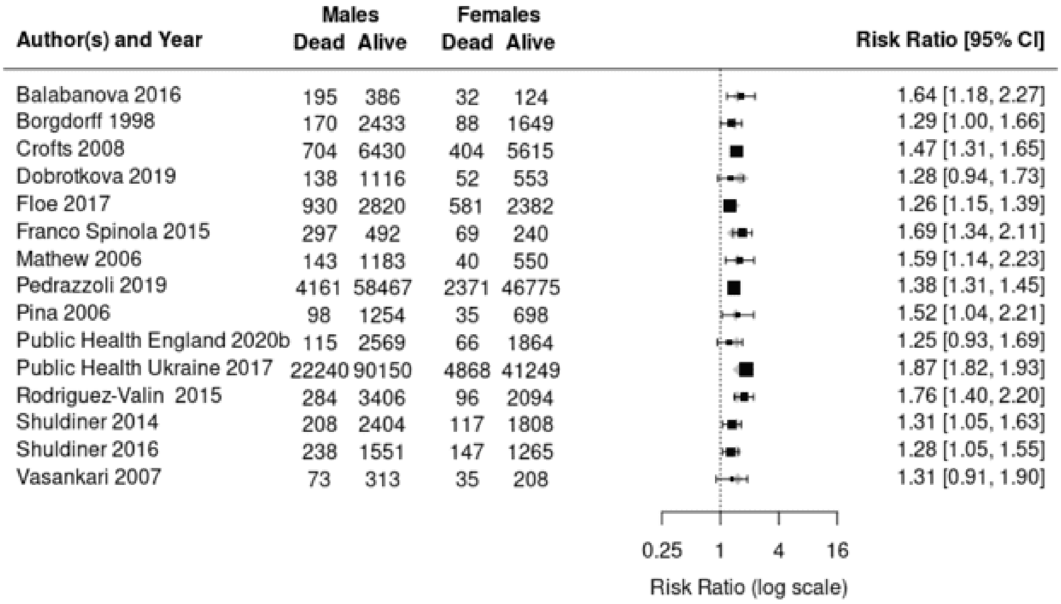

Figure A- 25: Forest plot of moderator age group 45-64

Moderator age group 45-64 - Predicted pooled risk ratio (with 95% confidence/prediction intervals)

	pred	ci.lb	ci.ub	pi.lb	pi.ub
Balabanova 2016	1.51	1.37	1.66	1.19	1.91
Borgdorff 1998	1.41	1.28	1.55	1.11	1.79
Crofts 2008	1.45	1.34	1.57	1.15	1.83
Dobrotkova 2019	1.62	1.35	1.93	1.22	2.14
Floe 2017	1.32	1.11	1.57	1.00	1.74
Franco Spinola 2015	1.36	1.18	1.56	1.05	1.76
Mathew 2006	1.50	1.37	1.64	1.19	1.90
Pedrazzoli 2019	1.45	1.34	1.57	1.15	1.83
Pina 2006	1.45	1.34	1.56	1.15	1.82
Public Health England 2020b	1.48	1.36	1.60	1.17	1.86
Public Health Ukraine 2017	1.55	1.37	1.75	1.21	1.99
Rodriguez-Valin 2015	1.48	1.37	1.61	1.18	1.87
Shuldiner 2014	1.46	1.35	1.58	1.16	1.84
Shuldiner 2016	1.48	1.37	1.61	1.18	1.87
Vasankari 2007	1.51	1.37	1.66	1.19	1.91

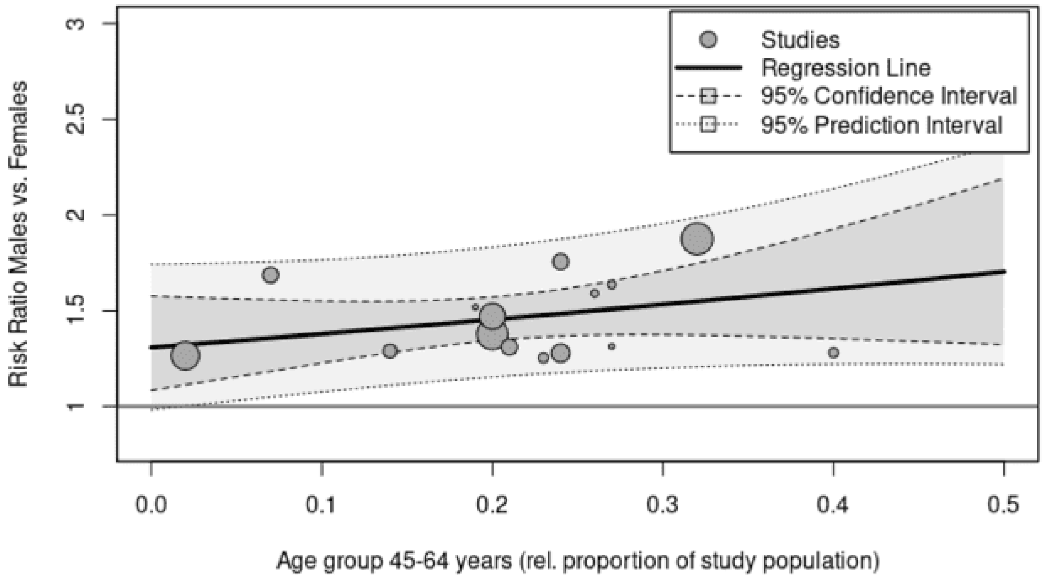

Figure A- 27: Bubble plot of moderator age group 45-64

V. *Moderator age group 65 and older*

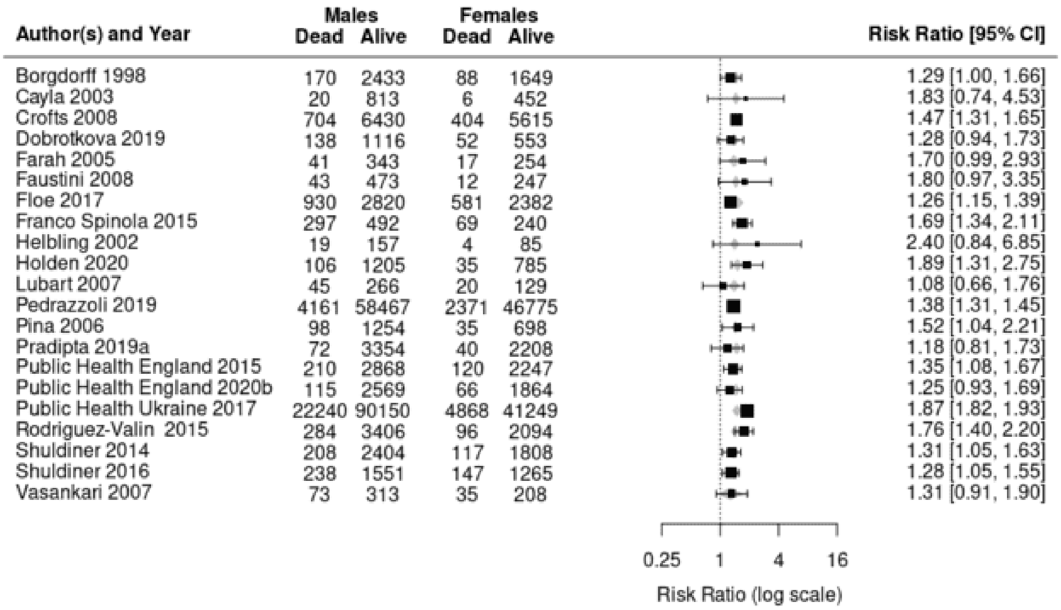

Figure A- 29: Forest plot of moderator age group 65 and older

Forest plot of moderator age group 65 and older - Predicted pooled risk ratio (with 95% confidence/prediction intervals)

	pred	ci.lb	ci.ub	pi.lb	pi.ub
Borgdorff 1998	1.46	1.35	1.58	1.13	1.88
Cayla 2003	1.45	1.34	1.57	1.13	1.87
Crofts 2008	1.44	1.33	1.56	1.12	1.85
Dobrotkova 2019	1.39	1.23	1.56	1.06	1.82
Farah 2005	1.38	1.22	1.57	1.06	1.81
Faustini 2008	1.45	1.34	1.57	1.13	1.87
Floe 2017	1.51	1.34	1.71	1.16	1.99
Franco Spinola 2015	1.50	1.35	1.67	1.15	1.95
Helbling 2002	1.41	1.28	1.55	1.09	1.83
Holden 2020	1.50	1.35	1.67	1.15	1.95
Lubart 2007	1.39	1.23	1.56	1.06	1.82
Pedrazzoli 2019	1.45	1.34	1.57	1.13	1.87
Pina 2006	1.43	1.32	1.55	1.11	1.85
Pradipta 2019a	1.48	1.35	1.62	1.14	1.91
Public Health England 2015	1.50	1.34	1.68	1.15	1.96
Public Health England 2020b	1.46	1.35	1.58	1.13	1.88
Public Health Ukraine 2017	1.49	1.35	1.64	1.15	1.93
Rodriguez-Valin 2015	1.42	1.30	1.55	1.10	1.84
Shuldiner 2014	1.39	1.25	1.56	1.07	1.82
Shuldiner 2016	1.39	1.25	1.56	1.07	1.82
Vasankari 2007	1.27	0.95	1.69	0.87	1.85

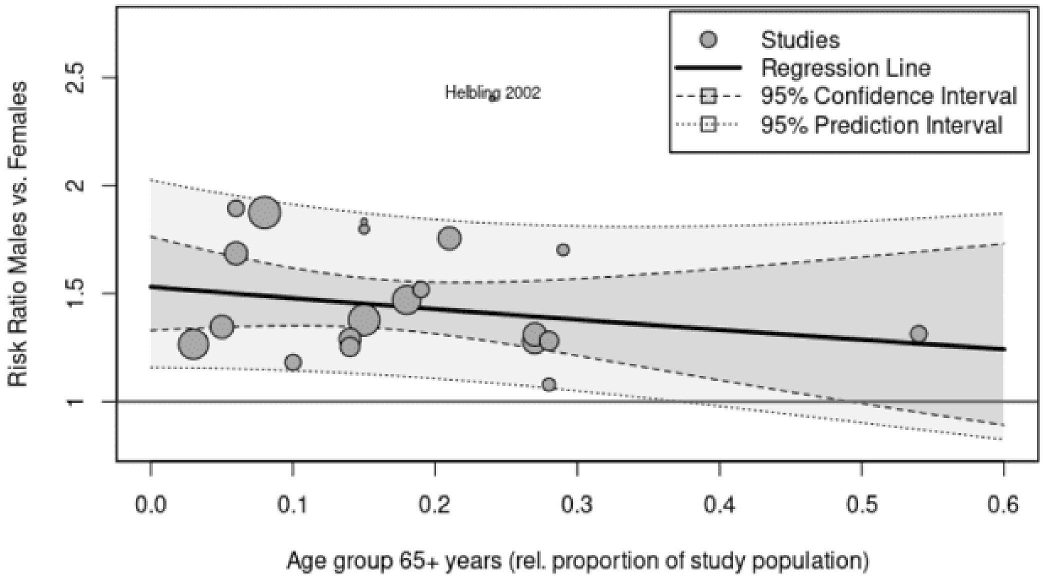

Figure A- 31: Bubble plot of moderator age group 65 and older

VI. *Moderator alcohol*

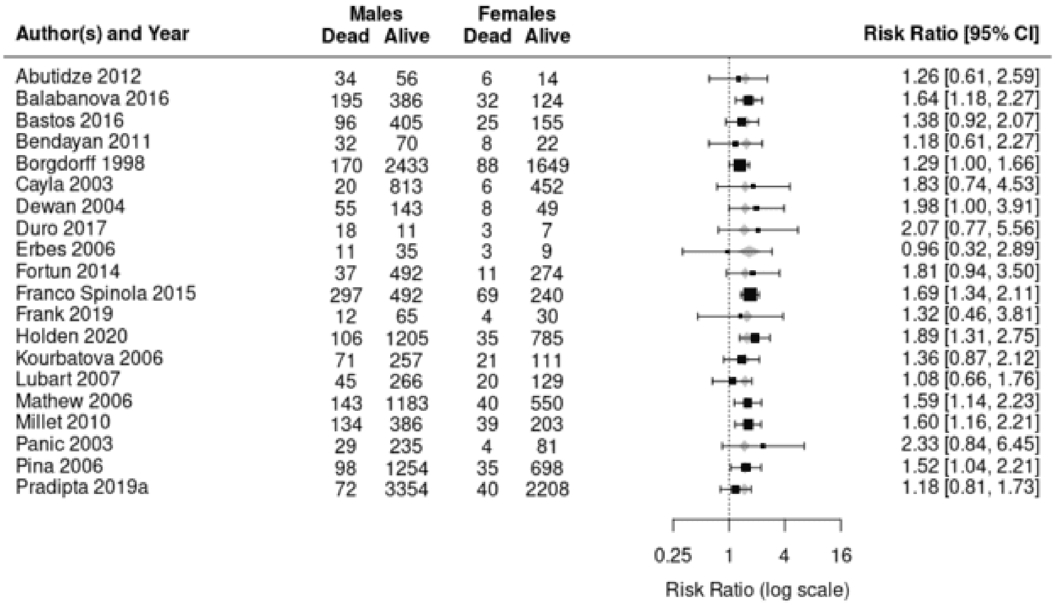

Figure A- 33: Forest plot of moderator alcohol

Moderator alcohol - Predicted pooled risk ratio (with 95% confidence/prediction intervals)

	pred	ci.lb	ci.ub	pi.lb	pi.ub
Abutidze 2012	1.51	1.37	1.66	1.37	1.66
Balabanova 2016	1.64	1.27	2.12	1.27	2.12
Bastos 2016	1.53	1.38	1.69	1.38	1.69
Bendayan 2011	1.54	1.37	1.72	1.37	1.72
Borgdorff 1998	1.46	1.28	1.65	1.28	1.65
Cayla 2003	1.49	1.35	1.64	1.35	1.64
Dewan 2004	1.49	1.35	1.64	1.35	1.64
Duro 2017	1.46	1.28	1.65	1.28	1.65
Erbes 2006	1.65	1.26	2.16	1.26	2.16
Fortun 2014	1.49	1.35	1.64	1.35	1.64
Franco Spinola 2015	1.47	1.31	1.64	1.31	1.64
Frank 2019	1.56	1.36	1.78	1.36	1.78
Holden 2020	1.56	1.36	1.79	1.36	1.79
Kourbatova 2006	1.48	1.33	1.64	1.33	1.64
Lubart 2007	1.49	1.35	1.64	1.35	1.64
Mathew 2006	1.55	1.37	1.75	1.37	1.75
Millet 2010	1.49	1.35	1.64	1.35	1.64
Panic 2003	1.46	1.30	1.65	1.30	1.65
Pina 2006	1.48	1.34	1.64	1.34	1.64
Pradipta 2019a	1.46	1.29	1.65	1.29	1.65

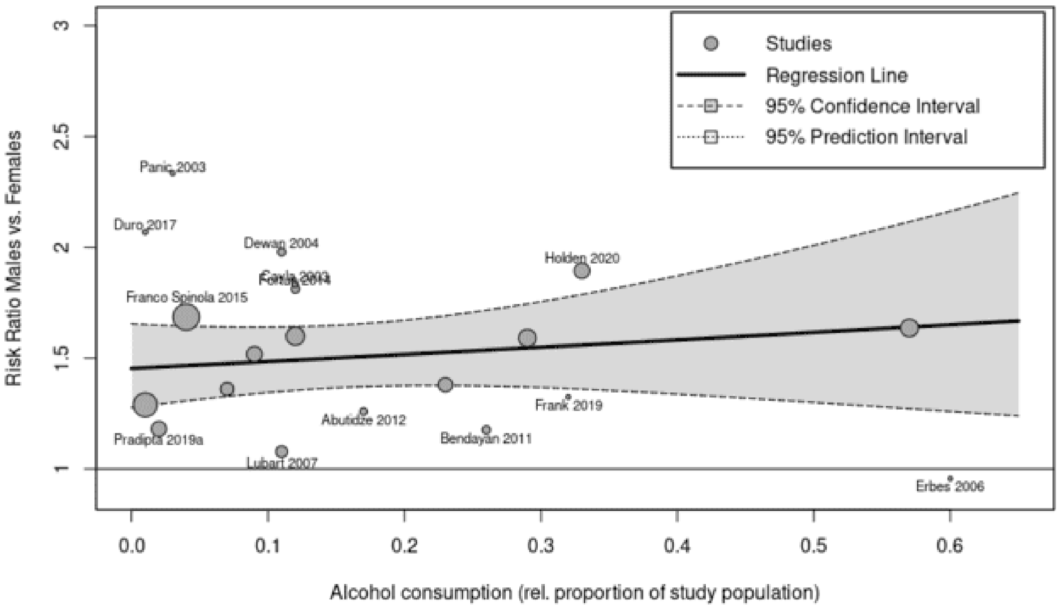

Figure A- 35: Bubble plot of moderator alcohol

VII. *Moderator any comorbidity*

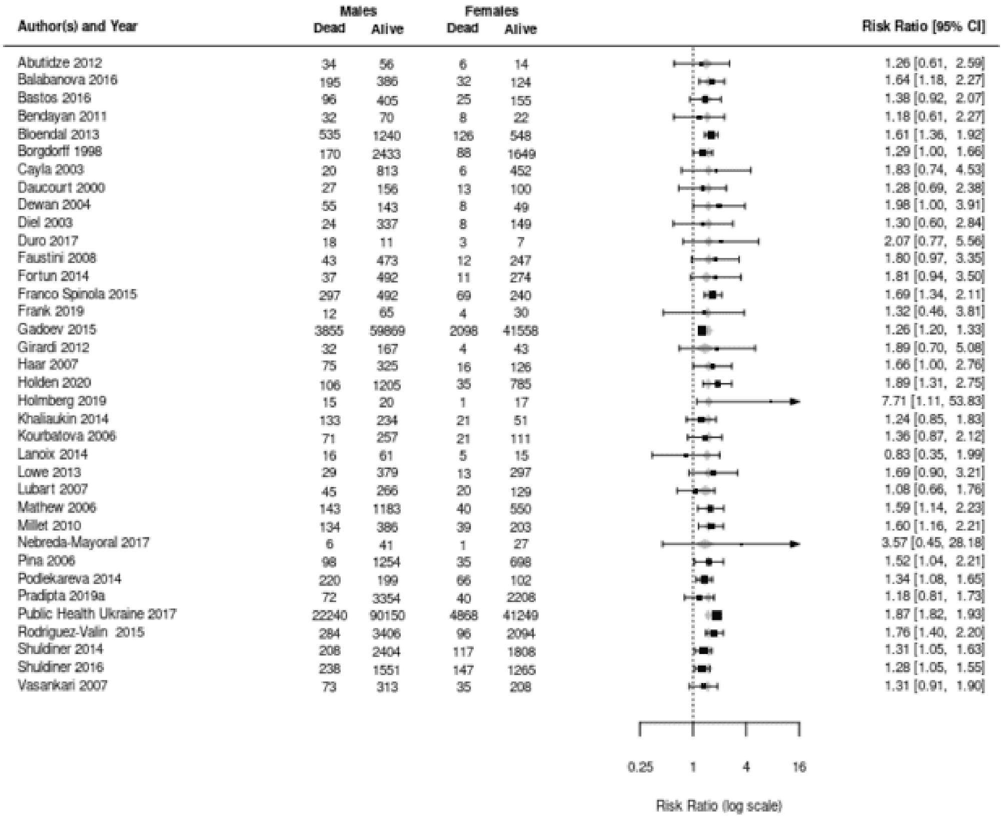

Figure A- 37: Forest plot of moderator any comorbidity

Moderator any comorbidity - Predicted pooled risk ratio (with 95% confidence/prediction intervals)

	pred	ci.lb	ci.ub	pi.lb	pi.ub
Abutidze 2012	1.41	1.22	1.63	1.07	1.87
Balabanova 2016	1.50	1.38	1.62	1.17	1.93
Bastos 2016	1.39	1.15	1.67	1.02	1.88
Bendayan 2011	1.46	1.33	1.59	1.13	1.88
Bloendal 2013	1.50	1.38	1.64	1.17	1.94
Borgdorff 1998	1.50	1.38	1.64	1.17	1.94
Cayla 2003	1.50	1.38	1.63	1.17	1.94
Daucourt 2000	1.50	1.38	1.62	1.16	1.93
Dewan 2004	1.50	1.38	1.62	1.16	1.92
Diel 2003	1.50	1.38	1.63	1.17	1.94
Duro 2017	1.51	1.38	1.65	1.17	1.95
Faustini 2008	1.51	1.38	1.65	1.17	1.95
Fortun 2014	1.42	1.24	1.63	1.08	1.87
Franco Spinola 2015	1.50	1.38	1.62	1.16	1.92
Frank 2019	1.47	1.36	1.59	1.14	1.89

Gadoev 2015	1.51	1.38	1.65	1.17	1.95
Girardi 2012	1.36	1.08	1.71	0.98	1.90
Haar 2007	1.51	1.38	1.64	1.17	1.94
Holden 2020	1.49	1.38	1.60	1.16	1.91
Holmberg 2019	1.51	1.38	1.64	1.17	1.94
Khaliaukin 2014	1.49	1.38	1.61	1.16	1.92
Kourbatova 2006	1.51	1.38	1.64	1.17	1.94
Lanoix 2014	1.45	1.32	1.59	1.12	1.87
Lowe 2013	1.49	1.38	1.61	1.16	1.92
Lubart 2007	1.38	1.14	1.68	1.02	1.88
Mathew 2006	1.51	1.38	1.64	1.17	1.94
Millet 2010	1.50	1.38	1.62	1.16	1.92
Nebreda-Mayoral 2017	1.36	1.08	1.71	0.98	1.90
Pina 2006	1.49	1.38	1.61	1.16	1.92
Podlekareva 2014	1.36	1.08	1.71	0.98	1.90
Pradipta 2019a	1.50	1.38	1.62	1.16	1.93
Public Health Ukraine 2017	1.49	1.38	1.60	1.16	1.91
Rodriguez-Valin 2015	1.50	1.38	1.63	1.17	1.94
Shuldiner 2014	1.50	1.38	1.64	1.17	1.94
Shuldiner 2016	1.51	1.38	1.64	1.17	1.94
Vasankari 2007	1.47	1.35	1.59	1.14	1.89

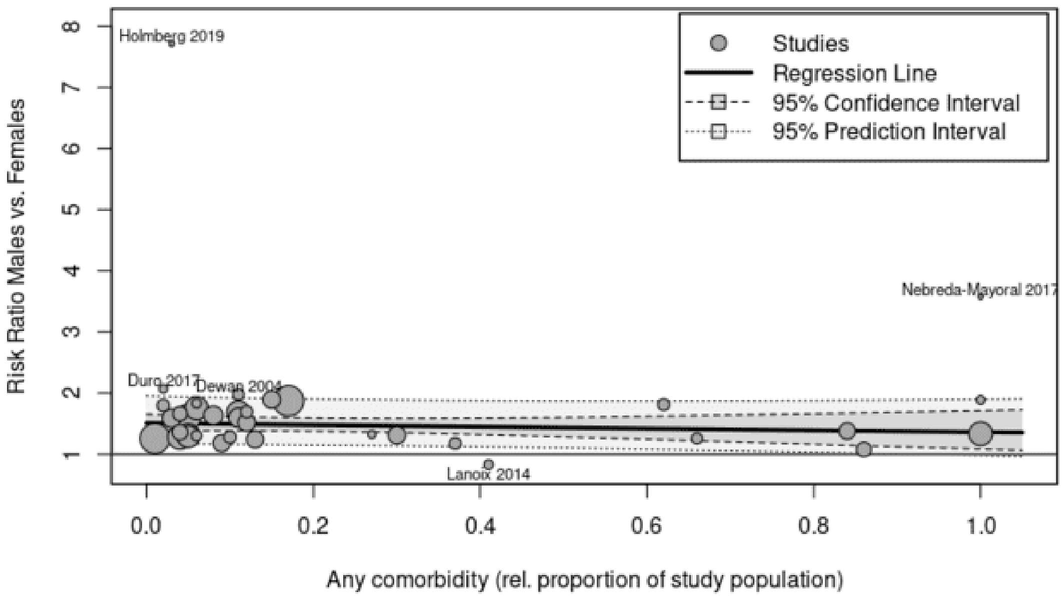

Figure A- 39: Bubble plot of moderator any comorbidity

VIII. *Moderator any comorbidity except for HIV/AIDS*

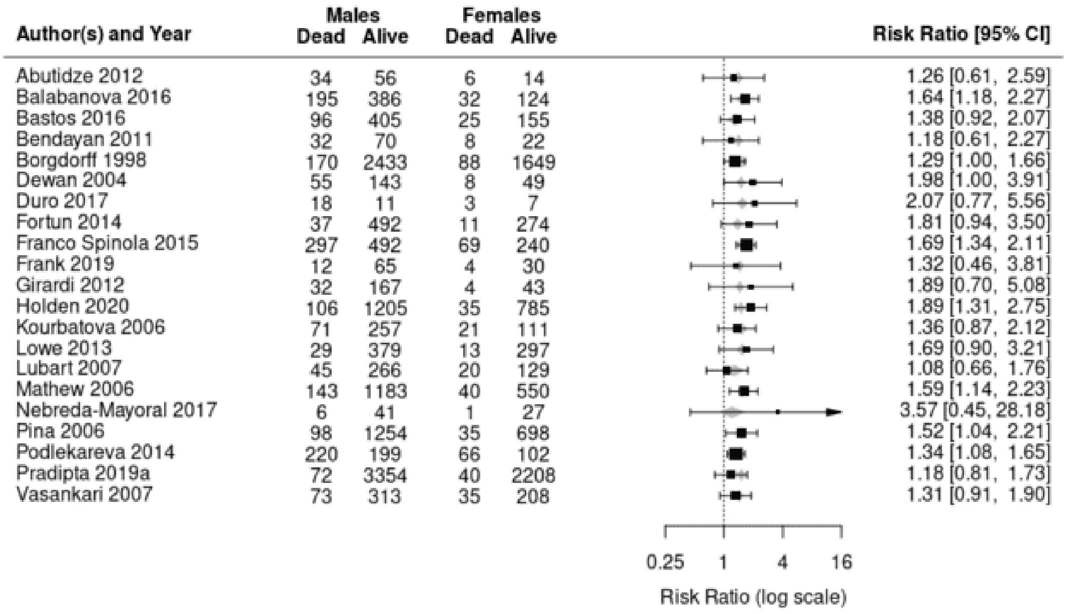

Figure A- 41: Forest plot of moderator any comorbidity except for HIV/AIDS

Moderator any comorbidity except for HIV/AIDS predicted pooled risk ratio (with 95% confidence/prediction intervals)

	pred	ci.lb	ci.ub	pi.lb	pi.ub
Abutidze 2012	1.40	1.26	1.56	1.26	1.56
Balabanova 2016	1.53	1.37	1.70	1.37	1.70
Bastos 2016	1.31	1.09	1.57	1.09	1.57
Bendayan 2011	1.41	1.27	1.56	1.27	1.56
Borgdorff 1998	1.54	1.37	1.72	1.37	1.72
Dewan 2004	1.50	1.37	1.66	1.37	1.66
Duro 2017	1.54	1.37	1.73	1.37	1.73
Fortun 2014	1.39	1.24	1.55	1.24	1.55
Franco Spinola 2015	1.52	1.37	1.68	1.37	1.68
Frank 2019	1.46	1.34	1.59	1.34	1.59
Girardi 2012	1.46	1.34	1.59	1.34	1.59
Holden 2020	1.50	1.37	1.65	1.37	1.65
Kourbatova 2006	1.53	1.37	1.71	1.37	1.71
Lowe 2013	1.53	1.37	1.72	1.37	1.72
Lubart 2007	1.28	1.04	1.57	1.04	1.57
Mathew 2006	1.54	1.37	1.72	1.37	1.72
Nebreda-Mayoral 2017	1.21	0.92	1.60	0.92	1.60
Pina 2006	1.52	1.37	1.69	1.37	1.69
Podlekareva 2014	1.34	1.16	1.56	1.16	1.56

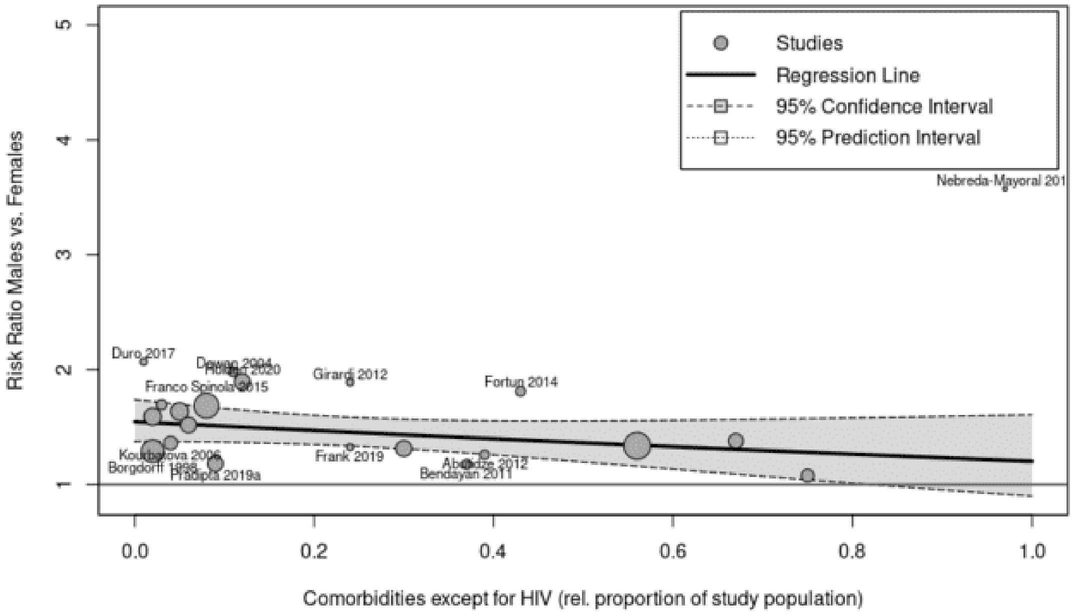

Figure A- 43: Bubble plot of moderator any comorbidity except for HIV/AIDS

IX. *Moderator cancers*

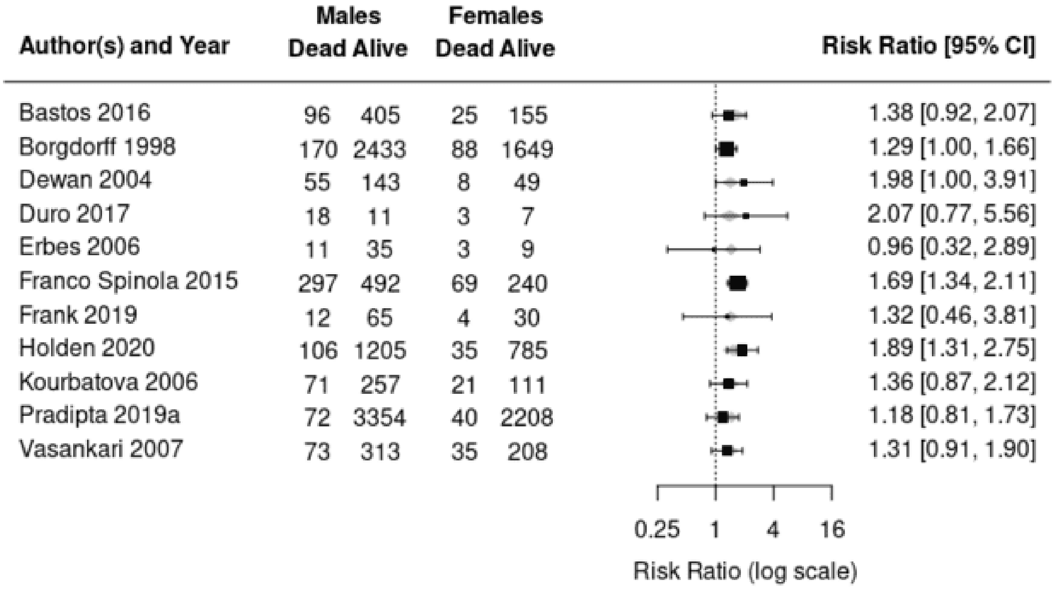

Figure A- 45: Forest plot of moderator cancers

Moderator cancers - Predicted pooled risk ratio (with 95% confidence/prediction intervals)

	pred	ci.lb	ci.ub	pi.lb	pi.ub
Bastos 2016	1.57	1.15	2.14	1.12	2.19
Borgdorff 1998	1.45	1.27	1.66	1.21	1.75
Dewan 2004	1.42	1.20	1.69	1.15	1.76
Duro 2017	1.40	1.11	1.76	1.07	1.82
Erbes 2006	1.45	1.27	1.66	1.21	1.75
Franco Spinola 2015	1.45	1.27	1.66	1.21	1.75
Frank 2019	1.42	1.20	1.69	1.15	1.76
Holden 2020	1.54	1.21	1.95	1.17	2.02
Kourbatova 2006	1.40	1.11	1.76	1.07	1.82
Pradipta 2019a	1.45	1.27	1.66	1.21	1.75
Vasankari 2007	1.45	1.27	1.66	1.21	1.75

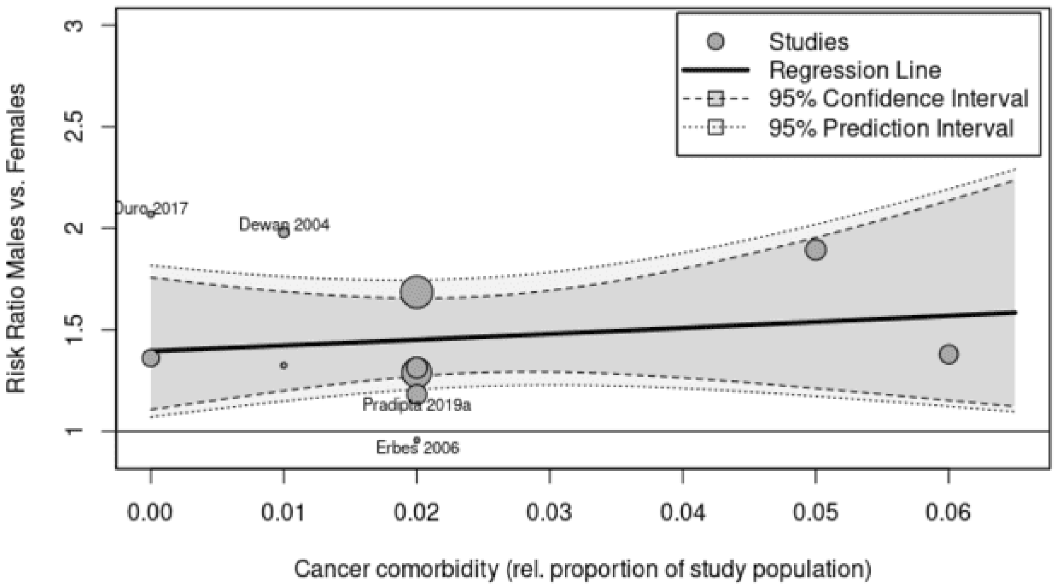

Figure A- 47: Bubble plot of moderator cancers

X. *Moderator diabetes*

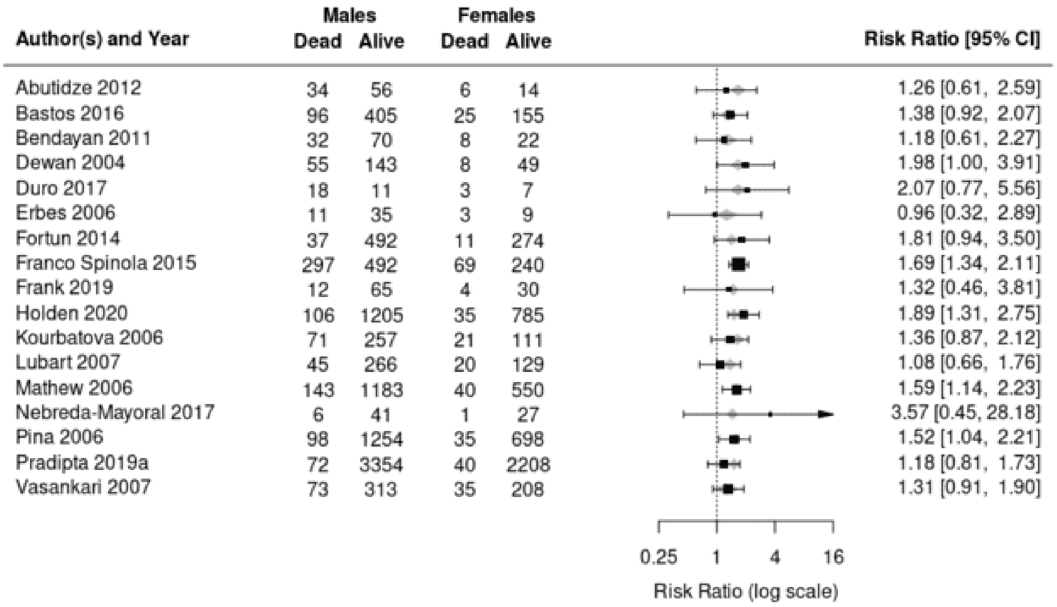

Figure A- 49: Forest plot of moderator diabetes

Moderator diabetes - Predicted pooled risk ratio (with 95% confidence/prediction intervals)

	pred	ci.lb	ci.ub	pi.lb	pi.ub
Abutidze 2012	1.66	1.41	1.95	1.41	1.95
Bastos 2016	1.31	1.09	1.59	1.09	1.59
Bendayan 2011	1.29	1.05	1.59	1.05	1.59
Dewan 2004	1.66	1.41	1.95	1.41	1.95
Duro 2017	1.66	1.41	1.95	1.41	1.95
Erbes 2006	1.26	1.01	1.59	1.01	1.59
Fortun 2014	1.42	1.25	1.61	1.25	1.61
Franco Spinola 2015	1.59	1.40	1.82	1.40	1.82
Frank 2019	1.48	1.32	1.65	1.32	1.65
Holden 2020	1.50	1.35	1.68	1.35	1.68
Kourbatova 2006	1.63	1.40	1.88	1.40	1.88
Lubart 2007	1.37	1.17	1.59	1.17	1.59
Mathew 2006	1.59	1.40	1.82	1.40	1.82
Nebreda-Mayoral 2017	1.45	1.29	1.63	1.29	1.63
Pina 2006	1.56	1.38	1.77	1.38	1.77
Pradipta 2019a	1.50	1.35	1.68	1.35	1.68
Vasankari 2007	1.24	0.97	1.59	0.97	1.59

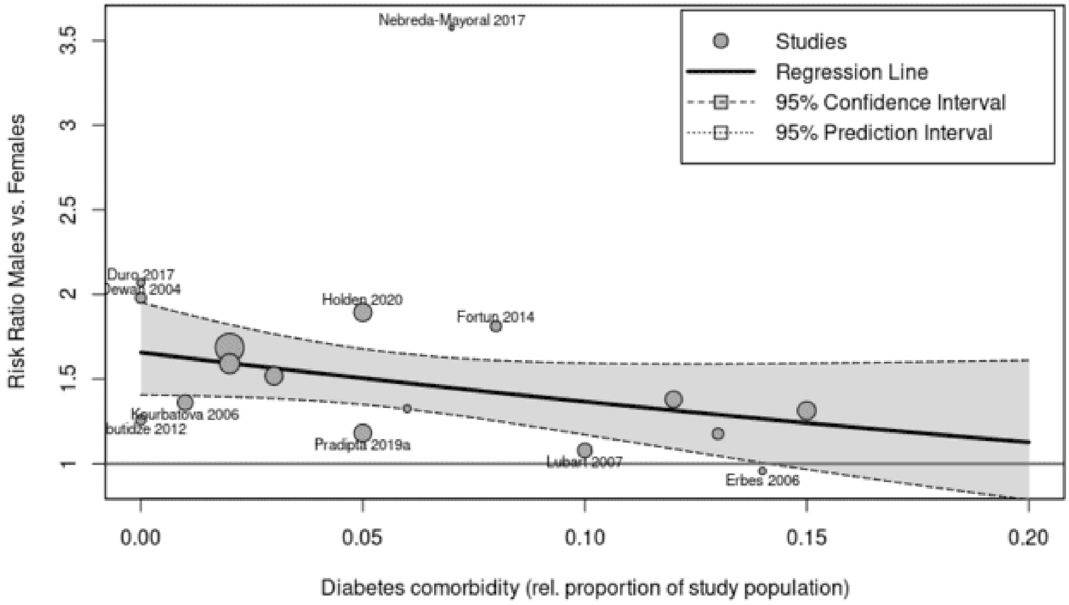

Figure A- 51: Bubble plot of moderator diabetes

XI. Moderator drugs

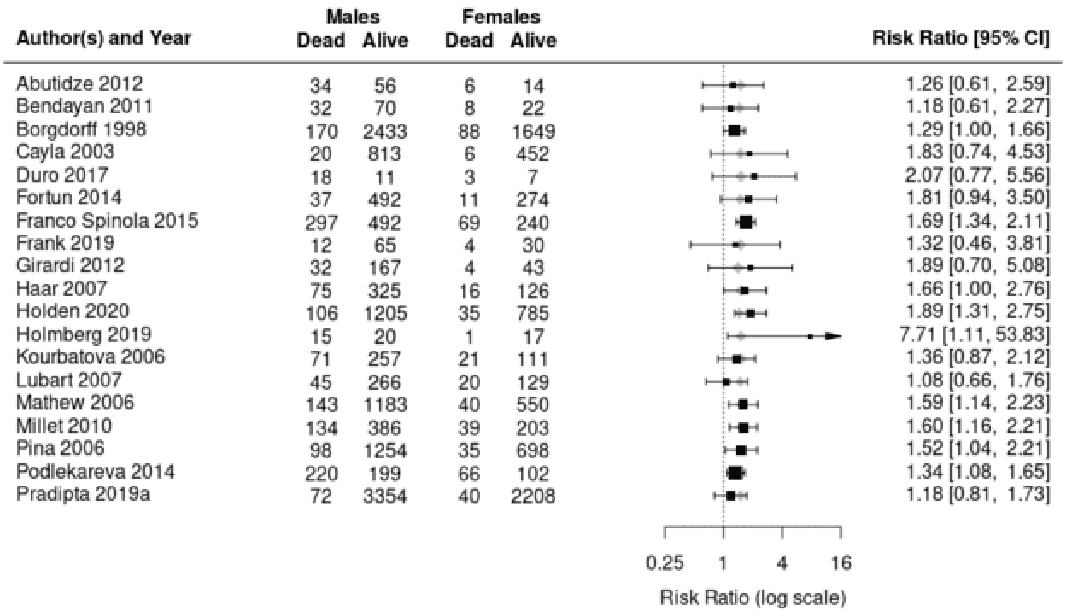

Figure A- 53: Forest plot of moderator drugs

Moderator drugs - Predicted pooled risk ratio (with 95% confidence/prediction intervals)

	pred	ci.lb	ci.ub	pi.lb	pi.ub
Abutidze 2012	1.50	1.35	1.67	1.35	1.67
Bendayan 2011	1.46	1.33	1.60	1.33	1.60
Borgdorff 1998	1.50	1.35	1.67	1.35	1.67
Cayla 2003	1.50	1.35	1.66	1.35	1.66
Duro 2017	1.51	1.35	1.68	1.35	1.68
Fortun 2014	1.49	1.35	1.63	1.35	1.63
Franco Spinola 2015	1.51	1.35	1.68	1.35	1.68
Frank 2019	1.50	1.35	1.67	1.35	1.67
Girardi 2012	1.41	1.23	1.62	1.23	1.62
Haar 2007	1.51	1.35	1.68	1.35	1.68
Holden 2020	1.46	1.33	1.60	1.33	1.60
Holmberg 2019	1.51	1.35	1.68	1.35	1.68
Kourbatova 2006	1.51	1.35	1.68	1.35	1.68
Lubart 2007	1.49	1.35	1.63	1.35	1.63
Mathew 2006	1.50	1.35	1.67	1.35	1.67
Millet 2010	1.49	1.35	1.65	1.35	1.65
Pina 2006	1.50	1.35	1.66	1.35	1.66
Podlekareva 2014	1.37	1.12	1.68	1.12	1.68
Pradipta 2019a	1.50	1.35	1.67	1.35	1.67

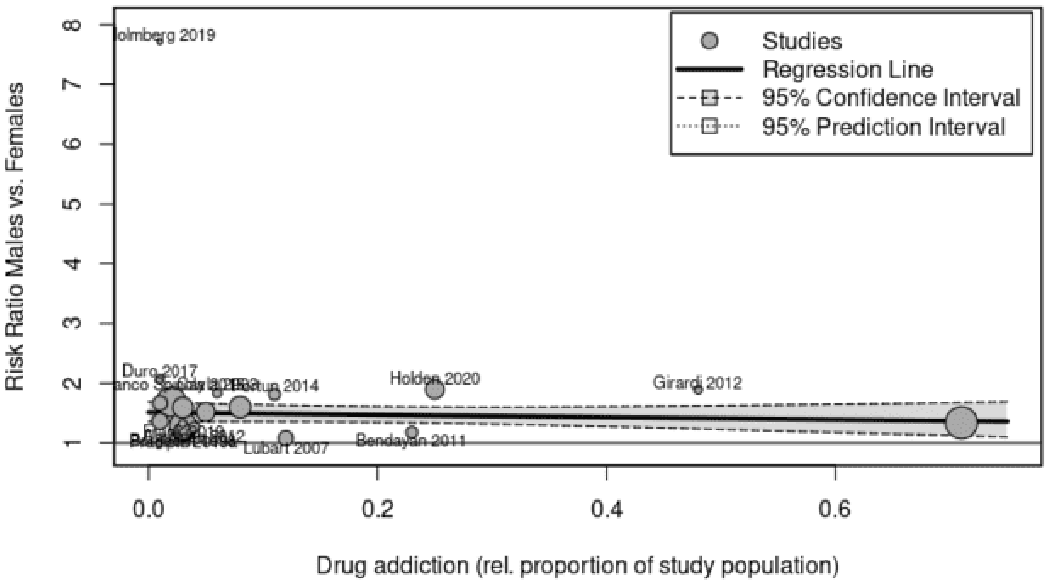

Figure A- 55: Bubble plot of moderator drugs

XII. Moderator extra-pulmonary TB (EPTB)

Author(s) and Year	Males Dead	Males Alive	Females Dead	Females Alive	Risk Ratio [95% CI]
Abutidze 2012	34	56	6	14	1.26 [0.61, 2.59]
Aguado 1997	10	20	6	15	1.17 [0.50, 2.71]
Borgdorff 1998	170	2433	88	1649	1.29 [1.00, 1.66]
Daucourt 2000	27	156	13	100	1.28 [0.69, 2.38]
Duro 2017	18	11	3	7	2.07 [0.77, 5.56]
Floe 2017	930	2820	581	2382	1.26 [1.15, 1.39]
Fortun 2014	37	492	11	274	1.81 [0.94, 3.50]
Franco Spinola 2015	297	492	69	240	1.69 [1.34, 2.11]
Frank 2019	12	65	4	30	1.32 [0.46, 3.81]
Gadoev 2015	3855	59869	2098	41558	1.26 [1.20, 1.33]
Girardi 2012	32	167	4	43	1.89 [0.70, 5.08]
Girardi 2014	142	3059	66	1891	1.32 [0.99, 1.75]
Haar 2007	75	325	16	126	1.66 [1.00, 2.76]
Holden 2020	106	1205	35	785	1.89 [1.31, 2.75]
Kherosheva 2003	28	546	3	172	2.85 [0.88, 9.25]
Korzeniewska-Kosela 2017	413	4044	113	1860	1.62 [1.32, 1.98]
Korzeniewska-Kosela 2018	420	4037	117	1870	1.60 [1.31, 1.95]
Korzeniewska-Kosela 2019	418	3708	125	1536	1.35 [1.11, 1.63]
Korzeniewska-Kosela 2020	383	3517	107	1480	1.46 [1.19, 1.79]
Kourbatova 2006	71	257	21	111	1.36 [0.87, 2.12]
Lowe 2013	29	379	13	297	1.69 [0.90, 3.21]
Loytved 2002	16	522	6	323	1.63 [0.64, 4.13]
Lubart 2007	45	266	20	129	1.08 [0.66, 1.76]
Majoor 2011	24	81	22	104	1.31 [0.78, 2.20]
Millet 2010	134	386	39	203	1.60 [1.16, 2.21]
Nebreda-Mayoral 2017	6	41	1	27	3.57 [0.45, 28.18]
Pedrazzoli 2019	4161	58467	2371	46775	1.38 [1.31, 1.45]
Pina 2006	98	1254	35	698	1.52 [1.04, 2.21]
Podlekareva 2014	220	199	66	102	1.34 [1.08, 1.65]
Podlekareva 2016	170	660	66	255	1.00 [0.77, 1.28]
Public Health England 2014a	300	4260	132	3200	1.66 [1.36, 2.03]
Public Health Ukraine 2017	22240	90150	4868	41249	1.87 [1.82, 1.93]
RKI 2014	97	2568	48	1589	1.24 [0.88, 1.74]
RKI 2017	65	3935	35	1870	0.88 [0.59, 1.33]
RKI 2019	85	3541	44	1751	0.96 [0.67, 1.37]
RKI 2020	82	3035	47	1618	0.93 [0.65, 1.33]
Rodriguez-Valin 2015	284	3406	96	2094	1.76 [1.40, 2.20]
Shuldiner 2014	208	2404	117	1808	1.31 [1.05, 1.63]
Shuldiner 2016	238	1551	147	1265	1.28 [1.05, 1.55]
Valade 2012	14	26	6	7	0.76 [0.37, 1.56]

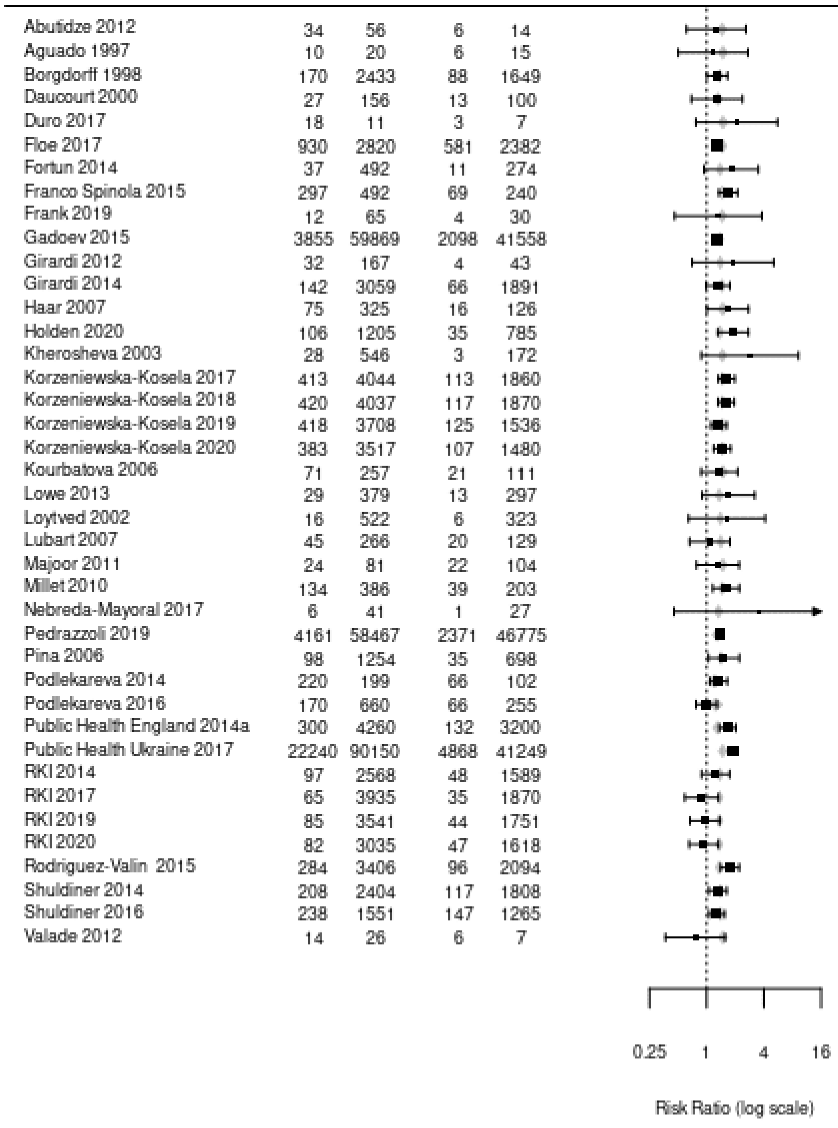

Figure A- 57: Forest plot of moderator extra-pulmonary TB

Moderator extra-pulmonary TB - Predicted pooled risk ratio (with 95% confidence/prediction intervals)

	pred	ci.lb	ci.ub	pi.lb	pi.ub
Abutidze 2012	1.46	1.34	1.58	1.11	1.91
Aguado 1997	1.48	1.35	1.63	1.13	1.95
Borgdorff 1998	1.37	1.28	1.47	1.05	1.79
Daucourt 2000	1.32	1.20	1.45	1.00	1.73
Duro 2017	1.48	1.35	1.63	1.13	1.95
Floe 2017	1.47	1.34	1.61	1.12	1.93
Fortun 2014	1.38	1.30	1.48	1.06	1.80
Franco Spinola 2015	1.46	1.34	1.59	1.12	1.91
Frank 2019	1.46	1.34	1.59	1.12	1.91
Gadoev 2015	1.38	1.29	1.48	1.06	1.80
Girardi 2012	1.39	1.31	1.49	1.07	1.82
Girardi 2014	1.45	1.34	1.57	1.11	1.90
Haar 2007	1.48	1.35	1.63	1.13	1.94
Holden 2020	1.39	1.30	1.48	1.07	1.81
Kherosheva 2003	1.47	1.34	1.60	1.12	1.92
Korzeniewska-Kosela 2017	1.46	1.34	1.59	1.12	1.91
Korzeniewska-Kosela 2018	1.46	1.34	1.59	1.12	1.91
Korzeniewska-Kosela 2019	1.47	1.34	1.60	1.12	1.92
Korzeniewska-Kosela 2020	1.47	1.34	1.60	1.12	1.92
Kourbatova 2006	1.48	1.35	1.63	1.13	1.95
Lowe 2013	1.35	1.26	1.46	1.04	1.77
Loytved 2002	1.39	1.31	1.49	1.07	1.82
Lubart 2007	1.41	1.32	1.51	1.08	1.84
Majoor 2011	1.48	1.35	1.63	1.13	1.94
Millet 2010	1.45	1.34	1.57	1.11	1.90
Nebreda-Mayoral 2017	1.33	1.22	1.45	1.01	1.74
Pedrazzoli 2019	1.29	1.15	1.45	0.98	1.71
Pina 2006	1.41	1.32	1.51	1.08	1.84
Podlekareva 2014	1.21	1.01	1.45	0.89	1.66
Podlekareva 2016	1.24	1.07	1.45	0.92	1.68
Public Health England 2014a	1.30	1.18	1.45	0.99	1.72
Public Health Ukraine 2017	1.47	1.35	1.62	1.12	1.94
RKI 2014	1.38	1.29	1.48	1.06	1.80
RKI 2017	1.37	1.28	1.47	1.05	1.79
RKI 2019	1.36	1.27	1.46	1.04	1.78
RKI 2020	1.36	1.26	1.46	1.04	1.77
Rodriguez-Valin 2015	1.35	1.26	1.46	1.04	1.77
Shuldiner 2014	1.40	1.31	1.49	1.07	1.82
Shuldiner 2016	1.40	1.31	1.50	1.08	1.83
Valade 2012	1.48	1.35	1.63	1.13	1.95

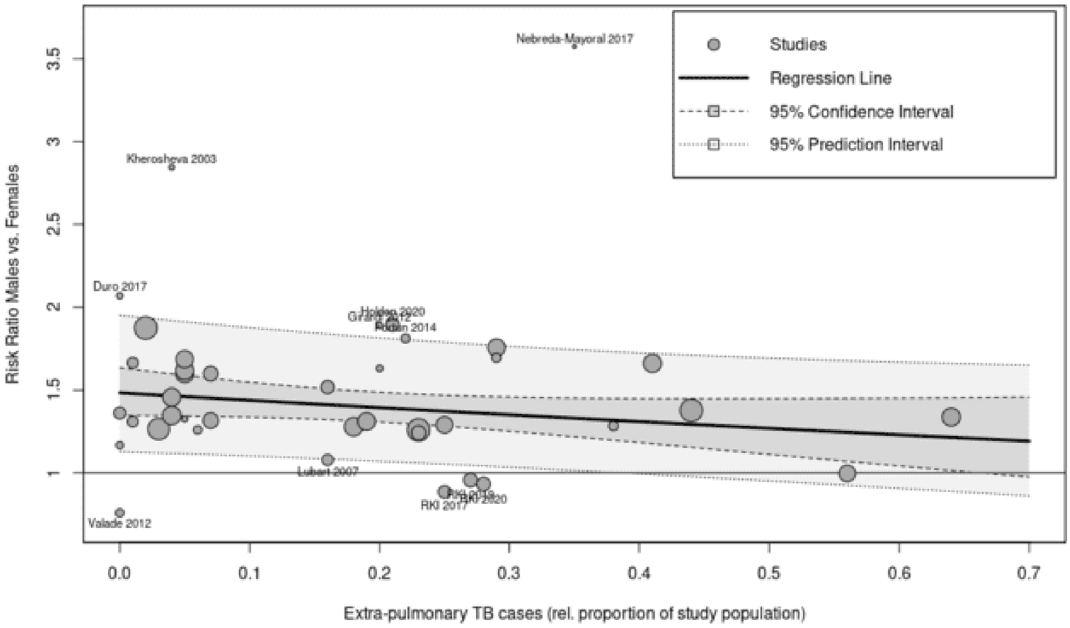

Figure A- 59: Bubble plot of moderator extra-pulmonary TB

XIII. *Moderator foreign origin*

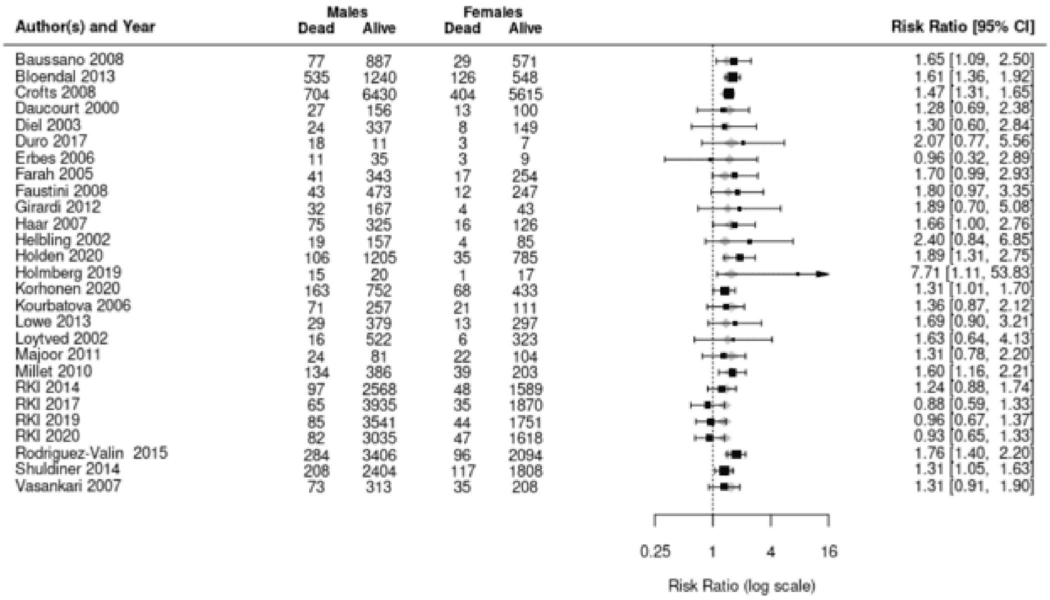

Figure A- 61: Forest plot of moderator foreign origin

Moderator foreign origin - Predicted pooled risk ratio (with 95% confidence/prediction intervals)

	pred	ci.lb	ci.ub	pi.lb	pi.ub
Baussano 2008	1.40	1.27	1.53	1.09	1.78
Bloendal 2013	1.40	1.27	1.53	1.09	1.78
Crofts 2008	1.35	1.19	1.53	1.05	1.75
Daucourt 2000	1.50	1.33	1.69	1.16	1.93
Diel 2003	1.41	1.29	1.54	1.10	1.79
Duro 2017	1.56	1.31	1.85	1.17	2.07
Erbes 2006	1.48	1.33	1.66	1.15	1.91
Farah 2005	1.33	1.16	1.53	1.02	1.74
Faustini 2008	1.45	1.32	1.59	1.13	1.85
Girardi 2012	1.43	1.31	1.56	1.12	1.82
Haar 2007	1.55	1.31	1.83	1.17	2.05
Helbling 2002	1.34	1.17	1.53	1.03	1.74
Holden 2020	1.37	1.23	1.53	1.07	1.76
Holmberg 2019	1.55	1.31	1.84	1.17	2.06
Korhonen 2020	1.45	1.32	1.60	1.14	1.86
Kourbatova 2006	1.56	1.31	1.85	1.17	2.07
Lowe 2013	1.37	1.23	1.53	1.07	1.76
Loytved 2002	1.43	1.30	1.56	1.12	1.82
Majoor 2011	1.55	1.31	1.84	1.17	2.06
Millet 2010	1.54	1.31	1.81	1.17	2.03
RKI 2014	1.39	1.26	1.53	1.09	1.78
RKI 2017	1.33	1.15	1.53	1.02	1.74
RKI 2019	1.31	1.12	1.54	1.00	1.73
RKI 2020	1.32	1.14	1.54	1.01	1.73
Rodriguez-Valin 2015	1.44	1.31	1.57	1.13	1.83
Shuldiner 2014	1.50	1.33	1.69	1.16	1.93
Vasankari 2007	1.54	1.31	1.81	1.17	2.03

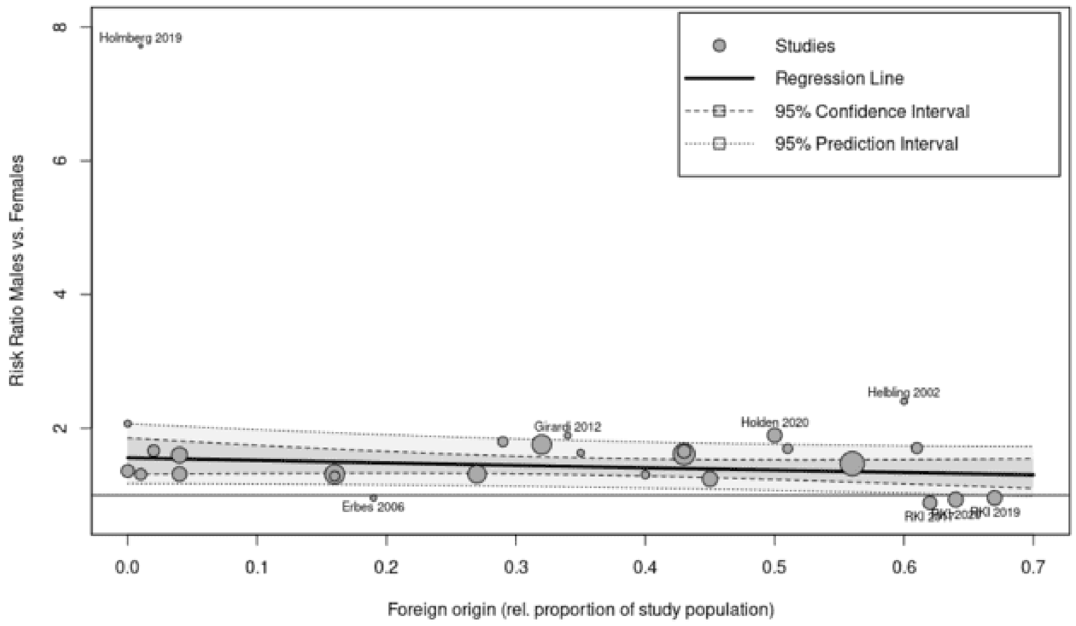

Figure A- 63: Bubble plot of moderator foreign origin

XIV. *Moderator former TB*

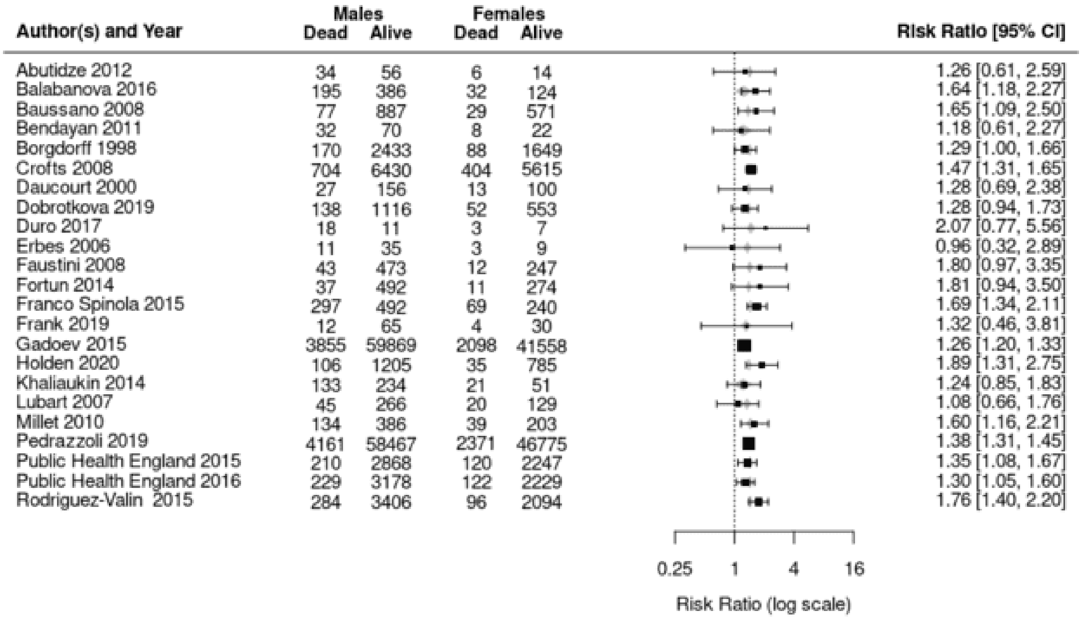

Figure A- 65: Forest plot of moderator former TB

Moderator former TB - Predicted pooled risk ratio (with 95% confidence/prediction intervals)

	pred	ci.lb	ci.ub	pi.lb	pi.ub
Abutidze 2012	1.43	1.34	1.52	1.26	1.62
Balabanova 2016	1.24	1.03	1.49	1.00	1.53
Baussano 2008	1.37	1.29	1.46	1.21	1.56
Bendayan 2011	1.22	1.00	1.49	0.97	1.54
Borgdorff 1998	1.42	1.34	1.51	1.25	1.62
Crofts 2008	1.43	1.34	1.53	1.26	1.63
Daucourt 2000	1.39	1.31	1.47	1.22	1.58
Dobrotkova 2019	1.39	1.32	1.48	1.23	1.58
Duro 2017	1.46	1.35	1.59	1.27	1.68
Erbes 2006	1.35	1.25	1.46	1.18	1.55
Faustini 2008	1.42	1.34	1.51	1.25	1.62
Fortun 2014	1.39	1.32	1.48	1.23	1.58
Franco Spinola 2015	1.46	1.35	1.57	1.27	1.67
Frank 2019	1.31	1.17	1.46	1.12	1.53
Gadoev 2015	1.35	1.26	1.46	1.18	1.55
Holden 2020	1.40	1.32	1.48	1.24	1.59
Khaliaukin 2014	1.22	0.99	1.49	0.97	1.54
Lubart 2007	1.33	1.22	1.46	1.16	1.54
Millet 2010	1.46	1.35	1.57	1.27	1.67
Pedrazzoli 2019	1.43	1.34	1.53	1.26	1.63
Public Health England 2015	1.44	1.34	1.54	1.26	1.64
Public Health England 2016	1.44	1.34	1.54	1.26	1.64
Rodriguez-Valin 2015	1.44	1.35	1.55	1.26	1.65

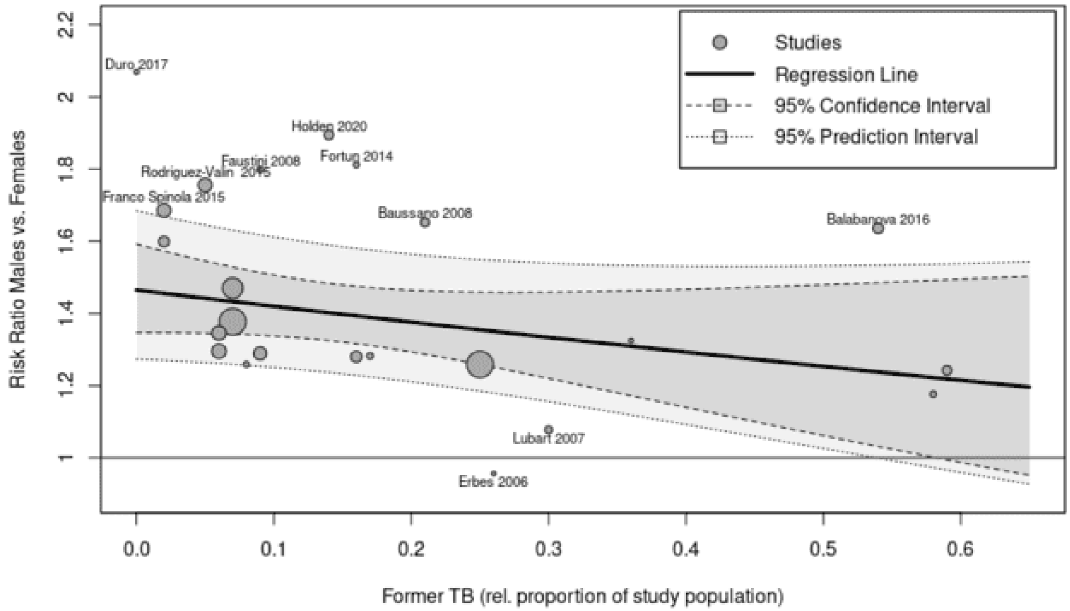

Figure A- 67: Bubble plot of moderator former TB

XV. *Moderator hepatitides/cirrhosis*

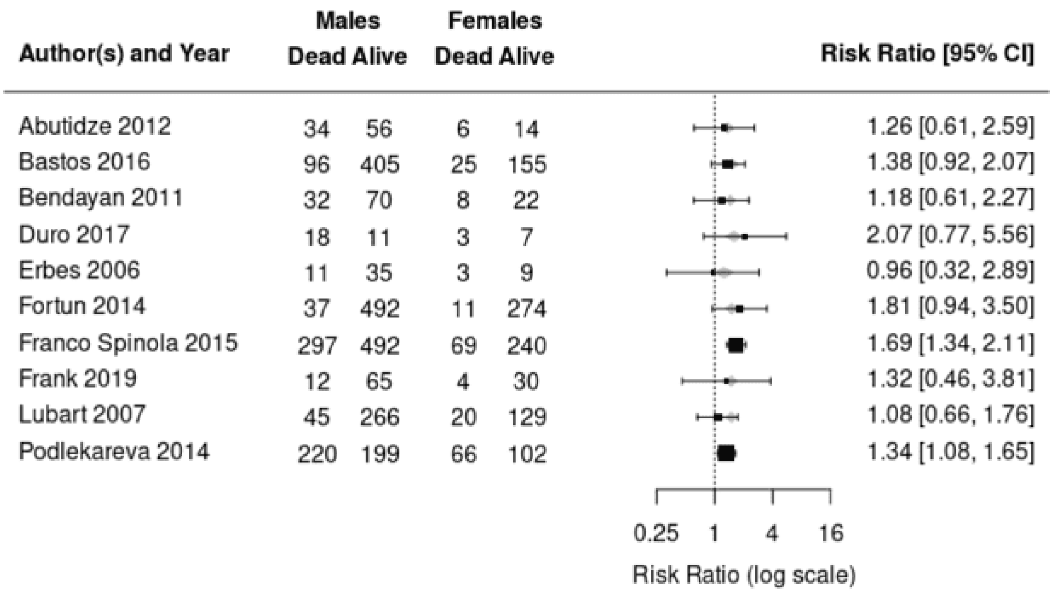

Figure A- 69: Forest plot of moderator hepatitides/cirrhosis

Moderator hepatitides/cirrhosis - Predicted pooled risk ratio (with 95% confidence/prediction intervals)

	pred	ci.lb	ci.ub	pi.lb	pi.ub
Abutidze 2012	1.37	1.19	1.58	1.19	1.58
Bastos 2016	1.51	1.31	1.75	1.31	1.75
Bendayan 2011	1.45	1.28	1.65	1.28	1.65
Duro 2017	1.59	1.31	1.92	1.31	1.92
Erbes 2006	1.25	1.00	1.56	1.00	1.56
Fortun 2014	1.50	1.30	1.72	1.30	1.72
Franco Spinola 2015	1.58	1.31	1.91	1.31	1.91
Frank 2019	1.49	1.30	1.71	1.30	1.71
Lubart 2007	1.50	1.30	1.72	1.30	1.72
Podlekareva 2014	1.28	1.05	1.56	1.05	1.56

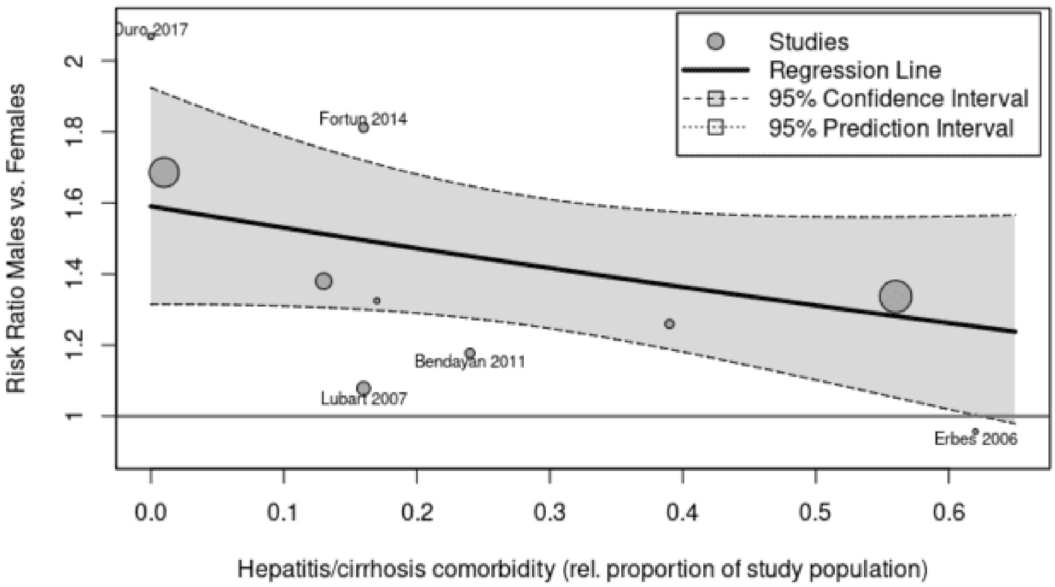

Figure A- 71: Bubble plot of moderator hepatitides/cirrhosis

XVI. *Moderator HIV/AIDS*

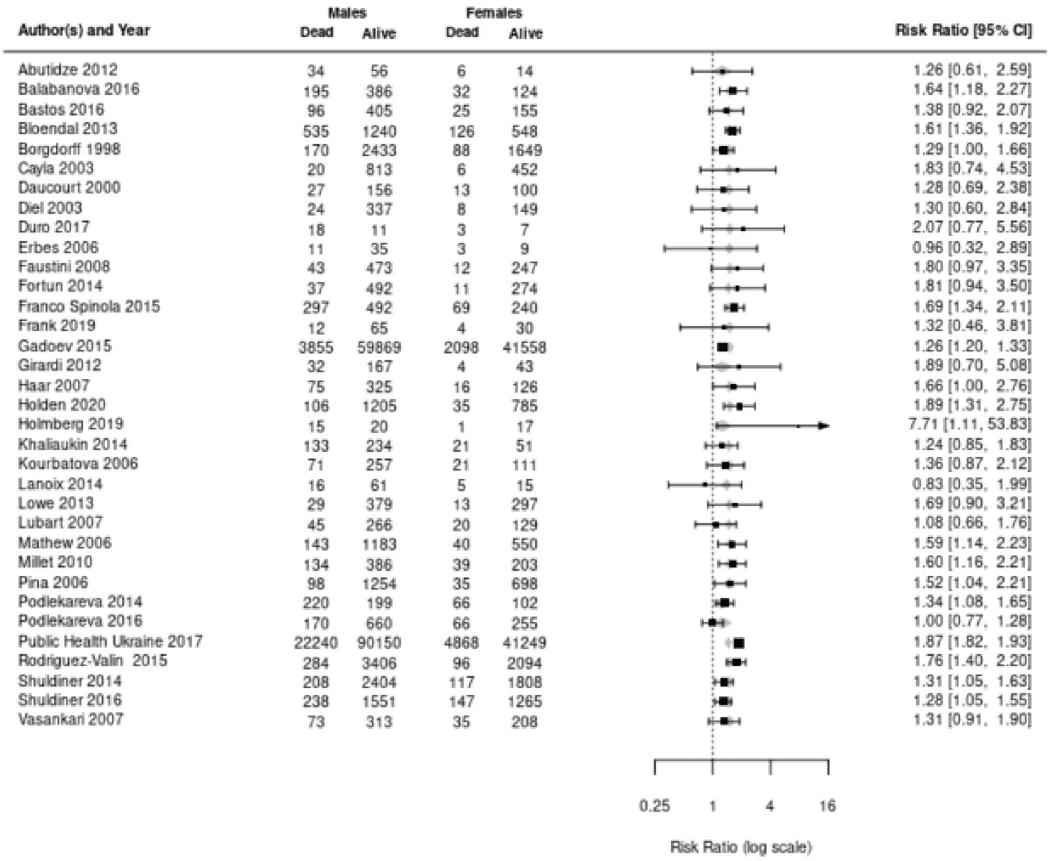

Figure A- 73: Forest plot of moderator HIV/AIDS

Moderator HIV/AIDS - Predicted pooled risk ratio (with 95% confidence/prediction intervals)

	pred	ci.lb	ci.ub	pi.lb	pi.ub
Abutidze 2012	1.25	1.00	1.56	0.90	1.75
Balabanova 2016	1.50	1.38	1.63	1.15	1.96
Bastos 2016	1.46	1.35	1.58	1.12	1.90
Bloendal 2013	1.49	1.38	1.62	1.14	1.95
Borgdorff 1998	1.50	1.38	1.63	1.15	1.96
Cayla 2003	1.49	1.37	1.62	1.14	1.94
Daucourt 2000	1.48	1.37	1.60	1.14	1.93
Diel 2003	1.49	1.37	1.62	1.14	1.94
Duro 2017	1.50	1.38	1.64	1.15	1.97
Erbes 2006	1.49	1.37	1.61	1.14	1.94
Faustini 2008	1.50	1.38	1.64	1.15	1.96
Fortun 2014	1.46	1.35	1.57	1.12	1.89
Franco Spinola 2015	1.50	1.38	1.63	1.15	1.96
Frank 2019	1.50	1.38	1.63	1.15	1.96
Gadoev 2015	1.50	1.38	1.64	1.15	1.97
Girardi 2012	1.25	1.00	1.56	0.90	1.75
Haar 2007	1.50	1.38	1.63	1.15	1.95

Holden 2020	1.50	1.38	1.63	1.15	1.96
Holmberg 2019	1.25	1.00	1.56	0.90	1.75
Khaliaukin 2014	1.47	1.36	1.59	1.13	1.92
Kourbatova 2006	1.50	1.38	1.64	1.15	1.97
Lanoix 2014	1.40	1.27	1.54	1.07	1.83
Lowe 2013	1.49	1.37	1.61	1.14	1.94
Lubart 2007	1.48	1.37	1.60	1.13	1.92
Mathew 2006	1.51	1.38	1.65	1.15	1.97
Millet 2010	1.48	1.37	1.60	1.13	1.92
Pina 2006	1.49	1.37	1.62	1.14	1.94
Podlekareva 2014	1.25	1.00	1.56	0.90	1.75
Podlekareva 2016	1.25	1.00	1.56	0.90	1.75
Public Health Ukraine 2017	1.46	1.35	1.58	1.12	1.90
Rodriguez-Valin 2015	1.49	1.37	1.62	1.14	1.94
Shuldiner 2014	1.49	1.38	1.62	1.14	1.95
Shuldiner 2016	1.50	1.38	1.63	1.15	1.95
Vasankari 2007	1.51	1.38	1.65	1.15	1.97

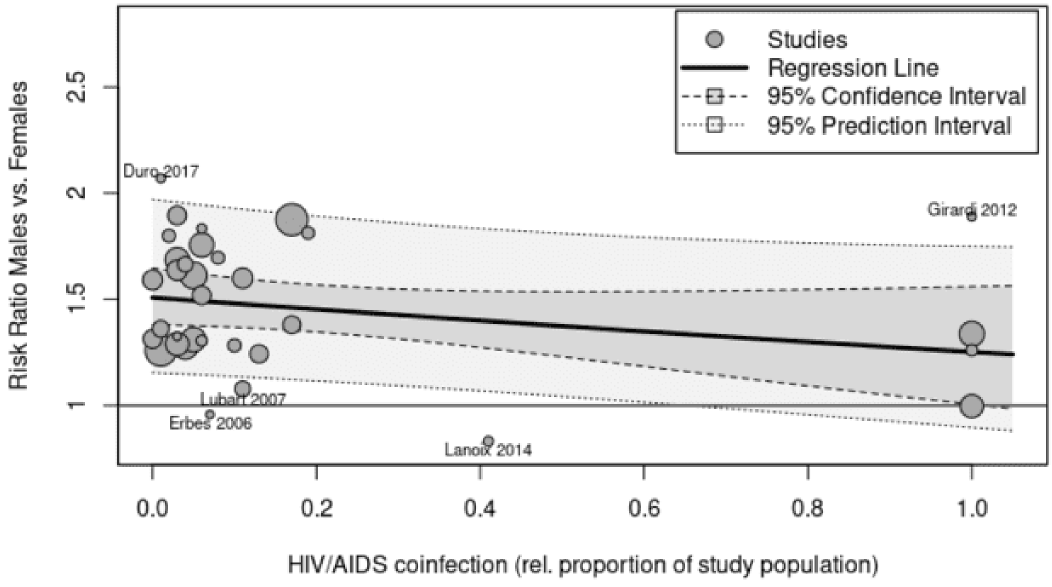

Figure A- 75: Bubble plot of moderator HIV/AIDS

XVII. *Moderator multi-drug resistant TB (MDR TB)*

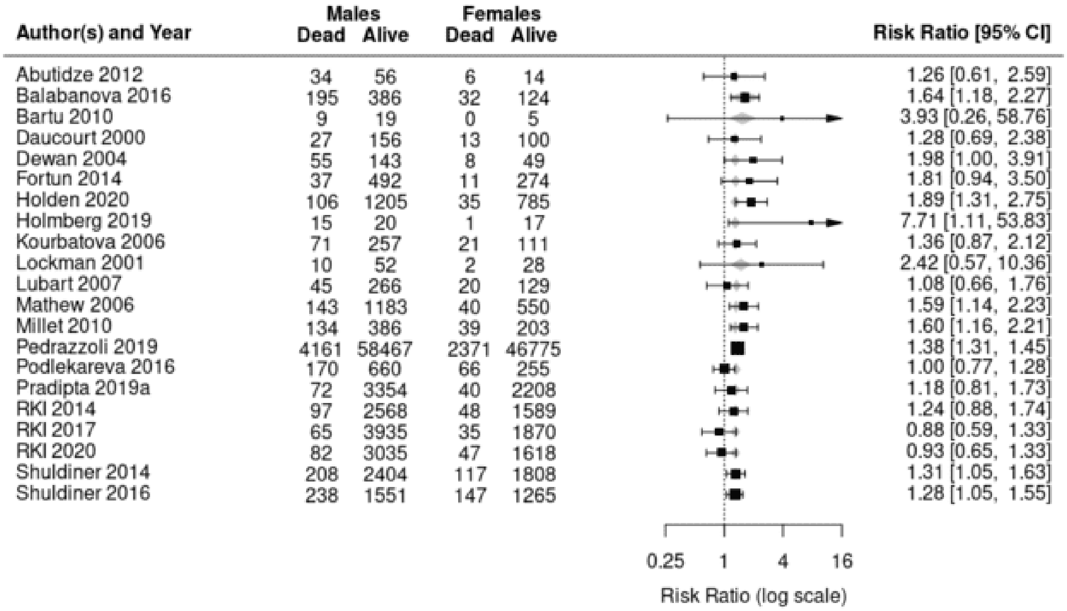

Figure A- 77: Forest plot of moderator multi-drug resistant TB

Moderator multi-drug resistant TB - Predicted pooled risk ratio (with 95% confidence/prediction intervals)

	pred	ci.lb	ci.ub	pi.lb	pi.ub
Abutidze 2012	1.31	1.19	1.44	1.04	1.63
Balabanova 2016	1.62	1.11	2.36	1.05	2.48
Bartu 2010	1.54	1.15	2.06	1.08	2.19
Daucourt 2000	1.30	1.18	1.43	1.04	1.63
Dewan 2004	1.30	1.18	1.43	1.04	1.63
Fortun 2014	1.31	1.19	1.44	1.05	1.64
Holden 2020	1.30	1.18	1.43	1.04	1.63
Holmberg 2019	1.30	1.18	1.43	1.03	1.63
Kourbatova 2006	1.30	1.18	1.43	1.04	1.63
Lockman 2001	1.48	1.19	1.84	1.10	1.99
Lubart 2007	1.34	1.22	1.47	1.07	1.67
Mathew 2006	1.32	1.21	1.45	1.06	1.65
Millet 2010	1.30	1.18	1.43	1.04	1.63
Pedrazzoli 2019	1.30	1.18	1.43	1.04	1.63
Podlekareva 2016	1.33	1.21	1.45	1.06	1.66
Pradipta 2019a	1.30	1.19	1.43	1.04	1.63
RKI 2014	1.30	1.19	1.43	1.04	1.63
RKI 2017	1.30	1.19	1.43	1.04	1.63
RKI 2020	1.30	1.19	1.43	1.04	1.63
Shuldiner 2014	1.31	1.19	1.44	1.05	1.64
Shuldiner 2016	1.31	1.19	1.44	1.05	1.64

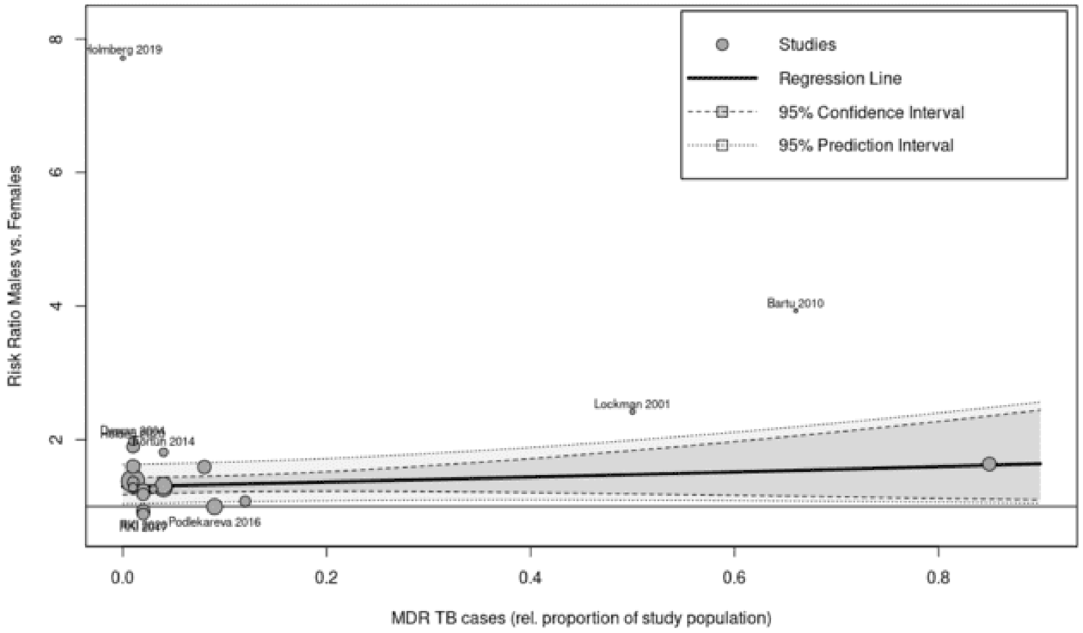

Figure A- 79: Bubble plot of moderator multi-drug resistant TB

XVIII. Moderator migrant

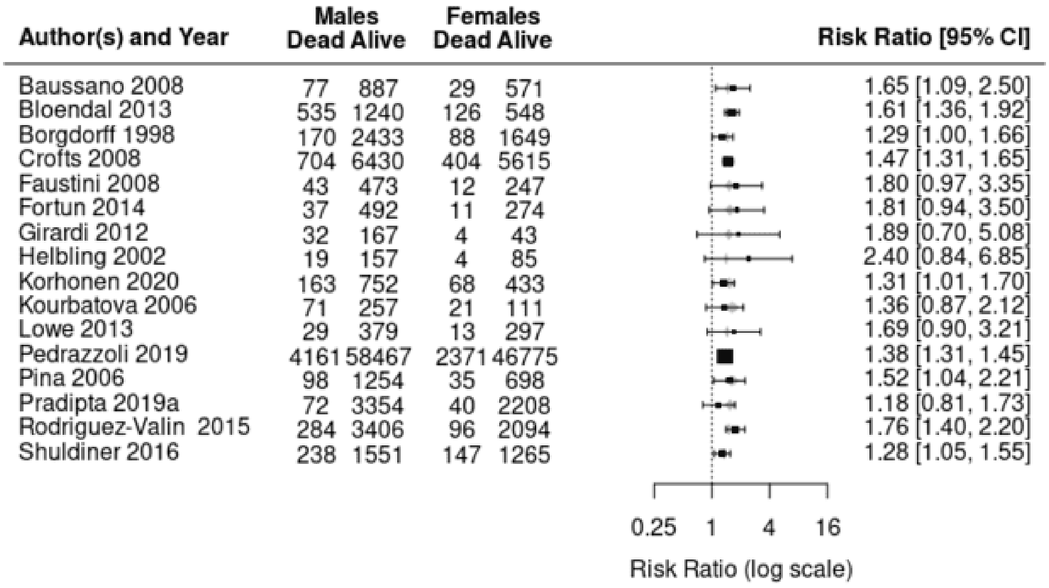

Figure A- 81: Forest plot of moderator migrant

Moderator migrant - Predicted pooled risk ratio (with 95% confidence/prediction intervals)

	pred	ci.lb	ci.ub	pi.lb	pi.ub
Baussano 2008	1.47	1.38	1.56	1.38	1.56
Bloendal 2013	1.47	1.38	1.56	1.38	1.56
Borgdorff 1998	1.45	1.38	1.52	1.38	1.52
Crofts 2008	1.42	1.36	1.48	1.36	1.48
Faustini 2008	1.51	1.38	1.66	1.38	1.66
Fortun 2014	1.54	1.38	1.71	1.38	1.71
Girardi 2012	1.50	1.38	1.62	1.38	1.62
Helbling 2002	1.41	1.35	1.46	1.35	1.46
Korhonen 2020	1.52	1.38	1.68	1.38	1.68
Kourbatova 2006	1.62	1.37	1.92	1.37	1.92
Lowe 2013	1.44	1.37	1.51	1.37	1.51
Pedrazzoli 2019	1.39	1.33	1.45	1.33	1.45
Pina 2006	1.61	1.37	1.89	1.37	1.89
Pradipta 2019a	1.54	1.38	1.73	1.38	1.73
Rodriguez-Valin 2015	1.50	1.38	1.64	1.38	1.64
Shuldiner 2016	1.34	1.24	1.44	1.24	1.44

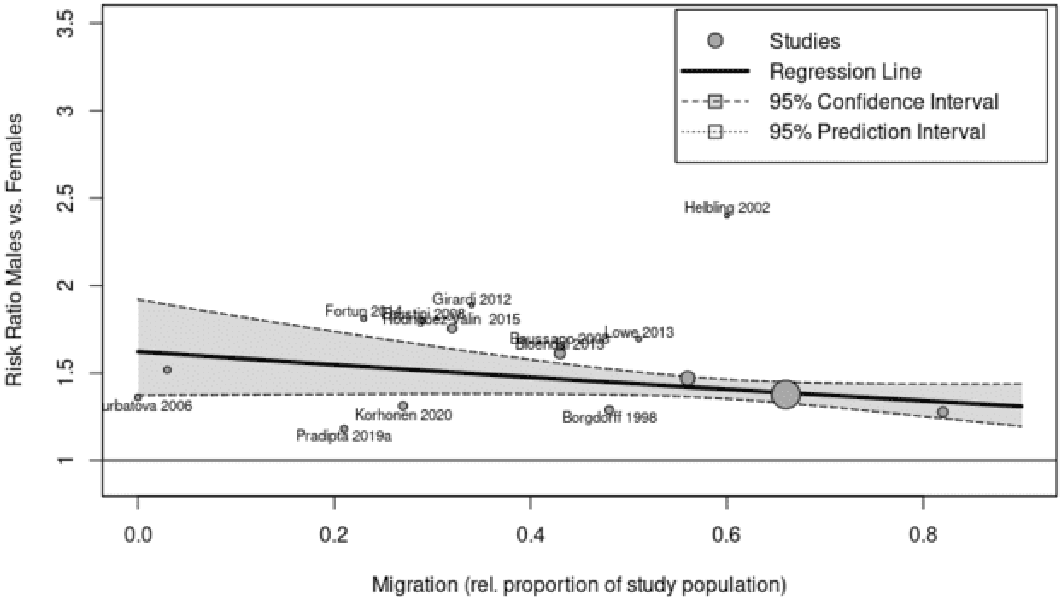

Figure A- 83: Bubble plot of moderator migrant

XIX. Moderator mixed TB

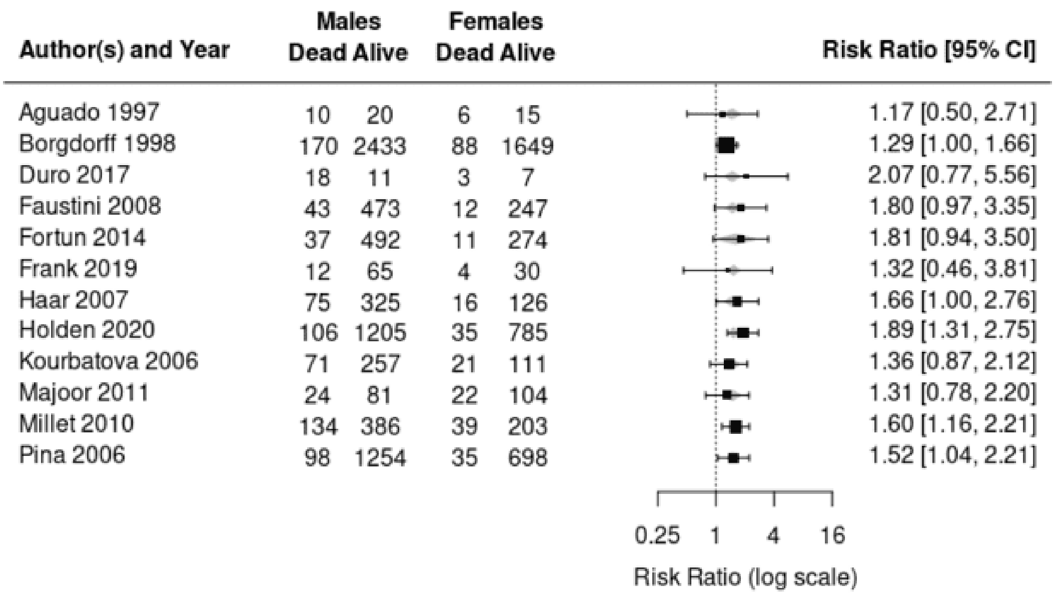

Figure A- 85: Forest plot of moderator mixed TB

Moderator mixed TB - Predicted pooled risk ratio (with 95% confidence/prediction intervals)

	pred	ci.lb	ci.ub	pi.lb	pi.ub
Aguado 1997	1.47	1.19	1.81	1.19	1.81
Borgdorff 1998	1.52	1.31	1.77	1.31	1.77
Duro 2017	1.47	1.23	1.77	1.23	1.77
Faustini 2008	1.48	1.26	1.74	1.26	1.74
Fortun 2014	1.61	0.99	2.62	0.99	2.62
Frank 2019	1.52	1.31	1.77	1.31	1.77
Haar 2007	1.47	1.23	1.77	1.23	1.77
Holden 2020	1.51	1.32	1.73	1.32	1.73
Kourbatova 2006	1.47	1.23	1.77	1.23	1.77
Majoor 2011	1.47	1.19	1.81	1.19	1.81
Millet 2010	1.51	1.32	1.71	1.32	1.71
Pina 2006	1.49	1.29	1.72	1.29	1.72

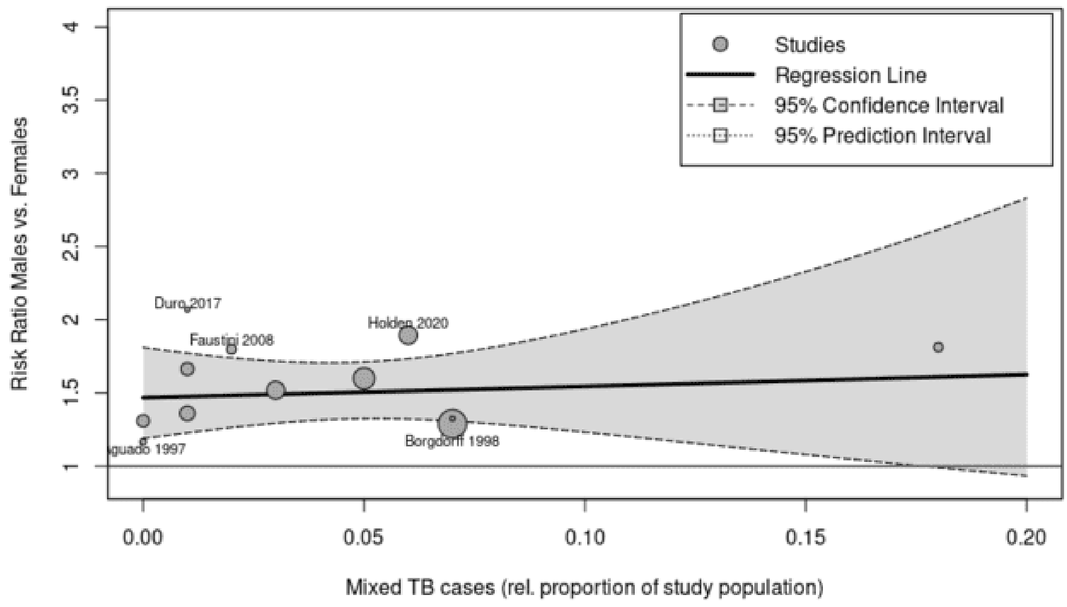

Figure A- 87: Bubble plot of moderator mixed TB

XX. *Moderator mono-drug resistant TB*

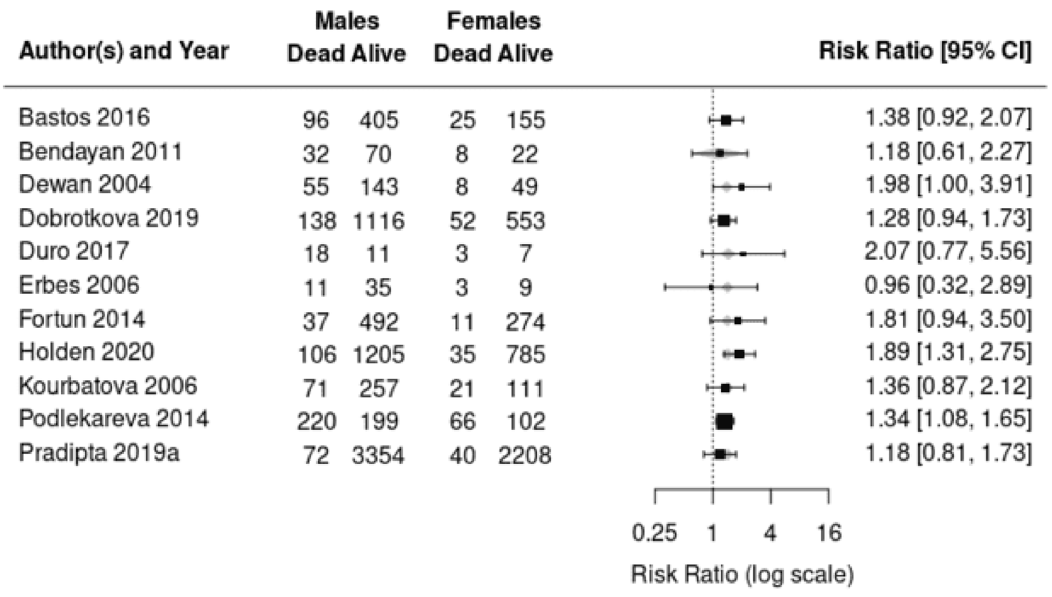

Figure A- 89: Forest plot of moderator mono-drug resistant TB

Moderator mono-drug resistant TB - Predicted pooled risk ratio (with 95% confidence/prediction intervals)

	pred	ci.lb	ci.ub	pi.lb	pi.ub
Bastos 2016	1.42	1.24	1.61	1.24	1.61
Bendayan 2011	1.14	0.62	2.10	0.62	2.10
Dewan 2004	1.39	1.23	1.57	1.23	1.57
Dobrotkova 2019	1.43	1.24	1.65	1.24	1.65
Duro 2017	1.44	1.23	1.69	1.23	1.69
Erbes 2006	1.42	1.24	1.61	1.24	1.61
Fortun 2014	1.41	1.24	1.60	1.24	1.60
Holden 2020	1.43	1.24	1.64	1.24	1.64
Kourbatova 2006	1.42	1.24	1.62	1.24	1.62
Podlekareva 2014	1.38	1.21	1.57	1.21	1.57
Pradipta 2019a	1.42	1.24	1.61	1.24	1.61

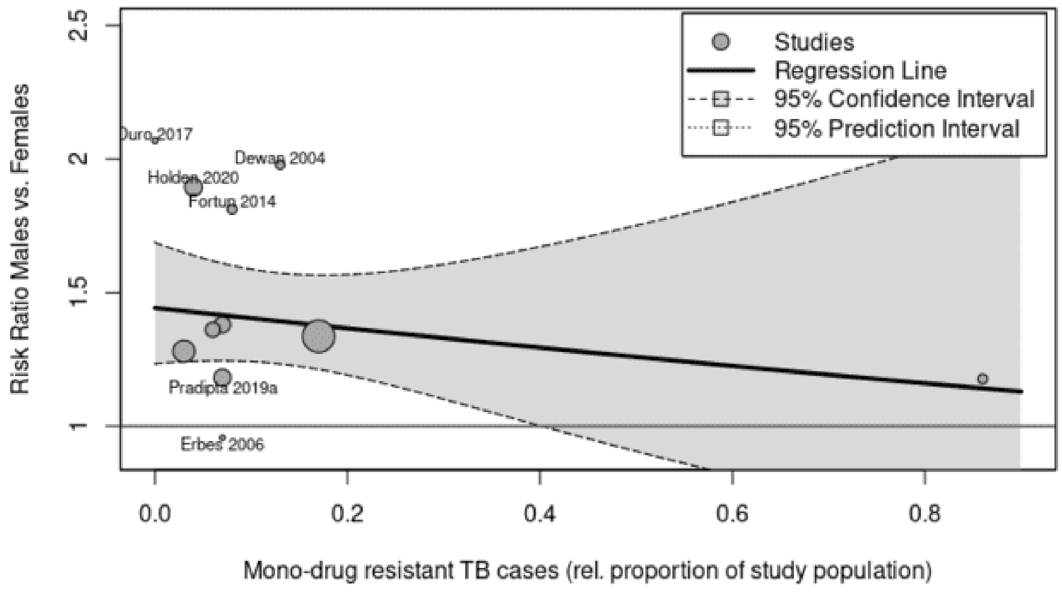

Figure A- 91: Bubble plot of moderator mono-drug resistant TB

XXI. Moderator new (incident/first-time) cases

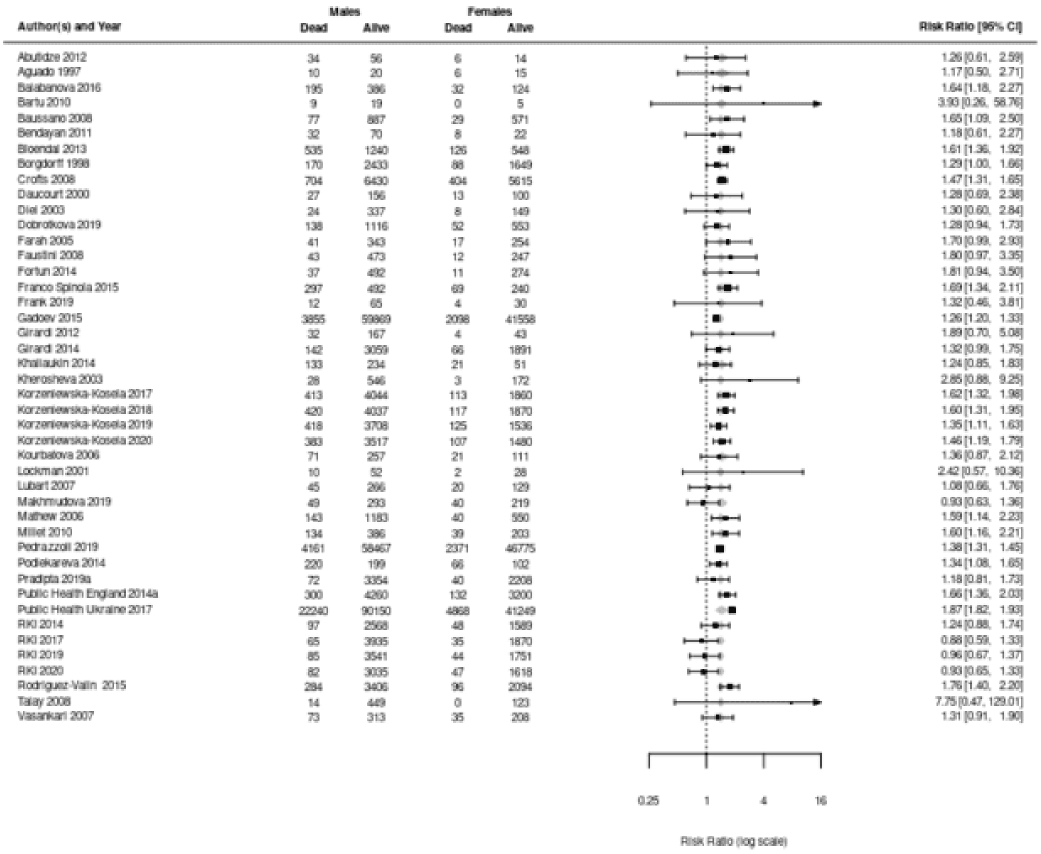

Figure A- 93: Forest plot of moderator new (incident/first-time) cases

Moderator new (incident/first-time) cases - Predicted pooled risk ratio (with 95% confidence/prediction intervals)

	pred	ci.lb	ci.ub	pi.lb	pi.ub
Abutidze 2012	1.45	1.26	1.66	1.10	1.92
Aguado 1997	1.46	1.22	1.73	1.08	1.97
Balabanova 2016	1.44	1.32	1.56	1.11	1.86
Bartu 2010	1.44	1.29	1.61	1.10	1.89
Baussano 2008	1.42	1.33	1.52	1.10	1.83
Bendayan 2011	1.44	1.31	1.58	1.11	1.87
Bloendal 2013	1.41	1.29	1.54	1.09	1.83
Borgdorff 1998	1.42	1.32	1.52	1.10	1.83
Crofts 2008	1.42	1.34	1.52	1.11	1.83
Daucourt 2000	1.42	1.33	1.52	1.10	1.83
Diel 2003	1.42	1.32	1.52	1.10	1.83
Dobrotkova 2019	1.42	1.33	1.52	1.10	1.83
Farah 2005	1.41	1.29	1.54	1.09	1.83
Faustini 2008	1.43	1.34	1.52	1.11	1.84

Fortun 2014	1.42	1.33	1.52	1.10	1.83
Franco Spinola 2015	1.45	1.27	1.66	1.10	1.91
Frank 2019	1.43	1.33	1.55	1.11	1.85
Gadoev 2015	1.42	1.34	1.51	1.11	1.83
Girardi 2012	1.42	1.33	1.52	1.10	1.83
Girardi 2014	1.41	1.29	1.54	1.09	1.83
Khaliaukin 2014	1.45	1.28	1.63	1.10	1.90
Kherosheva 2003	1.42	1.31	1.53	1.10	1.83
Korzeniewska-Kosela 2017	1.42	1.31	1.53	1.10	1.83
Korzeniewska-Kosela 2018	1.42	1.32	1.53	1.10	1.83
Korzeniewska-Kosela 2019	1.42	1.32	1.53	1.10	1.83
Korzeniewska-Kosela 2020	1.42	1.32	1.52	1.10	1.83
Kourbatova 2006	1.45	1.25	1.68	1.09	1.93
Lockman 2001	1.41	1.29	1.54	1.09	1.83
Lubart 2007	1.43	1.34	1.52	1.11	1.83
Makhmudova 2019	1.45	1.28	1.63	1.10	1.90
Mathew 2006	1.42	1.32	1.52	1.10	1.83
Millet 2010	1.44	1.31	1.57	1.11	1.87
Pedrazzoli 2019	1.42	1.31	1.53	1.10	1.83
Podlekareva 2014	1.42	1.32	1.52	1.10	1.83
Pradipta 2019a	1.42	1.31	1.53	1.10	1.83
Public Health England 2014a	1.42	1.32	1.53	1.10	1.83
Public Health Ukraine 2017	1.45	1.26	1.68	1.09	1.93
RKI 2014	1.42	1.34	1.51	1.11	1.83
RKI 2017	1.43	1.34	1.53	1.11	1.84
RKI 2019	1.43	1.34	1.52	1.11	1.84
RKI 2020	1.43	1.34	1.52	1.11	1.84
Rodriguez-Valin 2015	1.42	1.31	1.53	1.10	1.83
Talay 2008	1.42	1.34	1.51	1.11	1.83
Vasankari 2007	1.42	1.31	1.53	1.10	1.83

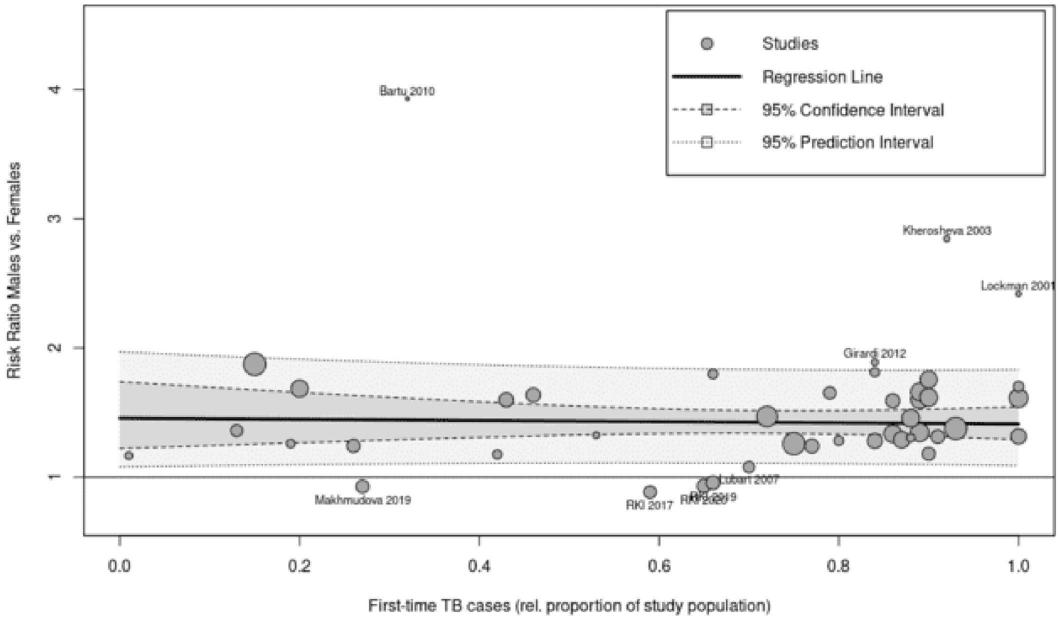

Figure A- 95: Bubble plot of moderator new (incident/first-time) cases

XXII. Moderator homelessness

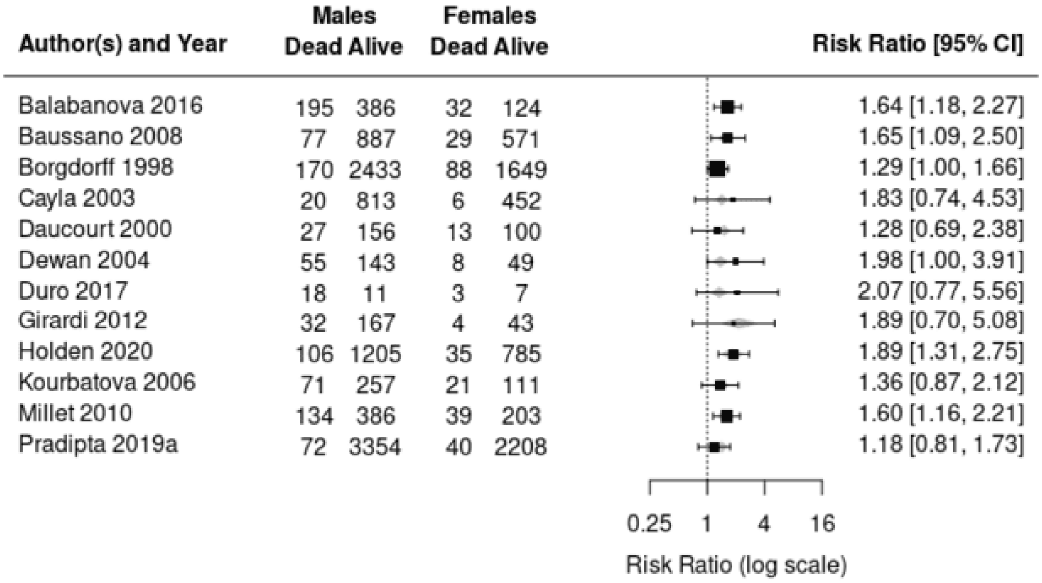

Figure A- 97: Forest plot of moderator homelessness

Moderator homelessness - Predicted pooled risk ratio (with 95% confidence/prediction intervals)

	pred	ci.lb	ci.ub	pi.lb	pi.ub
Balabanova 2016	1.66	1.40	1.97	1.40	1.97
Baussano 2008	1.51	1.34	1.71	1.34	1.71
Borgdorff 1998	1.37	1.17	1.61	1.17	1.61
Cayla 2003	1.42	1.23	1.63	1.23	1.63
Daucourt 2000	1.51	1.34	1.71	1.34	1.71
Dewan 2004	1.37	1.17	1.61	1.17	1.61
Duro 2017	1.33	1.10	1.61	1.10	1.61
Girardi 2012	2.14	1.38	3.32	1.38	3.32
Holden 2020	1.83	1.41	2.38	1.41	2.38
Kourbatova 2006	1.42	1.23	1.63	1.23	1.63
Millet 2010	1.46	1.29	1.66	1.29	1.66
Pradipta 2019a	1.42	1.23	1.63	1.23	1.63

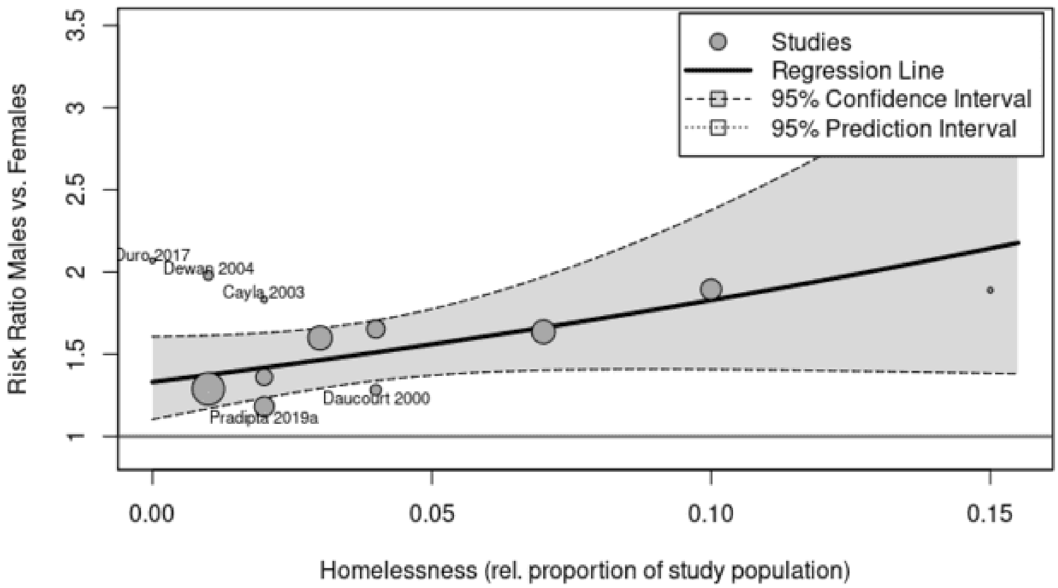

Figure A- 99: Bubble plot of moderator homelessnes

XXIII. Moderator other comorbidities

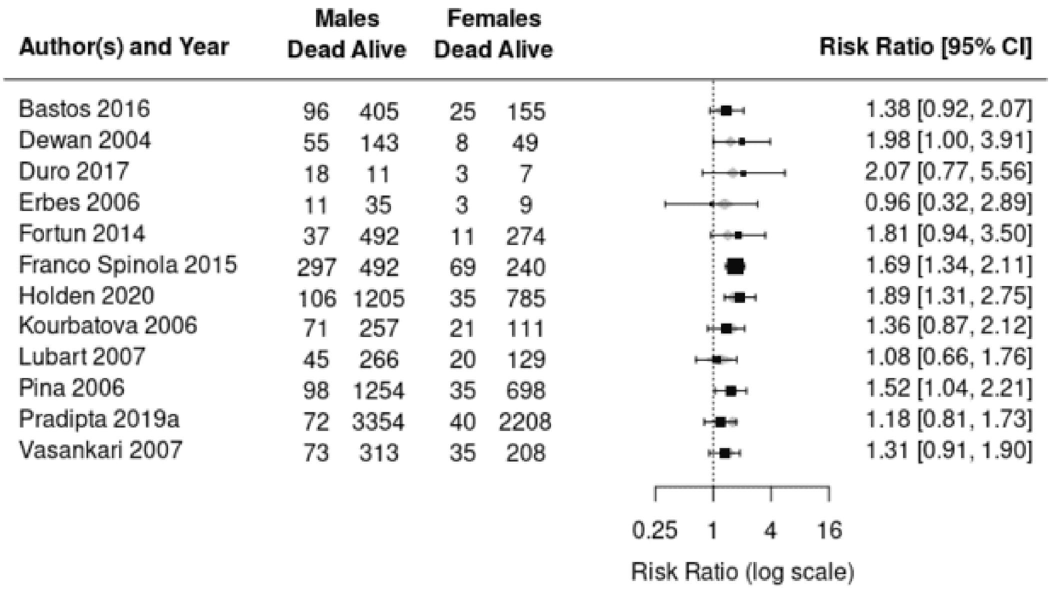

Figure A- 101: Forest plot of moderator other comorbidities

Moderator other comorbidities - Predicted pooled risk ratio (with 95% confidence/prediction intervals)

	pred	ci.lb	ci.ub	pi.lb	pi.ub
Bastos 2016	1.28	1.00	1.63	1.00	1.63
Dewan 2004	1.51	1.34	1.70	1.34	1.70
Duro 2017	1.60	1.38	1.85	1.38	1.85
Erbes 2006	1.31	1.06	1.62	1.06	1.62
Fortun 2014	1.42	1.23	1.63	1.23	1.63
Franco Spinola 2015	1.57	1.37	1.79	1.37	1.79
Holden 2020	1.59	1.38	1.83	1.38	1.83
Kourbatova 2006	1.58	1.38	1.81	1.38	1.81
Lubart 2007	1.16	0.81	1.66	0.81	1.66
Pina 2006	1.59	1.38	1.83	1.38	1.83
Pradipta 2019a	1.59	1.38	1.83	1.38	1.83
Vasankari 2007	1.48	1.31	1.67	1.31	1.67

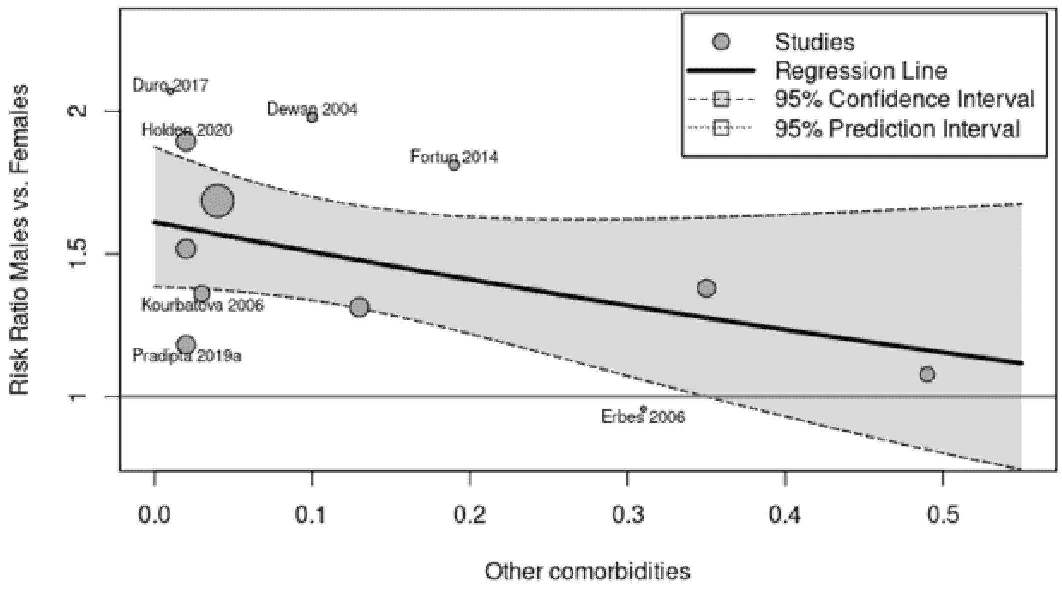

Figure A- 103: Bubble plot of moderator other comorbidities

XXIV. *Moderator other risk factors*

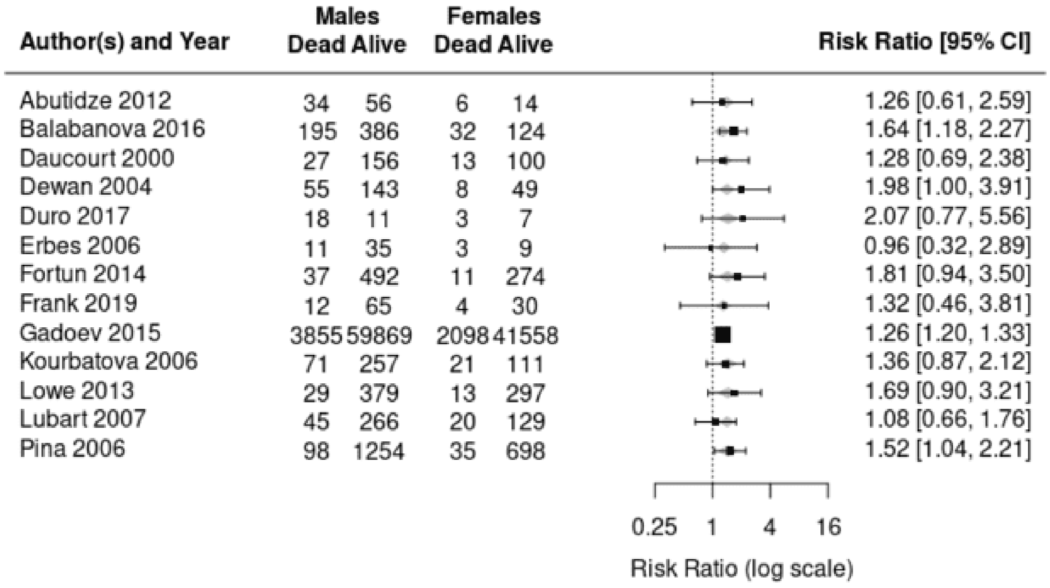

Figure A- 105: Forest plot of moderator other risk factors

Moderator other risk factors - Predicted pooled risk ratio (with 95% confidence/prediction intervals)

	pred	ci.lb	ci.ub	pi.lb	pi.ub
Abutidze 2012	1.40	1.21	1.62	1.14	1.72
Balabanova 2016	1.28	1.04	1.57	1.00	1.64
Daucourt 2000	1.37	1.22	1.54	1.14	1.65
Dewan 2004	1.41	1.20	1.67	1.13	1.76
Duro 2017	1.45	1.16	1.81	1.11	1.89
Erbes 2006	1.31	1.13	1.52	1.07	1.61
Fortun 2014	1.43	1.17	1.75	1.12	1.83
Frank 2019	1.30	1.09	1.54	1.04	1.62
Gadoev 2015	1.33	1.17	1.51	1.10	1.61
Kourbatova 2006	1.43	1.17	1.75	1.12	1.83
Lowe 2013	1.44	1.16	1.78	1.11	1.86
Lubart 2007	1.41	1.20	1.67	1.13	1.76
Pina 2006	1.44	1.16	1.80	1.11	1.88

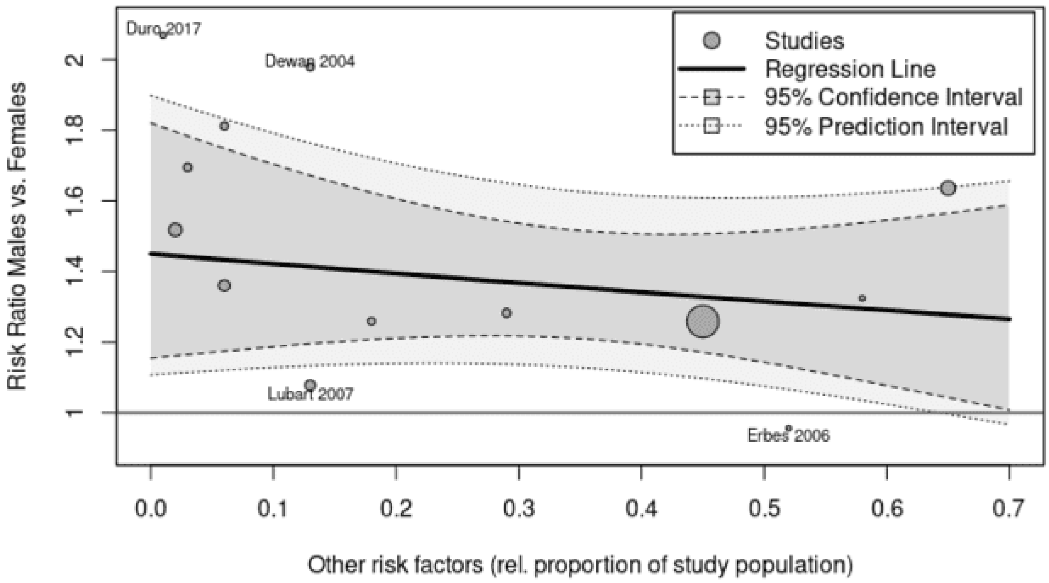

Figure A- 107: Bubble plot of moderator other risk factors

XXV. *Moderator prison*

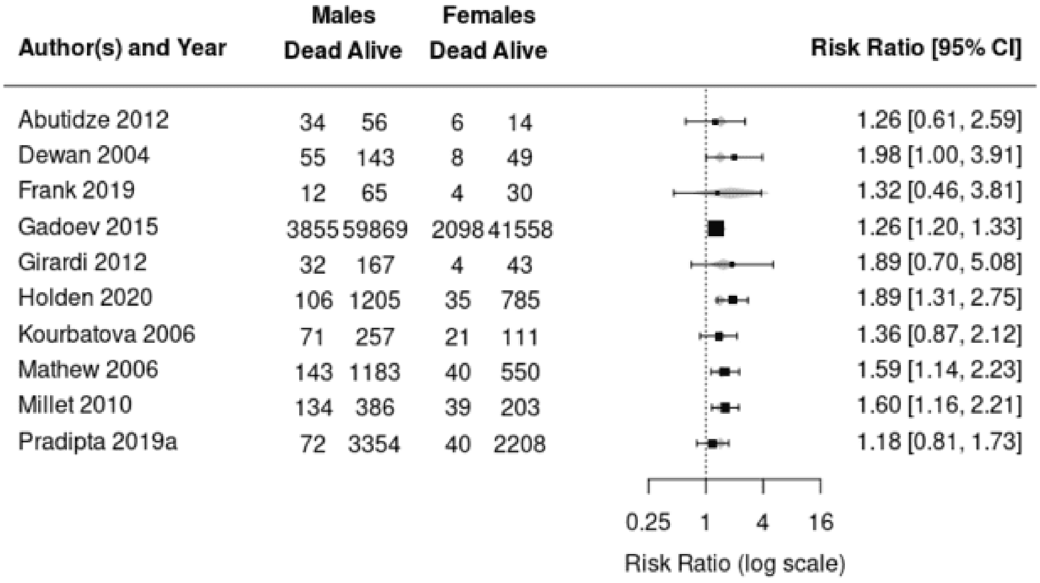

Figure A- 109: Forest plot of moderator prison

Moderator prison - Predicted pooled risk ratio (with 95% confidence/prediction intervals)

	pred	ci.lb	ci.ub	pi.lb	pi.ub
Abutidze 2012	1.43	1.25	1.64	1.13	1.82
Dewan 2004	1.41	1.24	1.61	1.12	1.79
Frank 2019	1.83	0.75	4.48	0.74	4.58
Gadoev 2015	1.38	1.19	1.61	1.08	1.77
Girardi 2012	1.53	1.14	2.06	1.07	2.19
Holden 2020	1.40	1.22	1.60	1.10	1.78
Kourbatova 2006	1.41	1.24	1.61	1.12	1.79
Mathew 2006	1.46	1.23	1.74	1.13	1.90
Millet 2010	1.43	1.25	1.64	1.13	1.82
Pradipta 2019a	1.41	1.24	1.61	1.12	1.79

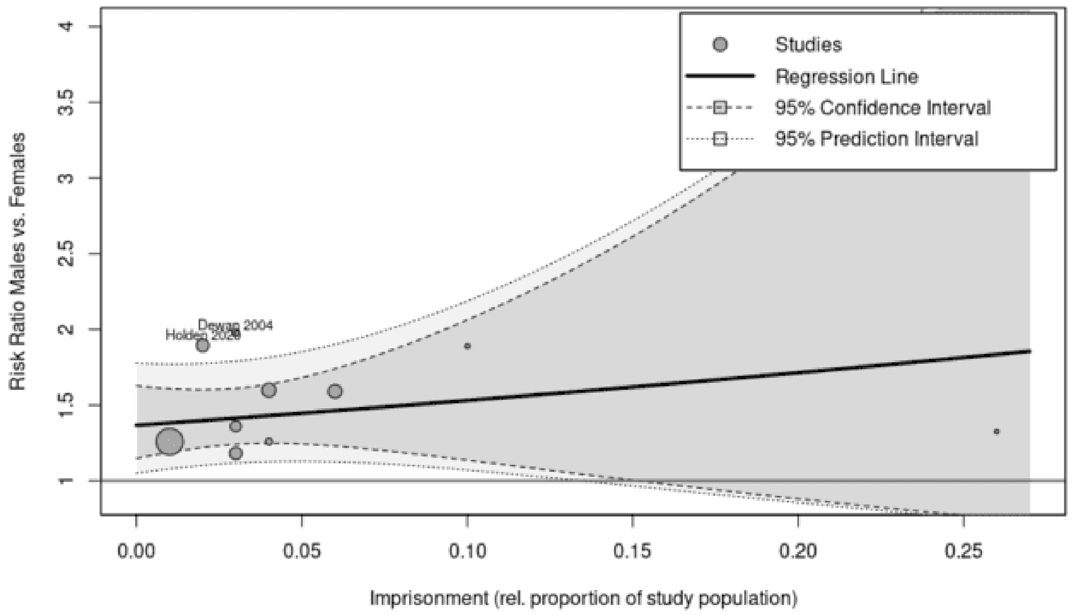

Figure A- 111: Bubble plot of moderator prison

XXVI. *Moderator pulmonary TB (PTB)*

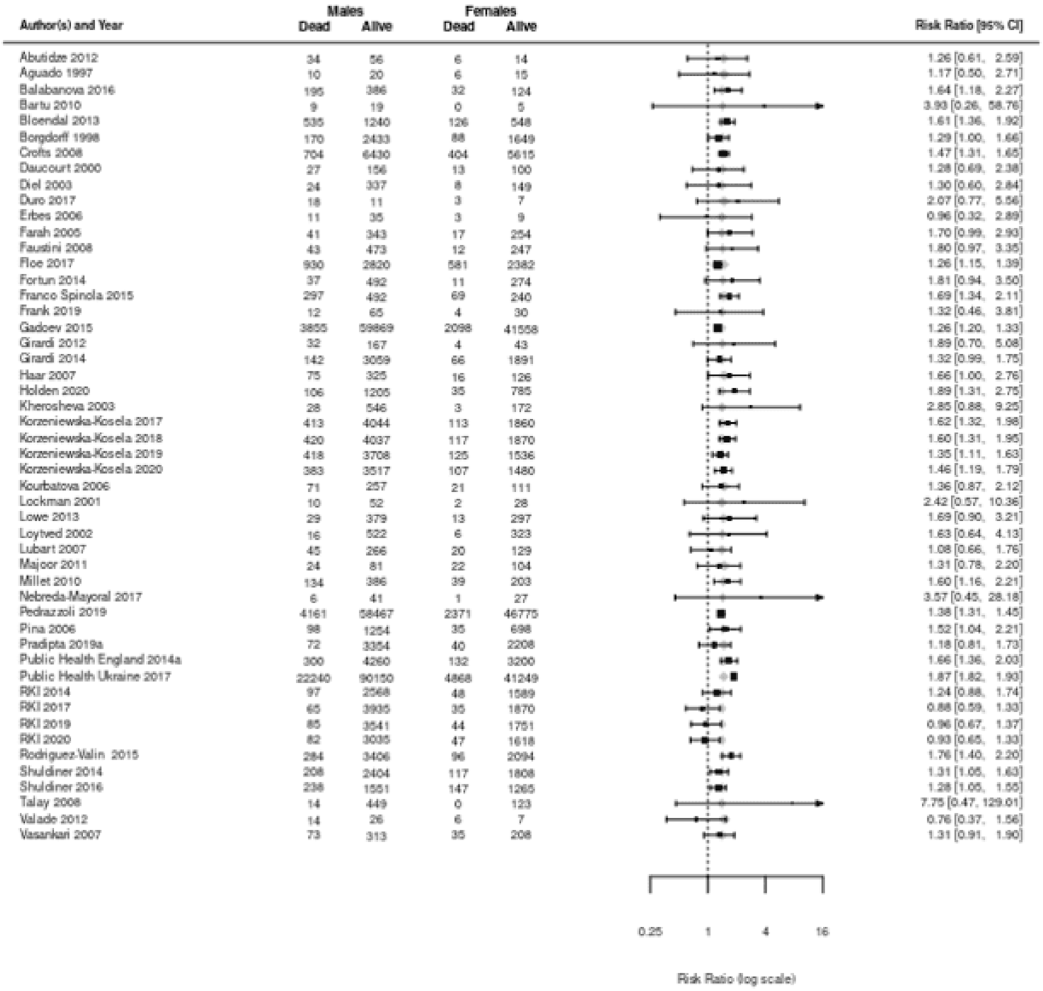

Figure A- 113: Forest plot of moderator pulmonary TB

Moderator pulmonary TB - Predicted pooled risk ratio (with 95% confidence/prediction intervals)

	pred	ci.lb	ci.ub	pi.lb	pi.ub
Abutidze 2012	1.46	1.33	1.61	1.13	1.89
Aguado 1997	1.48	1.30	1.68	1.13	1.93
Balabanova 2016	1.41	1.29	1.53	1.09	1.81
Bartu 2010	1.43	1.35	1.51	1.12	1.82
Bloendal 2013	1.40	1.28	1.54	1.09	1.81
Borgdorff 1998	1.44	1.35	1.52	1.13	1.83
Crofts 2008	1.43	1.35	1.52	1.12	1.83
Daucourt 2000	1.43	1.35	1.52	1.12	1.82

Diel 2003	1.40	1.28	1.54	1.09	1.81
Duro 2017	1.48	1.30	1.67	1.13	1.93
Erbes 2006	1.40	1.28	1.54	1.09	1.81
Farah 2005	1.40	1.28	1.54	1.09	1.81
Faustini 2008	1.43	1.35	1.52	1.12	1.82
Floe 2017	1.47	1.32	1.64	1.13	1.90
Fortun 2014	1.43	1.35	1.52	1.12	1.83
Franco Spinola 2015	1.47	1.32	1.63	1.13	1.90
Frank 2019	1.41	1.31	1.52	1.10	1.81
Gadoev 2015	1.42	1.33	1.52	1.11	1.81
Girardi 2012	1.42	1.32	1.52	1.11	1.81
Girardi 2014	1.41	1.29	1.53	1.10	1.81
Haar 2007	1.48	1.30	1.67	1.13	1.93
Holden 2020	1.42	1.34	1.51	1.11	1.82
Kherosheva 2003	1.41	1.29	1.53	1.09	1.81
Korzeniewska-Kosela 2017	1.41	1.29	1.53	1.09	1.81
Korzeniewska-Kosela 2018	1.41	1.29	1.53	1.09	1.81
Korzeniewska-Kosela 2019	1.41	1.29	1.53	1.09	1.81
Korzeniewska-Kosela 2020	1.42	1.34	1.51	1.12	1.82
Kourbatova 2006	1.48	1.31	1.67	1.13	1.92
Lockman 2001	1.40	1.28	1.54	1.09	1.81
Lowe 2013	1.46	1.34	1.58	1.13	1.87
Loytved 2002	1.42	1.32	1.52	1.11	1.81
Lubart 2007	1.40	1.28	1.54	1.09	1.81
Majoor 2011	1.48	1.30	1.68	1.13	1.93
Millet 2010	1.45	1.34	1.57	1.13	1.86
Nebreda-Mayoral 2017	1.43	1.35	1.52	1.12	1.83
Pedrazzoli 2019	1.44	1.35	1.52	1.13	1.83
Pina 2006	1.44	1.35	1.53	1.13	1.84
Pradipta 2019a	1.47	1.31	1.66	1.13	1.92
Public Health England 2014a	1.45	1.35	1.56	1.13	1.85
Public Health Ukraine 2017	1.47	1.32	1.62	1.13	1.89
RKI 2014	1.42	1.33	1.52	1.11	1.81
RKI 2017	1.42	1.33	1.51	1.11	1.82
RKI 2019	1.42	1.34	1.51	1.12	1.82
RKI 2020	1.42	1.34	1.51	1.12	1.82
Rodriguez-Valin 2015	1.42	1.34	1.51	1.12	1.82
Shuldiner 2014	1.42	1.32	1.52	1.11	1.81
Shuldiner 2016	1.42	1.32	1.52	1.11	1.81
Talay 2008	1.40	1.28	1.54	1.09	1.81
Valade 2012	1.47	1.31	1.66	1.13	1.92
Vasankari 2007	1.40	1.28	1.54	1.09	1.81

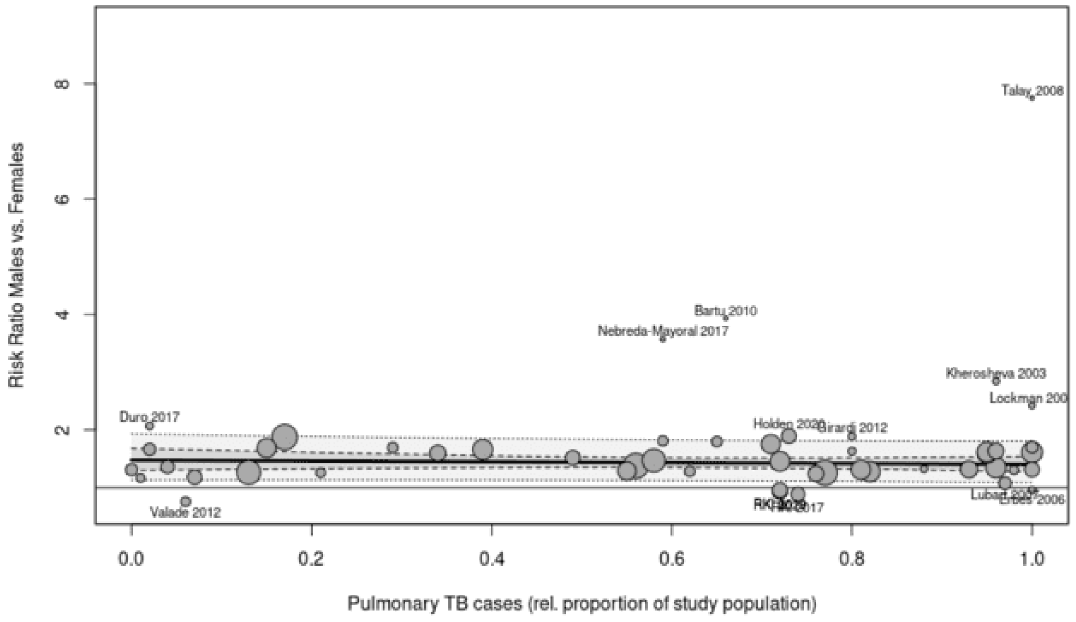

Figure A- 115: Bubble plot of moderator pulmonary TB

XXVII. Moderator smoker

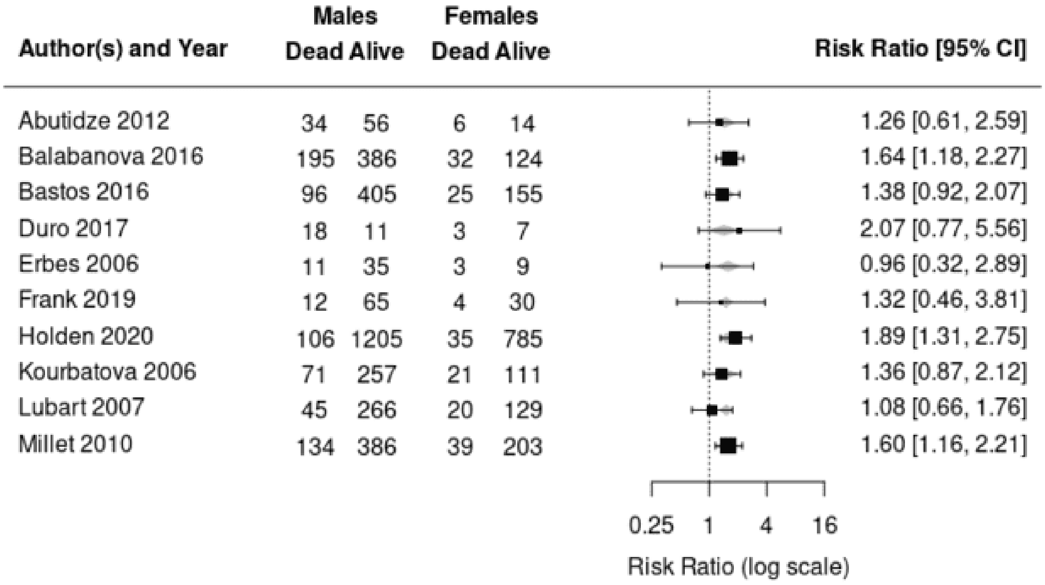

Figure A- 117: Forest plot of moderator smoker

Moderator smoker - Predicted pooled risk ratio (with 95% confidence/prediction intervals)

	pred	ci.lb	ci.ub	pi.lb	pi.ub
Abutidze 2012	1.46	1.17	1.83	1.17	1.83
Balabanova 2016	1.55	1.25	1.93	1.25	1.93
Bastos 2016	1.53	1.29	1.82	1.29	1.82
Duro 2017	1.42	1.02	1.99	1.02	1.99
Erbes 2006	1.58	1.19	2.08	1.19	2.08
Frank 2019	1.50	1.29	1.74	1.29	1.74
Holden 2020	1.54	1.28	1.86	1.28	1.86
Kourbatova 2006	1.44	1.09	1.91	1.09	1.91
Lubart 2007	1.51	1.30	1.75	1.30	1.75
Millet 2010	1.47	1.22	1.78	1.22	1.78

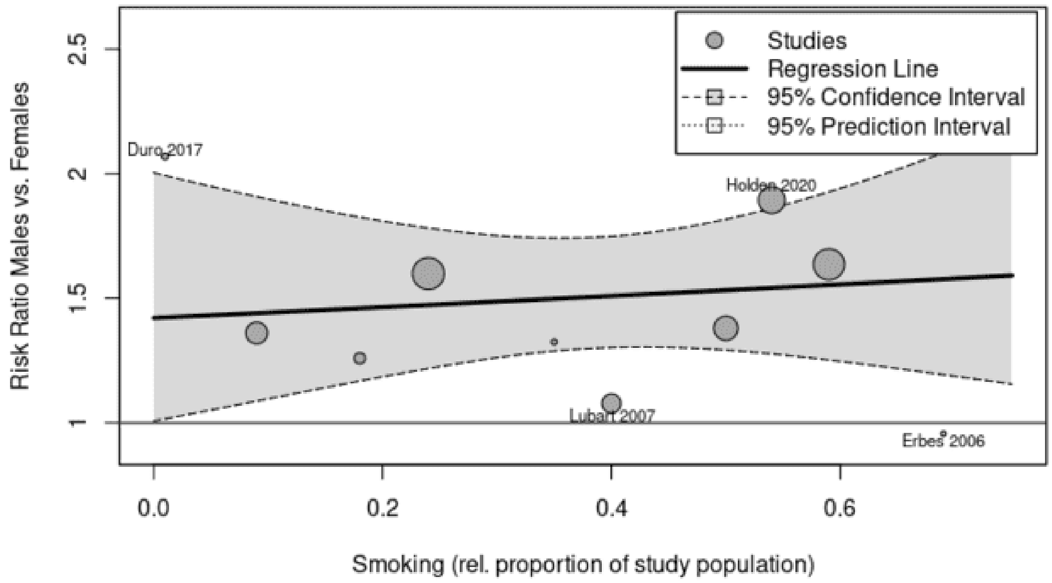

Figure A- 119: Bubble plot of moderator smoker

XXVIII. *Moderator extended-drug resistant TB (XDR TB)*

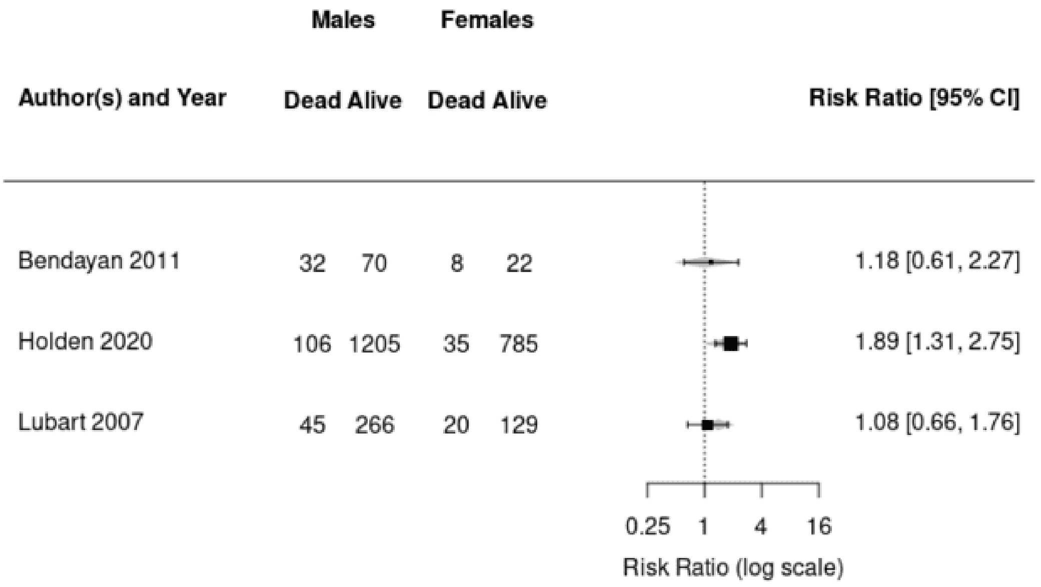

Figure A- 121: Forest plot of moderator extended drug-resistant TB

Moderator extended drug-resistant TB - Predicted pooled risk ratio (with 95% confidence/prediction intervals)

	pred	ci.lb	ci.ub	pi.lb	pi.ub
Bendayan 2011	1.02	0.50	2.05	0.46	2.25
Holden 2020	1.68	1.05	2.71	0.92	3.08
Lubart 2007	1.39	0.98	1.99	0.83	2.34

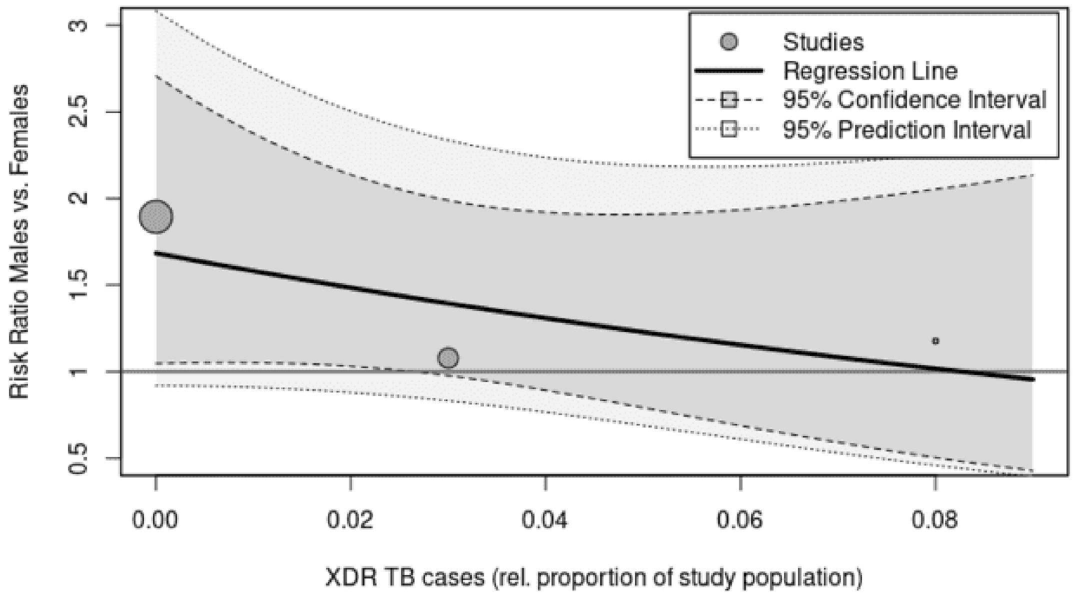

Figure A- 123: Bubble plot of moderator extended drug-resistant TB

XXIX. Subsequent analysis of the moderator median age

Mixed-Effects Model for RR of TB Mortality Males vs. Females with Moderator = Median Age

```
#### Supplementary analysis of the moderator median age
library(readr)
mrnew.dat <- read_csv("MRnewposit_medianage.csv")
View(mrnew.dat)
is.data.frame(mrnew.dat)
s <- spec(mrnew.dat)
s
summary(mrnew.dat)
any(is.na(mrnew.dat))
head(mrnew.dat)

### source of the following r codes:
https://wviechtb.github.io/metafor/reference/regplot.html;
https://wviechtb.github.io/metafor/

### copy mrnew data into 'dat'
dat <- mrnew.dat

### calculate log risk ratios and corresponding sampling variances
dat <- escalc(measure="RR", ai=m.e, bi=m.ne, ci=f.e, di=f.ne,
data=dat)

### fit mixed-effects model with the moderator median age
### note: method="REML" is the default, so one could leave this out
res <- rma(yi, vi, mods = ~ medianage, data=dat,
           slab=paste(study, sep=", ")) # also add study labels
res

# predicted pooled risk ratio (with 95% confidence/prediction
intervals)
predict(res, transf=exp, digits=2)

# forest plot
forest(res, atransf=exp, at=log(c(.25, 1, 4, 16)), xlim=c(-17,8),
       ilab=cbind(m.e, m.ne, f.e, f.ne), ilab.xpos=c(-9.5,-8,-6,-
4.5),
       header="Author(s) and Year")
text(c(-9.5,-8,-6,-4.5), 23, c("Dead", "Alive", "Dead", "Alive"),
font=2)
text(c(-8.75,-5.25),    24, c("Males", "Females"), font=2)

# funnel plot
funnel(res, ylim=c(0,0.8), las=1)

# regression test for funnel plot asymmetry
regtest(res)

### draw plot
regplot(res, mod="medianage", xlab="Median age of study population")

### adjust x-axis limits and back-transform to risk ratios
```

```
regplot(res, mod="medianage", xlab="Median age of study population",
xlim=c(29,68), transf=exp)

### also extend the prediction limits for the regression line
regplot(res, mod="medianage", xlab="Median age of study population",
xlim=c(29,68), predlim=c(29,68), transf=exp)

### add the prediction interval to the plot, add a reference line at
1, and add a legend
regplot(res, mod="medianage", pi=TRUE, xlab="Median age of study
population",
        xlim=c(29,68), predlim=c(29,68), transf=exp, refline=1,
legend=TRUE)

### label points outside of the prediction interval
regplot(res, mod="medianage", pi=TRUE, xlab="Median age of study
population", ylab="Risk Ratio Males vs. Females",
        xlim=c(29,68), ylim= c(0.4,8), predlim=c(29,68), transf=exp,
refline=1, legend=TRUE,  label="piout", labsize=0.7)

# assessing heterogeneity in r - report at least I² (with CI 95%) and
prediction intervals
```

Predicted pooled risk ratio (with 95% confidence/prediction intervals)

```
> predict(res, transf=exp, digits=2)

                 pred ci.lb ci.ub pi.lb pi.ub
Abutidze 2012    1.34  1.20  1.49  1.06  1.68
Bastos 2016      1.38  1.23  1.54  1.09  1.74
Baussano 2008    1.51  1.20  1.90  1.12  2.06
Bendayan 2011    1.32  1.18  1.47  1.05  1.66
Daucourt 2000    1.42  1.23  1.64  1.11  1.82
Dobrotkova 2019  1.42  1.23  1.64  1.11  1.82
Duro 2017        1.42  1.23  1.64  1.11  1.82
Frank 2019       1.27  1.11  1.46  1.00  1.63
Girardi 2012     1.29  1.15  1.46  1.02  1.64
Helbling 2002    1.31  1.16  1.47  1.03  1.65
Holden 2020      1.34  1.20  1.49  1.06  1.68
Holmberg 2019    1.31  1.17  1.47  1.04  1.65
Khaliaukin 2014  1.36  1.22  1.52  1.08  1.71
Korhonen 2020    1.49  1.21  1.83  1.12  1.99
Lockman 2001     1.40  1.23  1.60  1.10  1.78
Makhmudova 2019  1.26  1.08  1.47  0.98  1.62
Mathew 2006      1.34  1.20  1.49  1.06  1.68
Millet 2010      1.29  1.14  1.46  1.02  1.64
Podlekareva 2014 1.25  1.07  1.47  0.97  1.62
Podlekareva 2016 1.25  1.07  1.47  0.97  1.62
Valade 2012      1.33  1.19  1.48  1.06  1.67
```

Results- Univariate Model with Moderator = Median Age

```
Mixed-Effects Model (k = 21; tau^2 estimator: REML)

tau^2 (estimated amount of residual heterogeneity):     0.0107 (SE =
0.0170)
tau (square root of estimated tau^2 value):             0.1033
I^2 (residual heterogeneity / unaccounted variability): 18.20%
H^2 (unaccounted variability / sampling variability):   1.22
R^2 (amount of heterogeneity accounted for):            0.00%

Test for Residual Heterogeneity:
QE(df = 19) = 22.4396, p-val = 0.2629

Test of Moderators (coefficient 2):
QM(df = 1) = 1.3428, p-val = 0.2465

Model Results:

           estimate      se    zval    pval    ci.lb   ci.ub
intrcpt      0.0379  0.2279  0.1664  0.8678  -0.4087  0.4846
medianage    0.0060  0.0052  1.1588  0.2465  -0.0042  0.0162

---
Signif. codes:  0 ‘***’ 0.001 ‘**’ 0.01 ‘*’ 0.05 ‘.’ 0.1 ‘ ’ 1
```

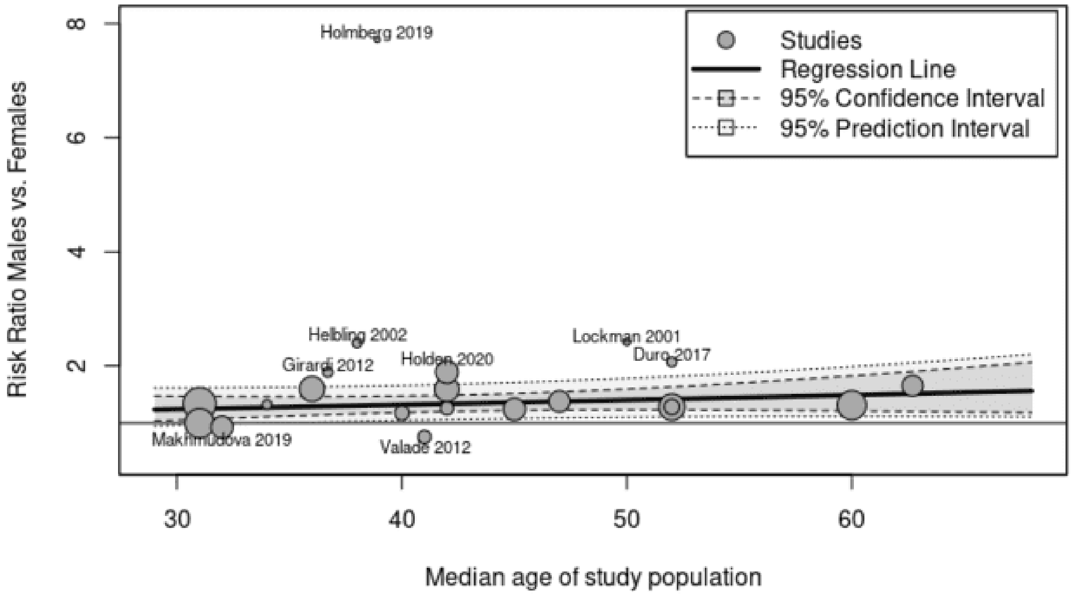

Figure A- 124: Bubble plot of moderator median age

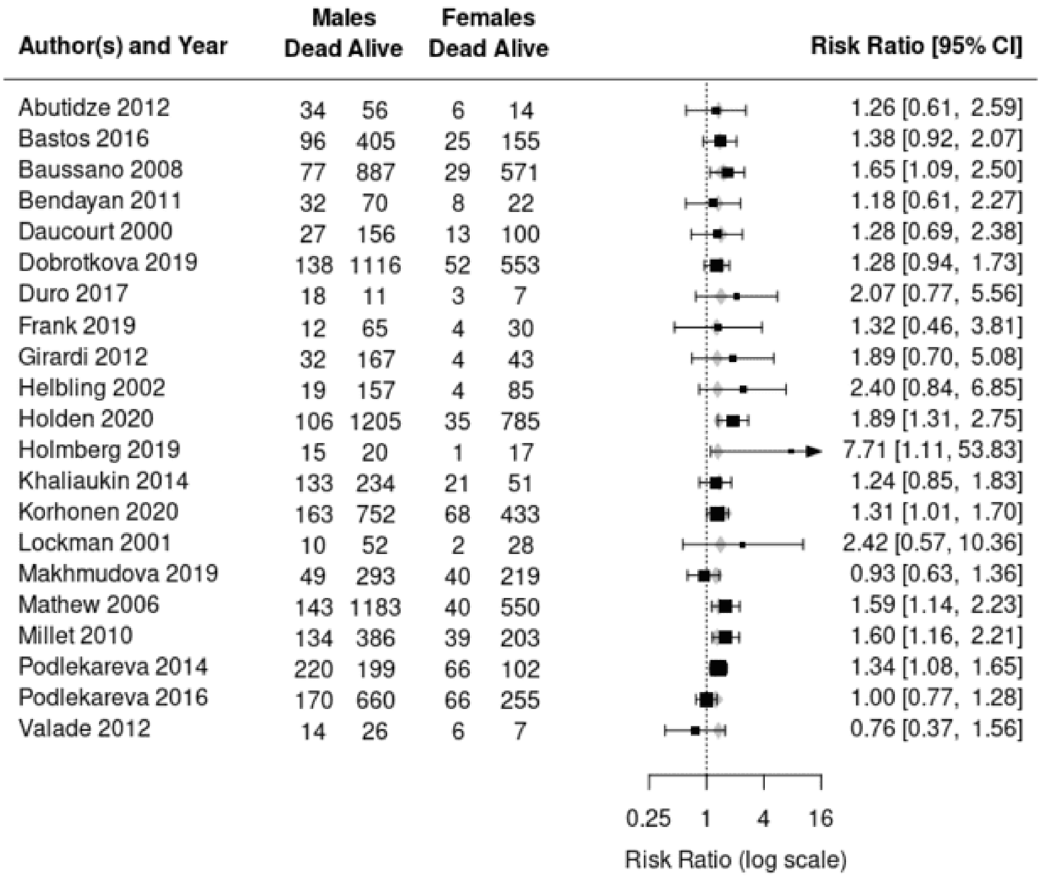

Figure A- 125: Forest plot of moderator median age

Regression Test for Funnel Plot Asymmetry

```
Model:     mixed-effects meta-regression model
Predictor: standard error

Test for Funnel Plot Asymmetry: z = 1.4716, p = 0.1411
```

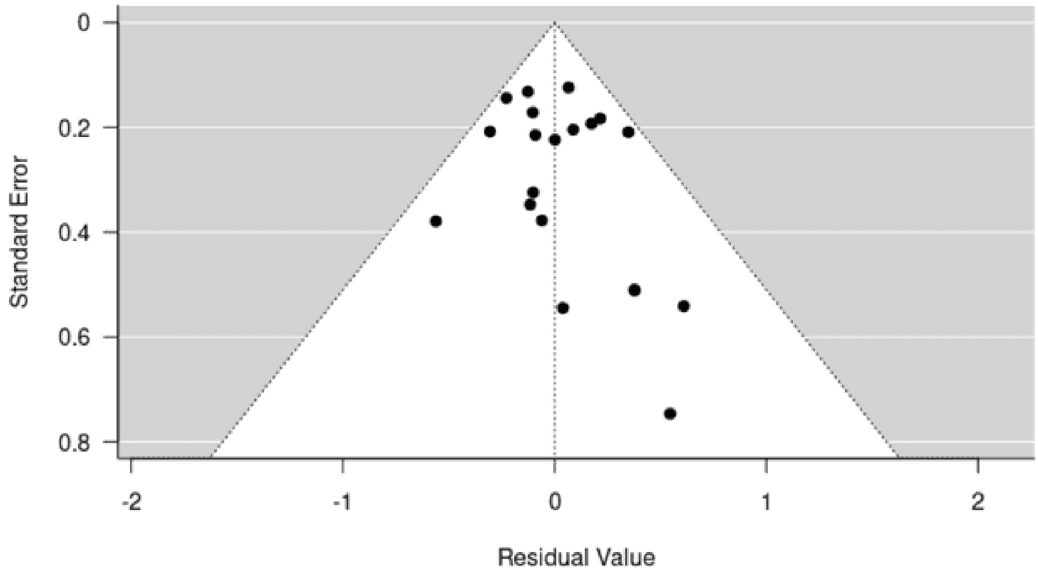

Figure A- 126: Funnel plot of moderator variable median age

Analysis Repetition w/o Holmberg 2019

Results- Univariate Model with Moderator = Median Age

```
Mixed-Effects Model (k = 20; tau^2 estimator: REML)

tau^2 (estimated amount of residual heterogeneity):     0.0103 (SE =
0.0168)
tau (square root of estimated tau^2 value):             0.1015
I^2 (residual heterogeneity / unaccounted variability): 18.42%
H^2 (unaccounted variability / sampling variability):   1.23
R^2 (amount of heterogeneity accounted for):            0.00%

Test for Residual Heterogeneity:
QE(df = 18) = 19.2245, p-val = 0.3781

Test of Moderators (coefficient 2):
QM(df = 1) = 1.4364, p-val = 0.2307

Model Results:

           estimate      se    zval    pval    ci.lb   ci.ub
intrcpt      0.0253  0.2266  0.1117  0.9111  -0.4189  0.4695
medianage    0.0062  0.0052  1.1985  0.2307  -0.0039  0.0163

---
Signif. codes:  0 ‘***’ 0.001 ‘**’ 0.01 ‘*’ 0.05 ‘.’ 0.1 ‘ ’ 1
```

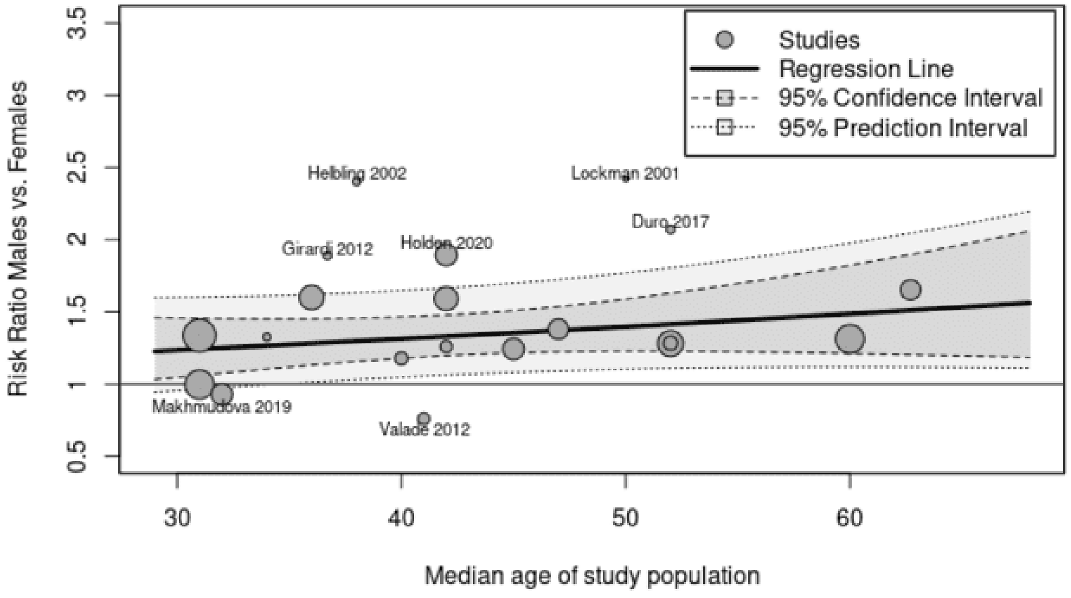

Figure A- 127: Bubble plot of moderatormedian age w/o Holmberg 2019

Author(s) and Year	Males Dead	Males Alive	Females Dead	Females Alive	Risk Ratio [95% CI]
Abutidze 2012	34	56	6	14	1.26 [0.61, 2.59]
Bastos 2016	96	405	25	155	1.38 [0.92, 2.07]
Baussano 2008	77	887	29	571	1.65 [1.09, 2.50]
Bendayan 2011	32	70	8	22	1.18 [0.61, 2.27]
Daucourt 2000	27	156	13	100	1.28 [0.69, 2.38]
Dobrotkova 2019	138	1116	52	553	1.28 [0.94, 1.73]
Duro 2017	18	11	3	7	2.07 [0.77, 5.56]
Frank 2019	12	65	4	30	1.32 [0.46, 3.81]
Girardi 2012	32	167	4	43	1.89 [0.70, 5.08]
Helbling 2002	19	157	4	85	2.40 [0.84, 6.85]
Holden 2020	106	1205	35	785	1.89 [1.31, 2.75]
Khaliaukin 2014	133	234	21	51	1.24 [0.85, 1.83]
Korhonen 2020	163	752	68	433	1.31 [1.01, 1.70]
Lockman 2001	10	52	2	28	2.42 [0.57, 10.36]
Makhmudova 2019	49	293	40	219	0.93 [0.63, 1.36]
Mathew 2006	143	1183	40	550	1.59 [1.14, 2.23]
Millet 2010	134	386	39	203	1.60 [1.16, 2.21]
Podlekareva 2014	220	199	66	102	1.34 [1.08, 1.65]
Podlekareva 2016	170	660	66	255	1.00 [0.77, 1.28]
Valade 2012	14	26	6	7	0.76 [0.37, 1.56]

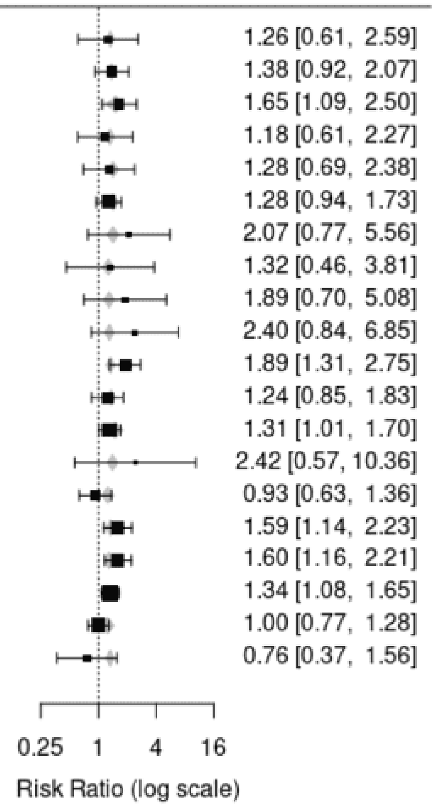

Figure A- 128: Forest plot of moderator median age w/o Holmberg 2019

Regression Test for Funnel Plot Asymmetry

```
Model:     mixed-effects meta-regression model
Predictor: standard error
Test for Funnel Plot Asymmetry: z = 0.8322, p = 0.4053
```

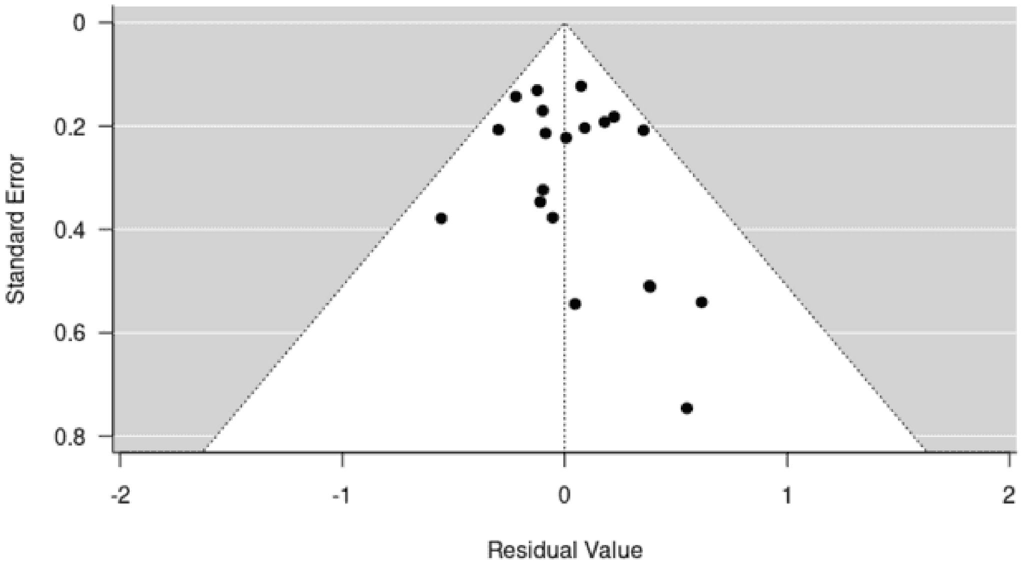

Figure A- 129: Funnel plot of moderator variable median age w/o Holmberg 2019
