# Supplementary material for: Tuberculosis case fatality is higher in male than female patients in Europe: a systematic review and meta-analysis
Source: Infection. 2024 Mar 23;52(5):1775–86. doi: 10.1007/s15010-024-02206-z (PMC11499538; doi:10.1007/s15010-024-02206-z)
Supplement: Supplementary file 29 — Online Resource 29 Multivariable meta-regression results (incl. R code) (PDF 269 KB) [file 15010_2024_2206_MOESM29_ESM.pdf]

Multivariable meta-regression results

I. *R code - start*

```
#### multivariable regression of different moderators
library(readr)
mrnew.dat <- read_csv("MRnewposit.csv")

View(mrnew.dat)
is.data.frame(mrnew.dat)

s <- spec(mrnew.dat)
s

summary(mrnew.dat)

any(is.na(mrnew.dat))

head(mrnew.dat)

### source of the following r codes:
https://wviechtb.github.io/metafor/reference/regplot.html;
https://wviechtb.github.io/metafor/


### copy mrnew data into 'dat'
dat <- mrnew.dat

### calculate log risk ratios and corresponding sampling variances
dat <- escalc(measure="RR", ai=m.e, bi=m.ne, ci=f.e, di=f.ne,
data=dat)
```

II. *Comorbidities*

Mixed-Effects Model for RR of TB Mortality Males vs. Females with Moderators = Diabetes + Cancers

```
### fit mixed-effects model with diabetes and cancers as moderators
res <- rma(yi, vi, mods = ~ diabetes.rel + cancers.rel, data=dat)
res

Mixed-Effects Model (k = 10; tau^2 estimator: REML)

tau^2 (estimated amount of residual heterogeneity):     0 (SE =
0.0234)
tau (square root of estimated tau^2 value):             0
I^2 (residual heterogeneity / unaccounted variability): 0.00%
H^2 (unaccounted variability / sampling variability):   1.00
R^2 (amount of heterogeneity accounted for):            0.00%

Test for Residual Heterogeneity:
QE(df = 7) = 3.5003, p-val = 0.8352

Test of Moderators (coefficients 2:3):
QM(df = 2) = 3.0652, p-val = 0.2160

Model Results:

              estimate      se     zval    pval    ci.lb    ci.ub
intrcpt         0.4444  0.1246   3.5674  0.0004   0.2003   0.6886
***
```

```
diabetes.rel   -2.5758  1.5041  -1.7125  0.0868  -5.5238
0.3722    .
cancers.rel     4.6123  4.3038   1.0717  0.2839  -3.8231  13.0476
---
Signif. codes:  0 '***' 0.001 '**' 0.01 '*' 0.05 '.' 0.1 ' ' 1
```

Mixed-Effects Model for RR of TB Mortality Males vs. Females with Moderators = HIV + Diabetes

```
### fit mixed-effects model with diabetes and cancers as moderators
res <- rma(yi, vi, mods = ~ hivaids.rel + diabetes.rel, data=dat)
res

Mixed-Effects Model (k = 13; tau^2 estimator: REML)

tau^2 (estimated amount of residual heterogeneity):     0 (SE =
0.0179)
tau (square root of estimated tau^2 value):             0
I^2 (residual heterogeneity / unaccounted variability): 0.00%
H^2 (unaccounted variability / sampling variability):   1.00
R^2 (amount of heterogeneity accounted for):            0.00%

Test for Residual Heterogeneity:
QE(df = 10) = 4.3015, p-val = 0.9327

Test of Moderators (coefficients 2:3):
QM(df = 2) = 2.5186, p-val = 0.2839

Model Results:

              estimate      se     zval    pval    ci.lb   ci.ub
intrcpt         0.5355  0.0938   5.7109  <.0001   0.3517  0.7193  ***
hivaids.rel    -0.2747  0.3691  -0.7442  0.4567  -0.9981  0.4487
diabetes.rel   -1.7970  1.2549  -1.4320  0.1521  -4.2566  0.6625


---
Signif. codes:  0 '***' 0.001 '**' 0.01 '*' 0.05 '.' 0.1 ' ' 1
```

Mixed-Effects Model for RR of TB Mortality Males vs. Females with Moderators = HIV + Hepcirrh

```
### fit mixed-effects model with diabetes and cancers as moderators
res <- rma(yi, vi, mods = ~ hivaids.rel + hepcirrh.rel, data=dat)
res

Mixed-Effects Model (k = 9; tau^2 estimator: REML)

tau^2 (estimated amount of residual heterogeneity):     0 (SE =
0.0329)
tau (square root of estimated tau^2 value):             0
I^2 (residual heterogeneity / unaccounted variability): 0.00%
H^2 (unaccounted variability / sampling variability):   1.00
R^2 (amount of heterogeneity accounted for):            100.00%

Test for Residual Heterogeneity:
QE(df = 6) = 2.5944, p-val = 0.8578

Test of Moderators (coefficients 2:3):
QM(df = 2) = 2.8090, p-val = 0.2455

Model Results:
```

```
               estimate      se     zval    pval    ci.lb   ci.ub
intrcpt          0.4802  0.0987   4.8653  <.0001   0.2868  0.6737  ***
hivaids.rel      0.3750  0.4557   0.8228  0.4106  -0.5182  1.2683
hepcirrh.rel    -1.0488  0.8443  -1.2423  0.2141  -2.7035  0.6059

---
Signif. codes:  0 ‘***’ 0.001 ‘**’ 0.01 ‘*’ 0.05 ‘.’ 0.1 ‘ ’ 1
```

Mixed-Effects Model for RR of TB Mortality Males vs. Females with Moderators = HIV + Cancers

```
### fit mixed-effects model with hivaids and cancers as moderators
res <- rma(yi, vi, mods = ~ hivaids.rel + cancers.rel, data=dat)
res

Mixed-Effects Model (k = 9; tau^2 estimator: REML)

tau^2 (estimated amount of residual heterogeneity):     0.0050 (SE =
0.0217)
tau (square root of estimated tau^2 value):             0.0707
I^2 (residual heterogeneity / unaccounted variability): 10.92%
H^2 (unaccounted variability / sampling variability):   1.12
R^2 (amount of heterogeneity accounted for):            0.00%

Test for Residual Heterogeneity:
QE(df = 6) = 4.5498, p-val = 0.6027

Test of Moderators (coefficients 2:3):
QM(df = 2) = 1.1770, p-val = 0.5551

Model Results:

              estimate      se     zval    pval    ci.lb    ci.ub
intrcpt         0.3139  0.1296   2.4230  0.0154   0.0600   0.5678  *
hivaids.rel    -2.1181  2.2386  -0.9461  0.3441  -6.5057   2.2696
cancers.rel     6.3873  6.0621   1.0536  0.2920  -5.4942  18.2687

---
Signif. Codes:  0 ‘***’ 0.001 ‘**’ 0.01 ‘*’ 0.05 ‘.’ 0.1 ‘ ’ 1
```

Mixed-Effects Model for RR of TB Mortality Males vs. Females with Moderators = HIV + Others

```
### fit mixed-effects model with hivaids and other as moderators
res <- rma(yi, vi, mods = ~ hivaids.rel + other.rel, data=dat)
res

Mixed-Effects Model (k = 10; tau^2 estimator: REML)

tau^2 (estimated amount of residual heterogeneity):     0 (SE =
0.0200)
tau (square root of estimated tau^2 value):             0
I^2 (residual heterogeneity / unaccounted variability): 0.00%
H^2 (unaccounted variability / sampling variability):   1.00
R^2 (amount of heterogeneity accounted for):            0.00%

Test for Residual Heterogeneity:
QE(df = 7) = 2.0987, p-val = 0.9542

Test of Moderators (coefficients 2:3):
QM(df = 2) = 4.3210, p-val = 0.1153
```

```
Model Results:

             estimate      se     zval    pval    ci.lb    ci.ub
intrcpt        0.4787  0.0921   5.1978  <.0001   0.2982   0.6591  ***
hivaids.rel    1.7799  1.6825   1.0579  0.2901  -1.5177   5.0775
other.rel     -1.3035  0.6487  -2.0095  0.0445  -2.5749  -0.0321    *

---
Signif. Codes:  0 '***' 0.001 '**' 0.01 '*' 0.05 '.' 0.1 ' ' 1
```

Mixed-Effects Model for RR of TB Mortality Males vs. Females with Moderators = Diabetes + Hepcirrh

```
### fit mixed-effects model with diabetes and hepcirrh as moderators
res <- rma(yi, vi, mods = ~ diabetes.rel + hepcirrh.rel, data=dat)
res

Mixed-Effects Model (k = 9; tau^2 estimator: REML)

tau^2 (estimated amount of residual heterogeneity):     0 (SE =
0.0497)
tau (square root of estimated tau^2 value):             0
I^2 (residual heterogeneity / unaccounted variability): 0.00%
H^2 (unaccounted variability / sampling variability):   1.00
R^2 (amount of heterogeneity accounted for):            100.00%

Test for Residual Heterogeneity:
QE(df = 6) = 1.5913, p-val = 0.9532

Test of Moderators (coefficients 2:3):
QM(df = 2) = 3.4995, p-val = 0.1738

Model Results:

              estimate      se     zval    pval    ci.lb   ci.ub
intrcpt         0.5697  0.1279   4.4533  <.0001   0.3189  0.8204  ***
diabetes.rel   -1.6312  2.0324  -0.8026  0.4222  -5.6147  2.3523
hepcirrh.rel   -0.7673  0.7567  -1.0141  0.3105  -2.2504  0.7157

---
Signif. codes:  0 '***' 0.001 '**' 0.01 '*' 0.05 '.' 0.1 ' ' 1
```

Mixed-Effects Model for RR of TB Mortality Males vs. Females with Moderators = Diabetes + Other

```
### fit mixed-effects model with diabetes and other as moderators
res <- rma(yi, vi, mods = ~ diabetes.rel + other.rel, data=dat)
res

Mixed-Effects Model (k = 12; tau^2 estimator: REML)

tau^2 (estimated amount of residual heterogeneity):     0 (SE =
0.0194)
tau (square root of estimated tau^2 value):             0
I^2 (residual heterogeneity / unaccounted variability): 0.00%
H^2 (unaccounted variability / sampling variability):   1.00
R^2 (amount of heterogeneity accounted for):            0.00%

Test for Residual Heterogeneity:
QE(df = 9) = 5.7465, p-val = 0.7650

Test of Moderators (coefficients 2:3):
QM(df = 2) = 2.8827, p-val = 0.2366
```

```
Model Results:

              estimate      se     zval    pval    ci.lb   ci.ub
intrcpt         0.5209  0.0947   5.4986  <.0001   0.3352  0.7066  ***
diabetes.rel   -1.3597  1.6991  -0.8002  0.4236  -4.6899  1.9705
other.rel      -0.3622  0.5859  -0.6183  0.5364  -1.5106  0.7861

---
Signif. codes:  0 ‘***’ 0.001 ‘**’ 0.01 ‘*’ 0.05 ‘.’ 0.1 ‘ ’ 1
```

Mixed-Effects Model for RR of TB Mortality Males vs. Females with Moderators = Cancers + Hepcirrh

```
### fit mixed-effects model with cancers and hepcirrh as moderators
res <- rma(yi, vi, mods = ~ cancers.rel + hepcirrh.rel, data=dat)
res

Mixed-Effects Model (k = 5; tau^2 estimator: REML)

tau^2 (estimated amount of residual heterogeneity):     0 (SE =
0.1061)
tau (square root of estimated tau^2 value):             0
I^2 (residual heterogeneity / unaccounted variability): 0.00%
H^2 (unaccounted variability / sampling variability):   1.00
R^2 (amount of heterogeneity accounted for):            0.00%

Test for Residual Heterogeneity:
QE(df = 2) = 0.1289, p-val = 0.9376

Test of Moderators (coefficients 2:3):
QM(df = 2) = 1.7929, p-val = 0.4080

Model Results:

              estimate      se     zval    pval     ci.lb   ci.ub
intrcpt         0.5857  0.1793   3.2664  0.0011    0.2343  0.9372  **
cancers.rel    -2.4072  5.8109  -0.4143  0.6787  -13.7964  8.9820
hepcirrh.rel   -0.9783  0.9129  -1.0716  0.2839   -2.7675  0.8109

---
Signif. codes:  0 ‘***’ 0.001 ‘**’ 0.01 ‘*’ 0.05 ‘.’ 0.1 ‘ ’ 1
```

Mixed-Effects Model for RR of TB Mortality Males vs. Females with Moderators = Cancers + Other

```
### fit mixed-effects model with cancers and other as moderators
res <- rma(yi, vi, mods = ~ cancers.rel + other.rel, data=dat)
res

Mixed-Effects Model (k = 9; tau^2 estimator: REML)

tau^2 (estimated amount of residual heterogeneity):     0 (SE =
0.0215)
tau (square root of estimated tau^2 value):             0
I^2 (residual heterogeneity / unaccounted variability): 0.00%
H^2 (unaccounted variability / sampling variability):   1.00
R^2 (amount of heterogeneity accounted for):            0.00%

Test for Residual Heterogeneity:
```

```
QE(df = 6) = 4.6590, p-val = 0.5882

Test of Moderators (coefficients 2:3):
QM(df = 2) = 1.8396, p-val = 0.3986

Model Results:

             estimate      se     zval    pval    ci.lb    ci.ub
intrcpt        0.3876  0.1210   3.2026  0.0014   0.1504   0.6248  **
cancers.rel    4.7963  4.7156   1.0171  0.3091  -4.4461  14.0388
other.rel     -1.0433  0.7942  -1.3137  0.1890  -2.5998   0.5133

---
Signif. codes:  0 ‘***’ 0.001 ‘**’ 0.01 ‘*’ 0.05 ‘.’ 0.1 ‘ ’ 1
```

Mixed-Effects Model for RR of TB Mortality Males vs. Females with Moderators = Hepcirrh + Other

```
### fit mixed-effects model with hepcirrh and other as moderators
res <- rma(yi, vi, mods = ~ hepcirrh.rel + other.rel, data=dat)
res

Mixed-Effects Model (k = 6; tau^2 estimator: REML)

tau^2 (estimated amount of residual heterogeneity):     0 (SE =
0.0558)
tau (square root of estimated tau^2 value):             0
I^2 (residual heterogeneity / unaccounted variability): 0.00%
H^2 (unaccounted variability / sampling variability):   1.00
R^2 (amount of heterogeneity accounted for):            100.00%

Test for Residual Heterogeneity:
QE(df = 3) = 0.7932, p-val = 0.8511

Test of Moderators (coefficients 2:3):
QM(df = 2) = 3.4308, p-val = 0.1799

Model Results:

              estimate      se     zval    pval    ci.lb   ci.ub
intrcpt         0.5850  0.1224   4.7802  <.0001   0.3452  0.8249  ***
hepcirrh.rel   -0.4287  1.0846  -0.3952  0.6927  -2.5545  1.6971
other.rel      -0.7455  0.6833  -1.0910  0.2753  -2.0847  0.5938

---
Signif. codes:  0 ‘***’ 0.001 ‘**’ 0.01 ‘*’ 0.05 ‘.’ 0.1 ‘ ’ 1
```

Mixed-Effects Model for RR of TB Mortality Males vs. Females with Moderators = HIV + Diabetes + Cancers

```
### fit mixed-effects model with hivaids, diabetes and cancers as
moderators
res <- rma(yi, vi, mods = ~ hivaids.rel + diabetes.rel + cancers.rel,
data=dat)
res

Mixed-Effects Model (k = 8; tau^2 estimator: REML)

tau^2 (estimated amount of residual heterogeneity):     0 (SE =
0.0342)
```

```
tau (square root of estimated tau^2 value):             0
I^2 (residual heterogeneity / unaccounted variability): 0.00%
H^2 (unaccounted variability / sampling variability):   1.00
R^2 (amount of heterogeneity accounted for):            0.00%

Test for Residual Heterogeneity:
QE(df = 4) = 0.7192, p-val = 0.9489

Test of Moderators (coefficients 2:4):
QM(df = 3) = 3.4854, p-val = 0.3227

Model Results:

              estimate      se     zval    pval    ci.lb    ci.ub
intrcpt         0.4492  0.1386   3.2415  0.0012   0.1776   0.7209  **
hivaids.rel    -1.9064  2.1145  -0.9016  0.3673  -6.0508   2.2380
diabetes.rel   -2.3894  1.5251  -1.5667  0.1172  -5.3785   0.5997
cancers.rel     7.9165  6.0012   1.3192  0.1871  -3.8456  19.6786

---
Signif. codes:  0 ‘***’ 0.001 ‘**’ 0.01 ‘*’ 0.05 ‘.’ 0.1 ‘ ’ 1
```

Mixed-Effects Model for RR of TB Mortality Males vs. Females with Moderators = HIV + Diabetes + Hepcirrh

```
### fit mixed-effects model with hivaids, diabetes and hepcirhh as
moderators
res <- rma(yi, vi, mods = ~ hivaids.rel + diabetes.rel +
hepcirrh.rel, data=dat)
res

Mixed-Effects Model (k = 8; tau^2 estimator: REML)

tau^2 (estimated amount of residual heterogeneity):     0 (SE =
0.0621)
tau (square root of estimated tau^2 value):             0
I^2 (residual heterogeneity / unaccounted variability): 0.00%
H^2 (unaccounted variability / sampling variability):   1.00
R^2 (amount of heterogeneity accounted for):            100.00%

Test for Residual Heterogeneity:
QE(df = 4) = 1.5899, p-val = 0.8106

Test of Moderators (coefficients 2:4):
QM(df = 3) = 2.9721, p-val = 0.3960

Model Results:

              estimate      se     zval    pval    ci.lb   ci.ub
intrcpt         0.5695  0.1408   4.0461  <.0001   0.2936  0.8454  ***
hivaids.rel    -0.0067  0.5971  -0.0112  0.9911  -1.1771  1.1637
diabetes.rel   -1.6213  2.4595  -0.6592  0.5098  -6.4417  3.1992
hepcirrh.rel   -0.7552  1.1584  -0.6520  0.5144  -3.0257  1.5152

---
Signif. codes:  0 ‘***’ 0.001 ‘**’ 0.01 ‘*’ 0.05 ‘.’ 0.1 ‘ ’ 1
```

Mixed-Effects Model for RR of TB Mortality Males vs. Females with Moderators = HIV + Diabetes + Other

```
### fit mixed-effects model with hivaids, diabetes and other as
moderators
```

```
res <- rma(yi, vi, mods = ~ hivaids.rel + diabetes.rel + other.rel,
data=dat)
res

Mixed-Effects Model (k = 10; tau^2 estimator: REML)

tau^2 (estimated amount of residual heterogeneity):     0 (SE =
0.0233)
tau (square root of estimated tau^2 value):             0
I^2 (residual heterogeneity / unaccounted variability): 0.00%
H^2 (unaccounted variability / sampling variability):   1.00
R^2 (amount of heterogeneity accounted for):            0.00%

Test for Residual Heterogeneity:
QE(df = 6) = 2.0727, p-val = 0.9129

Test of Moderators (coefficients 2:4):
QM(df = 3) = 4.3470, p-val = 0.2263

Model Results:

              estimate      se     zval    pval    ci.lb   ci.ub
intrcpt         0.4898  0.1150   4.2572  <.0001   0.2643  0.7153  ***
hivaids.rel     1.7068  1.7425   0.9795  0.3273  -1.7083  5.1220
diabetes.rel   -0.2967  1.8400  -0.1612  0.8719  -3.9030  3.3097
other.rel      -1.2154  0.8484  -1.4326  0.1520  -2.8781  0.4474

---
Signif. codes:  0 '***' 0.001 '**' 0.01 '*' 0.05 '.' 0.1 ' ' 1
```

Mixed-Effects Model for RR of TB Mortality Males vs. Females with Moderators = Diabetes + Cancers + Hepcirrh

```
### fit mixed-effects model with diabetes, cancers and hepcirrh as
moderators
res <- rma(yi, vi, mods = ~ diabetes.rel + cancers.rel +
hepcirrh.rel, data=dat)
res

Mixed-Effects Model (k = 5; tau^2 estimator: REML)

tau^2 (estimated amount of residual heterogeneity):     0 (SE =
0.2759)
tau (square root of estimated tau^2 value):             0
I^2 (residual heterogeneity / unaccounted variability): 0.00%
H^2 (unaccounted variability / sampling variability):   1.00
R^2 (amount of heterogeneity accounted for):            0.00%

Test for Residual Heterogeneity:
QE(df = 1) = 0.1289, p-val = 0.7196

Test of Moderators (coefficients 2:4):
QM(df = 3) = 1.7930, p-val = 0.6165

Model Results:

              estimate       se     zval    pval     ci.lb    ci.ub
intrcpt         0.5874   0.3210   1.8296  0.0673   -0.0419
1.2166  .
diabetes.rel    0.1079  17.3249   0.0062  0.9950  -33.8482  34.0640
cancers.rel    -2.5982  31.2030  -0.0833  0.9336  -63.7549  58.5586
hepcirrh.rel   -1.0007   3.7230  -0.2688  0.7881   -8.2977   6.2962
```

```
---
Signif. codes:  0 ‘***’ 0.001 ‘**’ 0.01 ‘*’ 0.05 ‘.’ 0.1 ‘ ’ 1
```

Mixed-Effects Model for RR of TB Mortality Males vs. Females with Moderators = Diabetes + Cancers + Other

```
### fit mixed-effects model with diabetes, cancers and other as
moderators
res <- rma(yi, vi, mods = ~ diabetes.rel + cancers.rel + other.rel,
data=dat)
res

Mixed-Effects Model (k = 9; tau^2 estimator: REML)

tau^2 (estimated amount of residual heterogeneity):     0 (SE =
0.0258)
tau (square root of estimated tau^2 value):             0
I^2 (residual heterogeneity / unaccounted variability): 0.00%
H^2 (unaccounted variability / sampling variability):   1.00
R^2 (amount of heterogeneity accounted for):            0.00%

Test for Residual Heterogeneity:
QE(df = 5) = 3.3517, p-val = 0.6459

Test of Moderators (coefficients 2:4):
QM(df = 3) = 3.1469, p-val = 0.3695

Model Results:

              estimate      se     zval    pval    ci.lb    ci.ub
intrcpt         0.4366  0.1284   3.4005  0.0007   0.1850   0.6883
***
diabetes.rel   -2.1486  1.8792  -1.1434  0.2529  -5.8318   1.5345
cancers.rel     5.2545  4.7326   1.1103  0.2669  -4.0212  14.5302
other.rel      -0.3676  0.9899  -0.3714  0.7104  -2.3078   1.5726

---
Signif. codes:  0 ‘***’ 0.001 ‘**’ 0.01 ‘*’ 0.05 ‘.’ 0.1 ‘ ’ 1
```

III. Risk Factors

Mixed-Effects Model for RR of TB Mortality Males vs. Females with Moderators = Alcohol + Nohome

```
### fit mixed-effects model with alcohol and nohome as moderators
res <- rma(yi, vi, mods = ~ alcohol.rel + nohome.rel, data=dat)
res

Mixed-Effects Model (k = 9; tau^2 estimator: REML)

tau^2 (estimated amount of residual heterogeneity):     0 (SE =
0.0230)
tau (square root of estimated tau^2 value):             0
I^2 (residual heterogeneity / unaccounted variability): 0.00%
H^2 (unaccounted variability / sampling variability):   1.00
R^2 (amount of heterogeneity accounted for):            100.00%

Test for Residual Heterogeneity:
QE(df = 6) = 3.6031, p-val = 0.7302

Test of Moderators (coefficients 2:3):
QM(df = 2) = 2.6475, p-val = 0.2661
```

```
Model Results:

             estimate      se    zval    pval    ci.lb    ci.ub
intrcpt        0.2804  0.1024  2.7391  0.0062   0.0798   0.4810  **
alcohol.rel    0.1368  0.5956  0.2297  0.8183  -1.0305   1.3041
nohome.rel     2.7043  3.7928  0.7130  0.4758  -4.7294  10.1381

---
Signif. codes:  0 ‘***’ 0.001 ‘**’ 0.01 ‘*’ 0.05 ‘.’ 0.1 ‘ ’ 1
```

Mixed-Effects Model for RR of TB Mortality Males vs. Females with Moderators = Alcohol + Smoker

```
### fit mixed-effects model with alcohol and smoker as moderators
res <- rma(yi, vi, mods = ~ alcohol.rel + smoker.rel, data=dat)
res

Mixed-Effects Model (k = 10; tau^2 estimator: REML)

tau^2 (estimated amount of residual heterogeneity):     0 (SE =
0.0303)
tau (square root of estimated tau^2 value):             0
I^2 (residual heterogeneity / unaccounted variability): 0.00%
H^2 (unaccounted variability / sampling variability):   1.00
R^2 (amount of heterogeneity accounted for):            0.00%

Test for Residual Heterogeneity:
QE(df = 7) = 4.8322, p-val = 0.6804

Test of Moderators (coefficients 2:3):
QM(df = 2) = 0.5160, p-val = 0.7726

Model Results:

             estimate      se     zval    pval    ci.lb   ci.ub
intrcpt        0.3811  0.1821   2.0924  0.0364   0.0241  0.7381  *
alcohol.rel    0.4621  0.7530   0.6136  0.5395  -1.0138  1.9379
smoker.rel    -0.2353  0.7487  -0.3142  0.7534  -1.7027  1.2322

---
Signif. codes:  0 ‘***’ 0.001 ‘**’ 0.01 ‘*’ 0.05 ‘.’ 0.1 ‘ ’ 1
```

Mixed-Effects Model for RR of TB Mortality Males vs. Females with Moderators = Alcohol + Drugs

```
### fit mixed-effects model with alcohol and drugs as moderators
res <- rma(yi, vi, mods = ~ alcohol.rel + drugs.rel, data=dat)
res

Mixed-Effects Model (k = 15; tau^2 estimator: REML)

tau^2 (estimated amount of residual heterogeneity):     0.0004 (SE =
0.0148)
tau (square root of estimated tau^2 value):             0.0199
I^2 (residual heterogeneity / unaccounted variability): 0.84%
H^2 (unaccounted variability / sampling variability):   1.01
R^2 (amount of heterogeneity accounted for):            0.00%

Test for Residual Heterogeneity:
QE(df = 12) = 8.1843, p-val = 0.7706
```

```
Test of Moderators (coefficients 2:3):
QM(df = 2) = 1.1418, p-val = 0.5650

Model Results:

             estimate      se     zval    pval    ci.lb   ci.ub
intrcpt        0.3408  0.0779   4.3735  <.0001   0.1881  0.4935  ***
alcohol.rel    0.5724  0.6615   0.8652  0.3869  -0.7242  1.8690
drugs.rel     -0.1027  1.0415  -0.0986  0.9215  -2.1440  1.9387

---
Signif. Codes:  0 ‘***’ 0.001 ‘**’ 0.01 ‘*’ 0.05 ‘.’ 0.1 ‘ ’ 1
```

Mixed-Effects Model for RR of TB Mortality Males vs. Females with Moderators = Alcohol + Migrant

```
### fit mixed-effects model with alcohol and migrant as moderators
res <- rma(yi, vi, mods = ~ alcohol.rel + migrant.rel, data=dat)
res

Mixed-Effects Model (k = 5; tau^2 estimator: REML)

tau^2 (estimated amount of residual heterogeneity):     0 (SE =
0.0434)
tau (square root of estimated tau^2 value):             0
I^2 (residual heterogeneity / unaccounted variability): 0.00%
H^2 (unaccounted variability / sampling variability):   1.00
R^2 (amount of heterogeneity accounted for):            0.00%

Test for Residual Heterogeneity:
QE(df = 2) = 0.0146, p-val = 0.9927

Test of Moderators (coefficients 2:3):
QM(df = 2) = 1.7303, p-val = 0.4210

Model Results:

             estimate      se    zval    pval    ci.lb    ci.ub
intrcpt        0.0070  0.3224  0.0216  0.9828  -0.6250   0.6389
alcohol.rel    4.2910  3.5771  1.1996  0.2303  -2.7200  11.3021
migrant.rel    0.4171  0.6766  0.6165  0.5376  -0.9089   1.7431

---
Signif. codes:  0 ‘***’ 0.001 ‘**’ 0.01 ‘*’ 0.05 ‘.’ 0.1 ‘ ’ 1
```

Mixed-Effects Model for RR of TB Mortality Males vs. Females with Moderators = Alcohol + Formertb

```
### fit mixed-effects model with alcohol and formertb as moderators
res <- rma(yi, vi, mods = ~ alcohol.rel + formertb.rel, data=dat)
res

Mixed-Effects Model (k = 12; tau^2 estimator: REML)

tau^2 (estimated amount of residual heterogeneity):     0 (SE =
0.0169)
tau (square root of estimated tau^2 value):             0
I^2 (residual heterogeneity / unaccounted variability): 0.00%
H^2 (unaccounted variability / sampling variability):   1.00
R^2 (amount of heterogeneity accounted for):            100.00%
```

```
Test for Residual Heterogeneity:
QE(df = 9) = 4.9155, p-val = 0.8416

Test of Moderators (coefficients 2:3):
QM(df = 2) = 3.3089, p-val = 0.1912

Model Results:

              estimate      se     zval    pval    ci.lb   ci.ub
intrcpt         0.4161  0.0791   5.2584  <.0001   0.2610  0.5712  ***
alcohol.rel     0.9276  0.5380   1.7241  0.0847  -0.1269  1.9821    .
formertb.rel   -0.9547  0.5488  -1.7396  0.0819  -2.0303  0.1209    .

---
Signif. codes:  0 ‘***’ 0.001 ‘**’ 0.01 ‘*’ 0.05 ‘.’ 0.1 ‘ ’ 1
```

Mixed-Effects Model for RR of TB Mortality Males vs. Females with Moderators = Alcohol + Prison

```
### fit mixed-effects model with alcohol and prison as moderators
res <- rma(yi, vi, mods = ~ alcohol.rel + prison.rel, data=dat)
res

Mixed-Effects Model (k = 8; tau^2 estimator: REML)

tau^2 (estimated amount of residual heterogeneity):     0 (SE =
0.0281)
tau (square root of estimated tau^2 value):             0
I^2 (residual heterogeneity / unaccounted variability): 0.00%
H^2 (unaccounted variability / sampling variability):   1.00
R^2 (amount of heterogeneity accounted for):            0.00%

Test for Residual Heterogeneity:
QE(df = 5) = 1.8860, p-val = 0.8647

Test of Moderators (coefficients 2:3):
QM(df = 2) = 2.4308, p-val = 0.2966

Model Results:

             estimate      se     zval    pval    ci.lb   ci.ub
intrcpt        0.3048  0.1569   1.9430  0.0520  -0.0027  0.6122  .
alcohol.rel    1.0728  0.7010   1.5304  0.1259  -0.3011  2.4467
prison.rel    -1.5944  2.3392  -0.6816  0.4955  -6.1792  2.9903

---
Signif. codes:  0 ‘***’ 0.001 ‘**’ 0.01 ‘*’ 0.05 ‘.’ 0.1 ‘ ’ 1
```

Mixed-Effects Model for RR of TB Mortality Males vs. Females with Moderators = Alcohol + Otherrf

```
### fit mixed-effects model with alcohol and otherrf as moderators
res <- rma(yi, vi, mods = ~ alcohol.rel + otherrf.rel, data=dat)
res

Mixed-Effects Model (k = 10; tau^2 estimator: REML)

tau^2 (estimated amount of residual heterogeneity):     0 (SE =
0.0445)
tau (square root of estimated tau^2 value):             0
I^2 (residual heterogeneity / unaccounted variability): 0.00%
H^2 (unaccounted variability / sampling variability):   1.00
```

```
R^2 (amount of heterogeneity accounted for):            0.00%

Test for Residual Heterogeneity:
QE(df = 7) = 4.3679, p-val = 0.7366

Test of Moderators (coefficients 2:3):
QM(df = 2) = 0.0956, p-val = 0.9533

Model Results:

             estimate      se     zval    pval    ci.lb   ci.ub
intrcpt        0.3590  0.1418   2.5314  0.0114   0.0810  0.6370  *
alcohol.rel    0.2902  1.9349   0.1500  0.8808  -3.5022  4.0826
otherrf.rel   -0.1437  1.5815  -0.0909  0.9276  -3.2434  2.9559

---
Signif. codes:  0 '***' 0.001 '**' 0.01 '*' 0.05 '.' 0.1 ' ' 1
```

Mixed-Effects Model for RR of TB Mortality Males vs. Females with Moderators = Alcohol + MDR

```
### fit mixed-effects model with alcohol and mdr as moderators
res <- rma(yi, vi, mods = ~ alcohol.rel + mdr.rel, data=dat)
res

Mixed-Effects Model (k = 10; tau^2 estimator: REML)

tau^2 (estimated amount of residual heterogeneity):     0 (SE =
0.0250)
tau (square root of estimated tau^2 value):             0
I^2 (residual heterogeneity / unaccounted variability): 0.00%
H^2 (unaccounted variability / sampling variability):   1.00
R^2 (amount of heterogeneity accounted for):            0.00%

Test for Residual Heterogeneity:
QE(df = 7) = 3.1920, p-val = 0.8667

Test of Moderators (coefficients 2:3):
QM(df = 2) = 3.4288, p-val = 0.1801

Model Results:

             estimate      se     zval    pval    ci.lb   ci.ub
intrcpt        0.2230  0.1269   1.7574  0.0789  -0.0257  0.4716  .
alcohol.rel    1.2575  0.6924   1.8161  0.0694  -0.0996  2.6147  .
mdr.rel       -0.5485  0.4127  -1.3291  0.1838  -1.3573  0.2603

---
Signif. codes:  0 '***' 0.001 '**' 0.01 '*' 0.05 '.' 0.1 ' ' 1
```

Mixed-Effects Model for RR of TB Mortality Males vs. Females with Moderators = Alcohol + MonoDR

```
### fit mixed-effects model with alcohol and monodr as moderators
res <- rma(yi, vi, mods = ~ alcohol.rel + monodr.rel, data=dat)
res

Mixed-Effects Model (k = 9; tau^2 estimator: REML)

tau^2 (estimated amount of residual heterogeneity):     0 (SE =
0.0377)
tau (square root of estimated tau^2 value):             0
```

```
I^2 (residual heterogeneity / unaccounted variability): 0.00%
H^2 (unaccounted variability / sampling variability):   1.00
R^2 (amount of heterogeneity accounted for):            0.00%

Test for Residual Heterogeneity:
QE(df = 6) = 4.8686, p-val = 0.5608

Test of Moderators (coefficients 2:3):
QM(df = 2) = 0.9782, p-val = 0.6132

Model Results:

             estimate      se     zval    pval    ci.lb   ci.ub
intrcpt        0.3591  0.1473   2.4378  0.0148   0.0704  0.6478  *
alcohol.rel    0.4266  0.6545   0.6518  0.5145  -0.8561  1.7094
monodr.rel    -0.3636  0.4391  -0.8281  0.4076  -1.2241  0.4969

---
Signif. codes:  0 ‘***’ 0.001 ‘**’ 0.01 ‘*’ 0.05 ‘.’ 0.1 ‘ ’ 1
```

Mixed-Effects Model for RR of TB Mortality Males vs. Females with Moderators = Nohome + Smoker

```
### fit mixed-effects model with nohome and smoker as moderators
res <- rma(yi, vi, mods = ~ nohome.rel + smoker.rel, data=dat)
res

Mixed-Effects Model (k = 5; tau^2 estimator: REML)

tau^2 (estimated amount of residual heterogeneity):     0 (SE =
0.0468)
tau (square root of estimated tau^2 value):             0
I^2 (residual heterogeneity / unaccounted variability): 0.00%
H^2 (unaccounted variability / sampling variability):   1.00
R^2 (amount of heterogeneity accounted for):            0.00%

Test for Residual Heterogeneity:
QE(df = 2) = 0.8326, p-val = 0.6595

Test of Moderators (coefficients 2:3):
QM(df = 2) = 0.6627, p-val = 0.7180

Model Results:

            estimate      se     zval    pval    ci.lb    ci.ub
intrcpt       0.3817  0.1889   2.0204  0.0433   0.0114   0.7520  *
nohome.rel    3.2228  6.5592   0.4913  0.6232  -9.6329  16.0785
smoker.rel   -0.1624  1.0114  -0.1606  0.8724  -2.1446   1.8198

---
Signif. codes:  0 ‘***’ 0.001 ‘**’ 0.01 ‘*’ 0.05 ‘.’ 0.1 ‘ ’ 1
```

Mixed-Effects Model for RR of TB Mortality Males vs. Females with Moderators = Nohome + Drugs

```
### fit mixed-effects model with nohome and drugs as moderators
res <- rma(yi, vi, mods = ~ nohome.rel + drugs.rel, data=dat)
res

Mixed-Effects Model (k = 8; tau^2 estimator: REML)
```

```
tau^2 (estimated amount of residual heterogeneity):     0 (SE = 
0.0260)
tau (square root of estimated tau^2 value):             0
I^2 (residual heterogeneity / unaccounted variability): 0.00%
H^2 (unaccounted variability / sampling variability):   1.00
R^2 (amount of heterogeneity accounted for):            100.00%

Test for Residual Heterogeneity:
QE(df = 5) = 2.4623, p-val = 0.7822

Test of Moderators (coefficients 2:3):
QM(df = 2) = 2.9322, p-val = 0.2308

Model Results:

            estimate       se    zval    pval     ci.lb    ci.ub
intrcpt       0.2677   0.1079  2.4816  0.0131    0.0563   0.4791  *
nohome.rel    0.7551  10.3238  0.0731  0.9417  -19.4791  20.9893
drugs.rel     1.0242   3.6764  0.2786  0.7806   -6.1815   8.2299

---
Signif. codes:  0 ‘***’ 0.001 ‘**’ 0.01 ‘*’ 0.05 ‘.’ 0.1 ‘ ’ 1
```

Mixed-Effects Model for RR of TB Mortality Males vs. Females with Moderators = Nohome + Migrant

```
### fit mixed-effects model with nohome and migrant as moderators
res <- rma(yi, vi, mods = ~ nohome.rel + migrant.rel, data=dat)
res

Mixed-Effects Model (k = 5; tau^2 estimator: REML)

tau^2 (estimated amount of residual heterogeneity):     0 (SE = 
0.0398)
tau (square root of estimated tau^2 value):             0
I^2 (residual heterogeneity / unaccounted variability): 0.00%
H^2 (unaccounted variability / sampling variability):   1.00
R^2 (amount of heterogeneity accounted for):            0.00%

Test for Residual Heterogeneity:
QE(df = 2) = 0.8199, p-val = 0.6637

Test of Moderators (coefficients 2:3):
QM(df = 2) = 1.1365, p-val = 0.5665

Model Results:

             estimate      se    zval    pval    ci.lb    ci.ub
intrcpt        0.1644  0.2127  0.7729  0.4396  -0.2525   0.5814
nohome.rel     3.7001  3.5369  1.0462  0.2955  -3.2320  10.6322
migrant.rel    0.1441  0.4962  0.2903  0.7716  -0.8285   1.1167

---
Signif. codes:  0 ‘***’ 0.001 ‘**’ 0.01 ‘*’ 0.05 ‘.’ 0.1 ‘ ’ 1
```

Mixed-Effects Model for RR of TB Mortality Males vs. Females with Moderators = Nohome + Formertb

```
### fit mixed-effects model with nohome and formertb as moderators
res <- rma(yi, vi, mods = ~ nohome.rel + formertb.rel, data=dat)
res
```

```
Mixed-Effects Model (k = 7; tau^2 estimator: REML)

tau^2 (estimated amount of residual heterogeneity):     0 (SE =
0.0273)
tau (square root of estimated tau^2 value):             0
I^2 (residual heterogeneity / unaccounted variability): 0.00%
H^2 (unaccounted variability / sampling variability):   1.00
R^2 (amount of heterogeneity accounted for):            100.00%

Test for Residual Heterogeneity:
QE(df = 4) = 1.6209, p-val = 0.8050

Test of Moderators (coefficients 2:3):
QM(df = 2) = 2.4805, p-val = 0.2893

Model Results:

              estimate      se     zval    pval    ci.lb   ci.ub
intrcpt         0.2942  0.1213   2.4244  0.0153   0.0563  0.5320  *
nohome.rel      3.9599  2.6315   1.5048  0.1324  -1.1977  9.1176
formertb.rel   -0.1756  0.4674  -0.3757  0.7071  -1.0917  0.7405

---
Signif. codes:  0 ‘***’ 0.001 ‘**’ 0.01 ‘*’ 0.05 ‘.’ 0.1 ‘ ’ 1
```

Mixed-Effects Model for RR of TB Mortality Males vs. Females with Moderators = Nohome + Prison

```
### fit mixed-effects model with nohome and prison as moderators
res <- rma(yi, vi, mods = ~ nohome.rel + prison.rel, data=dat)
res

Mixed-Effects Model (k = 6; tau^2 estimator: REML)

tau^2 (estimated amount of residual heterogeneity):     0 (SE =
0.0370)
tau (square root of estimated tau^2 value):             0
I^2 (residual heterogeneity / unaccounted variability): 0.00%
H^2 (unaccounted variability / sampling variability):   1.00
R^2 (amount of heterogeneity accounted for):            0.00%

Test for Residual Heterogeneity:
QE(df = 3) = 2.3800, p-val = 0.4974

Test of Moderators (coefficients 2:3):
QM(df = 2) = 1.6910, p-val = 0.4293

Model Results:

            estimate      se     zval    pval     ci.lb    ci.ub
intrcpt       0.3173  0.2429   1.3065  0.1914   -0.1587   0.7933
nohome.rel    3.1351  2.4142   1.2986  0.1941   -1.5966   7.8667
prison.rel   -0.6079  6.4263  -0.0946  0.9246  -13.2033  11.9874

---
Signif. codes:  0 ‘***’ 0.001 ‘**’ 0.01 ‘*’ 0.05 ‘.’ 0.1 ‘ ’ 1
```

Mixed-Effects Model for RR of TB Mortality Males vs. Females with Moderators = Nohome + Otherrf

```
### fit mixed-effects model with nohome and otherrf as moderators
res <- rma(yi, vi, mods = ~ nohome.rel + otherrf.rel, data=dat)
```

```
res

Mixed-Effects Model (k = 5; tau^2 estimator: REML)

tau^2 (estimated amount of residual heterogeneity):     0 (SE =
0.1035)
tau (square root of estimated tau^2 value):             0
I^2 (residual heterogeneity / unaccounted variability): 0.00%
H^2 (unaccounted variability / sampling variability):   1.00
R^2 (amount of heterogeneity accounted for):            0.00%

Test for Residual Heterogeneity:
QE(df = 2) = 0.1347, p-val = 0.9349

Test of Moderators (coefficients 2:3):
QM(df = 2) = 1.4798, p-val = 0.4772

Model Results:

             estimate       se     zval    pval     ci.lb    ci.ub
intrcpt        0.6433   0.2740   2.3478  0.0189    0.1063   1.1803  *
nohome.rel   -25.7846  21.6427  -1.1914  0.2335  -68.2034  16.6342
otherrf.rel    2.5262   2.0767   1.2165  0.2238   -1.5440   6.5964

---
Signif. codes:  0 '***' 0.001 '**' 0.01 '*' 0.05 '.' 0.1 ' ' 1
```

Mixed-Effects Model for RR of TB Mortality Males vs. Females with Moderators = Nohome + MDR

```
### fit mixed-effects model with nohome and mdr as moderators
res <- rma(yi, vi, mods = ~ nohome.rel + mdr.rel, data=dat)
res

Mixed-Effects Model (k = 7; tau^2 estimator: REML)

tau^2 (estimated amount of residual heterogeneity):     0 (SE =
0.0335)
tau (square root of estimated tau^2 value):             0
I^2 (residual heterogeneity / unaccounted variability): 0.00%
H^2 (unaccounted variability / sampling variability):   1.00
R^2 (amount of heterogeneity accounted for):            0.00%

Test for Residual Heterogeneity:
QE(df = 4) = 2.5884, p-val = 0.6289

Test of Moderators (coefficients 2:3):
QM(df = 2) = 1.7878, p-val = 0.4091

Model Results:

            estimate      se     zval    pval    ci.lb   ci.ub
intrcpt       0.2713  0.1440   1.8838  0.0596  -0.0110  0.5536  .
nohome.rel    3.5550  2.7771   1.2801  0.2005  -1.8880  8.9981
mdr.rel      -0.0345  0.2457  -0.1405  0.8883  -0.5160  0.4470

---
Signif. codes:  0 '***' 0.001 '**' 0.01 '*' 0.05 '.' 0.1 ' ' 1
```

Mixed-Effects Model for RR of TB Mortality Males vs. Females with Moderators = Nohome + MonoDR

```
### fit mixed-effects model with nohome and monodr as moderators
res <- rma(yi, vi, mods = ~ nohome.rel + monodr.rel, data=dat)
res

Mixed-Effects Model (k = 5; tau^2 estimator: REML)

tau^2 (estimated amount of residual heterogeneity):     0.0077 (SE =
0.0663)
tau (square root of estimated tau^2 value):             0.0878
I^2 (residual heterogeneity / unaccounted variability): 11.14%
H^2 (unaccounted variability / sampling variability):   1.13
R^2 (amount of heterogeneity accounted for):            52.04%

Test for Residual Heterogeneity:
QE(df = 2) = 2.7776, p-val = 0.2494

Test of Moderators (coefficients 2:3):
QM(df = 2) = 1.0659, p-val = 0.5869

Model Results:

            estimate      se    zval    pval    ci.lb    ci.ub
intrcpt       0.2418  0.3854  0.6274  0.5304  -0.5135   0.9971
nohome.rel    3.3347  3.4098  0.9780  0.3281  -3.3485  10.0178
monodr.rel    0.7544  4.5071  0.1674  0.8671  -8.0794   9.5882

---
Signif. codes:  0 '***' 0.001 '**' 0.01 '*' 0.05 '.' 0.1 ' ' 1
```

Mixed-Effects Model for RR of TB Mortality Males vs. Females with Moderators = Smoker + Drugs

```
### fit mixed-effects model with smoker and drugs as moderators
res <- rma(yi, vi, mods = ~ smoker.rel + drugs.rel, data=dat)
res

Mixed-Effects Model (k = 7; tau^2 estimator: REML)

tau^2 (estimated amount of residual heterogeneity):     0 (SE =
0.0415)
tau (square root of estimated tau^2 value):             0
I^2 (residual heterogeneity / unaccounted variability): 0.00%
H^2 (unaccounted variability / sampling variability):   1.00
R^2 (amount of heterogeneity accounted for):            0.00%

Test for Residual Heterogeneity:
QE(df = 4) = 1.4650, p-val = 0.8328

Test of Moderators (coefficients 2:3):
QM(df = 2) = 2.8109, p-val = 0.2453

Model Results:

            estimate      se     zval    pval    ci.lb    ci.ub
intrcpt       0.5403  0.2391   2.2601  0.0238   0.0717   1.0088  *
smoker.rel   -2.3725  1.8082  -1.3121  0.1895  -5.9164   1.1715
drugs.rel     5.4413  3.4648   1.5704  0.1163  -1.3496  12.2322

---
Signif. codes:  0 '***' 0.001 '**' 0.01 '*' 0.05 '.' 0.1 ' ' 1
```

Mixed-Effects Model for RR of TB Mortality Males vs. Females with Moderators = Smoker + Migrant

```
### fit mixed-effects model with smoker and migrant as moderators
res <- rma(yi, vi, mods = ~ smoker.rel + migrant.rel, data=dat)
res

Warning messages:
1: Studies with NAs omitted from model fitting.
2: Redundant predictors dropped from the model.

Random-Effects Model (k = 1; tau^2 estimator: REML)

tau^2 (estimated amount of total heterogeneity): 0
tau (square root of estimated tau^2 value):      0
I^2 (total heterogeneity / total variability):   0.00%
H^2 (total variability / sampling variability):  1.00

Test for Heterogeneity:
Q(df = 0) = 0.0000, p-val = 1.0000

Model Results:

estimate      se    zval    pval    ci.lb   ci.ub
  0.3079  0.2260  1.3626  0.1730  -0.1350  0.7509

---
Signif. codes:  0 ‘***’ 0.001 ‘**’ 0.01 ‘*’ 0.05 ‘.’ 0.1 ‘ ’ 1
```

Mixed-Effects Model for RR of TB Mortality Males vs. Females with Moderators = Smoker + Formertb

```
### fit mixed-effects model with smoker and formertb as moderators
res <- rma(yi, vi, mods = ~ smoker.rel + formertb.rel, data=dat)
res

Mixed-Effects Model (k = 8; tau^2 estimator: REML)

tau^2 (estimated amount of residual heterogeneity):     0.0005 (SE =
0.0483)
tau (square root of estimated tau^2 value):             0.0220
I^2 (residual heterogeneity / unaccounted variability): 0.53%
H^2 (unaccounted variability / sampling variability):   1.01
R^2 (amount of heterogeneity accounted for):            0.00%

Test for Residual Heterogeneity:
QE(df = 5) = 4.3122, p-val = 0.5054

Test of Moderators (coefficients 2:3):
QM(df = 2) = 0.5133, p-val = 0.7736

Model Results:

              estimate      se     zval    pval    ci.lb   ci.ub
intrcpt         0.3335  0.2450   1.3612  0.1735  -0.1467  0.8138
smoker.rel      0.4969  0.7602   0.6536  0.5134  -0.9932  1.9869
formertb.rel   -0.4311  0.6309  -0.6833  0.4944  -1.6676  0.8054

---
Signif. codes:  0 ‘***’ 0.001 ‘**’ 0.01 ‘*’ 0.05 ‘.’ 0.1 ‘ ’ 1
```

Mixed-Effects Model for RR of TB Mortality Males vs. Females with Moderators = Smoker + Prison

```
### fit mixed-effects model with smoker and prison as moderators
res <- rma(yi, vi, mods = ~ smoker.rel + prison.rel, data=dat)
res

Mixed-Effects Model (k = 5; tau^2 estimator: REML)

tau^2 (estimated amount of residual heterogeneity):     0 (SE = 
0.0461)
tau (square root of estimated tau^2 value):             0
I^2 (residual heterogeneity / unaccounted variability): 0.00%
H^2 (unaccounted variability / sampling variability):   1.00
R^2 (amount of heterogeneity accounted for):            0.00%

Test for Residual Heterogeneity:
QE(df = 2) = 0.2593, p-val = 0.8784

Test of Moderators (coefficients 2:3):
QM(df = 2) = 1.5846, p-val = 0.4528

Model Results:

            estimate      se     zval    pval    ci.lb   ci.ub
intrcpt       0.2877  0.2339   1.2301  0.2186  -0.1707  0.7461
smoker.rel    0.7083  0.6094   1.1623  0.2451  -0.4861  1.9027
prison.rel   -0.9268  2.3680  -0.3914  0.6955  -5.5681  3.7144

---
Signif. codes:  0 ‘***’ 0.001 ‘**’ 0.01 ‘*’ 0.05 ‘.’ 0.1 ‘ ’ 1
```

Mixed-Effects Model for RR of TB Mortality Males vs. Females with Moderators = Smoker + Otherrf

```
### fit mixed-effects model with smoker and otherrf as moderators
res <- rma(yi, vi, mods = ~ smoker.rel + otherrf.rel, data=dat)
res

Mixed-Effects Model (k = 7; tau^2 estimator: REML)

tau^2 (estimated amount of residual heterogeneity):     0 (SE = 
0.0895)
tau (square root of estimated tau^2 value):             0
I^2 (residual heterogeneity / unaccounted variability): 0.00%
H^2 (unaccounted variability / sampling variability):   1.00
R^2 (amount of heterogeneity accounted for):            0.00%

Test for Residual Heterogeneity:
QE(df = 4) = 1.3357, p-val = 0.8553

Test of Moderators (coefficients 2:3):
QM(df = 2) = 1.7852, p-val = 0.4096

Model Results:

             estimate      se     zval    pval    ci.lb   ci.ub
intrcpt        0.3606  0.2162   1.6684  0.0952  -0.0630  0.7843  .
smoker.rel    -1.0586  1.0055  -1.0528  0.2924  -3.0293  0.9121
otherrf.rel    1.0800  0.8197   1.3176  0.1876  -0.5265  2.6865

---
Signif. codes:  0 ‘***’ 0.001 ‘**’ 0.01 ‘*’ 0.05 ‘.’ 0.1 ‘ ’ 1
```

Mixed-Effects Model for RR of TB Mortality Males vs. Females with Moderators = Smoker + MDR

```
### fit mixed-effects model with smoker and mdr as moderators
res <- rma(yi, vi, mods = ~ smoker.rel + mdr.rel, data=dat)
res

Mixed-Effects Model (k = 6; tau^2 estimator: REML)

tau^2 (estimated amount of residual heterogeneity):     0 (SE =
0.0395)
tau (square root of estimated tau^2 value):             0
I^2 (residual heterogeneity / unaccounted variability): 0.00%
H^2 (unaccounted variability / sampling variability):   1.00
R^2 (amount of heterogeneity accounted for):            0.00%

Test for Residual Heterogeneity:
QE(df = 3) = 2.9633, p-val = 0.3973

Test of Moderators (coefficients 2:3):
QM(df = 2) = 1.0511, p-val = 0.5912

Model Results:

            estimate      se     zval    pval    ci.lb   ci.ub
intrcpt       0.2370  0.2054   1.1539  0.2486  -0.1655  0.6395
smoker.rel    0.5909  0.6003   0.9844  0.3249  -0.5856  1.7674
mdr.rel      -0.1370  0.3131  -0.4375  0.6617  -0.7506  0.4766

---
Signif. codes:  0 '***' 0.001 '**' 0.01 '*' 0.05 '.' 0.1 ' ' 1
```

Mixed-Effects Model for RR of TB Mortality Males vs. Females with Moderators = Smoker + MonoDR

```
### fit mixed-effects model with smoker and monodr as moderators
res <- rma(yi, vi, mods = ~ smoker.rel + monodr.rel, data=dat)
res

Mixed-Effects Model (k = 5; tau^2 estimator: REML)

tau^2 (estimated amount of residual heterogeneity):     0 (SE =
0.0805)
tau (square root of estimated tau^2 value):             0
I^2 (residual heterogeneity / unaccounted variability): 0.00%
H^2 (unaccounted variability / sampling variability):   1.00
R^2 (amount of heterogeneity accounted for):            0.00%

Test for Residual Heterogeneity:
QE(df = 2) = 0.6006, p-val = 0.7406

Test of Moderators (coefficients 2:3):
QM(df = 2) = 2.2229, p-val = 0.3291

Model Results:

            estimate      se     zval    pval     ci.lb   ci.ub
intrcpt       0.8527  0.3940   2.1642  0.0304    0.0805  1.6250  *
smoker.rel    0.2366  0.5392   0.4389  0.6607   -0.8202  1.2934
monodr.rel   -9.5566  6.4822  -1.4743  0.1404  -22.2614  3.1483

---
Signif. codes:  0 '***' 0.001 '**' 0.01 '*' 0.05 '.' 0.1 ' ' 1
```

Mixed-Effects Model for RR of TB Mortality Males vs. Females with Moderators = Drugs + Migrant

```
### fit mixed-effects model with drugs and migrant as moderators
res <- rma(yi, vi, mods = ~ drugs.rel + migrant.rel, data=dat)
res

Mixed-Effects Model (k = 6; tau^2 estimator: REML)

tau^2 (estimated amount of residual heterogeneity):     0 (SE =
0.0378)
tau (square root of estimated tau^2 value):             0
I^2 (residual heterogeneity / unaccounted variability): 0.00%
H^2 (unaccounted variability / sampling variability):   1.00
R^2 (amount of heterogeneity accounted for):            0.00%

Test for Residual Heterogeneity:
QE(df = 3) = 1.0283, p-val = 0.7944

Test of Moderators (coefficients 2:3):
QM(df = 2) = 1.1547, p-val = 0.5614

Model Results:

             estimate      se     zval    pval    ci.lb   ci.ub
intrcpt        0.3150  0.1459   2.1584  0.0309   0.0290  0.6011  *
drugs.rel      1.0504  1.1031   0.9522  0.3410  -1.1116  3.2124
migrant.rel   -0.2207  0.4144  -0.5327  0.5943  -1.0329  0.5915

---
Signif. codes:  0 '***' 0.001 '**' 0.01 '*' 0.05 '.' 0.1 ' ' 1
```

Mixed-Effects Model for RR of TB Mortality Males vs. Females with Moderators = Drugs + Formertb

```
### fit mixed-effects model with drugs and formertb as moderators
res <- rma(yi, vi, mods = ~ drugs.rel + formertb.rel, data=dat)
res

Mixed-Effects Model (k = 10; tau^2 estimator: REML)

tau^2 (estimated amount of residual heterogeneity):     0 (SE =
0.0179)
tau (square root of estimated tau^2 value):             0
I^2 (residual heterogeneity / unaccounted variability): 0.00%
H^2 (unaccounted variability / sampling variability):   1.00
R^2 (amount of heterogeneity accounted for):            100.00%

Test for Residual Heterogeneity:
QE(df = 7) = 3.0065, p-val = 0.8844

Test of Moderators (coefficients 2:3):
QM(df = 2) = 4.3475, p-val = 0.1138

Model Results:

              estimate      se     zval    pval    ci.lb    ci.ub
intrcpt         0.4270  0.0903   4.7308  <.0001   0.2501   0.6039
***
drugs.rel       1.5284  0.9970   1.5330  0.1253  -0.4257   3.4825
formertb.rel   -1.2788  0.6284  -2.0351  0.0418  -2.5103  -0.0472
*
```

```
---
Signif. codes:  0 ‘***’ 0.001 ‘**’ 0.01 ‘*’ 0.05 ‘.’ 0.1 ‘ ’ 1
```

Mixed-Effects Model for RR of TB Mortality Males vs. Females with Moderators = Drugs + Prison

```
### fit mixed-effects model with drugs and prison as moderators
res <- rma(yi, vi, mods = ~ drugs.rel + prison.rel, data=dat)
res

Mixed-Effects Model (k = 8; tau^2 estimator: REML)

tau^2 (estimated amount of residual heterogeneity):     0 (SE =
0.0271)
tau (square root of estimated tau^2 value):             0
I^2 (residual heterogeneity / unaccounted variability): 0.00%
H^2 (unaccounted variability / sampling variability):   1.00
R^2 (amount of heterogeneity accounted for):            0.00%

Test for Residual Heterogeneity:
QE(df = 5) = 1.8589, p-val = 0.8683

Test of Moderators (coefficients 2:3):
QM(df = 2) = 2.0722, p-val = 0.3548

Model Results:

            estimate      se    zval    pval    ci.lb   ci.ub
intrcpt       0.3179  0.1472  2.1593  0.0308   0.0293  0.6064  *
drugs.rel     1.1097  0.7737  1.4344  0.1515  -0.4066  2.6261
prison.rel    0.0064  2.2062  0.0029  0.9977  -4.3176  4.3304

---
Signif. codes:  0 ‘***’ 0.001 ‘**’ 0.01 ‘*’ 0.05 ‘.’ 0.1 ‘ ’ 1
```

Mixed-Effects Model for RR of TB Mortality Males vs. Females with Moderators = Drugs + Otherrf

```
### fit mixed-effects model with drugs and otherrf as moderators
res <- rma(yi, vi, mods = ~ drugs.rel + otherrf.rel, data=dat)
res

Mixed-Effects Model (k = 7; tau^2 estimator: REML)

tau^2 (estimated amount of residual heterogeneity):     0 (SE =
0.0529)
tau (square root of estimated tau^2 value):             0
I^2 (residual heterogeneity / unaccounted variability): 0.00%
H^2 (unaccounted variability / sampling variability):   1.00
R^2 (amount of heterogeneity accounted for):            0.00%

Test for Residual Heterogeneity:
QE(df = 4) = 1.9780, p-val = 0.7398

Test of Moderators (coefficients 2:3):
QM(df = 2) = 0.5974, p-val = 0.7418

Model Results:

             estimate      se     zval    pval    ci.lb   ci.ub
intrcpt        0.4574  0.1980   2.3105  0.0209   0.0694  0.8454  *
drugs.rel     -1.1669  2.6172  -0.4459  0.6557  -6.2965  3.9626
```

```
otherrf.rel   -0.5764  0.9574  -0.6020  0.5472  -2.4529  1.3001

---
Signif. codes:  0 ‘***’ 0.001 ‘**’ 0.01 ‘*’ 0.05 ‘.’ 0.1 ‘ ’ 1
```

Mixed-Effects Model for RR of TB Mortality Males vs. Females with Moderators = Drugs + MDR

```
### fit mixed-effects model with drugs and mdr as moderators
res <- rma(yi, vi, mods = ~ drugs.rel + mdr.rel, data=dat)
res

Mixed-Effects Model (k = 9; tau^2 estimator: REML)

tau^2 (estimated amount of residual heterogeneity):     0.0000 (SE = 0.0287)
tau (square root of estimated tau^2 value):             0.0006
I^2 (residual heterogeneity / unaccounted variability): 0.00%
H^2 (unaccounted variability / sampling variability):   1.00
R^2 (amount of heterogeneity accounted for):            48.20%

Test for Residual Heterogeneity:
QE(df = 6) = 6.4727, p-val = 0.3724

Test of Moderators (coefficients 2:3):
QM(df = 2) = 2.0147, p-val = 0.3652

Model Results:

           estimate      se     zval    pval    ci.lb   ci.ub
intrcpt      0.3783  0.1419   2.6653  0.0077   0.1001  0.6564  **
drugs.rel    0.9963  0.9682   1.0290  0.3035  -0.9013  2.8939
mdr.rel     -1.6438  2.0503  -0.8017  0.4227  -5.6624  2.3747

---
Signif. codes:  0 ‘***’ 0.001 ‘**’ 0.01 ‘*’ 0.05 ‘.’ 0.1 ‘ ’ 1
```

Mixed-Effects Model for RR of TB Mortality Males vs. Females with Moderators = Drugs + MonoDR

```
### fit mixed-effects model with drugs and monodr as moderators
res <- rma(yi, vi, mods = ~ drugs.rel + monodr.rel, data=dat)
res

Mixed-Effects Model (k = 7; tau^2 estimator: REML)

tau^2 (estimated amount of residual heterogeneity):     0.0148 (SE = 0.0503)
tau (square root of estimated tau^2 value):             0.1217
I^2 (residual heterogeneity / unaccounted variability): 19.71%
H^2 (unaccounted variability / sampling variability):   1.25
R^2 (amount of heterogeneity accounted for):            0.00%

Test for Residual Heterogeneity:
QE(df = 4) = 4.0738, p-val = 0.3961

Test of Moderators (coefficients 2:3):
QM(df = 2) = 0.6661, p-val = 0.7167

Model Results:

            estimate      se     zval     pval     ci.lb   ci.ub
```

```
intrcpt       0.4287  0.1497   2.8630  0.0042   0.1352  0.7222  **
drugs.rel    -0.0398  0.3251  -0.1224  0.9026  -0.6771  0.5975
monodr.rel   -0.3683  0.4748  -0.7756  0.4380  -1.2989  0.5624

---
Signif. codes:  0 '***' 0.001 '**' 0.01 '*' 0.05 '.' 0.1 ' ' 1
```

Mixed-Effects Model for RR of TB Mortality Males vs. Females with Moderators = Migrant + Formertb

```
### fit mixed-effects model with migrant and formertb as moderators
res <- rma(yi, vi, mods = ~ migrant.rel + formertb.rel, data=dat)
res

Mixed-Effects Model (k = 7; tau^2 estimator: REML)

tau^2 (estimated amount of residual heterogeneity):     0 (SE =
0.0042)
tau (square root of estimated tau^2 value):             0
I^2 (residual heterogeneity / unaccounted variability): 0.00%
H^2 (unaccounted variability / sampling variability):   1.00
R^2 (amount of heterogeneity accounted for):            100.00%

Test for Residual Heterogeneity:
QE(df = 4) = 2.0198, p-val = 0.7321

Test of Moderators (coefficients 2:3):
QM(df = 2) = 5.1775, p-val = 0.0751

Model Results:

              estimate      se     zval    pval    ci.lb    ci.ub
intrcpt         0.7263  0.2146   3.3845  0.0007   0.3057   1.1469
***
migrant.rel    -0.5990  0.2679  -2.2360  0.0254  -1.1241  -0.0740
*
formertb.rel   -0.1826  1.3756  -0.1327  0.8944  -2.8786   2.5135

---
Signif. codes:  0 '***' 0.001 '**' 0.01 '*' 0.05 '.' 0.1 ' ' 1
```

Mixed-Effects Model for RR of TB Mortality Males vs. Females with Moderators = Migrant + Prison

```
### fit mixed-effects model with migrant and prison as moderators
res <- rma(yi, vi, mods = ~ migrant.rel + prison.rel, data=dat)
res

Error in rma(yi, vi, mods = ~migrant.rel + prison.rel, data = dat) :
  Number of parameters to be estimated is larger than the number of
observations.
In addition: Warning message:
Studies with NAs omitted from model fitting.
```

Mixed-Effects Model for RR of TB Mortality Males vs. Females with Moderators = Migrant + Otherrf

```
### fit mixed-effects model with migrant and otherrf as moderators
res <- rma(yi, vi, mods = ~ migrant.rel + otherrf.rel, data=dat)
res
```

```
Mixed-Effects Model (k = 4; tau^2 estimator: REML)

tau^2 (estimated amount of residual heterogeneity):     0 (SE =
0.1200)
tau (square root of estimated tau^2 value):             0
I^2 (residual heterogeneity / unaccounted variability): 0.00%
H^2 (unaccounted variability / sampling variability):   1.00
R^2 (amount of heterogeneity accounted for):            0.00%

Test for Residual Heterogeneity:
QE(df = 1) = 0.2724, p-val = 0.6018

Test of Moderators (coefficients 2:3):
QM(df = 2) = 0.3525, p-val = 0.8384

Model Results:

             estimate      se     zval    pval     ci.lb    ci.ub
intrcpt        0.4024  0.3014   1.3349  0.1819   -0.1884   0.9932
migrant.rel    0.4041  0.7004   0.5770  0.5640   -0.9687   1.7769
otherrf.rel   -0.6514  6.5871  -0.0989  0.9212  -13.5619  12.2592

---
Signif. codes:  0 ‘***’ 0.001 ‘**’ 0.01 ‘*’ 0.05 ‘.’ 0.1 ‘ ’ 1
```

Mixed-Effects Model for RR of TB Mortality Males vs. Females with Moderators = Migrant + MDR

```
### fit mixed-effects model with migrant and mdr as moderators
res <- rma(yi, vi, mods = ~ migrant.rel + mdr.rel, data=dat)
res

Mixed-Effects Model (k = 5; tau^2 estimator: REML)

tau^2 (estimated amount of residual heterogeneity):     0 (SE =
0.0441)
tau (square root of estimated tau^2 value):             0
I^2 (residual heterogeneity / unaccounted variability): 0.00%
H^2 (unaccounted variability / sampling variability):   1.00
R^2 (amount of heterogeneity accounted for):            0.00%

Test for Residual Heterogeneity:
QE(df = 2) = 1.4813, p-val = 0.4768

Test of Moderators (coefficients 2:3):
QM(df = 2) = 0.3331, p-val = 0.8466

Model Results:

             estimate      se     zval    pval    ci.lb   ci.ub
intrcpt        0.3315  0.1558   2.1280  0.0333   0.0262  0.6368  *
migrant.rel    0.0090  0.2413   0.0374  0.9702  -0.4639  0.4820
mdr.rel       -1.8894  3.3130  -0.5703  0.5685  -8.3827  4.6039

---
Signif. codes:  0 ‘***’ 0.001 ‘**’ 0.01 ‘*’ 0.05 ‘.’ 0.1 ‘ ’ 1
```

Mixed-Effects Model for RR of TB Mortality Males vs. Females with Moderators = Migrant + MonoDR

```
### fit mixed-effects model with migrant and monodr as moderators
res <- rma(yi, vi, mods = ~ migrant.rel + monodr.rel, data=dat)
```

```
res

Error in rma(yi, vi, mods = ~migrant.rel + monodr.rel, data = dat) :
  Number of parameters to be estimated is larger than the number of
observations.
In addition: Warning message:
Studies with NAs omitted from model fitting.
```

Mixed-Effects Model for RR of TB Mortality Males vs. Females with Moderators = Formertb + Prison

```
### fit mixed-effects model with formertb and prison as moderators
res <- rma(yi, vi, mods = ~ formertb.rel + prison.rel, data=dat)
res

Mixed-Effects Model (k = 5; tau^2 estimator: REML)

tau^2 (estimated amount of residual heterogeneity):     0.0174 (SE =
0.0597)
tau (square root of estimated tau^2 value):             0.1320
I^2 (residual heterogeneity / unaccounted variability): 28.56%
H^2 (unaccounted variability / sampling variability):   1.40
R^2 (amount of heterogeneity accounted for):            21.86%

Test for Residual Heterogeneity:
QE(df = 2) = 2.5006, p-val = 0.2864

Test of Moderators (coefficients 2:3):
QM(df = 2) = 1.1165, p-val = 0.5722

Model Results:

              estimate      se     zval    pval    ci.lb   ci.ub
intrcpt         0.5249  0.2040   2.5726  0.0101   0.1250  0.9248  *
formertb.rel   -1.0300  0.9797  -1.0513  0.2931  -2.9503  0.8902
prison.rel      0.3450  2.2508   0.1533  0.8782  -4.0664  4.7564

---
Signif. codes:  0 ‘***’ 0.001 ‘**’ 0.01 ‘*’ 0.05 ‘.’ 0.1 ‘ ’ 1
```

Mixed-Effects Model for RR of TB Mortality Males vs. Females with Moderators = Formertb + Otherrf

```
### fit mixed-effects model with formertb and otherrf as moderators
res <- rma(yi, vi, mods = ~ formertb.rel + otherrf.rel, data=dat)
res

Mixed-Effects Model (k = 9; tau^2 estimator: REML)

tau^2 (estimated amount of residual heterogeneity):     0 (SE =
0.0351)
tau (square root of estimated tau^2 value):             0
I^2 (residual heterogeneity / unaccounted variability): 0.00%
H^2 (unaccounted variability / sampling variability):   1.00
R^2 (amount of heterogeneity accounted for):            100.00%

Test for Residual Heterogeneity:
QE(df = 6) = 4.0331, p-val = 0.6722

Test of Moderators (coefficients 2:3):
QM(df = 2) = 1.0991, p-val = 0.5772
```

```
Model Results:

               estimate      se     zval    pval    ci.lb   ci.ub
intrcpt          0.1812  0.1845   0.9825  0.3259  -0.1803  0.5428
formertb.rel     0.7214  0.7021   1.0276  0.3042  -0.6546  2.0975
otherrf.rel     -0.2859  0.5443  -0.5253  0.5994  -1.3528  0.7809

---
Signif. codes:  0 '***' 0.001 '**' 0.01 '*' 0.05 '.' 0.1 ' ' 1
```

Mixed-Effects Model for RR of TB Mortality Males vs. Females with Moderators = Formertb + MDR

```
### fit mixed-effects model with formertb and mdr as moderators
res <- rma(yi, vi, mods = ~ formertb.rel + mdr.rel, data=dat)
res

Mixed-Effects Model (k = 8; tau^2 estimator: REML)

tau^2 (estimated amount of residual heterogeneity):     0.0085 (SE =
0.0227)
tau (square root of estimated tau^2 value):             0.0922
I^2 (residual heterogeneity / unaccounted variability): 21.89%
H^2 (unaccounted variability / sampling variability):   1.28
R^2 (amount of heterogeneity accounted for):            0.00%

Test for Residual Heterogeneity:
QE(df = 5) = 5.3565, p-val = 0.3739

Test of Moderators (coefficients 2:3):
QM(df = 2) = 0.5371, p-val = 0.7645

Model Results:

               estimate      se     zval    pval    ci.lb   ci.ub
intrcpt          0.4186  0.1271   3.2938  0.0010   0.1695  0.6678  ***
formertb.rel    -0.6435  1.2470  -0.5160  0.6058  -3.0876  1.8006
mdr.rel          0.4811  0.7277   0.6611  0.5085  -0.9452  1.9075

---
Signif. codes:  0 '***' 0.001 '**' 0.01 '*' 0.05 '.' 0.1 ' ' 1
```

Mixed-Effects Model for RR of TB Mortality Males vs. Females with Moderators = Formertb + MonoDR

```
### fit mixed-effects model with formertb and monodr as moderators
res <- rma(yi, vi, mods = ~ formertb.rel + monodr.rel, data=dat)
res

Mixed-Effects Model (k = 6; tau^2 estimator: REML)

tau^2 (estimated amount of residual heterogeneity):     0 (SE =
0.0434)
tau (square root of estimated tau^2 value):             0
I^2 (residual heterogeneity / unaccounted variability): 0.00%
H^2 (unaccounted variability / sampling variability):   1.00
R^2 (amount of heterogeneity accounted for):            100.00%

Test for Residual Heterogeneity:
QE(df = 3) = 1.8085, p-val = 0.6131

Test of Moderators (coefficients 2:3):
```

```
QM(df = 2) = 2.6214, p-val = 0.2696

Model Results:

              estimate      se     zval    pval     ci.lb   ci.ub
intrcpt         1.0078  0.4117   2.4479  0.0144    0.2009  1.8147  *
formertb.rel   -4.4467  3.0610  -1.4527  0.1463  -10.4461  1.5527
monodr.rel      2.0376  1.6698   1.2203  0.2224   -1.2351  5.3104

---
Signif. codes:  0 ‘***’ 0.001 ‘**’ 0.01 ‘*’ 0.05 ‘.’ 0.1 ‘ ’ 1
```

Mixed-Effects Model for RR of TB Mortality Males vs. Females with Moderators = Prison + Otherrf

```
### fit mixed-effects model with prison and otherrf as moderators
res <- rma(yi, vi, mods = ~ prison.rel + otherrf.rel, data=dat)
res

Mixed-Effects Model (k = 5; tau^2 estimator: REML)

tau^2 (estimated amount of residual heterogeneity):     0 (SE =
0.0985)
tau (square root of estimated tau^2 value):             0
I^2 (residual heterogeneity / unaccounted variability): 0.00%
H^2 (unaccounted variability / sampling variability):   1.00
R^2 (amount of heterogeneity accounted for):            0.00%

Test for Residual Heterogeneity:
QE(df = 2) = 1.0815, p-val = 0.5823

Test of Moderators (coefficients 2:3):
QM(df = 2) = 0.7237, p-val = 0.6964

Model Results:

             estimate      se     zval    pval    ci.lb   ci.ub
intrcpt        0.4005  0.2206   1.8156  0.0694  -0.0319  0.8329  .
prison.rel     0.3849  2.1024   0.1831  0.8547  -3.7358  4.5056
otherrf.rel   -0.3867  0.4854  -0.7967  0.4256  -1.3381  0.5647

---
Signif. codes:  0 ‘***’ 0.001 ‘**’ 0.01 ‘*’ 0.05 ‘.’ 0.1 ‘ ’ 1
```

Mixed-Effects Model for RR of TB Mortality Males vs. Females with Moderators = Prison + MDR

```
### fit mixed-effects model with prison and mdr as moderators
res <- rma(yi, vi, mods = ~ prison.rel + mdr.rel, data=dat)
res

Mixed-Effects Model (k = 7; tau^2 estimator: REML)

tau^2 (estimated amount of residual heterogeneity):     0.0146 (SE =
0.0505)
tau (square root of estimated tau^2 value):             0.1207
I^2 (residual heterogeneity / unaccounted variability): 19.68%
H^2 (unaccounted variability / sampling variability):   1.24
R^2 (amount of heterogeneity accounted for):            0.00%

Test for Residual Heterogeneity:
QE(df = 4) = 4.2058, p-val = 0.3789
```

```
Test of Moderators (coefficients 2:3):
QM(df = 2) = 0.0352, p-val = 0.9825

Model Results:

            estimate       se     zval    pval     ci.lb    ci.ub
intrcpt       0.4975   0.3932   1.2653  0.2057   -0.2731   1.2682
prison.rel   -2.6283  14.5350  -0.1808  0.8565  -31.1164  25.8599
mdr.rel       0.9483   7.1018   0.1335  0.8938  -12.9710  14.8677

---
Signif. codes:  0 '***' 0.001 '**' 0.01 '*' 0.05 '.' 0.1 ' ' 1
```

Mixed-Effects Model for RR of TB Mortality Males vs. Females with Moderators = Prison + MonoDR

```
### fit mixed-effects model with prison and monodr as moderators
res <- rma(yi, vi, mods = ~ prison.rel + monodr.rel, data=dat)
res

Mixed-Effects Model (k = 4; tau^2 estimator: REML)

tau^2 (estimated amount of residual heterogeneity):     0 (SE =
0.0630)
tau (square root of estimated tau^2 value):             0
I^2 (residual heterogeneity / unaccounted variability): 0.00%
H^2 (unaccounted variability / sampling variability):   1.00
R^2 (amount of heterogeneity accounted for):            100.00%

Test for Residual Heterogeneity:
QE(df = 1) = 0.4868, p-val = 0.4853

Test of Moderators (coefficients 2:3):
QM(df = 2) = 3.3418, p-val = 0.1881

Model Results:

            estimate       se     zval    pval      ci.lb    ci.ub
intrcpt       1.5192   0.6560   2.3157  0.0206     0.2334   2.8051  *
prison.rel  -56.6191  31.0699  -1.8223  0.0684  -117.5150   4.2768  .
monodr.rel    6.3002   5.7613   1.0935  0.2742    -4.9917  17.5921

---
Signif. codes:  0 '***' 0.001 '**' 0.01 '*' 0.05 '.' 0.1 ' ' 1
```

Mixed-Effects Model for RR of TB Mortality Males vs. Females with Moderators = Otherrf + MDR

```
### fit mixed-effects model with otherrf and mdr as moderators
res <- rma(yi, vi, mods = ~ otherrf.rel + mdr.rel, data=dat)
res

Mixed-Effects Model (k = 7; tau^2 estimator: REML)

tau^2 (estimated amount of residual heterogeneity):     0 (SE =
0.0587)
tau (square root of estimated tau^2 value):             0
I^2 (residual heterogeneity / unaccounted variability): 0.00%
H^2 (unaccounted variability / sampling variability):   1.00
R^2 (amount of heterogeneity accounted for):            0.00%
```

```
Test for Residual Heterogeneity:
QE(df = 4) = 2.9869, p-val = 0.5600

Test of Moderators (coefficients 2:3):
QM(df = 2) = 0.5602, p-val = 0.7557

Model Results:

             estimate      se     zval    pval    ci.lb   ci.ub
intrcpt        0.3277  0.1965   1.6672  0.0955  -0.0575  0.7129  .
otherrf.rel   -0.0936  1.4360  -0.0652  0.9481  -2.9080  2.7209
mdr.rel        0.2487  0.9488   0.2621  0.7932  -1.6110  2.1084

---
Signif. codes:  0 '***' 0.001 '**' 0.01 '*' 0.05 '.' 0.1 ' ' 1
```

Mixed-Effects Model for RR of TB Mortality Males vs. Females with Moderators = Otherrf + MonoDR

```
### fit mixed-effects model with otherrf and monodr as moderators
res <- rma(yi, vi, mods = ~ otherrf.rel + monodr.rel, data=dat)
res

Mixed-Effects Model (k = 5; tau^2 estimator: REML)

tau^2 (estimated amount of residual heterogeneity):     0 (SE =
0.0966)
tau (square root of estimated tau^2 value):             0
I^2 (residual heterogeneity / unaccounted variability): 0.00%
H^2 (unaccounted variability / sampling variability):   1.00
R^2 (amount of heterogeneity accounted for):            0.00%

Test for Residual Heterogeneity:
QE(df = 2) = 1.1589, p-val = 0.5602

Test of Moderators (coefficients 2:3):
QM(df = 2) = 0.9384, p-val = 0.6255

Model Results:

             estimate      se     zval    pval    ci.lb    ci.ub
intrcpt        0.3832  0.3573   1.0726  0.2835  -0.3170   1.0834
otherrf.rel   -1.1642  1.2907  -0.9020  0.3670  -3.6939   1.3654
monodr.rel     2.5405  4.5338   0.5603  0.5752  -6.3456  11.4265

---
Signif. codes:  0 '***' 0.001 '**' 0.01 '*' 0.05 '.' 0.1 ' ' 1
```

Mixed-Effects Model for RR of TB Mortality Males vs. Females with Moderators = Alcohol + Drugs + Formertb

```
### fit mixed-effects model with alcohol, drugs and formertb as
moderators
res <- rma(yi, vi, mods = ~ alcohol.rel + drugs.rel + formertb.rel,
data=dat)
res

Mixed-Effects Model (k = 10; tau^2 estimator: REML)

tau^2 (estimated amount of residual heterogeneity):     0 (SE =
0.0192)
tau (square root of estimated tau^2 value):             0
```

```
I^2 (residual heterogeneity / unaccounted variability): 0.00%
H^2 (unaccounted variability / sampling variability):   1.00
R^2 (amount of heterogeneity accounted for):            100.00%

Test for Residual Heterogeneity:
QE(df = 6) = 2.6864, p-val = 0.8471

Test of Moderators (coefficients 2:4):
QM(df = 3) = 4.6676, p-val = 0.1978

Model Results:

              estimate      se     zval    pval    ci.lb    ci.ub
intrcpt         0.4196  0.0912   4.6000  <.0001   0.2408   0.5983
***
alcohol.rel     0.8087  1.4294   0.5658  0.5715  -1.9928   3.6102
drugs.rel       0.5140  2.0514   0.2505  0.8022  -3.5068   4.5347
formertb.rel   -1.2493  0.6305  -1.9814  0.0475  -2.4851  -0.0135
*

---
Signif. codes:  0 ‘***’ 0.001 ‘**’ 0.01 ‘*’ 0.05 ‘.’ 0.1 ‘ ’ 1
```

Mixed-Effects Model for RR of TB Mortality Males vs. Females with Moderators = Alcohol + Drugs + Prison

```
### fit mixed-effects model with alcohol, drugs and prison as
moderators
res <- rma(yi, vi, mods = ~ alcohol.rel + drugs.rel + prison.rel,
data=dat)
res

Mixed-Effects Model (k = 7; tau^2 estimator: REML)

tau^2 (estimated amount of residual heterogeneity):     0 (SE =
0.0352)
tau (square root of estimated tau^2 value):             0
I^2 (residual heterogeneity / unaccounted variability): 0.00%
H^2 (unaccounted variability / sampling variability):   1.00
R^2 (amount of heterogeneity accounted for):            0.00%

Test for Residual Heterogeneity:
QE(df = 3) = 0.8233, p-val = 0.8439

Test of Moderators (coefficients 2:4):
QM(df = 3) = 2.9145, p-val = 0.4050

Model Results:

             estimate      se     zval    pval    ci.lb   ci.ub
intrcpt        0.2439  0.1711   1.4259  0.1539  -0.0914  0.5792
alcohol.rel    0.8229  0.9871   0.8336  0.4045  -1.1117  2.7574
drugs.rel      0.6654  1.4209   0.4683  0.6396  -2.1196  3.4503
prison.rel    -0.7451  2.8294  -0.2633  0.7923  -6.2905  4.8004

---
Signif. codes:  0 ‘***’ 0.001 ‘**’ 0.01 ‘*’ 0.05 ‘.’ 0.1 ‘ ’ 1
```

Mixed-Effects Model for RR of TB Mortality Males vs. Females with Moderators = Alcohol + Drugs + Otherrf

```
### fit mixed-effects model with alcohol, drugs and otherrf as
moderators
res <- rma(yi, vi, mods = ~ alcohol.rel + drugs.rel + otherrf.rel,
data=dat)
res

Mixed-Effects Model (k = 7; tau^2 estimator: REML)

tau^2 (estimated amount of residual heterogeneity):     0 (SE =
0.0684)
tau (square root of estimated tau^2 value):             0
I^2 (residual heterogeneity / unaccounted variability): 0.00%
H^2 (unaccounted variability / sampling variability):   1.00
R^2 (amount of heterogeneity accounted for):            0.00%

Test for Residual Heterogeneity:
QE(df = 3) = 1.8034, p-val = 0.6142

Test of Moderators (coefficients 2:4):
QM(df = 3) = 0.7720, p-val = 0.8562

Model Results:

             estimate      se     zval    pval    ci.lb    ci.ub
intrcpt        0.3410  0.3417   0.9981  0.3182  -0.3286   1.0107
alcohol.rel    2.0860  4.9921   0.4179  0.6760  -7.6984  11.8704
drugs.rel     -1.5305  2.7580  -0.5549  0.5789  -6.9362   3.8751
otherrf.rel   -1.4892  2.3852  -0.6244  0.5324  -6.1640   3.1856

---
Signif. codes:  0 ‘***’ 0.001 ‘**’ 0.01 ‘*’ 0.05 ‘.’ 0.1 ‘ ’ 1
```

Mixed-Effects Model for RR of TB Mortality Males vs. Females with Moderators = Alcohol + Drugs + MDR

```
### fit mixed-effects model with alcohol, drugs and mdr as moderators
res <- rma(yi, vi, mods = ~ alcohol.rel + drugs.rel + mdr.rel,
data=dat)
res

Mixed-Effects Model (k = 8; tau^2 estimator: REML)

tau^2 (estimated amount of residual heterogeneity):     0 (SE =
0.0339)
tau (square root of estimated tau^2 value):             0
I^2 (residual heterogeneity / unaccounted variability): 0.00%
H^2 (unaccounted variability / sampling variability):   1.00
R^2 (amount of heterogeneity accounted for):            0.00%

Test for Residual Heterogeneity:
QE(df = 4) = 1.3141, p-val = 0.8590

Test of Moderators (coefficients 2:4):
QM(df = 3) = 4.4169, p-val = 0.2198

Model Results:

             estimate      se     zval    pval    ci.lb   ci.ub
intrcpt        0.2634  0.1530   1.7215  0.0852  -0.0365  0.5633  .
alcohol.rel    1.3225  0.8686   1.5226  0.1279  -0.3799  3.0248
drugs.rel      0.0288  1.2141   0.0237  0.9811  -2.3509  2.4084
```

```
mdr.rel       -2.5047  2.1969  -1.1401  0.2542  -6.8105  1.8011

---
Signif. codes:  0 ‘***’ 0.001 ‘**’ 0.01 ‘*’ 0.05 ‘.’ 0.1 ‘ ’ 1
```

Mixed-Effects Model for RR of TB Mortality Males vs. Females with Moderators = Alcohol + Drugs + MonoDR

```
### fit mixed-effects model with alcohol, drugs and monodr as
moderators
res <- rma(yi, vi, mods = ~ alcohol.rel + drugs.rel + monodr.rel,
data=dat)
res

Mixed-Effects Model (k = 6; tau^2 estimator: REML)

tau^2 (estimated amount of residual heterogeneity):     0 (SE =
0.1010)
tau (square root of estimated tau^2 value):             0
I^2 (residual heterogeneity / unaccounted variability): 0.00%
H^2 (unaccounted variability / sampling variability):   1.00
R^2 (amount of heterogeneity accounted for):            100.00%

Test for Residual Heterogeneity:
QE(df = 2) = 1.4191, p-val = 0.4919

Test of Moderators (coefficients 2:4):
QM(df = 3) = 3.0141, p-val = 0.3895

Model Results:

             estimate      se     zval    pval    ci.lb   ci.ub
intrcpt        0.2832  0.1627   1.7406  0.0818  -0.0357  0.6021  .
alcohol.rel    0.7718  3.7395   0.2064  0.8365  -6.5576  8.1011
drugs.rel      0.5752  4.7739   0.1205  0.9041  -8.7815  9.9319
monodr.rel    -0.5412  0.4977  -1.0874  0.2769  -1.5168  0.4343

---
Signif. codes:  0 ‘***’ 0.001 ‘**’ 0.01 ‘*’ 0.05 ‘.’ 0.1 ‘ ’ 1
```

Mixed-Effects Model for RR of TB Mortality Males vs. Females with Moderators = Alcohol + Smoker + Drugs

```
### fit mixed-effects model with alcohol, drugs and smoker as
moderators
res <- rma(yi, vi, mods = ~ alcohol.rel + smoker.rel + drugs.rel,
data=dat)
res

Mixed-Effects Model (k = 7; tau^2 estimator: REML)

tau^2 (estimated amount of residual heterogeneity):     0 (SE =
0.0449)
tau (square root of estimated tau^2 value):             0
I^2 (residual heterogeneity / unaccounted variability): 0.00%
H^2 (unaccounted variability / sampling variability):   1.00
R^2 (amount of heterogeneity accounted for):            0.00%

Test for Residual Heterogeneity:
QE(df = 3) = 0.9806, p-val = 0.8060

Test of Moderators (coefficients 2:4):
```

```
QM(df = 3) = 3.2953, p-val = 0.3483

Model Results:

             estimate      se     zval    pval    ci.lb    ci.ub
intrcpt        0.5073  0.2437   2.0815  0.0374   0.0296   0.9849  *
alcohol.rel    1.2492  1.7947   0.6960  0.4864  -2.2685   4.7668
smoker.rel    -2.7348  1.8816  -1.4534  0.1461  -6.4226   0.9531
drugs.rel      4.8347  3.5727   1.3532  0.1760  -2.1677  11.8371

---
Signif. codes:  0 '***' 0.001 '**' 0.01 '*' 0.05 '.' 0.1 ' ' 1
```

Mixed-Effects Model for RR of TB Mortality Males vs. Females with Moderators = Alcohol + Smoker + Formertb

```
### fit mixed-effects model with alcohol, smoker and formertb as
moderators
res <- rma(yi, vi, mods = ~ alcohol.rel + smoker.rel + formertb.rel,
data=dat)
res

Mixed-Effects Model (k = 8; tau^2 estimator: REML)

tau^2 (estimated amount of residual heterogeneity):     0 (SE =
0.0612)
tau (square root of estimated tau^2 value):             0
I^2 (residual heterogeneity / unaccounted variability): 0.00%
H^2 (unaccounted variability / sampling variability):   1.00
R^2 (amount of heterogeneity accounted for):            0.00%

Test for Residual Heterogeneity:
QE(df = 4) = 3.1912, p-val = 0.5264

Test of Moderators (coefficients 2:4):
QM(df = 3) = 1.6441, p-val = 0.6494

Model Results:

              estimate      se     zval    pval    ci.lb   ci.ub
intrcpt         0.4042  0.2531   1.5971  0.1102  -0.0918  0.9001
alcohol.rel     1.1483  1.0845   1.0588  0.2897  -0.9773  3.2739
smoker.rel     -0.2446  1.0335  -0.2367  0.8129  -2.2702  1.7809
formertb.rel   -0.8693  0.7508  -1.1579  0.2469  -2.3408  0.6022

---
Signif. codes:  0 '***' 0.001 '**' 0.01 '*' 0.05 '.' 0.1 ' ' 1
```

Mixed-Effects Model for RR of TB Mortality Males vs. Females with Moderators = Alcohol + Smoker + Otherrf

```
### fit mixed-effects model with alcohol, smoker and otherrf as
moderators
res <- rma(yi, vi, mods = ~ alcohol.rel + smoker.rel + otherrf.rel,
data=dat)
res

Mixed-Effects Model (k = 7; tau^2 estimator: REML)

tau^2 (estimated amount of residual heterogeneity):     0 (SE =
0.1015)
tau (square root of estimated tau^2 value):             0
```

```
I^2 (residual heterogeneity / unaccounted variability): 0.00%
H^2 (unaccounted variability / sampling variability):   1.00
R^2 (amount of heterogeneity accounted for):            0.00%

Test for Residual Heterogeneity:
QE(df = 3) = 1.2822, p-val = 0.7334

Test of Moderators (coefficients 2:4):
QM(df = 3) = 1.8386, p-val = 0.6066

Model Results:

             estimate      se     zval    pval    ci.lb   ci.ub
intrcpt        0.3664  0.2176   1.6839  0.0922  -0.0601  0.7928  .
alcohol.rel    0.5741  2.4827   0.2312  0.8171  -4.2919  5.4400
smoker.rel    -1.1540  1.0868  -1.0618  0.2883  -3.2841  0.9762
otherrf.rel    0.6602  1.9919   0.3315  0.7403  -3.2439  4.5643

---
Signif. codes:  0 ‘***’ 0.001 ‘**’ 0.01 ‘*’ 0.05 ‘.’ 0.1 ‘ ’ 1
```

Mixed-Effects Model for RR of TB Mortality Males vs. Females with Moderators = Alcohol + Nohome + Drugs

```
### fit mixed-effects model with alcohol, nohome and drugs as
moderators
res <- rma(yi, vi, mods = ~ alcohol.rel + nohome.rel + drugs.rel,
data=dat)
res

Mixed-Effects Model (k = 7; tau^2 estimator: REML)

tau^2 (estimated amount of residual heterogeneity):     0 (SE =
0.0365)
tau (square root of estimated tau^2 value):             0
I^2 (residual heterogeneity / unaccounted variability): 0.00%
H^2 (unaccounted variability / sampling variability):   1.00
R^2 (amount of heterogeneity accounted for):            100.00%

Test for Residual Heterogeneity:
QE(df = 3) = 0.5410, p-val = 0.9098

Test of Moderators (coefficients 2:4):
QM(df = 3) = 4.5809, p-val = 0.2052

Model Results:

             estimate       se     zval    pval     ci.lb    ci.ub
intrcpt        0.3150   0.1161   2.7140  0.0066    0.0875   0.5425
**
alcohol.rel    3.8509   3.2554   1.1829  0.2368   -2.5296  10.2314
nohome.rel   -14.8528  15.3587  -0.9671  0.3335  -44.9554  15.2498
drugs.rel      2.0987   4.9918   0.4204  0.6742   -7.6850  11.8825

---
Signif. codes:  0 ‘***’ 0.001 ‘**’ 0.01 ‘*’ 0.05 ‘.’ 0.1 ‘ ’ 1
```

Mixed-Effects Model for RR of TB Mortality Males vs. Females with Moderators = Alcohol + Nohome + Formertb

```
### fit mixed-effects model with alcohol, nohome and formertb as
moderators
```

```
res <- rma(yi, vi, mods = ~ alcohol.rel + nohome.rel + formertb.rel,
data=dat)
res

Mixed-Effects Model (k = 5; tau^2 estimator: REML)

tau^2 (estimated amount of residual heterogeneity):     0 (SE =
0.1834)
tau (square root of estimated tau^2 value):             0
I^2 (residual heterogeneity / unaccounted variability): 0.00%
H^2 (unaccounted variability / sampling variability):   1.00
R^2 (amount of heterogeneity accounted for):            100.00%

Test for Residual Heterogeneity:
QE(df = 1) = 0.5206, p-val = 0.4706

Test of Moderators (coefficients 2:4):
QM(df = 3) = 3.1348, p-val = 0.3713

Model Results:

              estimate      se     zval    pval     ci.lb    ci.ub
intrcpt         0.3691  0.1574   2.3445  0.0191    0.0605   0.6777  *
alcohol.rel     1.5212  1.9204   0.7921  0.4283   -2.2428   5.2852
nohome.rel     -0.6739  6.4760  -0.1041  0.9171  -13.3666  12.0188
formertb.rel   -1.2908  1.4635  -0.8820  0.3778   -4.1592   1.5777

---
Signif. codes:  0 ‘***’ 0.001 ‘**’ 0.01 ‘*’ 0.05 ‘.’ 0.1 ‘ ’ 1
```

Mixed-Effects Model for RR of TB Mortality Males vs. Females with Moderators = Alcohol + Nohome + MDR

```
### fit mixed-effects model with alcohol, nohome and mdr as
moderators
res <- rma(yi, vi, mods = ~ alcohol.rel + nohome.rel + mdr.rel,
data=dat)
res

Mixed-Effects Model (k = 6; tau^2 estimator: REML)

tau^2 (estimated amount of residual heterogeneity):     0 (SE =
0.0443)
tau (square root of estimated tau^2 value):             0
I^2 (residual heterogeneity / unaccounted variability): 0.00%
H^2 (unaccounted variability / sampling variability):   1.00
R^2 (amount of heterogeneity accounted for):            0.00%

Test for Residual Heterogeneity:
QE(df = 2) = 0.1028, p-val = 0.9499

Test of Moderators (coefficients 2:4):
QM(df = 3) = 3.9119, p-val = 0.2711

Model Results:

             estimate      se     zval    pval     ci.lb   ci.ub
intrcpt        0.2930  0.1466   1.9982  0.0457    0.0056  0.5803  *
alcohol.rel    4.0895  2.7625   1.4803  0.1388   -1.3249  9.5039
nohome.rel    -9.8907  9.4890  -1.0423  0.2973  -28.4889  8.7074
mdr.rel       -1.6930  1.1366  -1.4895  0.1364   -3.9207  0.5347

---
```

```
Signif. codes:  0 ‘***’ 0.001 ‘**’ 0.01 ‘*’ 0.05 ‘.’ 0.1 ‘ ’ 1
```

Mixed-Effects Model for RR of TB Mortality Males vs. Females with Moderators = Alcohol + Formertb + Otherrf

```
### fit mixed-effects model with alcohol, formertb and otherrf as
moderators
res <- rma(yi, vi, mods = ~ alcohol.rel + formertb.rel + otherrf.rel,
data=dat)
res

Mixed-Effects Model (k = 7; tau^2 estimator: REML)

tau^2 (estimated amount of residual heterogeneity):     0.0336 (SE =
0.1382)
tau (square root of estimated tau^2 value):             0.1834
I^2 (residual heterogeneity / unaccounted variability): 19.65%
H^2 (unaccounted variability / sampling variability):   1.24
R^2 (amount of heterogeneity accounted for):            0.00%

Test for Residual Heterogeneity:
QE(df = 3) = 3.2890, p-val = 0.3492

Test of Moderators (coefficients 2:4):
QM(df = 3) = 0.0316, p-val = 0.9985

Model Results:

              estimate      se     zval    pval    ci.lb   ci.ub
intrcpt         0.3433  0.2940   1.1680  0.2428  -0.2328  0.9195
alcohol.rel     0.0428  2.3192   0.0185  0.9853  -4.5028  4.5884
formertb.rel   -0.1414  1.5433  -0.0916  0.9270  -3.1661  2.8833
otherrf.rel     0.1337  2.2302   0.0599  0.9522  -4.2375  4.5049

---
Signif. codes:  0 ‘***’ 0.001 ‘**’ 0.01 ‘*’ 0.05 ‘.’ 0.1 ‘ ’ 1
```

Mixed-Effects Model for RR of TB Mortality Males vs. Females with Moderators = Alcohol + Formertb + MDR

```
### fit mixed-effects model with alcohol, formertb and mdr as
moderators
res <- rma(yi, vi, mods = ~ alcohol.rel + formertb.rel + mdr.rel,
data=dat)
res

Mixed-Effects Model (k = 6; tau^2 estimator: REML)

tau^2 (estimated amount of residual heterogeneity):     0 (SE =
0.0957)
tau (square root of estimated tau^2 value):             0
I^2 (residual heterogeneity / unaccounted variability): 0.00%
H^2 (unaccounted variability / sampling variability):   1.00
R^2 (amount of heterogeneity accounted for):            0.00%

Test for Residual Heterogeneity:
QE(df = 2) = 1.2067, p-val = 0.5470

Test of Moderators (coefficients 2:4):
QM(df = 3) = 2.6505, p-val = 0.4487

Model Results:
```

```
              estimate      se     zval    pval    ci.lb   ci.ub
intrcpt         0.3183  0.2279   1.3966  0.1625  -0.1284  0.7649
alcohol.rel     1.4101  0.9825   1.4352  0.1512  -0.5156  3.3357
formertb.rel   -1.0695  1.2647  -0.8457  0.3977  -3.5484  1.4093
mdr.rel        -0.0646  0.8232  -0.0785  0.9375  -1.6781  1.5489

---
Signif. codes:  0 ‘***’ 0.001 ‘**’ 0.01 ‘*’ 0.05 ‘.’ 0.1 ‘ ’ 1
```
