# Supplementary material for: Tuberculosis case fatality is higher in male than female patients in Europe: a systematic review and meta-analysis
Source: Infection. 2024 Mar 23;52(5):1775–86. doi: 10.1007/s15010-024-02206-z (PMC11499538; doi:10.1007/s15010-024-02206-z)
Supplement: Supplementary file 31 — Online Resource 31 Listing of the random sample of 14 publications for risk of bias assessment (by a second reviewer) (PDF 185 KB) [file 15010_2024_2206_MOESM31_ESM.pdf]

Random sample of 14 publications for risk of bias assessment

1. Balabanova, Y., V. Nikolayevskyy, O. Ignatyeva, I. Kontsevaya, C. M. Rutterford, and A. Shakhmistova. 2011. “Survival of Civilian and Prisoner Drug-Sensitive, Multi- and Extensive Drug-Resistant Tuberculosis Cohorts Prospectively Followed in Russia” 6: e20531.

2. Bhering, M., A. Kritski, C. Nunes, and R. Duarte. 2019. “Multidrug-Resistant Tuberculosis in Lisbon: Unfavourable Treatment and Associated Factors, 2000–2014.” International Journal of Tuberculosis and Lung Disease 23 (10): 1075–81.

3. Brodhun, B., D. Altmann, B. Hauer, L. Fiebig, and W. Haas. n.d. “Current Epidemiology of Tuberculosis in Germany.” Pneumologie 69 (5): 263–70.

4. Daucourt, V., S. Elia-Pasquet, L. Portel, S. Petit-Carrié, G. Courty, M. Dupon, J. Texier-Maugein, et al. 2000. “Follow-up of Tuberculosis Patients and Link with HIV Infection in a French District, Gironde, 1995-1996.” Medecine et Maladies Infectieuses 30 (3): 152–61.

5. Dobrotková, A. 2019. “Impact of Sociodemographic and Clinical Risk Factors on Pulmonary Tuberculosis Treatment Outcome in the Slovak Republic in 2007-2016.” Studia Pneumologica et Phthiseologica 79 (1): 17–24.

6. Helbling, P., C. Medinger, E. Altpeter, P. A. Raeber, D. Beeli, and J. P. Zellweger. n.d. “Outcome of Treatment of Pulmonary Tuberculosis in Switzerland in 1996.” Swiss Medical Weekly 132 (35): 517–22.

7. Jamilloux, Y., M. B. Delphine, S. Kerever, M. Gerfaud-Valentin, C. Broussolle, M. Eb, D. Valeyre, and P. Seve. 2016. “Sarcoidosis-Related Mortality in France: A Multiple-Cause-of-Death Analysis.” European Respiratory Journal 48 (6): 1700–1709.

8. Kleina, R., O. Fjodorova, A. Dabužinskienė, J. Nazarovs, and O. Mahmajeva. 2017. “Multiform Spectrum of Pulmonary Disease in Lethal HIV Infection Cases in Latvia (2012-2016).” Papers on Anthropology 26 (2): 53–67.

9. Loytved, G., B. Steidle, E. Benz, and W. Koszczynski. 2002. “[Tuberculosis control in lower Franconia 1995 - 2001. Case-finding and treatment outcome].” Pneumologie (Stuttgart, Germany) 56 (6): 349–56.

10. Milanov, V., D. Falzon, M. Zamfirova, T. Varleva, E. Bachiyska, A. Koleva, and M. Dara. n.d. “Factors Associated with Treatment Success and Death in Cases with Multidrug-Resistant Tuberculosis in Bulgaria, 2009-2010.” International Journal of Mycobacteriology 4 (2): 131–37.

11. Millet, J., A. Orcau, M. Casals, P. Garcia De Olalla, C. Rius, and J. Cayla. 2010. “Predictors of Death among Patients WHO Completed Tuberculosis Treatment.” American Journal of Respiratory and Critical Care Medicine 181 (1).

12. Nebreda-Mayoral, T., M. F. Brezmes-Valdivieso, N. Gutierrez-Zufiaurre, S. Garcia-de Cruz, C. Labayru-Echeverria, R. Lopez-Medrano, L. Lopez-Urrutia-Lorente, A. Tinajas-Puertas, and O. Rivero-Lezcano. n.d. “Human Mycobacterium Bovis Infection in Castile and Leon (Spain), 2006-2015.” Enfermedades Infecciosas y Microbiologia Clinica 37 (1): 19–24.

13. “Report on the Epidemiology of Tuberculosis in Germany 2018.” 2019. Berlin: Robert Koch-Institut.

14. Vasankari, T., P. Holmstrom, J. Ollgren, K. Liippo, M. Kokki, and P. Ruutu. n.d. “Risk Factors for Poor Tuberculosis Treatment Outcome in Finland: A Cohort Study.” BMC Public Health 7: 291.
